# Rhodium(II)-Catalyzed Asymmetric Cyclopropanation and Desymmetrization of [2.2]Paracyclophanes

**DOI:** 10.1021/acscatal.4c01292

**Published:** 2024-04-11

**Authors:** Duc Ly, John Bacsa, Huw M. L. Davies

**Affiliations:** Department of Chemistry, Emory University, 1515 Dickey Drive, Atlanta, Georgia 30322, United States

**Keywords:** paracyclophane, rhodium carbene, Büchner reaction, desymmetrization, asymmetric catalysis

## Abstract

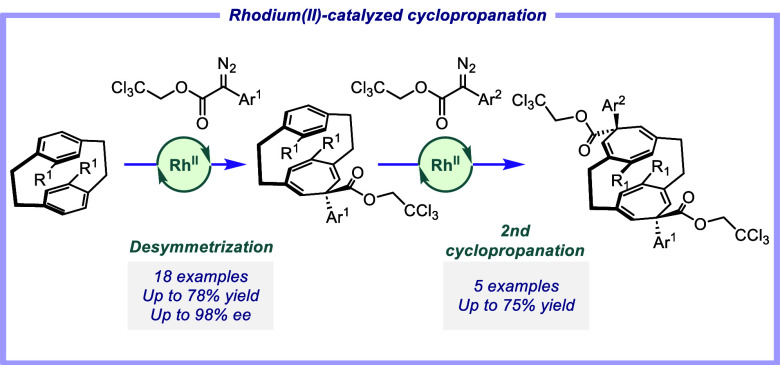

Chiral [2.2]paracyclophane derivatives are of considerable interest because of their potential in asymmetric catalysis and the development of chiral materials. This study describes the scope of rhodium-catalyzed reactions of aryldiazoacetates with [2.2]paracyclophanes. The reaction with the parent [2.2]paracyclophane resulted in cyclopropanation at two positions, the ratio of which is catalyst-controlled. Because of the strain in the system, one of the cyclopropanes exists primarily as the norcaradiene structure, whereas the other preferentially exists as the cycloheptatriene conformer. In contrast, the reaction with [3.3]paracyclophane results in benzylic C–H functionalization. The reactions with substituted [2.2]paracyclophanes using chiral catalysts can result in either kinetic resolution or desymmetrization. The Rh_2_(*S*-*p*-PhTPCP)]_4_-catalyzed reaction of monosubstituted paracyclophanes results in kinetic resolution with a selectivity (*s*) factor of up to 20, whereas reactions on C_2v_-symmetric disubstituted [2.2]paracyclophanes with Rh_2_(*S*-TPPTTL)_4_ [TPPTTL = 2-(1,3-dioxo-4,5,6,7-tetraphenylisoindolin-2-yl)-3,3-dimethylbutanoate] results in effective desymmetrization to form cycloheptatriene-incorporated paracyclophanes in 78–98% ee.

## Introduction

**Scheme 1:**
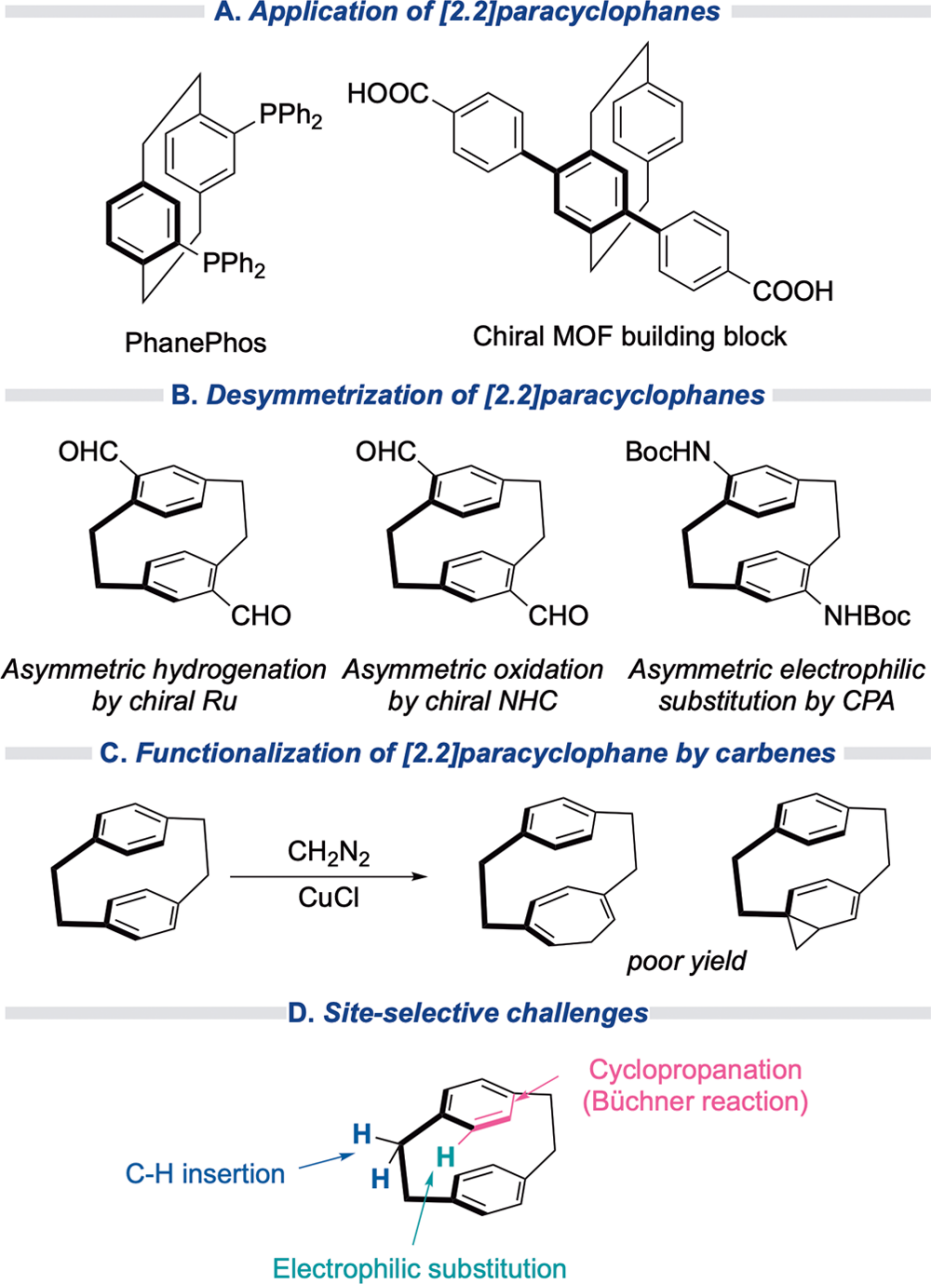
Introduction to [2.2]Paracyclophanes: (A) Application of [2.2]Paracyclophanes,^4−6^ (B) Desymmetrization of [2.2]Paracyclophanes,^9,13,14^ (C) Functionalization of [2.2]Paracyclophanes by Carbenes,^15,16^ and (D) Site-Selective Challenges

[2.2]Paracyclophanes, discovered by Brown and Farthing in 1949, have generated considerable interest because they are strained compounds as evidenced by the bent nature of the aromatic rings.^1^ Furthermore, even though the parent structure is achiral, the introduction of additional functionality can generate derivatives with planar chirality. These chiral derivatives have been used in numerous applications, including chiral ligands for asymmetric synthesis, such as PhanePhos,^2,3^ and as chiral components in new materials, such as metal–organic frameworks (MOFs)^4^ and circularly polarized luminescence (CPL) emitters^5,6^ (Scheme 1A). Consequently, the development of efficient synthesis of complex paracyclophanes has generated considerable interest.^7^ Even so, the asymmetric synthesis of functionalized paracyclophanes remains a significant challenge. Typically, enantiomerically pure [2.2]paracyclophanes are produced either by resolution of diastereomeric derivatives^2,8^ or by kinetic resolution.^9−12^ In recent years, new approaches using desymmetrization or dynamic kinetic resolution have been developed (Scheme 1B). These include desymmetrization of *meso*-diformyl[2.2]paracyclophanes by oxidation^13^ or reduction^14^ and of diamido[2.2]paracyclophanes by asymmetric electrophilic substitution.^9^

In this study, we examined whether the enantioselective reaction of donor/acceptor carbenes with paracyclophanes would result in the ready generation of novel paracyclophane derivatives. Previous studies on the reaction of [2.2]paracyclophane with diazomethane resulted in the formation of a mixture of mono-, di-, tri-, and polysubstituted derivatives, including two regioisomeric monosubstituted products in undefined yields (Scheme 1C).^15,16^ The only effective carbene example of C–H functionalization involved an intramolecular process that required considerable effort to generate the carbene precursor.^17^ The plan here was to use rhodium-stabilized donor/acceptor carbenes as the reactive intermediates in intermolecular reactions and use catalysts to control the reaction outcome (Scheme 1D). We have shown that donor/acceptor carbenes are far more selective than other types of carbenes,^18^ and numerous dirhodium catalysts have been designed that modulate site selectivity and are capable of very high levels of asymmetric induction.^19^ Enantioselective benzylic C–H functionalization by donor/acceptor carbenes is well established, and if it can be conducted on the parent paracyclophane, then it would offer a one-step entry into chiral derivatives. Normally, a 1,4-disubtitued benzene ring is sterically protected from cyclopropanation with donor/acceptor carbenes,^20−22^ but the steric strain in paracyclophanes was expected to challenge this standard reactivity profile.

## Results and Discussion

The study began by examining the reaction of [2.2]paracyclophane (**1a**) with 2,2,2-trichloroethyl 2-(4-bromophenyl)-2-diazoacetate (**2a**) (1.2 equiv) at 25 °C to gain an initial understanding of the reactivity profile of donor/acceptor carbenes toward [2.2]paracyclophanes (Table 1). The reaction was conducted with a series of standard achiral dirhodium catalysts. In each case, a mixture of regioisomers (**3aa** and **4aa**) of cyclopropanation (Büchner reaction)^23a−23e^ products was observed with high levels of diastereoselectivity. In no instance was the C(sp^3^)–H functionalization product observed. As previously seen in the reaction with diazomethane,^15,16^**3aa** derived from cyclopropanation of the C2–C3 bond preferentially exists in the cycloheptatriene conformer, whereas **4aa** derived from cyclopropanation of the C1–C2 bond preferentially exists in the norcaradiene conformer. The reason for the change in the cycloheptatriene/norcaradiene ratio is likely due to the influence of hybridization [C(sp^2^) versus C(sp^3^)] on the strain associated with the paracyclophanes. If **4aa** existed in the cycloheptatriene form, it would add considerable strain to the paracyclophane, and thus, the equilibrium in this case prefers the norcaradiene form.

**Table 1:**
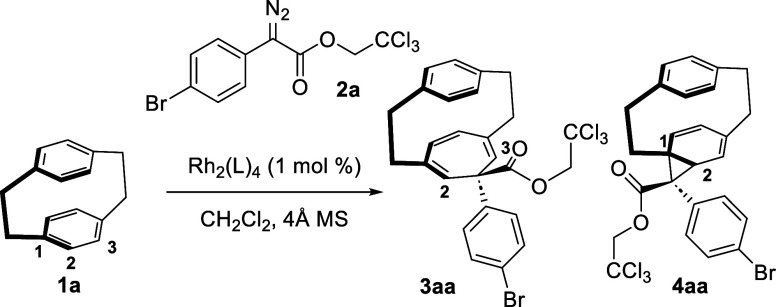
Regioselective Cyclopropanation of [2.2]Paracyclophane (**1a**)

entry^a^	**1a**/**2a**	catalyst	yield, %^b^	rr^c^ (**3aa**/**4aa**)
1	1:1.2	Rh_2_(OPiv)_4_	42	1:2
2	1:1.2	Rh_2_(TPA)_4_	54	1:5
3	1:1.2	Rh_2_(esp)_2_	48	1:3
4	1:1.2	Rh_2_(TFA)_4_	28	1:1
5	1:1.2	Rh_2_(OAc)_4_	44	4:1
6	1:1.2	Rh_2_(OBz)_4_	54	4:1
7^d^	2:1	Rh_2_(OBz)_4_	58	6:1
8^d^	3:1	Rh_2_(OBz)_4_	74	8:1

a Reaction conditions: **1a** (0.2 mmol), **2a**, 1 mol % Rh_2_(L)_4_, 4 Å molecular sieves in CH_2_Cl_2_ (0.1 M), 25 °C, 1 h slow additions.

b Combined ^1^H NMR yields using 1,1,2,2-tetrachloroethane as an internal standard.

c rr was determined by ^1^H NMR.

d Reaction ran at 39 °C.

The regioselectivity changes dramatically upon use of different catalysts. While the relatively more sterically demanding catalysts, including Rh_2_(esp)_2_, Rh_2_(TPA)_4_, and Rh_2_(OPiv)_4_, favored the formation of norcaradiene **4aa** (Table 1, entries 1–3), cycloheptatriene **3aa** is favored when using the less crowded catalysts (entries 4–6). The use of 1.2 equiv of **2a** resulted in the formation of numerous byproducts, which is attributed to the further reaction of **3aa** and **4aa** with **2a**. Cleaner reactions were obtained using the carbene precursor **2a** as the limiting reagent, and when the Rh_2_(OBz)_4_-catalyzed^24^ reaction was conducted in refluxing dichloromethane, **3aa** was formed in 74% yield, with an 8:1 regioselective ratio (rr) (entry 8).

Having established that Rh_2_(OBz)_4_ is the optimum achiral catalyst for selective cyclopropanation at C2–C3, the Rh_2_(OBz)_4_-catalyzed reaction of **1a** was examined with a range of donor/acceptor carbenes (Table 2). Generally, the selectivity and yield of the reactions were found to be moderate, as illustrated in the formation of **3aa**–**3aj** in 25–66% yield with regioselectivity ranging from 2:1 to 8:1. The products **3aa**–**3aj** are meso compounds, but there is the possibility of forming two diastereomers. Only the diastereomer with the aryl group pointing away from the paracyclophane was formed. Under standard conditions, the reaction of **1a** with **2a** afforded the desired product **3aa** in 65% yield with a regioselectivity of 8:1. Electron-donating groups, such as Ph (**3ab**), *t-*Bu (**3ac**), OMe (**3ad**), and H (**3ae**), are compatible, although the reactions tend to proceed in lower yields (37–57%). However, the reaction occurred smoothly with aryldiazoacetates containing electron-withdrawing groups, such as NO_2_ to form **3af** in 51% yield and CF_3_ to form **3ag** in 55% yield. Meta substituents were similarly compatible with the reaction and formed **3ah**, **3ai**, and **3aj** in 41–47% yield. Different ring systems, such as naphthalene and pyridine, were also compatible and afforded **3ak** and **3al** in 57% and 35% yield, respectively. It is worth noting that the yield of this transformation is moderate because of inefficient trapping of the carbene by **1a**, which led to carbene dimer formation rather than other side reactions occurring between the carbene and the paracyclophane.

**Table 2:**
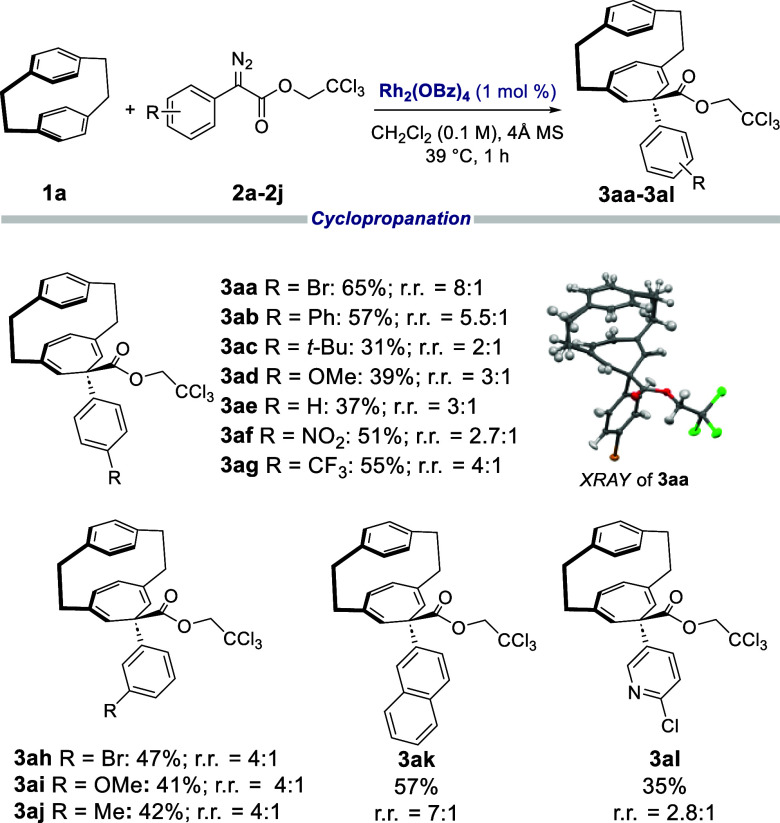
Reaction of [2.2]Paracyclophane (**1a**) with Various Aryldiazoacetates^a^

a Reaction conditions: **2** (0.2 mmol), **1a** (0.6 mmol), and Rh_2_(OBz)_4_ (1 mol %) in CH_2_Cl_2_ (2 mL) with 4 Å molecular sieves (100 wt %) at 39 °C, 1 h slow addition. Yields refer to isolated yields.

**Scheme 2:**
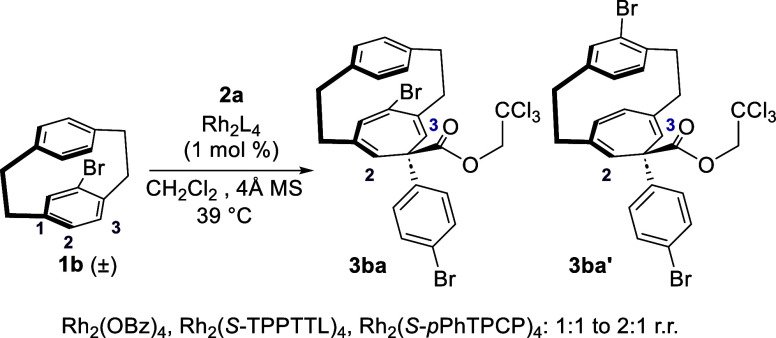
Exploratory Kinetic Resolution

Having established with achiral catalysts that the Büchner reaction of [2.2]paracyclophane (**1a**) with donor/acceptor carbenes is a viable process, we then began exploring the possibility of achieving asymmetric reactions using chiral catalysts. A few examples of enantioselective Büchner reactions are known but these reactions were with simple aromatic systems.^23a,23b^ The first series of experiments explored whether racemic monosubstituted paracyclophanes would be susceptible to kinetic resolution. This reaction is challenging because it would be necessary to differentiate between the two aryl rings and, furthermore, would need to proceed regioselectively (C2–C3 versus C1–C2) at the reacting aromatic ring. Brominated derivative **1b**^**2**^ was initially examined but was not very successful because an inseparable mixture of products was obtained (Scheme 2). Distinctive signals in the alkene region of the partially purified mixture revealed that a mixture of regioisomers **3ba** and **3ba**′ had been formed, which indicated that both rings are susceptible to cyclopropanation. A catalyst screen revealed that the ratio of the mixture remained relatively constant and varied between 1:1 and 2:1 ratio of **3ba** and **3ba**′.

**Scheme 3:**
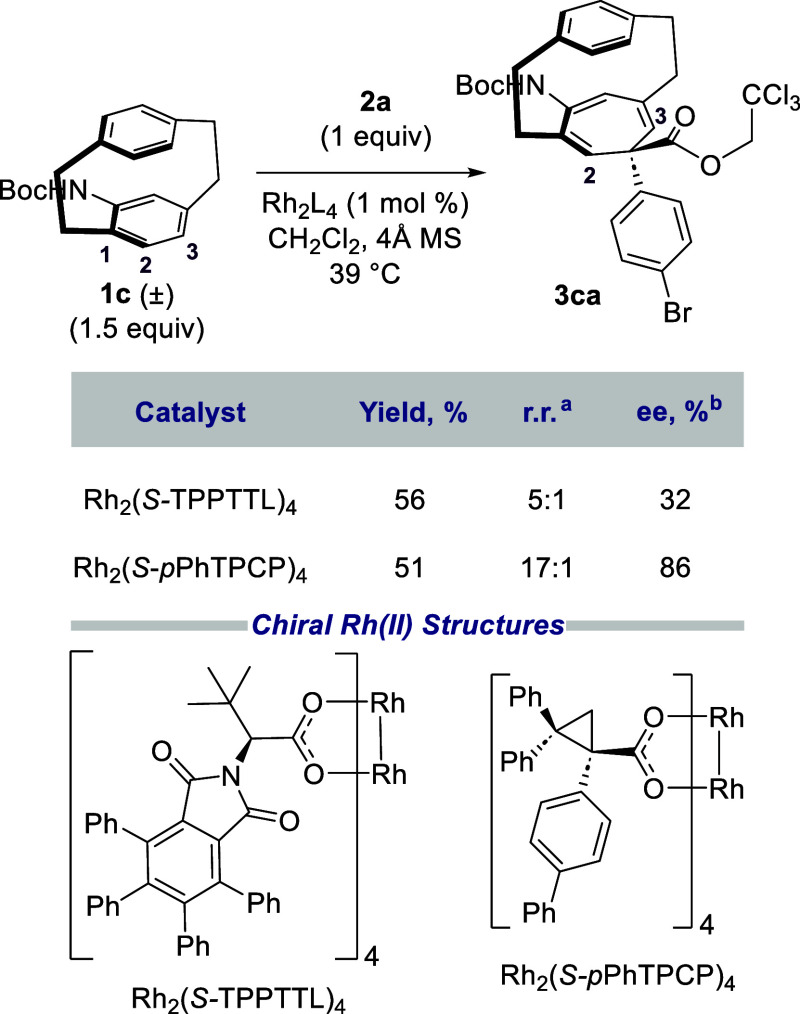
Optimized System for Kinetic Resolution rr was determined by ^1^H NMR 4.52 ppm (d, 1H); 5.70 ppm (s, 1H). ee was determined by SFC analysis.

In order to favor one ring over the other, the studies were extended to acetamido derivative **1c**^9^ (Scheme 3). In this case, the reaction went cleanly at the acetamide-functionalized ring. Because of the directing influence of the acetamido group, many of the catalysts gave a mixture of the C2–C3 (**3ca**) and the C1–C2 cyclopropanation products (see the Supporting Information for the full set of catalysts studied). For example, when the reaction was conducted with Rh_2_(*S*-TPPTTL)_4_ [TPPTTL = 2-(1,3-dioxo-4,5,6,7-tetraphenylisoindolin-2-yl)-3,3-dimethylbutanoate], one of our newer C_4_-symmetric bowl-shaped catalysts,^25^ the regioselectivity favoring **3ca** was only 5:1. When sterically bulky catalysts were used, such as Rh_2_(*S*-*p*-PhTPCP)_4_^26^ the reaction strongly preferred the C2–C3 product **3ca** with a 17:1 rr. Furthermore, **3ca** was obtained in 86% ee, which suggests a reasonably effective kinetic resolution. By measuring the enantioselectivity of recovered starting material **1c** (47% ee) and the overall conversion based on **1c** (35%), a selectivity (*s*) factor for the reaction was estimated to be 20. Optical rotation comparison showed that the recovered [2.2]paracyclophane **1c** was enriched in the (*R*_p_) enantiomer.^9^ Hence, product **3ca** is drawn as the product derived from the (*S*_f_) enantiomer of **1c**.

Even though the kinetic resolution was reasonably successful, it lacked broad generality because the functionality needed to be carefully selected to achieve the desired transformation and avoid the formation of a mixture of regioisomers. Therefore, we decided to alter the approach and explore the reactions of C_2v_-symmetric pseudo-*para*-disubstituted [2.2]paracyclophanes because the selection of which ring is functionalized would now lead to desymmetrization instead of formation of regioisomers. In order to evaluate this possibility, a catalyst screen was conducted with the dibromo[2.2]paracyclophanes **1d**.^2^ For a successful reaction to occur, it would be necessary to control which double bond in the paracyclophane is functionalized, in addition to achieving high levels of asymmetric induction. Therefore, a catalyst screen was conducted under standard conditions using **2a** as the carbene source (Table 3). The initial studies were not promising as the reference reaction with Rh_2_(OBz)_4_ resulted in low yield and regioselectivity (entry 1). The bulky catalyst, Rh_2_(*S*-*p*-PhTPCP)_4_, which had been the optimum catalyst for the kinetic resolution in Scheme 3, gave a low yield and no improvement in the regioselectivity (entry 2). Similarly poor performance was observed with some of our other established catalysts (entries 3–5).^19^ However, the C_4_-symmetric bowl-shaped catalyst, Rh_2_(*R*-TPPTTL)_4_, performed extremely well in the desymmetrization of **1d** to generate the desired product in >20:1 rr with enantioselectivity of 90% ee (entry 6). In all of the reactions to date, a substantial amount of carbene dimer was observed, presumably because paracyclophane **1d** is somewhat deactivated and is not effectively trapped by the carbene. Therefore, the stoichiometry of the reaction was changed to an excess of the diazo compound (1.5 equiv), and under these conditions, the desired product **3da** was formed in 77% yield and 95% ee (entry 7). This compound could be enantioenriched to 99% ee by recrystallization from hot hexane. A brief study of the influence of the ester group revealed that trichloroethyl ester gave superior yield to the trifluoroethyl and methyl ester derivatives (entries 8 and 9).

**Table 3:**
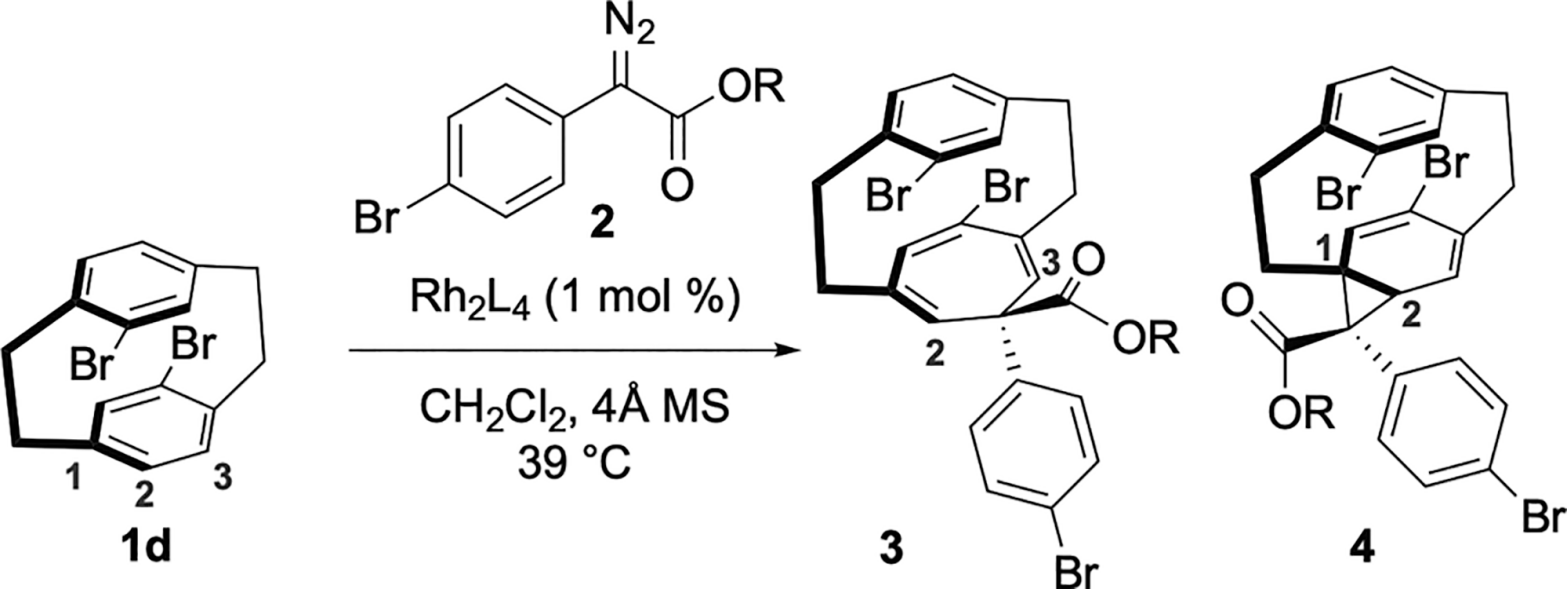
Optimization of Desymmetrization Reaction

entry	**1c**/**2**	R	catalyst	yield, %^b^	rr^c^ (**3**/**4**)	ee, % (**4**)^d^
1	2:1	CH_2_CCl_3_	Rh_2_(OBz)_4_	30	2:1	0
2	2:1	CH_2_CCl_3_	Rh_2_(*S*-*p*PhTPCP)_4_	18	7:1	3
3	2:1	CH_2_CCl_3_	Rh_2_(*R*-DOSP)_4_	32	5:1	0
4	2:1	CH_2_CCl_3_	Rh_2_(*S*-PTAD)_4_	16	2:1	67
5	2:1	CH_2_CCl_3_	Rh_2_(*S*-PTTL)_4_	16	2:1	67
6	2:1	CH_2_CCl_3_	Rh_2_(*R*-TPPTTL)_4_	29	20:1	–90
7^e^	1:1.5	CH_2_CCl_3_	Rh_2_(*S*-TPPTTL)_4_	77	20:1	95
8^e^	1:1.5	CH_2_CF_3_	Rh_2_(*S*-TPPTTL)_4_	20	20:1	91
9^e^	1:1.5	CH_3_	Rh_2_(*S*-TPPTTL)_4_	trace	n.d.	n.d.

a Reaction conditions: **1c** (0.2 mmol), **2a** (0.1 mmol), 1 mol % Rh_2_(L)_4_, 4 Å molecular sieves in CH_2_Cl_2_ (0.05 M), 39 °C, 3 h slow addition.

b Isolated yield.

c rr was determined by ^1^H NMR 5.80 ppm (s, 1H); 5.39 ppm (d, 1H).

d ee was determined by SFC analysis. A negative value indicates that the major enantiomer is opposite to the one drawn.

e **1c** (0.1 mmol), **2** (0.15 mmol), 0.5 mol % Rh_2_(L)_4_.

Having obtained the optimal conditions for desymmetrizing **1d**, the scope of the reaction was then studied (Table 4). In general, the reaction of aryldiazoacetates with the dibromo[2.2]paracyclophane **1d** proceeded in high yield (50–75% yield) and high levels of enantioselectivity (92–98% ee), as seen for the products **3da–3dq**. Only a single diastereomer of the product was formed. The reaction was not as effective when there were electron-donating para substituents on the phenyl ring, as seen with the Ph (**3db**) and *t*-Bu (**3dc**) derivatives, which were formed in 23% yield and 84% ee and 43% yield and 88% ee, respectively. The meta substituents had minimal effects on yield and enantioselectivity. This substitution pattern performed with moderate to good yields and excellent levels of asymmetric induction, as seen with **3dh**–**3dj** and **3dn**. The reaction could tolerate steric bulk around the carbene center imposed by an ortho substituent (**3dm**) because the yield and enantioselectivity remained high (70% yield, 95% ee). A naphthalene-derived diazo compound was not very effective because only a 28% yield and 82% ee of **3dk** was observed. Heterocycles, such as pyridine, were also compatible and resulted in the formation of **3dl** in 68% yield with 97% ee.

**Table 4:**
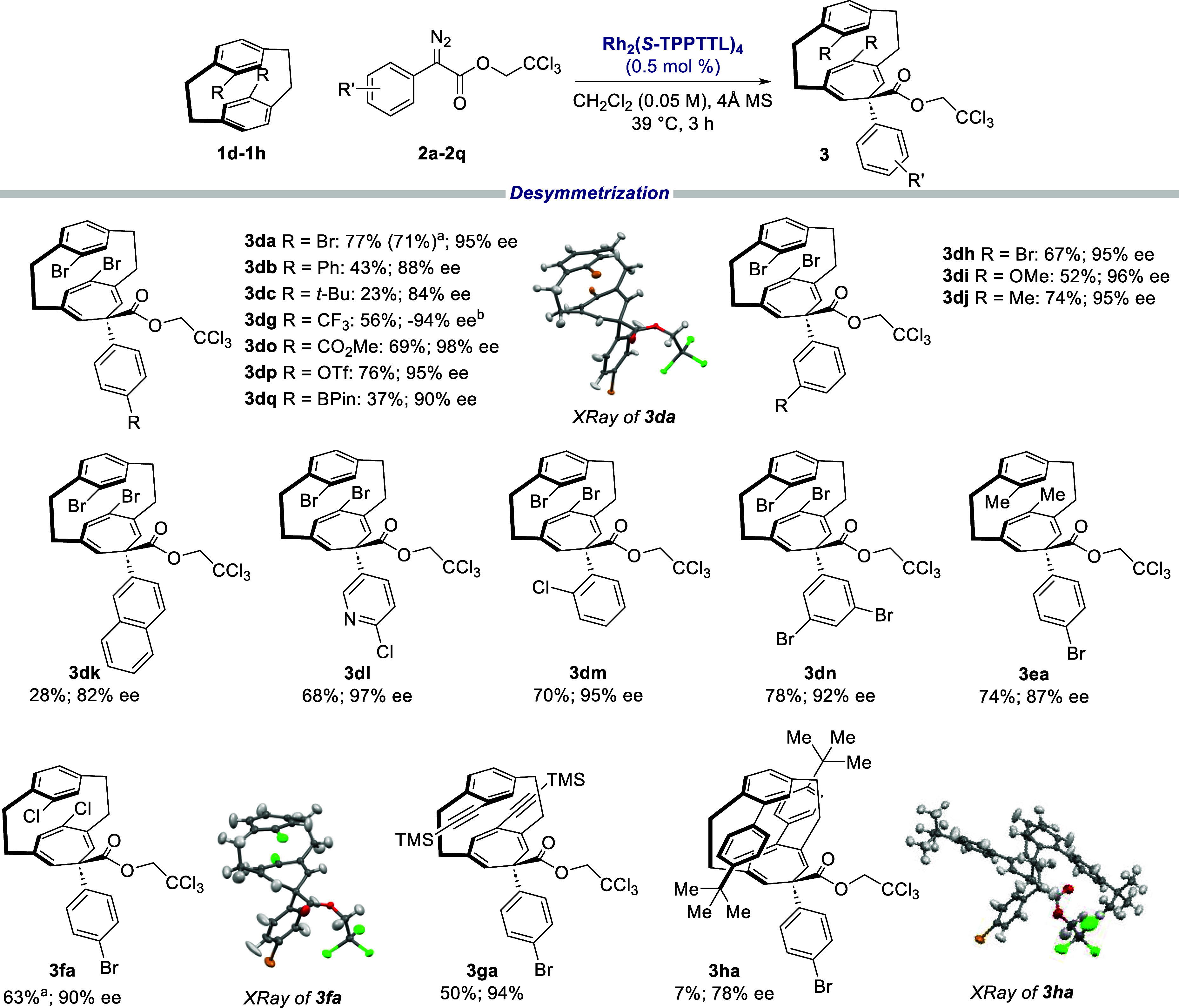
Scope of Desymmetrization of 4,16-Disubstituted [2.2]Paracyclophanes^c^

a Reaction scale of 1 g.

b Rh_2_(*R*-TPPTTL)_4_ was used as catalyst.

c Reaction conditions: **1** (0.2 mmol), **2** (0.3 mmol), Rh_2_(*S*-TPPTTL)_4_ (0.5 mol %) in CH_2_Cl_2_ (4 mL) with 4 Å molecular sieves (100 wt %) at 39 °C, 3 h slow addition. Yields refer to isolated yields. All the reactions proceeded in >30:1 dr in all cases,

Not only is the Büchner reaction compatible with various types of diazo compounds but it can also be extended to other disubstituted [2.2]paracyclophanes. Donating groups, such as Me (**3ea**), gave excellent results with 74% yield and 87% ee. The reaction was also compatible with slightly withdrawing groups where Cl (**3fa**) and ethynyl (**3ga**) substituents resulted in 63% yield and 90% ee and 50% yield and 94% ee, respectively. However, bigger substituents on the phenyl ring, such as *p-tert*-butylphenyl, gave a very low yield (7%) of **3ha**. This would indicate that the bowl-shaped catalyst, Rh_2_(*S*-TPPTTL)_4_, has limitations with regard to the size of the [2.2]paracyclophane, presumably because if it is too big, then it will not fit in the bowl. The absolute configurations of three products **3da**, **3fa**, and **3ha** were unambiguously determined by X-ray crystallography. The absolute configuration of the other products were tentatively assigned by analogy.

**Scheme 4:**
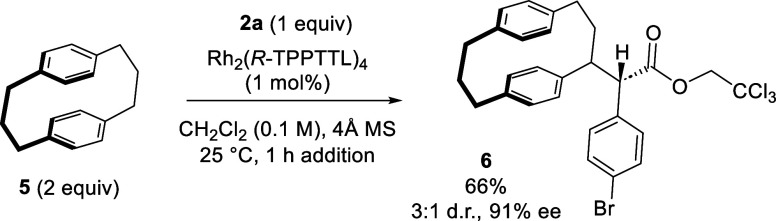
C–H Functionalization of [3.3]Paracyclophane **5**

The preference for aromatic functionalization over C(sp^3^)–H functionalization was considered to be due to the bent and strained nature of the benzene ring in [2.2]paracyclophanes. In order to test this hypothesis, a similar reaction was conducted on [3.3]paracyclophane **5**. Under the standard conditions, **5** resulted exclusively in C–H insertion at a benzylic position under the presence of Rh_2_(*R*-TPPTTL)_4_ to afford **6** with moderate diastereoselectivity (3:1 dr) and good enantioselectivity (91% ee) (Scheme 4). The preference for C–H insertion at the benzylic position in **5** can be attributed to a less activated aromatic ring, presumably due to lower strain energy.^27^

Having established an effective method for the monocyclopropanation of the paracyclophanes, we became interested in determining whether it would be possible to conduct a second cyclopropanation on the remaining phenyl ring of the initial product **3da**. In order to test this concept, we explored the Rh_2_(*S*-TPPTTL)_4_ reaction of **3da** (99% ee) with the standard aryldiazoacetate **2a** (Table 5). The reaction proceeded poorly, and only 29% of the double cyclopropanated product **7a** was formed (entry 1). This would suggest that this was a mismatched reaction. Hence, the reaction was repeated using Rh_2_(*R*-TPPTTL)_4_ as catalyst and, in this case, the *meso* double cyclopropanated product **7a** was formed in 96% yield (entry 2). The cyclopropanation of **3da** by **2a** could be achieved using the achiral catalyst Rh_2_(OBz)_4_ to afford **7a** in 63% yield (entry 3). Chiral products would be generated if the second cyclopropanation was conducted with a different aryldiazoacetate (entries 4 and 5). Although this would be feasible using the opposite enantiomer of Rh_2_(TPPTTL)_4_ to the one that did the first cyclopropanation, it was found that even an achiral catalyst could be used in the second cyclopropanation because the stereochemical outcome of the second cyclopropanation is strongly controlled by the configuration of the monocyclopropanated product **3da**. Hence, Rh_2_(OBz)_4_-catalyzed cyclopropanation of **3da** (99% ee) with **2f** (4-CF_3_) or **2n** (3,5-diBr) generated the double cyclopropanated product **7b** or **7c** as single diastereomers in 98–99% ee.

**Table 5:**
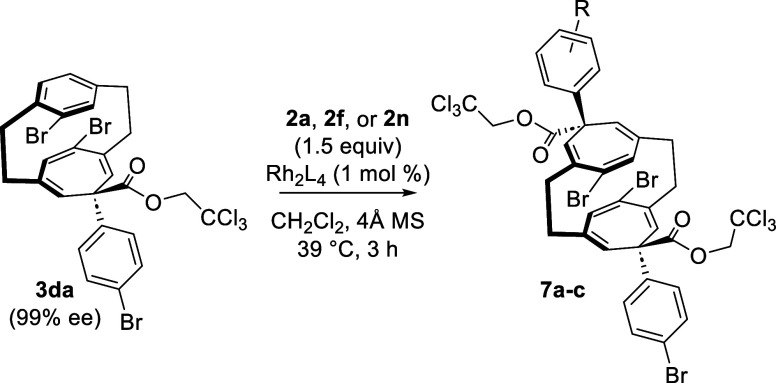
Second Cyclopropanation of [2.2]Paracyclophane **3da**

entry	R	catalyst	product	yield, %^a^	ee, %
1	4-Br	Rh_2_(*S*-TPPTTL)_4_	**7a**	29	*meso*
2	4-Br	Rh_2_(*R*-TPPTTL)_4_	**7a**	96	*meso*
3	4-Br	Rh_2_(OBz)_4_	**7a**	63	*meso*
4	3,5-diBr	Rh_2_(OBz)_4_	**7b**	67	99
5	4-CF_3_	Rh_2_(OBz)_4_	**7c**	75	98

a Isolated yields.

The direct formation of the *meso* double cyclopropanated product **7a** could, in principle, be achieved directly from the starting paracyclophane **1d** using an achiral catalyst (Table 6). However, the double cyclopropanation of **1d** with **2a** using Rh_2_(OBz)_4_, the most regioselective achiral catalyst from the studies in Table 1, failed to cleanly give the desired product (entry 1). A much more effective way to achieve direct double cyclopropanation was to conduct the reaction with the racemic catalyst, Rh_2_(*R/S*-TPPTTL)_4_, and under these conditions, **7a** was formed in 85% yield (entry 2). Presumably, Rh_2_(*R/S*-TPPTTL)_4_ is more regioselective than Rh_2_(OBz)_4_ in the cyclopropanation of the C2–C3 double bonds versus the C1–C2 double bonds with **1d**, and with a racemic mixture, Rh_2_(*R/S*-TPPTTL)_4_, a matched catalyst is present to undergo the second cyclopropanation reactions. Similar reactions were possible with other aryldiazoacetates, as illustrated with **2g** (*p*-CF_3_) and **2n** (3,5-diBr), which generated the double cyclopropanated products **7d** and **7e** in 47% and 64% yields, respectively (entries 3 and 4). It is worth noting that yields obtained from double cyclopropanation tend to be higher than yields obtained from sequential cyclopropanation because of incomplete consumption of **1d** when the two cyclopropanations are conducted in separate reactions.

**Table 6:**
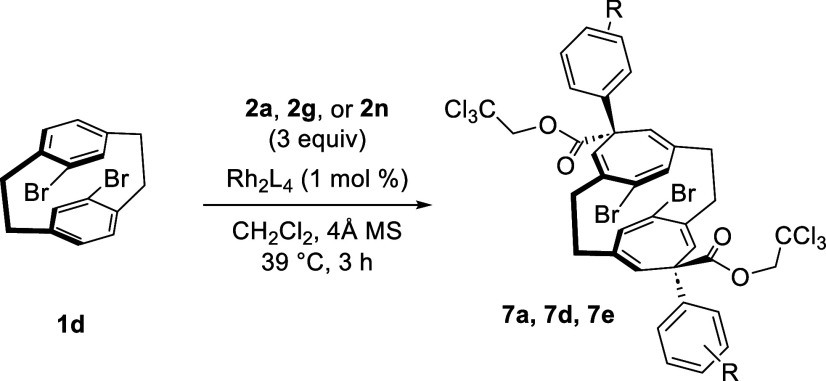
Double Cyclopropanation of [2.2]Paracyclophane **1d**

entry	R	catalyst	product	yield, %^a^
1	4-Br	Rh_2_(OBz)_4_	**7a**	39
2	4-Br	Rh_2_(*R*/*S*-TPPTTL)_4_	**7a**	85
3	3,5-diBr	Rh_2_(*R*/*S*-TPPTTL)_4_	**7d**	47
4	4-CF_3_	Rh_2_(*R*/*S*-TPPTTL)_4_	**7e**	64

a Isolated yields.

**Scheme 5:**
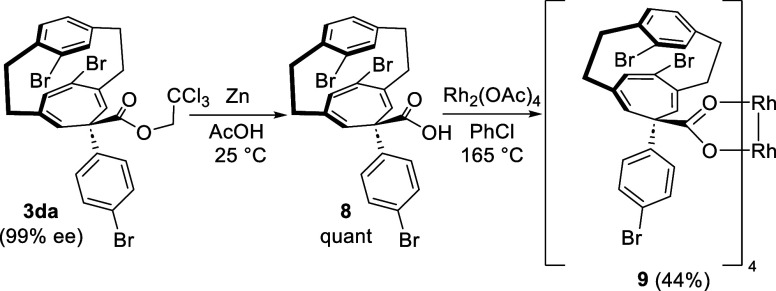
Synthesis of Novel Chiral Dirhodium Catalyst on the Basis of [2.2]Paracyclophane Scaffold

One of the most compelling examples of the use of chiral paracyclophanes is as ligands for asymmetric catalysis. Hence, we were intrigued by whether the paracyclophanes we had prepared could be used to generate effective chiral dirhodium tetracarboxylate catalysts. The trichloroethyl ester in **3da** could be readily converted to acid **8** upon treatment with zinc, and we were pleased to see that the reaction of **8** with dirhodium tetraacetate (Rh_2_(OAc)_4_) under ligand exchange conditions effectively generated the desired catalyst **9** in 44% yield (Scheme 5).

**Figure 1:**
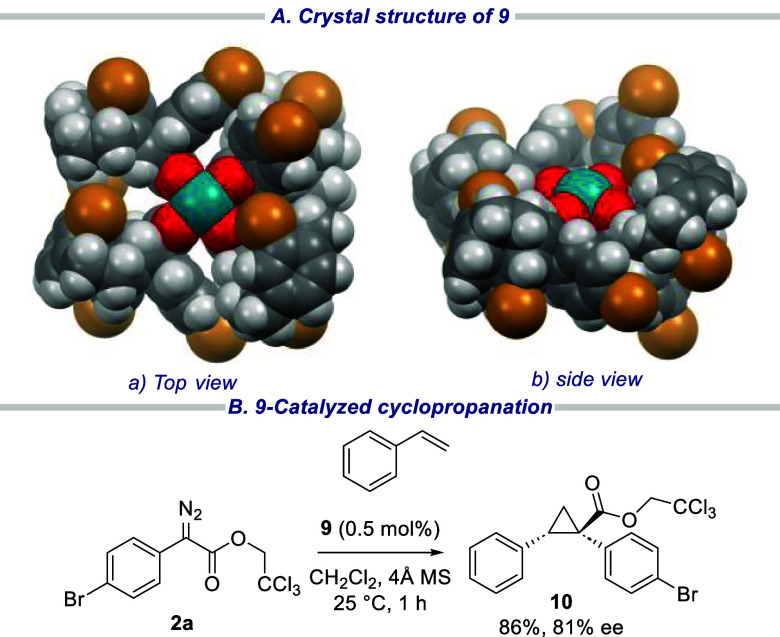
Crystal structure of **9** and its utility in asymmetric cyclopropanation. (A) X-ray structure of **9**. (B) Cyclpropoanation of styrene and **2a** was catalyzed by **9**.

One of the reasons why dirhodium tetracarboxylates can be such effective chiral catalysts is because the self-assembly of the four ligands around the dirhodium catalyst core can result in complexes of higher symmetry than the ligands themselves.^19^ Therefore, we were intrigued to explore whether the new catalysts would adopt a highly symmetrical structure. As can be seen from the X-ray structure shown in Figure 1, catalyst **9** adopts a C_2_-symmetric structure, which means the stereochemical environment on both faces of the catalyst are the same. A quick study was carried out to determine if catalyst **9** has potential as a chiral catalyst, and we were pleased to observe that it did result in the asymmetric cyclopropanation of styrene to form **10** in high yield (86%) with reasonably high levels of asymmetric induction (81% ee). Further studies will be conducted to determine if this class of chiral ligands could lead to catalysts with unusual features in terms of site-selective or enantioselective carbene reactions that would make them useful additions to the toolbox of chiral catalysts that have now been developed for the rhodium-carbene chemistry.^19^

**Figure 2:**
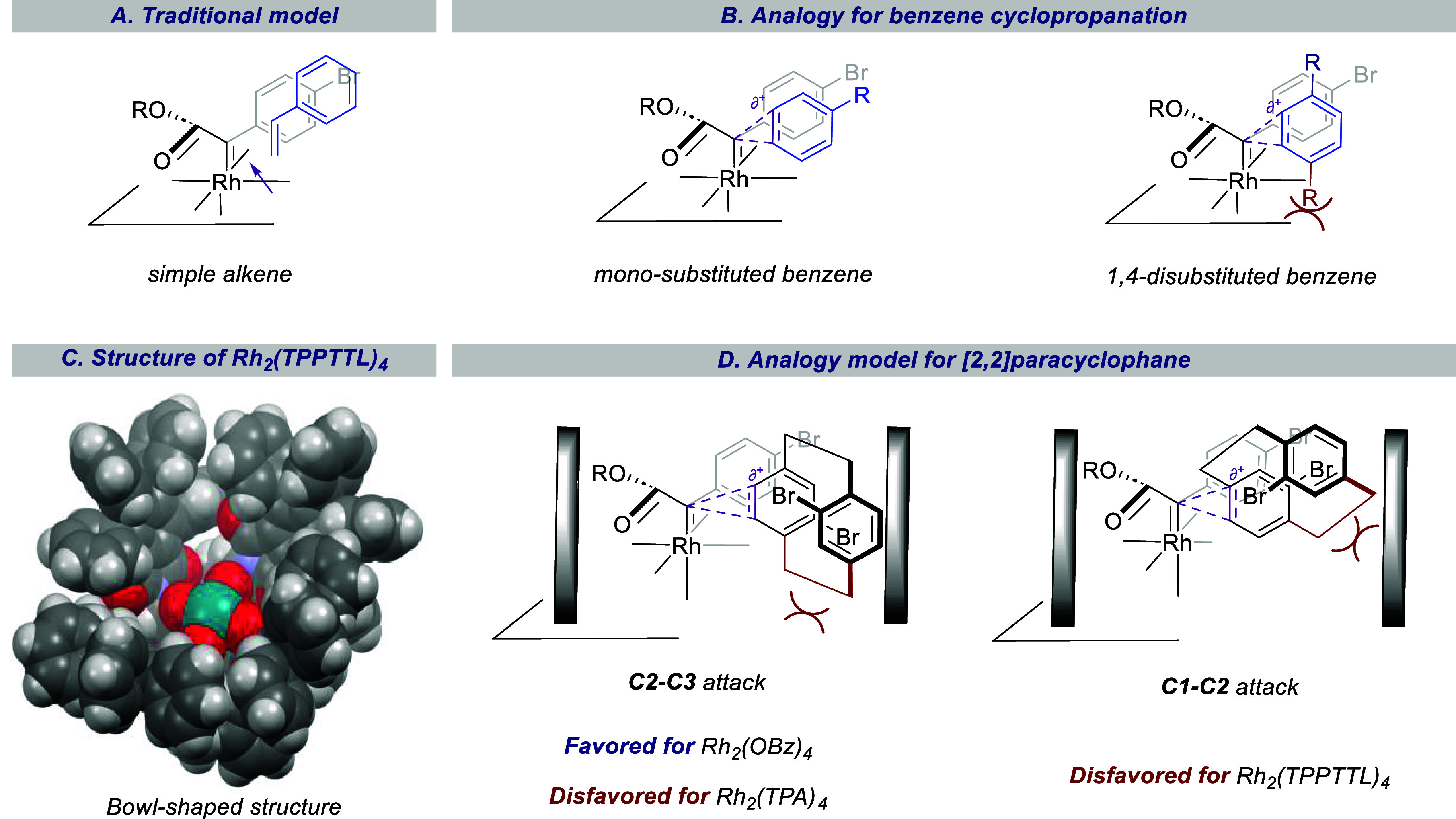
Model to rationalize the regio- and diastereoselectivity of the cyclopropanation by donor/acceptor carbenes of [2.2]paracyclophane. (A) The traditional model for concerted asynchronous cyclopropanation of styrene illustrates the end-on approach of the styrene with the phenyl ring aligned over the donor group, which results in a highly diastereoselective reaction. (B) Illustration how monosubstituted aryl rings can be cyclopropanated, whereas 1,4-disubstited benzene rings are sterically protected. (C) X-ray structure of Rh_2_(TPPTTL)_4_ illustrating the bowl-shaped structure. (D) Steric influence of the catalysts versus the wall of bowl-shaped catalysts on C2–C3-cyclopropanation versus C1–C2 cyclopropanation.

The successful cyclopropanation of aryldiazoacetates with the paracyclophanes is intriguing because the [2.2]paracyclophanes have structural features that could have interfered with the cyclopropanation. In order to rationalize the results, one needs to consider the standard reactivity profile of donor/acceptor carbenes.^28a−28g^ When these carbenes are rhodium-bound, they behave as sterically demanding intermediates capable of exceptional site selectivity. The computationally supported model that has been used for the regular cyclopropanation is shown in Figure 2A.^28a^ The rhodium carbene complexes are considered to be coordinatively saturated, and all the reactions occur because of how substrates approach the carbene without any prior coordination to the rhodium. The aryl group of the carbene (the donor group) lies in the plane of the rhodium carbene bond, whereas the ester group (the acceptor group) aligns orthogonally.^28,29^ The alkene approaches the carbene end-on with the substituent oriented over the donor group, which leads to high diastereoselectivity. The cyclopropanation is considered to be a concerted, asynchronous process. Hence, alkenes that are not sterically constrained on one side are generally preferred: monosubstituted, 1,1-disubstituted alkenes are excellent substrates, *cis*-1,2-disubstiuted alkenes are less reactive, and *trans*-1,2-disubstituted alkenes or more highly substituted alkenes tend to undergo preferential allylic C–H functionalization.^30,31^

Similar steric influences are seen with benzene derivatives (Figure 2B). Monosubstituted benzene derivatives are capable of undergoing cyclopropanation with donor/acceptor carbenes^22,23a^ but when they are 1,4-disubstituted they are sterically protected because there will be steric interference with an adjacent substituent pointing toward the catalyst surface during the asynchronous cyclopropanation (Figure 2B).

Another factor that must be considered is the steric influence of the catalysts. In general, the surface of the rhodium carboxylate is considered as a steric wall, and substrates will tend to approach away from the surface, although there are a few exceptions.^32−34^ We have generated a wide variety of catalysts with different steric demand, and they greatly influence the outcome of this chemistry.^19^ The two most extensively used catalysts in the current study, the achiral Rh_2_(OBz)_4_ and the chiral Rh_2_(*S*-TPPTTL)_4_ have very different structural profiles. Rh_2_(OBz)_4_ has a relatively flat surface, and substrates approaching the carbene would simply need to avoid close proximity to the catalyst surface. In contrast, Rh_2_(*S*-TPPTTL)_4_ adopts a C_4_-symmetric bowl shape, as illustrated in the X-ray structure shown in Figure 2C.^25,35^ Hence, substrates approaching the carbene in reactions catalyzed by Rh_2_(*S*-TPPTTL)_4_ would need to avoid the surface of the catalyst and the wall of the bowl.

Cyclopropanation of [2.2]paracyclophane offers an interesting dilemma. A standard 1,4-disubstituted benzene ring is sterically protected, as was seen in the case of the [3.3] paracyclophane. However, because of the strain associated with the [2.2]paracyclophane, the benzene ring is actually bent and more reactive, thereby making the system more susceptible for cyclopropanation despite the steric difficulties (Figure 2D). If an attack occurs at the C2–C3 double bond with a similar type of orientation to that proposed for a cyclopropanation of a monosubstituted alkene, then the alkyl chain points inward toward the catalyst wall. This steric clash with the catalyst wall can be circumvented somewhat by reaction at the C1–C2 double bond of the paracyclophane, and this is reasonably favorable, even though it involves the cyclopropanation of a more sterically demanding tertiary double bond. When an attack is occurring at the C1–C2 double bond, it does place the paracyclophane in a position where it could have interference with the sidewall of a bowl-shaped catalyst (Figure 2D). Thus, the reaction with bulky catalysts, like dirhodium triphenylacetate, will preferentially form the C1–C2 products in order to limit the clashes between the alkyl component of the paracyclophane and the catalyst wall. If the catalysts are not bulky, such as Rh_2_(OBz)_4_, then the reaction preferentially occurs at C2–C3 because the steric interference with the wall is not so pronounced, and the inherent preference for reaction with a disubstituted double bond versus a trisubstituted double bond can occur. This can be further reinforced with Rh_2_(*R/S*-TPPTTL)_4_ because this catalyst is not very sterically demanding at the position of the carbene, but the side walls of this catalysts would be expected to disfavor C1–C2 cyclopropanation.^25^

In conclusion, this work illustrates the subtle regiocontrol in the rhodium-catalyzed reactions of donor/acceptor carbenes. The reaction with [2.2]paracyclophanes results in cyclopropanation of the benzene ring, whereas the reaction with [3.3]paracyclophane results in benzylic C–H functionalization. Two possible cyclopropanes can be generated from the reaction of [2.2]paracyclophanes, and the ratio of products can be predictably controlled by using the appropriate catalyst. The reaction with C_2v_-symmetric [2.2]paracyclophanes results in desymmetrization with high levels of asymmetric induction. These studies demonstrate an effective new entry into a collection of unusual paracyclophane derivatives, including ready access to enantiomerically enriched materials.

## Data Availability

The data underlying this study are available in the published article and its online Supporting Information.

## Supporting Information Available

Complete experimental procedures and compound characterization (PDF)
CIF file of **3aa** (CIF)
CIF file of **3da** (CIF)
CIF file of **3fa** (CIF)
CIF file of **3ha** (CIF)
CIF file of **8** (CIF)
CIF file of **9** (CIF)

## Supplementary Material

cs4c01292_si_001.pdf

cs4c01292_si_002.cif

cs4c01292_si_003.cif

cs4c01292_si_004.cif

cs4c01292_si_005.cif

cs4c01292_si_006.cif

cs4c01292_si_007.cif

## Accession Codes

The following crystal structure has been deposited in the Cambridge Crystallographic Data Centre: Compound **3aa** (CCDC 2336106), compound **3da** (CCDC 2335504), compound **3fa** (CCDC 2335506), compound **3ha** (CCDC 2335554), compound **8** (CCDC 2336626), and compound **9** (CCDC 2335496). These data can be obtained free of charge via www.ccdc.cam.ac.uk/data_request/cif, or by emailing data_request@ccdc.cam.ac.uk, or by contacting The Cambridge Crystallographic Data Centre, 12 Union Road, Cambridge CB2 1EZ, UK; fax: + 44 902 1223 336033.
