# Supplementary material for: Rhodium(II)-Catalyzed Asymmetric Cyclopropanation and Desymmetrization of [2.2]Paracyclophanes
Source: ACS Catal. 2024 Apr 11;14(9):6423–31. doi: 10.1021/acscatal.4c01292 (PMC11075029; doi:10.1021/acscatal.4c01292)
Supplement: Supplementary file 1 — cs4c01292_si_001.pdf [file cs4c01292_si_001.pdf]

Supporting Information

Rhodium(II)-Catalyzed Asymmetric Cyclopropanation and Desymmetrization of [2.2]Paracyclophanes

*Duc Ly, John Bacsa and Huw M. L. Davies**

Department of Chemistry, Emory University, 1515 Dickey Drive, Atlanta, Georgia 30322, United States

Corresponding author email: hmdavie@emory.edu

Complete experimental procedures, materials, and compound characterizations

Table of Contents

1. General Information S2
2. Catalyst structure S4
3. Preparation of starting materials S5
4. Optimization S8
   4.1. Optimization for cyclopropanation of [2.2]paracyclophane S8
   4.2. Kinetic resolution cyclopropanation S10
   4.3. Optimization for desymmetrization cyclopropanation of [2.2]paracyclophane S15
5. C-H insertion of [3.3]paracyclophane S16
6. Rhodium catalyzed cyclopropanation of [2.2]paracyclophane and characterization of productS19
7. Rhodium catalyzed enantioselective cyclopropanation of 4,16-substituted [2.2]paracyclophane and characterization of product S26
8. Second cyclopropanation of S54
9. Double cyclopropanation S60
10. [2.2]paracyclophane-based dirhodium catalyst synthesis S63
11. Dirhodium activity S66
12. Copies of NMR spectra S67
13. X-Ray Crystallographic S123
14. Reference S130

1. General Information

All reagents and solvents were used as purchased from commercial sources (Sigma) for substrate synthesis unless otherwise noted. Dichloromethane used in C–H insertion reactions was prepared from solvent purification system. 4 Å molecular sieves was activated at 220 °C for 4 hours under vacuum and stored in an oven over 100 °C. All column chromatography was performed on silica gel (SiliaFlash® P60, 40-63 μm). Thin layer chromatographic (TLC) analysis was performed with aluminum-sheet silica gel plates. 1 H, 13C and 19F NMR spectra were recorded at 800MHz or 600 MHz on Bruker-800 spectrometer, Bruker-600 spectrometer or Varian IVONA-600 spectrometer (13C at 151 MHz), 500 MHz on Varian INOVA-500 spectrometer, or 400 MHz (13C at 101 MHz, 19F at 376 MHz) on Bruker-400 spectrometer and all were reported in parts per million (ppm). Unless otherwise noted, ^{1}H, ^{13}C and ^{19}F NMR spectra were performed in solutions of deuterated chloroform (CDCl3) with the residue chloroform set as an internal standard (7.26 ppm for 1 H, and 77.16 ppm for 13C). Abbreviations for signal multiplicity are as follows: br = broad, s = singlet, d = doublet, t = triplet, q = quartet, m = multiplet, dd = doublet of doublet, tt = triplet of triplet, qt = quartet of triplet, dtd = doublet of triplet of doublet. Coupling constants (J values) were calculated directly from the spectra. IR spectra were collected on a Nicolet iS10 FT-IR spectrometer. Mass spectra were taken on a Thermo Finnigan LTQ-FTMS spectrometer with APCI, ESI or NSI. Melting points (m.p.) were measured in open

capillary tubes with a Mel-Temp Electrothermal melting points apparatus and are uncorrected. The enantiomeric excess (ee) was determined by High Performance Liquid Chromatography analysis was performed on either Varian Prostar model 410 HPLC, Agilent 1100 Technologies HPLC, Agilent Technologies 1290 Infinity UHPLC instruments, or by Super Critical Fluid Chromatography using Water Acquity UPC^2 SFC system and the data outlined below varies in presentation based on the software used for each system. HPLC/SFC traces are reported based on the racemic retention times. The HPLC instruments used isopropanol/hexane gradient and commercial ChiralPak/ChiralCel columns from Daicel Chemical Industries, notably ChiralPak AD-H (5 µm particle size, 4.6 mm vs. 250 mm), ChiralCel OZ-H (5 µm particle size, 4.6 mm vs. 250 mm), and ChiralCel OD-H (5 µm particle size, 4.6 mm vs. 250 mm), ChiralCel AS-H (5 µm particle size, 4.6 mm vs. 250 mm), ChiralCel OJ-H (5 µm particle size, 4.6 mm vs. 250 mm), and Regis (S,S) Whelk-O1 5/100 Kromasil. The SFC system utilized supercritical fluid CO_2 with cosolvents of either HPLC-grade methanol, or acetonitrile, or ethanol, or isopropanol, or 1:1 MeOH:IPA with 0.2% formic acid, or 1:1 Ethanol:IPA with 0.2% formic acid, or 1:1 Ethanol:ACN with 0.2% formic acid, or 1:1:1 Ethanol:IPA:ACN with 20 mM ammonium formate with SFC columns: Trefoil AMY1 Column (2.5 µm, 3.0 mm X 150 mm), Trefoil CEL1 Column (2.5 µm, 3.0 mm X 150 mm), Trefoil CEL2 Column (2.5 µm, 3.0 mm X 150 mm), Regis (S,S) Whelk-O 1 Kromasil (3.5 µm, 3.0 mm X 150 mm), ChiralPak AD-3 (3.0 µm, 3.0 mm X 150 mm SFC), ChiralCel OZ-3 (3.0 µm, 3.0 mm X 150 mm), ChiralCel OD-3 (3.0 µm, 3.0 mm X 150 mm SFC), ChiralCel OX-3 (3.0 µm, 3.0 mm X 150 mm SFC); ChiralCel OJ-3 (3.0 µm, 3.0 mm X 150 mm SFC); ChiralPak AS-3 (3.0 µm, 3.0 mm X 150 mm SFC). In general, chiral HPLC or SFC conditions were determined by obtaining separation of the racemic products using $Rh_2(OBz)_4$ as a catalyst.

2. Catalyst structure

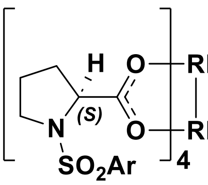

Ar = *p*-(C$_{12}$H$_{25}$)C$_6$H$_4$

Rh$_2$(*S*-DOSP)$_4$

Generation: Prolinato
Name: Rh$_2$(*S*-DOSP)$_4$[1]

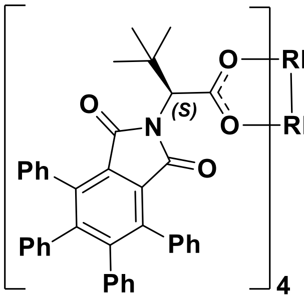

Rh$_2$(*S*-TPPTTL)$_4$

Generation: Phthalimido
Name: Rh$_2$(*S*-TPPTL)$_4$[2]

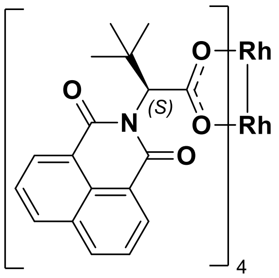

Rh$_2$(*S*-NTTL)$_4$

Generation: Napthalimido
Name: Rh$_2$(*S*-NTTL)$_4$[3]

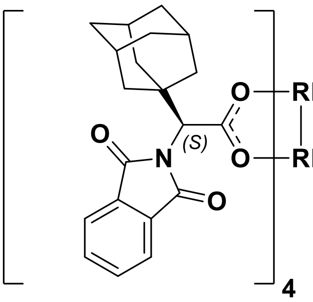

Rh$_2$(*S*-PTAD)$_4$

Generation: Phthalimido
Name: Rh$_2$(*S*-PTAD)$_4$[4]

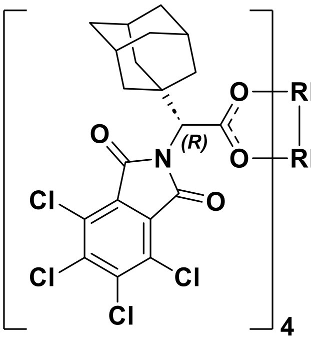

Rh$_2$(*R*-TCPTAD)$_4$

Generation: Phthalimido
Name: Rh$_2$(*R*-TCPTAD)$_4$[4]

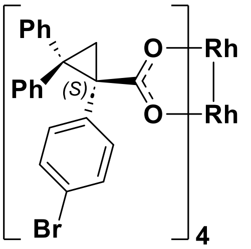

Rh$_2$(*S*-*p*-BrTPCP)$_4$

Generation: TPCP
Name: Rh$_2$(*S*-*p*-BrTPCP)$_4$[5]

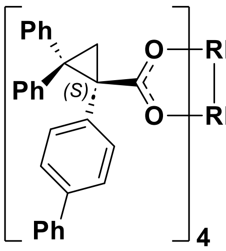

Rh$_2$(*S*-*p*-PhTPCP)$_4$

Generation: TPCP
Name: Rh$_2$(*S*-*p*-PhTPCP)$_4$[5]

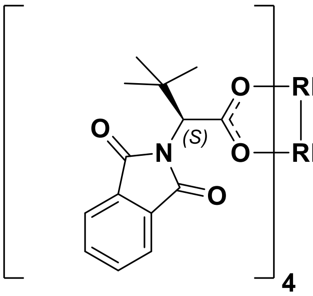

Rh$_2$(*S*-PTTL)$_4$

Generation: Phthalimido
Name: Rh$_2$(*S*-PTTL)$_4$[4]

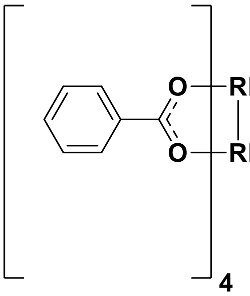

Rh$_2$(OBz)$_4$

Generation: Achiral catalyst
Name: Rh$_2$(OBz)$_4$[6]

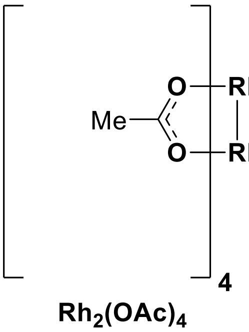

Rh$_2$(OAc)$_4$

Generation: Achiral catalyst
Name: Rh$_2$(OAc)$_4$

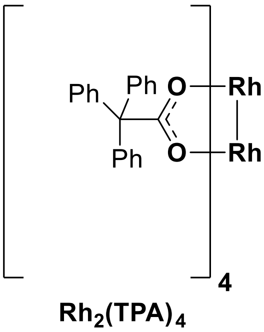

Rh$_2$(TPA)$_4$

Generation: Achiral catalyst
Name: Rh$_2$(TPA)$_4$

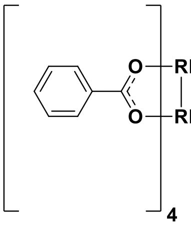

$Rh_2(OBz)_4$

Dirhodium tetrabenzoate ($Rh_2(OBz)_4$)

This compound was synthesized according to reported procedures with slightly changed conditions.[6] To a flame-dried 50 ml round bottom flask equipped with a magnetic stir bar was charged with benzoic acid (391 mg, 3.2 mmol, 8.0 equiv) and $Rh_2(OAc)_4$ (177 mg, 0.4 mmol, 1.0 equiv). After that, 20 ml of anhydrous chlorobenzene was added to the above reaction mixture. The flask was fitted with a Soxhlet extractor with a thimble filled with a layer of K_2CO_3 and sand. A water condenser was connected onto the top of the Soxhlet extractor, and the reaction mixture was refluxed on heating block at 160 °C for 24 hours. After the reaction was completed as indicated that all $Rh_2(OAc)_4$ was consumed by TLC (5% MeOH in DCM), chlorobenzene solvent was removed under vacuum resulting to a crude green solid. The crude mixture was re-dissolved into dichloromethane and washed with $NaHCO_3$ to remove excess amounts of benzoic acid. The organic layer was then washed with brine and dried over Na_2SO_4. The solvent was then removed under vacuum to give the desired product $Rh_2(OBz)_4$ as a green solid (268.3 mg, 97%). *Note: depending on the source of $Rh_2(OAc)_4$, the product might need to be purified further by column chromatography. If so, the green solid was then dried load on silica and purified by flash chromatography (0-5% MeOH in DCM).*

R_f (5% MeOH in DCM) = 0.8

^{1}H NMR (400 MHz, Acetone-*d_6*) δ 7.91 – 7.84 (m, 8H), 7.43 – 7.37 (m, 4H), 7.33 – 7.26 (m, 8H).

^{13}C NMR (101 MHz, Acetone-*d_6*) δ 185.2, 132.7, 129.3, 128.7 (*missing 1 carbon*)

HRMS (-p ESI) calcd. for $[C_{28}H_{20}O_8{}^{35}Cl^{103}Rh_2]$ ($[M+Cl]^-$) 724.8962 found 724.8961.

3. Preparation of starting materials

A. Diazo compound

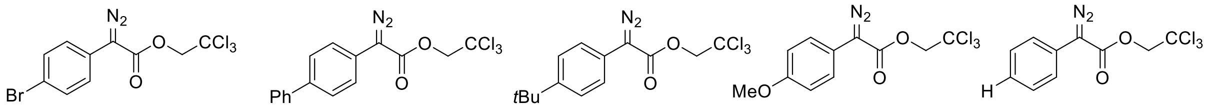

2a 2b 2c 2d 2e

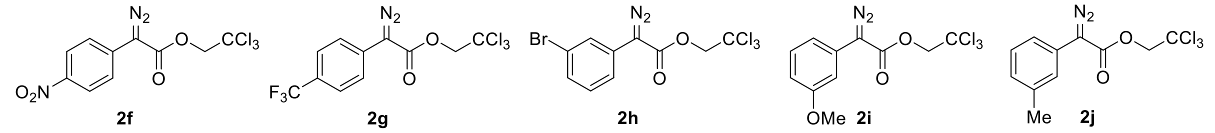

2f 2g 2h 2i 2j

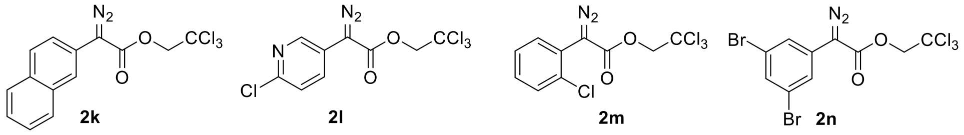

2k 2l 2m 2n

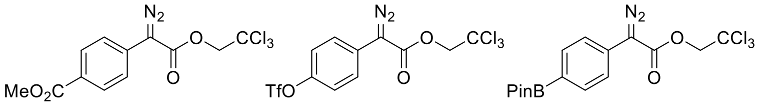

2o 2p 2q

- Diazo compounds 2a, 2c, 2g, 2l were prepared using the procedure reported in the literature.[7]
- Diazo compounds 2k, 2f, 2e, 2d, 2h, 2i, 2j, 2o, 2q were prepared using the procedure reported in the literature.[8]
- Diazo compounds 2b, 2n, and 2m were prepared using the procedure reported in the literature.[9]

B. Paracyclophane

4,16-disubstituted [2,2]PCP

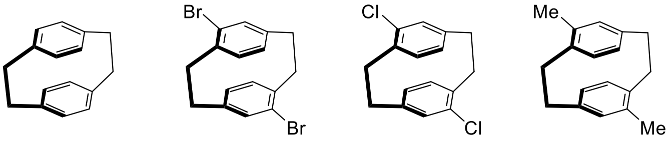

1a 1d 1f 1e

monosubstituted [2,2]PCP

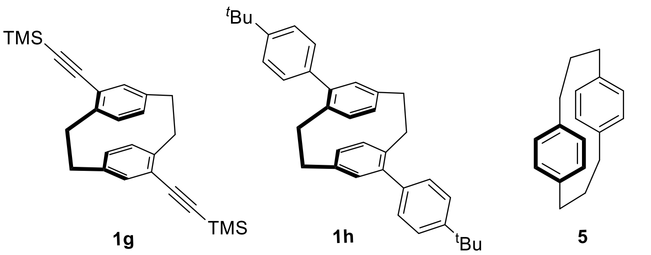

1g 1h 5

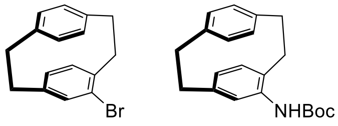

1b 1c

Unsuccessful substrate

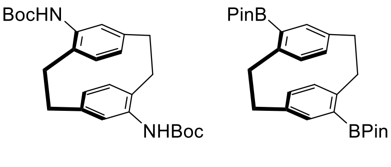

SI-1 SI-2

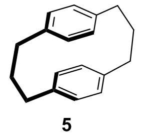

5

The paracyclophanes used for this study are listed below, in which **1a** and **1c** were purchased from Ambeed

- **1d** are prepared according to reported literature.[10]
- **1e** are prepared according to reported literature.[11]
- **1g** are prepared according to reported literature.[12],
- **5** are prepared according to reported literature.[13]
- **SI-1, 1c** are prepared according to reported literature.[14]
- **SI-2** are prepared according to reported literature.[15]
- **1g** are prepared according to reported literature.[12]
- **5** was prepared according to reported literature.[16]

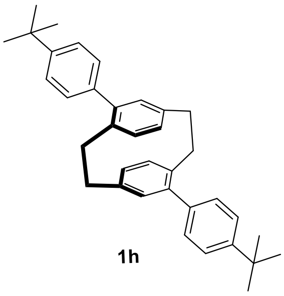

1h

12,43-bis(4-(tert-butyl)phenyl)-1,4(1,4)-dibenzenacyclohexaphane (1h)

To a 16 mL vial was charged with 12,43-dibromo-1,4(1,4)-dibenzenacyclohexaphane (732 mg, 2.00 mmol, 1 equiv,), (4-(tert-butyl)phenyl)boronic acid (1.42 g, 8.00 mmol, 4 equiv), Na_2CO_3 (848 mg, 8.00 mmol, 4 equiv) in the mixed solvent DMSO (12.1 mL) and Water (1.21 mL) under a nitrogen atmosphere, and then the mixture was degassed for 15 mins, then $Pd(PPh_3)_4$ (231 mg, 200 µmol, 0.1 equiv) was added. The reaction mixture is stirred at 90 °C for 254 hours. Then after cooling to room temperature, the resulting mixture was diluted with CH_2Cl_2 (100 mL) and water (50 mL). Afterwards, the resulting mixture was extracted three times CH_2Cl_2 (3×50 mL). The organic layer was washed with brine, dried over anhydrous Na_2SO_4, and solvent was removed in vacuo. Purification of the residue by flash column chromatography (0-10% DCM in hexane) to give a desired product as a white solid (281.0 mg, 30%)

R_f (6H/1DCM) = 0.33

^{1}H NMR (400 MHz, $CDCl_3$) δ 7.50 (d, *J* = 8.4 Hz, 4H), 7.46 (d, *J* = 8.5 Hz, 4H), 6.77 – 6.48 (m, 6H), 3.47 (ddd, *J* = 13.8, 9.1, 5.1 Hz, 2H), 3.02 (ddd, *J* = 13.8, 9.0, 5.5 Hz, 2H), 2.92 – 2.68 (m, 4H), 1.41 (s, 18H).

^{13}C NMR (101 MHz, $CDCl_3$) δ 149.6, 142.0, 139.9, 138.5, 136.8, 134.8, 132.3, 129.4, 129.1, 125.4, 34.8, 34.6, 33.8, 31.5.

HRMS (+p APCI) calcd. for [$C_{36}H_{41}$] ([$M+H]^+$) 473.3203 found 473.3200.

4. Optimization

4.1. Optimization for cyclopropanation of [2.2]paracyclophane (1a)

A. Catalyst screening

Table S1. Catalyst screening for [2.2]cyclopropanation with 1a

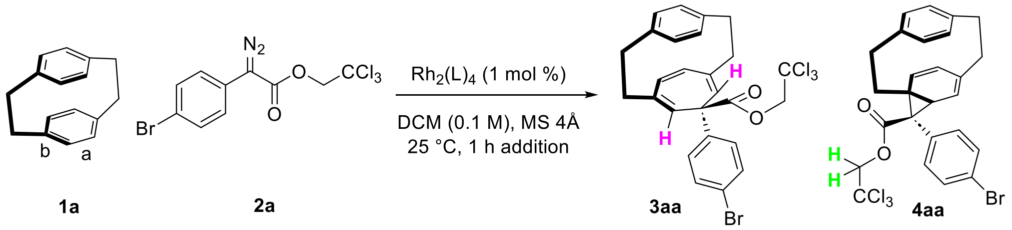

**Entry**[a]	**1a**	**2a**	**Catalyst (1 mol%)**	**Yield (%)**[b]	**r.r. (3aa:4aa)**[c]
**01**	1.0	1.2	$Rh_2(OAc)_4$	44	3.7:1
**02**	1.0	1.2	$Rh_2(OBz)_4$	54	2.4:1
**03**	1.0	1.2	$Rh_2(TFA)_4$	28	1.3:1
**04**	1.0	1.2	$Rh_2(OPiv)_4$	42	1:2
**05**	1.0	1.2	$Rh_2(TPA)_4$	54	1:7
**06**	1.0	1.2	$Rh_2(esp)_2$	48	1:3
**07**	1.0	1.2	$Rh_2(S\text{-}TPPTTL)_4$	48	5:1

[a]Reaction conditions: 1a (0.1 mmol), 2a, Rh_2L_4 (1.0 mol%), DCM (0.1 M), MS 4Å 1-hour slow addition. [b]Yields are NMR yield, using 1,2-dichloroethane as internal standard. [c]r.r. was determined by crude NMR using signal at 5.1 ppm (1H, d), and 5.8 ppm (2H, s). (Highlighted hydrogens are for r.r. determination).

Note: *Although $Rh_2(S\text{-}TPPTTL)_4$ gave decent regio-selectivity and yield, the desired product **3aa** is meso compound. Therefore, achiral dirhodium catalyst was chosen for further optimization.*

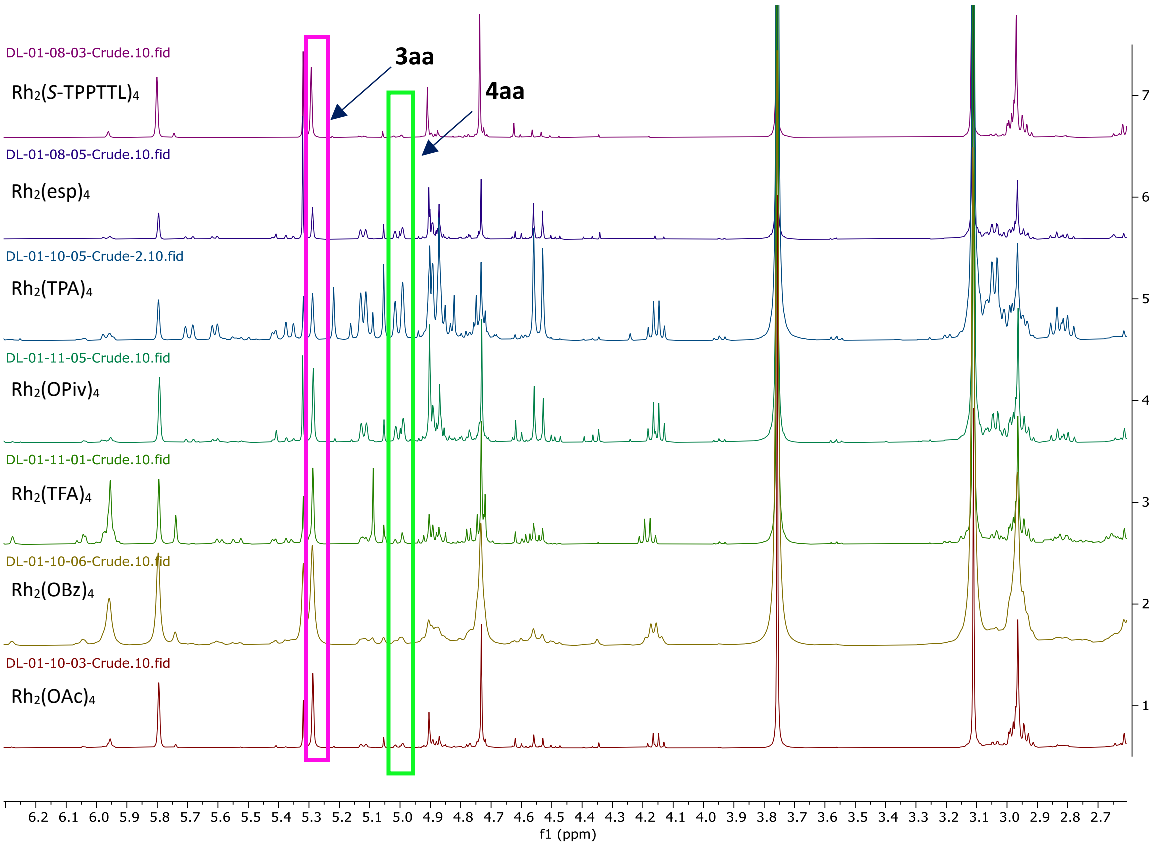

Figure S1. Catalyst screen for reaction with 2a (Crude NMR for r.r. determination, pinks for **3aa** and green for **4aa**)

B. Temperature screening

Table S2. Temperature screening

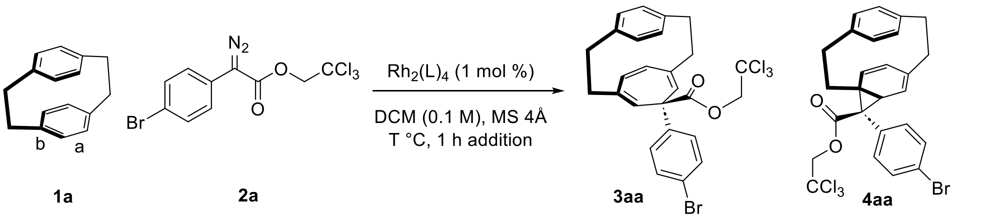

Entry[a]	1a	2a	Catalyst (1 mol%)	T °C	Yield (%)[b]	r.r. (3aa:4aa)[c]
**01**	3.0	1.0	$Rh_2(OAc)_4$	25	22	3.3:1
**02**	3.0	1.0	$Rh_2(OAc)_4$	39	54	6:1
**03**	3.0	1.0	$Rh_2(OBz)_4$	25	50	3.5:1
**04**	3.0	1.0	$Rh_2(OBz)_4$	39	74	8:1

[a]Reaction conditions: 2a (0.1 mmol), 1a, Rh_2L_4 (1.0 mol%), DCM (0.1 M), MS 4Å 3-hour slow addition. [b]Yields are NMR yield, using 1,2-dichloroethane as internal standard. [c]r.r. was determined by crude NMR using signal at 5.1 ppm (1H, d), and 5.8 ppm (2H, s). (Highlighted hydrogens are for r.r. determination

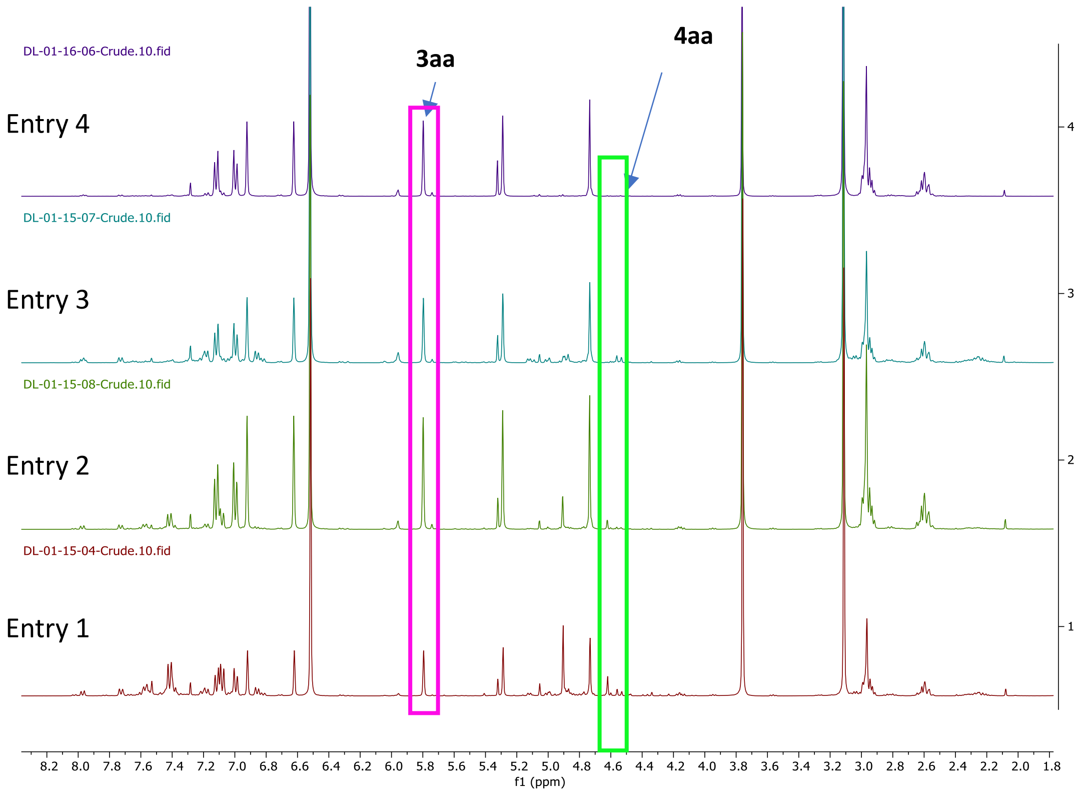

Figure S2. Temperature screen for reaction with **2a** (Crude NMR for r.r. determination, pinks for **3aa** and green for **4aa**)

4.2. Kinetic resolution cyclopropanation

4.2.1. Mono-bromo-[2.2]paracyclophane 1b

Table S3. Catalyst screening for cyclopropanation of 1b

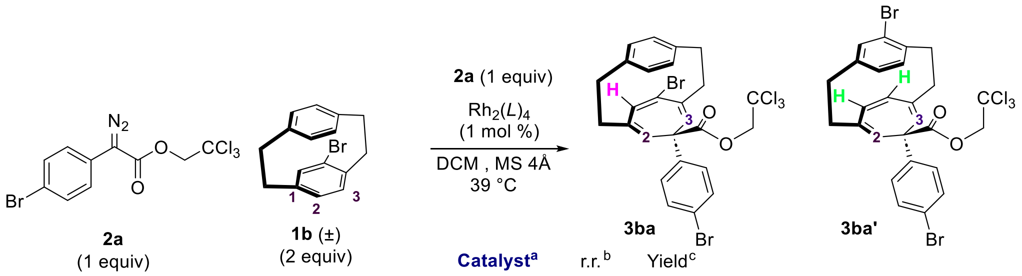

Catalyst[a]	r.r.[b]	Yield[c]
$Rh_2(OBz)_4$	1:1.8	n.d.
Rh_2(S-TPPTTL)$_4$	1:2.9	56%
Rh_2(*S*-*p*PhTPCP)$_4$	1:2.4	n.d.

[a]Reaction conditions: 2a (0.1 mmol), 1b, Rh_2L_4 (1.0 mol%), DCM (0.05 M), MS 4Å 3-hour slow addition. [b]r.r. was determined by crude NMR using signal at 6.0 ppm (1H, s) (pink), and 5.8 ppm (2H, m) (green) (Highlighted hydrogens are for r.r. determination). [c]Isolated yields.

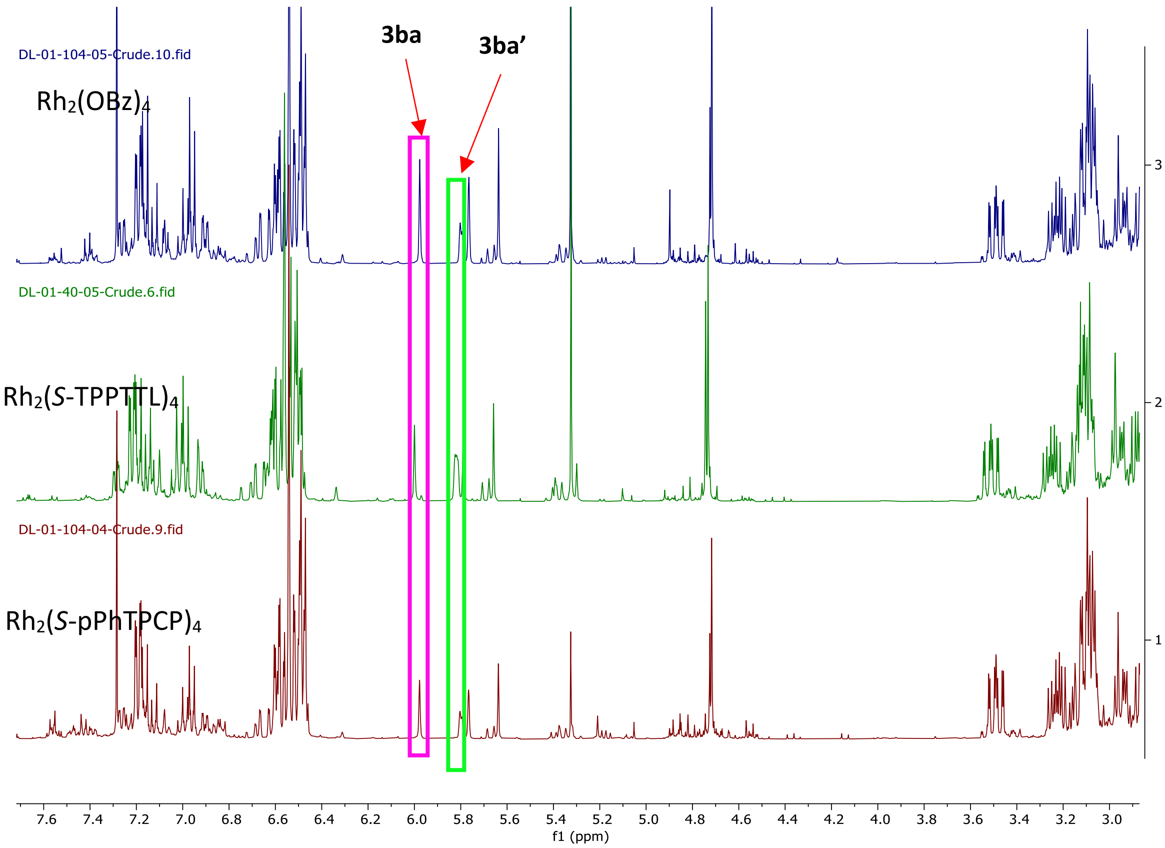

Figure S3. Crude NMR for kinetic resolution cyclopropanation of **1b** with **2a** (Highlighted hydrogens are used for r.r. determination pink for **3ba** and green for **3ba'**)

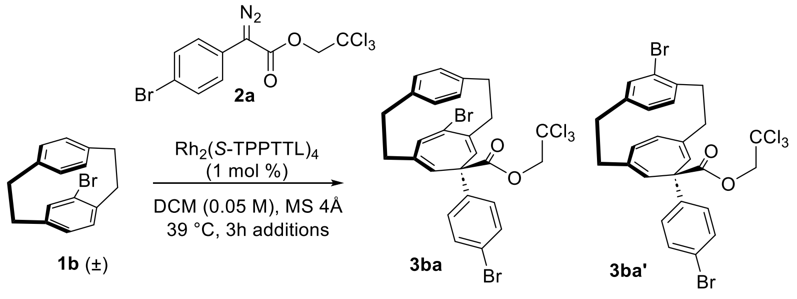

To an 8 ml vial was charged with 12-bromo-1,4(1,4)-dibenzenacyclohexaphane **1b** (115 mg, 0.4 mmol, 2.0 equiv), MS 4Å (100 wt%), and Rh_2(*S*-TPPTTL)$_4$ (5.0 mg, 0.002 mmol, 0.01 equiv). The vial was vacuumed and backfilled with nitrogen 3 times, before 2 ml of dry DCM was added. The mixture was then brought up to reflux (40 °C) by heating block. A solution of 2,2,2-trichloroethyl 2-(4-bromophenyl)-2-diazoacetate (74.5 mg, 0.2 mmol, 1.0 equiv) in 2 ml of dry DCM was added dropwise to the above 8 ml vial within 3 hours by a well-calibrated syringe pump. After 3 hours, the mixture was filtered through celite and washed by DCM to remove MS 4Å. The crude mixture was then dry-loaded on silica, followed by flash chromatography (gradient 0%-10% Et_2O in hexane) afforded **3ba** and **3ba'** as an inseparable mixture (64.5 mg, 51%).

$\mathbf{R_f}$ (9H/1Et_2O) = 0.60

^{1}**H NMR** (400 MHz, $CDCl_3$) δ Major regio-isomer **3ba**: 7.23 (dd, *J* = 7.7, 1.8 Hz, 1H), 7.13 (d, *J* = 8.7 Hz, 2H), 6.93 (d, *J* = 8.7 Hz, 2H), 6.88 (dd, *J* = 7.8, 1.8 Hz, 1H), 6.65 (dd, *J* = 7.9, 1.8 Hz, 1H), 6.59 (dd, *J* = 7.8, 1.8 Hz, 1H), 5.95 (s, 1H), 5.75 – 5.70 (m, 1H), 5.61 (s, 1H), 4.69 (s, 2H). *Minor regio-isomer **3ba'**:* 7.09 (d, *J* = 8.7 Hz, 2H), 7.05 (d, *J* = 1.2 Hz, 1H), 6.96 (d, *J* = 8.7 Hz, 2H), 6.56 (d, *J* = 0.9 Hz, 2H), 5.77 (d, *J* = 3.7 Hz, 2H), 5.70 – 5.62 (m, 1H), 5.33 (d, *J* = 11.2 Hz, 1H), 4.70 (s, 2H). *Mixture of 3ba and 3ba'*: 3.45-2.25 (m, 8H)

^{13}**C NMR** (101 MHz, $CDCl_3$) δ Major regio-isomer 3ba: 172.0, 139.0, 138.9, 138.2, 138.0, 136.8, 136.4, 132.8, 132.2, 130.3, 129.9, 129.4, 128.7, 128.3, 128.0, 127.1, 121.3, 94.6, 74.7, 37.8, 37.2, 33.9, 31.4. Minor regio-isomer 3ba': 172.7, 141.5, 138.5, 138.5, 136.8, 136.5, 134.7, 134.6, 134.1, 132.8, 131.6, 129.9, 129.1, 126.54, 126.52, 124.9, 120.9, 94.8, 74.6, 55.8, 37.0, 34.6, 34.1, 32.6.f

HRMS (+p APCI) calcd. for $[C_{26}H_{22}O_2{}^{79}Br_2{}^{35}Cl_3]$ ($[M+H]^+$) 628.9047 found 628.9049

4.2.2. Kinetic resolution cyclopropanation with 1c

Table S4. Catalyst screening for cyclopropanation of 1c

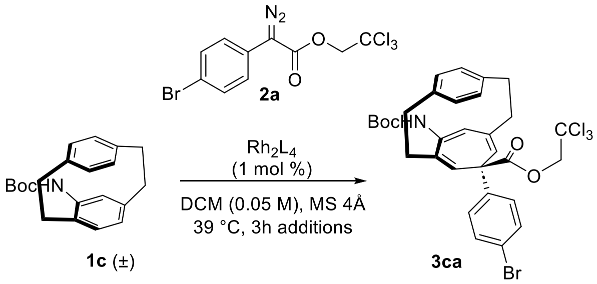

Entry[a]	Catalyst	Yield[b], %	r.r.[c]	ee_P[d], %	ee_S[e], %	s[h]
1	$Rh_2(OBz)_4$	45	20:1	0	0	n.d.
2	Rh_2(*S*-TPPTTL)$_4$	56	2.6:1	32	34	2.6
3	Rh_2(*S*-DOSP)$_4$	54	20:1	-26	-25	2.1
4	Rh_2(*S*-TCPTAD)$_4$	48	20:1	40	36	3.3
5	Rh_2(S-NTTL)$_4$	50	n.d.	73	33	9
6	Rh_2(S-*p-PhT*PCP)$_4$	51	17:1	86	47	21
7[f]	Rh_2(S-*p-PhT*PCP)$_4$	39	n.d.	84	24	14
8[g]	Rh_2(S-*p-PhT*PCP)$_4$	40	n.d.	87	15	17

[a]Reaction conditions: (±) **1c** (0.15 mmol, 1.5 equiv), **2a** (0.1 mmol), Rh_2L_4 (1 mol%), MS 4Å in DCM (0.05 M), 39 °C, 3-h slow addition. [b]NMR yield using 1,3,5-trimethoxybenzene as an internal standard. [c]r.r. was determined by NMR 4.52 ppm (d, 1H); 5.70 ppm (s, 1H).[d]ee_P is enantiomeric excess of **3ca** and was determined by SFC.[e] ee_S is enantiomeric excess of recovered **1c** and was determined by SFC. [f]**1c** (0.2 mmol, 2.0 equiv). [g]**1h** (0.3 mmol, 3 equiv).[h]*s* factor was determined by formular $c = ee_S/(ee_S+ee_P)$, $s = \ln(1-c(1+ee_P))/\ln(1-c\,(1-ee_P))$ or $s = \ln((1-c)(1+ee_S))/\ln((1-c)(1+ee_S))$. n.d. = not determined

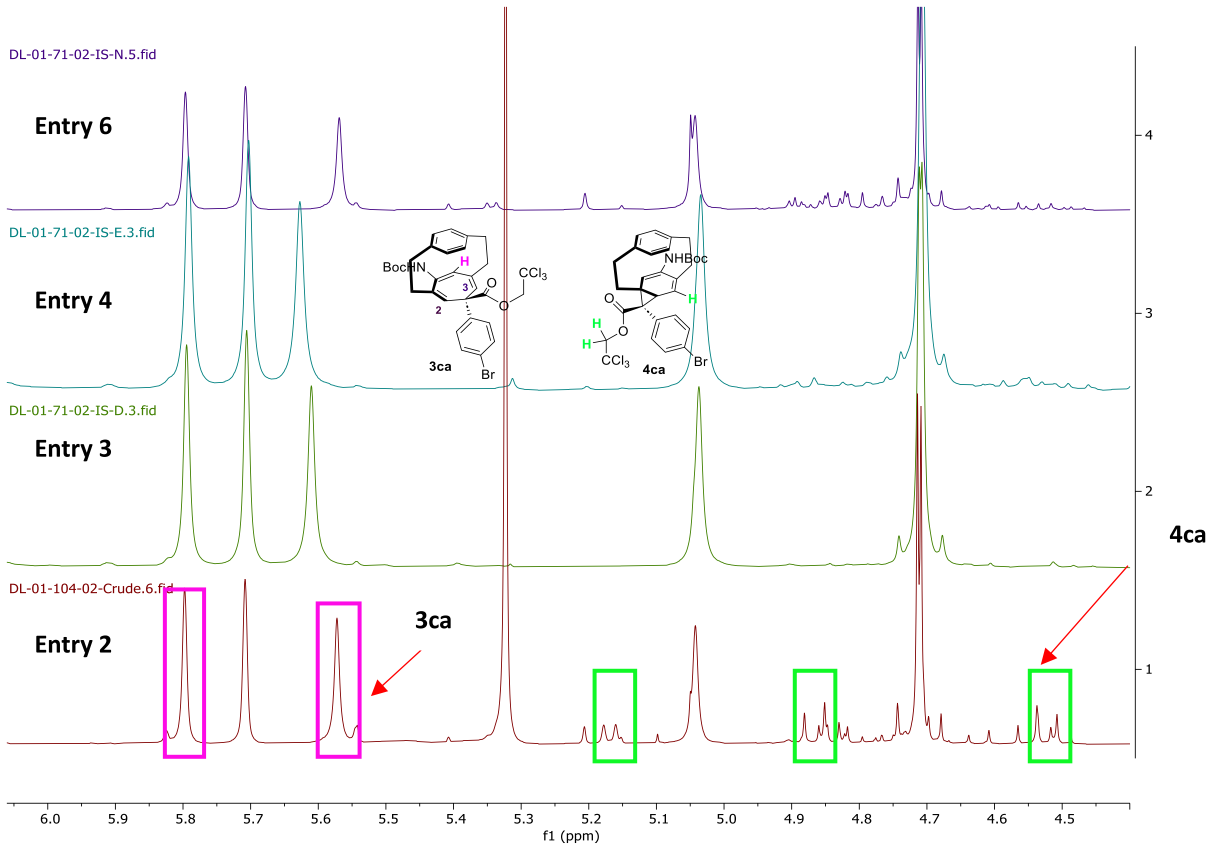

Figure S4. Crude NMR for kinetic resolution cyclopropanation of **1c** with **2a** (Highlighted hydrogens is used for r.r. determination)

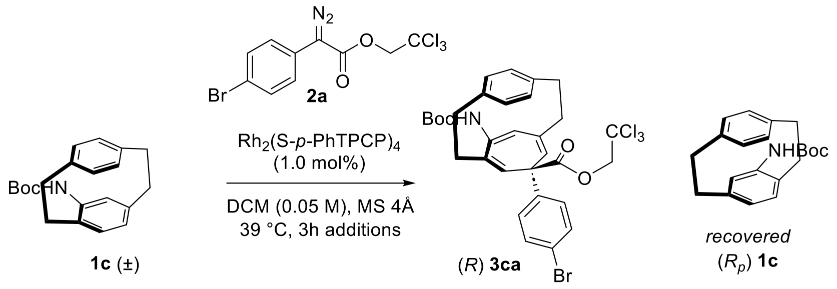

To an 8 ml vial was charged with tert-butyl 1,4(1,4)-dibenzenacyclohexaphane-12-ylcarbamate (±) **1c** (97.0 mg, 0.3 mmol, 1.5 equiv), MS 4Å (100 wt%), and Rh_2(*S*-*p*-PhTPCP)$_4$ (3.5 mg, 0.002 mmol, 0.01 equiv). The vial was vacuumed and backfilled with nitrogen 3 times, before 2 ml of dry DCM was added. The mixture was then brought up to reflux (40 °C) by heating block. A solution of 2,2,2-trichloroethyl 2-(4-bromophenyl)-2-diazoacetate (74.5 mg, 0.2 mmol, 1.0 equiv) in 2 ml of dry DCM was added dropwise to the above 8 ml vial within 3 hours by a well-calibrated syringe pump. After 3 hours, the mixture was filtered through celite and washed by DCM to remove MS 4Å. The crude mixture was then dry-loaded on silica, followed by flash chromatography (gradient 0%-10% DCM in toluene) afforded **3ha** as a fluffy white solid **(**68.5 mg, 51%, 86% ee**)** and recovered starting material (R_p)-**1c** (35.5 mg, 37% recovery**,** 47% ee). ***Note:*** *Absolute stereocenter is assigned based on literature reported*

optical rotation value[14] *of* ***1c**. (R_p)-1c was recovered, therefore (S_p)-**1c** was resolved into (R)* ***3ca***. The *s* factor was estimated to be 20.

R$_f$ (5Toluene/1DCM) = 0.45

^{1}H NMR (400 MHz, $CDCl_3$) δ 7.10 – 7.00 (m, 4H), 6.97 (dd, *J* = 7.8, 1.7 Hz, 1H), 6.82 (dd, *J* = 7.7, 1.7 Hz, 1H), 6.65 (dd, *J* = 7.8, 1.7 Hz, 1H), 6.61 (dd, *J* = 7.9, 1.7 Hz, 1H), 5.77 (s, 1H), 5.69 (s, 1H), 5.52 (s, 1H), 5.03 (s, 1H), 4.69 (ab q, *J* = 2.1 Hz, 2H), 3.11 – 2.73 (m, 7H), 2.39 – 2.25 (m, 1H), 1.44 (s, 9H).

^{13}C NMR (101 MHz, $CDCl_3$) δ 172.7, 152.4, 139.2, 139.0, 138.0, 136.7, 136.0, 132.9, 132.6, 132.2, 130.4, 129.8, 129.0, 128.7, 125.6, 125.0, 123.1, 120.8, 94.8, 80.5, 74.7, 56.0, 37.7, 36.1, 33.6, 32.3, 28.4.

HRMS (+p APCI) calcd. for [$C_{31}H_{32}O_4N^{79}Br^{35}Cl_3$] ($[M+H]^+$) 666.0575 found 666.0576

SFC: (SSWHELK, 10% (50% methanol in isopropanol with 0.2% Formic Acid), 2.5 mL/min, 1.0 mg/ml, 15 min, λ = 230 nm) retention times of 3.79 (major) and 4.74 min (minor) 86% ee.

$[\alpha]^{20}_D$: +92.1° (c = 0.6 g/100 ml, $CHCl_3$, 86% ee)

Recover starting material ***1c***:

^{1}H NMR (400 MHz, $CDCl_3$) δ 6.79 (dd, *J* = 7.8, 2.0 Hz, 1H), 6.73 (s, 1H), 6.52 (ddd, *J* = 14.2, 7.8, 2.0 Hz, 2H), 6.46 – 6.32 (m, 3H), 6.25 (s, 1H), 3.28 – 2.93 (m, 7H), 2.77 (ddd, *J* = 14.1, 10.3, 6.8 Hz, 1H), 1.57 (s, 9H). (NMR matched literature report)[14]

SFC: (SSWHELK, 10% (50% methanol in isopropanol with 0.2% Formic Acid), 2.5 mL/min, 1.0 mg/ml, 15 min, λ = 230 nm) retention times of 2.62 (minor) and 3.79 min (major) 47% ee.

$[\alpha]^{20}_D$: -106.73° (c = 0.26 g/100 ml, $CHCl_3$, 47% ee)

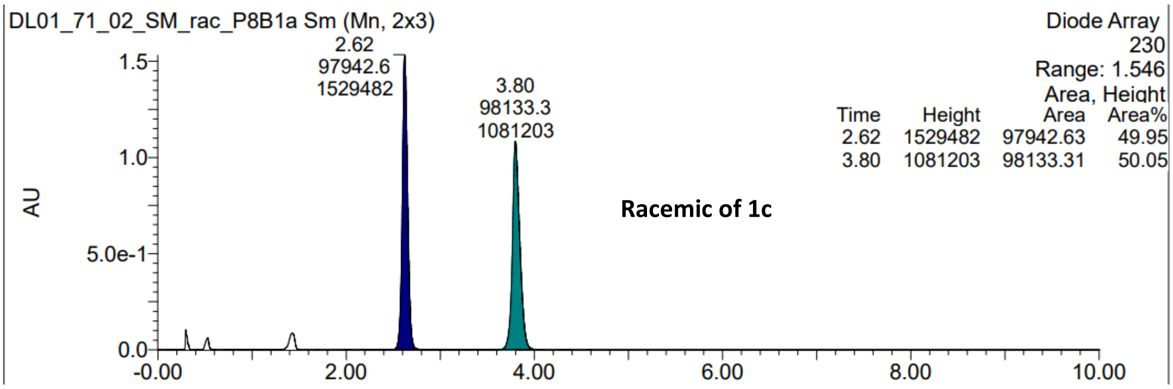

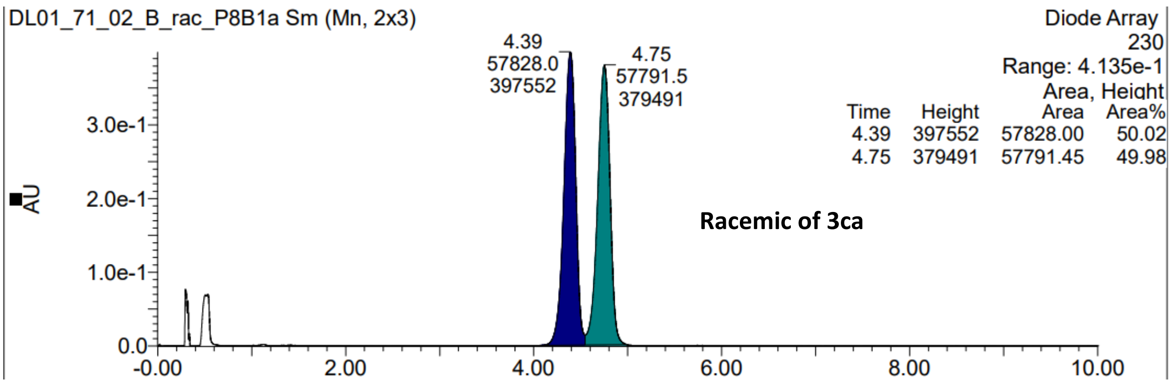

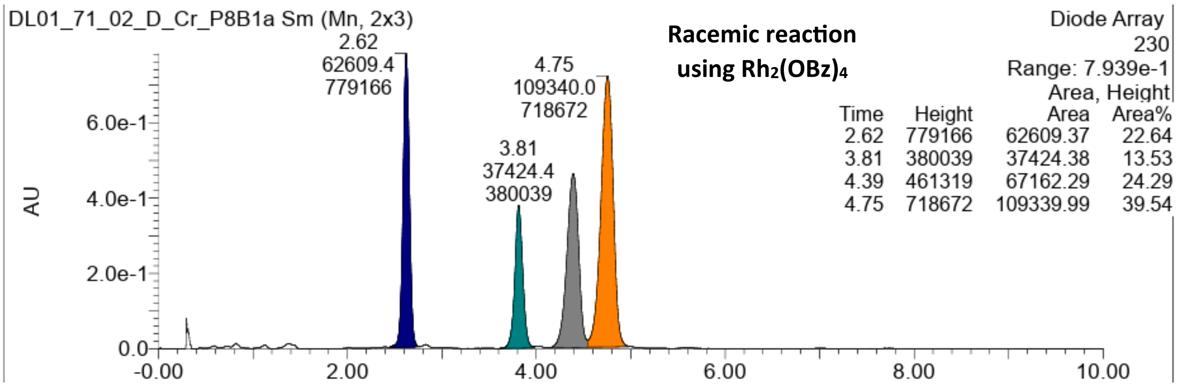

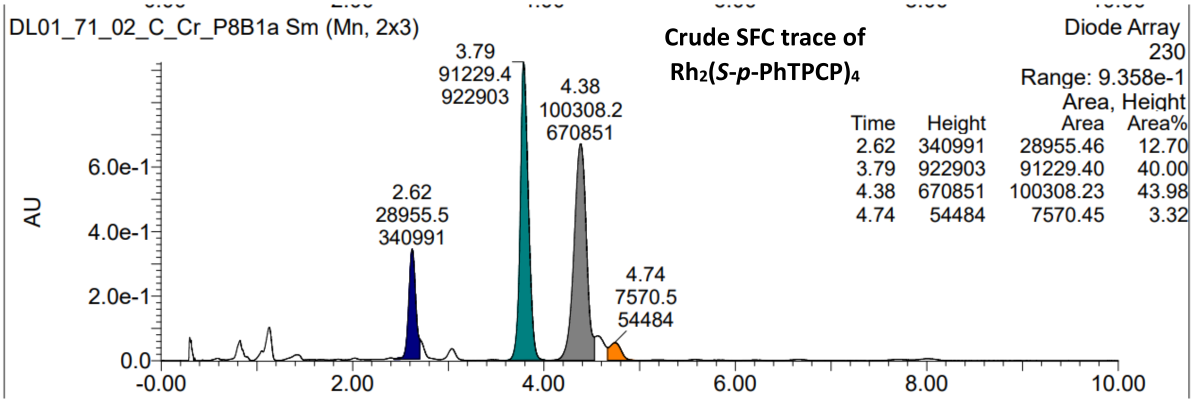

Figure S5. SFC for 3ca and 1c

4.3. Optimization for desymmetrization cyclopropanation of [2.2]paracyclophane

A. Catalyst screening

Table S5. Catalyst screening for cyclopropanation of 1d

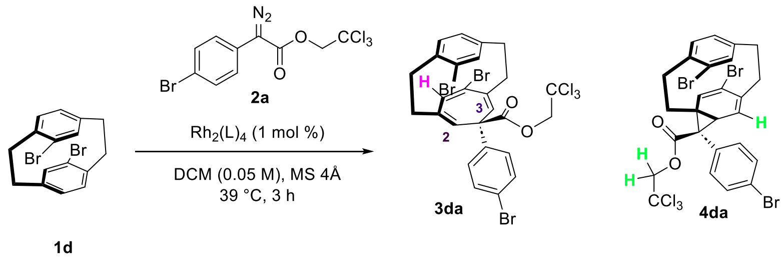

Entry	1d (eq)	2a (eq)	Rh_2L_4 (1 mol%)	T (°C)	Yield[a], %	r.r.[b]	ee, %[c]
01[a]	3.0	1.0	Rh_2(*R*-TPPTTL)$_4$	39	29	>20:1	-90
02	3.0	1.0	Rh_2(*R*-DOSP)$_4$	39	32	5:1	0
03	3.0	1.0	Rh_2(*R*-4-BrTPCP)$_4$	39	16	2:1	n.d.
04	2.0	1.0	Rh_2(*S*-NTTL)$_4$	39	trace	x	x
05	2.0	1.0	Rh_2(*S*-TBPTTL)$_4$	39	trace	x	x
06	2.0	1.0	Rh_2(*S*-TCPTAD)$_4$	39	trace	x	x
07	2.0	1.0	Rh_2(*S*-PTTL)$_4$	39	16	2:1	67
08	2.0	1.0	Rh_2(*S*-PTAD)$_4$	39	16	1:1	67
09	1.0	1.0	Rh_2(*S*-TPPTTL)$_4$	39	29	>20:1	90
10	1.0	1.5	Rh_2(*S*-TPPTTL)$_4$	39	77	>20:1	95
11	1.0	2.0	Rh_2(*S*-TPPTTL)$_4$	39	84	>20:1	96

[a]Reaction conditions (0.1 mmol scale): 1b, Rh_2L_4 (1.0 mol%), DCM (0.05 M), MS 4Å 3-hour slow addition. [b]r.r. was determined by crude NMR using signal at 5.68 ppm (1H, s) for **3da**, and 5.37 ppm (**1H**, s) for **4da**. [c]ee was determined by SFC.

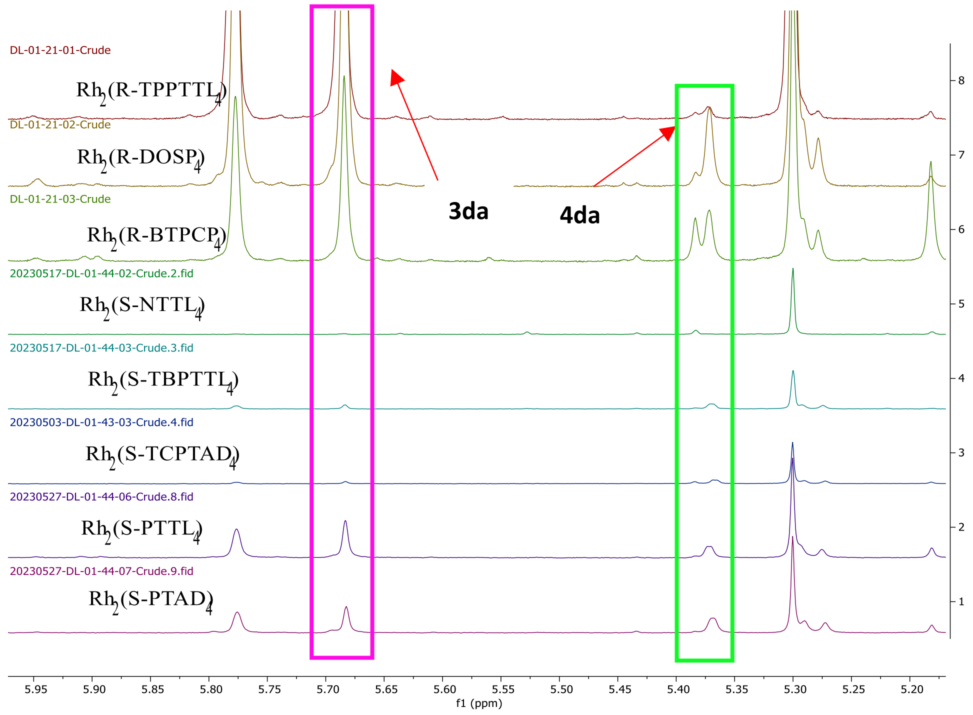

Figure S6. Crude NMR for desymmetrization cyclopropanation of **1d** with **2a** (highlighted hydrogens is used for r.r. determination, pink for **3da** and green for **4da**)

5. C-H insertion of [3.3]paracyclophane

Table S6. Catalyst screening for C-H functionalization of 5

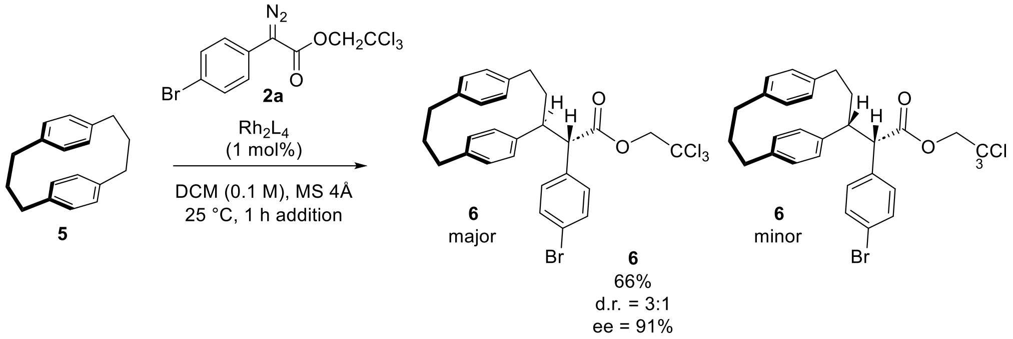

Entry[a]	[3.3]PCP (eq)	2 (eq)	Rh_2L_4 (1 mol%)	T (°C)	Yield[b], %	dr.[c]	ee[d], % (major)	ee[d], % (minor)
01	2.0	1.0	Rh_2(R-DOSP)$_4$	rt	59	3:1	50	0
02	2.0	1.0	Rh_2(R-TPPTTL)$_4$	rt	66	3:1	91	77
03	2.0	1.0	Rh_2(oct)$_4$	rt	51	2:1	0	0

[a]Reaction conditions: **1** (0.2 mmol), **2** (0.1 mmol), 1 mol% catalyst, MS 4Å in 2 ml DCM, rt. [b]Isolated yield. [c]d.r. was determined by crude NMR. [d]ee was determined by SFC.

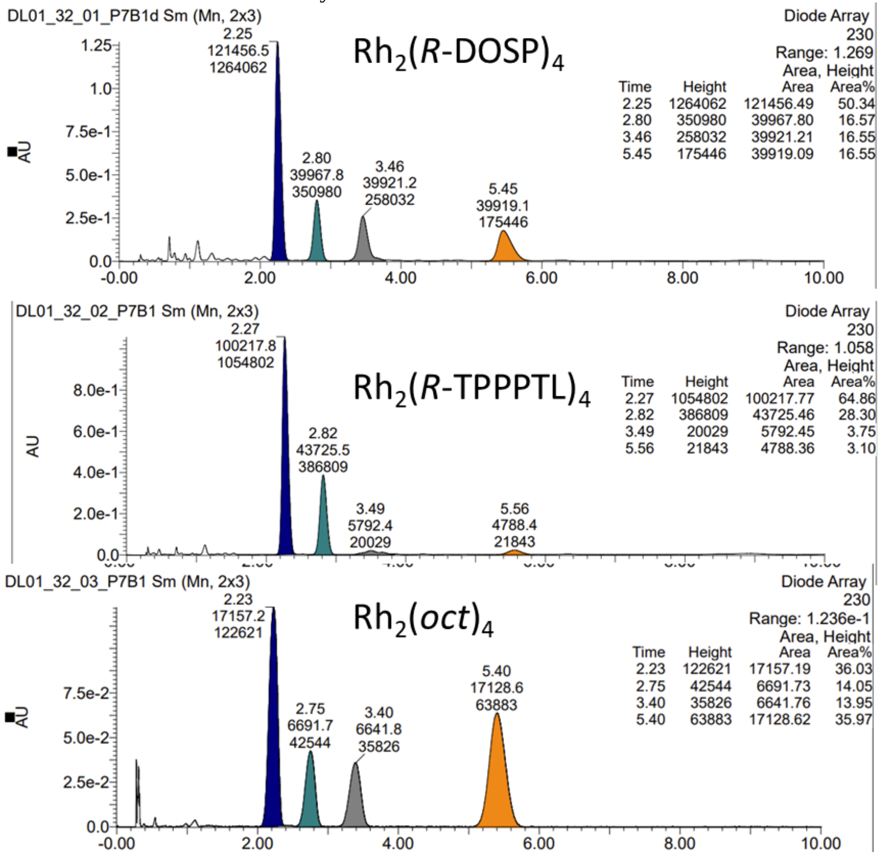

Figure S7. SFC for ee determination of **6** (ChiralCel OJ-3)

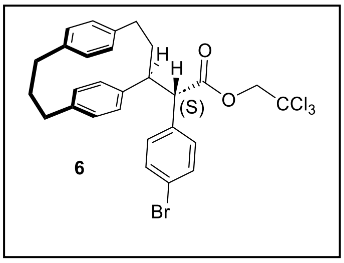

2,2,2-trichloroethyl 2-(1,5(1,4)-dibenzenacyclooctaphane-2-yl)-2-(4-bromophenyl)acetate (6)

A flame-dried clean 8.0 ml vial equipped with a magnetic stir-bar was evacuated and back-filled with nitrogen 3 times. After cooling down to room temperature, the corresponding Rh_2(*R*-TPPTTL)$_4$ (1 mol %) followed by [3.3]paracyclophane (47.3 mg, 0.2 mmol, 2.0 equiv), and MS 4Å (100 wt%) were then added. The vial was once again evacuated and purged with argon (3-5 times) and dried dichloromethane (1 mL) was added. The diazo solution was prepared by of 2,2,2-trichloroethyl 2-(4-bromophenyl)-2-diazoacetate (37.2 mg, 0.1 mmol, 1.0 equiv) to the other 8 ml vial. The vial was evacuated and purged with argon (2-3 times) and of dry dichloromethane (1.0 mL) was then added to obtain a 0.1 M solution of the diazo compound. The 0.1 M solution was transferred into a plastic syringe (3 mL, 9.83 mm diameter). The 0.1 M solution of diazo was then added slowly to a catalyst and trap solution within 1 hour by using a well-calibrated syringe pump (1.00 mL/h). After complete addition (1 hour later), the residual diazo compound in the 3.0 mL plastic syringe was rinsed with 0.5 mL dry dichloromethane and transferred dropwise into the stirring reaction mixture of vial-A. The mixture was stirred for an additional 15 min and then concentrated under reduced pressure. The crude product was filtered through celite to remove dust from MS 4Å then dried load on silica and purified by flash chromatography to afford clear oil **6** as a 3:1 mixture of two diastereomer (38.6 mg, 66.5%). (*Note: The diastereomers were not able to be separated, NMR was analyzed by mixture, the NMR got complicated in the aliphatic region due to the slow equilibrium of boat and chair conformations of [3.3]paracyclophane*). *Absolute stereochemistry is tentatively assigned based on analogy with reaction with Rh_2(R-DOSP)$_4$*

R_f (2H/1DCM) = 0.35

^{1}H NMR (800 MHz, $CDCl_3$) δ Major diastereomer 7.20 (d, *J* = 8.6 Hz, 2H), 7.05 (d, *J* = 8.6 Hz, 2H), 6.72 – 6.69 (m, 2H) (***overlap with minor***), 6.63 – 6.61 (m, 2H) (***overlap with minor***), 6.61 (d, *J* = 7.7 Hz, 1H), 6.53 (d, *J* = 7.6 Hz, 1H), 6.50 (d, *J* = 7.7 Hz, 1H), 6.31 (d, *J* = 7.6 Hz, 1H), 4.82 (d, *J* = 1.3 Hz, 1H), 4.74 (d, *J* = 11.9 Hz, 1H), 3.85 (d, *J* = 11.5 Hz, 1H), 3.32 (t, *J* = 11.2 Hz, 1H), 3.07 (d, *J* = 14.6 Hz, 1H), 2.83 – 2.62 (m, 4H) (***overlap with minor***), 2.50 (t, *J* = 13.6 Hz, 1H), 2.20 – 2.12 (m, 1H), 2.11 – 2.03 (m, 3H) (***overlap with minor***). **Minor diastereomer** 7.53 (d, *J* = 8.4 Hz, 2H), 7.41 (d, *J* = 8.0 Hz, 2H), 6.79 (d, *J* = 7.9 Hz, 1H), 6.75 (d, *J* = 7.1 Hz, 1H), 6.71 (m, *J* = 7.9 Hz, 2H) (***overlap with major***), 6.67 (d, *J* = 7.9 Hz, 1H), 6.65 (d, *J* = 7.6 Hz, 1H), 6.62 (m, 2H) (***overlap with major***), 4.40 (d, *J* = 12.1 Hz, 1H), 4.28 (d, *J* = 12.1, 1H), 3.86 (d, *J* = Hz, 1H) (***overlap with major***), 3.24 (dt, *J* = 12.8, 6.5 Hz, 1H), 2.91 (dt, *J* = 14.5, 4.3 Hz, 1H), 2.62-2.55 (m, 4H), 2.32 – 2.25 (m, 1H), 2.18 – 2.15 (m, 1H), 2.04 – 1.95 (m, 1H), 1.74 (q, *J* = 6.1 Hz, 2H). *The assignment is based on COSEY, HSQC, and HMBC.*

^{13}C NMR (201 MHz, $CDCl_3$) δ Major diastereomer 172.0, 139.5, 138.7, 137.5, 136.9, 135.4, 132.5, 131.5, 131.4, 130.6, 130.3, 129.6, 128.4, 121.5, 94.9, 74.4, 58.2, 49.9, 36.1, 35.9, 35.4, 35.1, 29.90. **Minor diastereomer** 170.6, 140.0, 138.7, 137.8, 136.0, 132.3, 132.1, 131.9, 131.6, 131.4, 130.7, 130.3, 129.0, 122.1, 94.6, 74.1, 58.7, 49.9, 36.1, 36.0, 35.2, 32.8, 29.9. (missing 3 carbons for major and 3 for minor, presumably due to the broadened signal 130.5-129.5 ppm – a chair and boat equilibrium) *The assignment based on COSEY, HSQC, and HMBC.*

HRMS (+p APCI) calcd. for [$C_{28}H_{25}O_2{}^{79}Br^{35}Cl_3$] ($[M+H]^+$) 577.0109 found 577.0112

SFC: (ChiralCel OJ-3, 10% (50% methanol in isopropanol with 0.2% Formic Acid), 2.5 mL/min, 1.0 mg/ml, 10 min, λ = 230 nm) retention times of for major diastereomer 2.23 (major) and 5.56 min (major) 91% ee for minor diastereomer 2.82 min (major) and 3.49 min (minor) 77% ee.

$[\alpha]^{20}_D$: +82.6° (c = 0.53 g/100 ml, $CHCl_3$, 91% ee (major) and 77% ee (minor), a mixture of 3:1)

6. Rhodium catalyzed cyclopropanation of [2.2]paracyclophane and characterization of product.

General Procedure A

To a flame-dried clean 8.0 ml vials equipped with a magnetic stir-bar was evacuated and back-filled with nitrogen 3 times. After cooling down to room temperature, the corresponding dirhodium catalyst (1 mol %) followed by [2.2]paracyclophane (124.8 mg, 0.6 mmol, 3.0 equiv), and MS 4Å (100 wt%) were then added. The vial was once again evacuated and purged with argon (3-5 times) and dried dichloromethane (1 mL) was added. The vial and its contents were then set to stir at 40 °C by heating block under an nitrogen atmosphere. The diazo solution was prepared by adding diazo compound (0.2 mmol, 1 equiv) to the other 8 ml vial. The vial was evacuated and purged with argon (2-3 times) and of dry dichloromethane (1.0 mL) was then added to obtain a 0.2 M solution of the diazo compound. The 0.2 M solution was transferred into a plastic syringe (3 mL, 9.83 mm diameter). The 0.2 M solution of diazo was then added slowly to a catalyst and trap solution within 1 hour by using a well-calibrated syringe pump (1.00 mL/h). After complete addition (1 hour later), the residual diazo compound in the 3.0 mL plastic syringe was rinsed with 0.5 mL dry dichloromethane and transferred dropwise into the stirring reaction mixture of vial-A. The mixture was stirred for an additional 15 min and then concentrated under reduced pressure. The crude product was filtered through celite to remove dust from MS 4Å then dried load on silica and purified by flash chromatography to obtain the purified product.

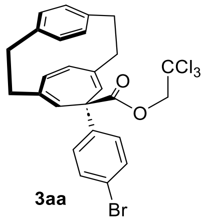
3aa

2,2,2-trichloroethyl (Z)-16-(4-bromophenyl)-1(1,4)-cycloheptana-4(1,4) benzenacyclohexaphane-11(17),12,14-triene-16-carboxylate (3aa)

Prepared according to general procedure A. [2.2]Paracyclophane (125 mg, 0.6 mmol, 3.0 equiv), MS 4Å (100 wt%), $Rh_2(OBz)_4$ (1.4 mg, 0.0002 mmol, 0.001 equiv), and 2,2,2-trichloroethyl 2-(4-bromophenyl)-2-diazoacetate (74.5 mg, 0.2 mmol, 1.0 equiv) were used. The crude mixture was then dry-loaded on silica, followed by flash chromatography (gradient 5%-30% DCM in hexane) afforded **3aa** as a white solid (71.8 mg, 65% yield) and **4aa** as a white solid by-product (9.0 mg, 8%)

R_f (2H/1DCM) = 0.34

^{1}H NMR (400 MHz, $CDCl_3$) δ 7.11 (d, J = 8.6 Hz, 1H), 6.99 (d, J = 8.6 Hz, 2H), 6.91 (d, J = 1.6 Hz, 2H), 6.61 (d, J = 1.6 Hz, 2H), 5.79 (s, 2H), 5.28 (s, 2H), 4.72 (s, 2H), 3.02 – 2.88 (m, 6H), 2.65 – 2.51 (m, 2H).

^{13}C NMR (101 MHz, CDCl3) δ 172.7, 139.2, 138.6, 136.4, 134.2, 132.8, 130.0, 129.9, 129.8, 126.1, 120.7, 94.8, 74.5, 55.8, 37.3, 33.4.

HRMS (+p APCI) calcd. for[$C_{26}H_{23}O_2{}^{79}Br^{35}Cl_3$] ($[M+H]^+$) 550.9942, found 550.9940

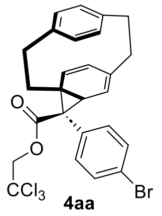
4aa

4aa Characterization *(Minor isomer)*

R_f (2H/1DCM) = 0.32

^{1}H NMR (400 MHz, $CDCl_3$) δ 7.21 – 7.11 (m, 3H), 7.00 (d, *J* = 7.7 Hz, 1H), 6.88 – 6.75 (m, 4H), 5.09 (d, *J* = 6.9 Hz, 1H), 4.97 (d, *J* = 9.8 Hz, 1H), 4.90 – 4.81 (m, 2H), 4.51 (d, *J* = 11.9 Hz, 1H), 3.15 – 2.89 (m, 4H), 2.78 (dt, *J* = 13.9, 8.3 Hz, 1H), 2.40 – 2.09 (m, 4H).

^{13}C NMR (101 MHz, CDCl3) δ 171.9, 139.7, 139.5, 135.2, 133.8, 133.2, 132.7, 132.6, 132.0, 131.0, 130.0, 128.8, 128.0, 120.7, 95.0, 74.6, 42.5, 41.3, 35.0, 34.9, 34.5, 32.4.

HRMS (+p APCI) calcd. for[$C_{26}H_{23}O_2{}^{79}Br^{35}Cl_3$] ($[M+H]^+$) 550.9942, found 550.9939

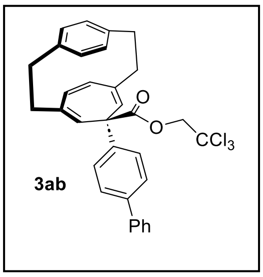

2,2,2-trichloroethyl (11R,17S,Z)-17-([1,1'-biphenyl]-4-yl)-1(1,4)-bicyclo[4.1.0]heptana-4(1,4)-benzenacyclohexaphane-12,14-diene-17-carboxylate (3ab)

Prepared according to general procedure A. [2.2]Paracyclophane (125 mg, 0.6 mmol, 3.0 equiv), MS 4Å (100 wt%), $Rh_2(OBz)_4$ (1.4 mg, 0.0002 mmol, 0.001 equiv), and 2,2,2-trichloroethyl 2-([1,1'-biphenyl]-4-yl)-2-diazoacetate (73.9 mg, 0.2 mmol, 1.0 equiv) were used. The crude mixture was then dry-loaded on silica, followed by flash chromatography (gradient 10%-40% DCM in hexane) afforded **3ab** as a white solid (62.1 mg, 57% yield).

R_f (3H/2DCM) = 0.30

^{1}H NMR (400 MHz, $CDCl_3$) δ 7.49 (d, *J* = 7.0 Hz, 2H), 7.38 (t, *J* = 7.6 Hz, 2H), 7.33 – 7.27 (m, 1H), 7.25 (d, *J* = 8.5 Hz, 2H), 7.18 (d, *J* = 8.5 Hz, 2H), 6.94 (d, *J* = 1.5 Hz, 2H), 6.66 (d, *J* = 1.6 Hz, 2H), 5.87 (s, 2H), 5.33 (s, 2H), 4.76 (s, 2H), 3.00 (hept, *J* = 3.6 Hz, 6H), 2.70 – 2.56 (m, 2H).

^{13}C NMR (101 MHz, $CDCl_3$) δ 173.2, 140.7, 139.5, 139.3, 138.6, 136.2, 134.1, 132.9, 130.0, 128.6, 128.5, 127.1, 126.9, 126.4, 125.4, 94.8, 74.5, 56.0, 37.3, 33.4.

HRMS (+p APCI) calcd. for $[C_{32}H_{28}O_2{}^{35}Cl_3]$ ($[M+H]^+$) 549.1149 found 549.1149

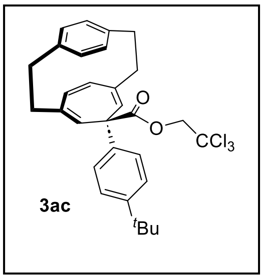

2,2,2-trichloroethyl (Z)-16-(4-(tert-butyl)phenyl)-1(1,4)-cycloheptana-4(1,4)-benzenacyclohexaphane-11(17),12,14-triene-16-carboxylate (3ac)

Prepared according to general procedure A. [2.2]Paracyclophane (125 mg, 0.6 mmol, 3.0 equiv), MS 4Å (100 wt%), $Rh_2(OBz)_4$ (1.4 mg, 0.0002 mmol, 0.001 equiv), and 2,2,2-trichloroethyl 2-(4-(tert-butyl)phenyl)-2-diazoacetate (69.9 mg, 0.2 mmol, 1.0 equiv) were used. The crude mixture was then dry-loaded on silica, followed by flash chromatography (gradient 10%-33% DCM in hexane) afforded **3ac** as an off-white solid (32.2 mg, 31% yield).

R_f (2H/1DCM) = 0.15

^{1}H NMR (400 MHz, $CDCl_3$) δ 7.00 – 6.92 (m, 4H), 6.89 (s, 2H), 6.60 (s, 2H), 5.79 (s, 2H), 5.26 (s, 2H), 4.70 (s, 2H), 3.01 – 2.86 (m, 6H), 2.65 – 2.49 (m, 2H), 1.16 (s, 9H).

^{13}C NMR (101 MHz, $CDCl_3$) δ 173.4, 149.4, 139.3, 136.2, 135.9, 134.0, 132.9, 130.0, 127.6, 126.5, 123.5, 94.9, 74.3, 55.9, 37.3, 34.2, 33.4, 31.3

HRMS (+p APCI) calcd. for $[C_{30}H_{32}O_2{}^{35}Cl_3]$ ($[M+H]^+$) 529.1462 found 529.1469

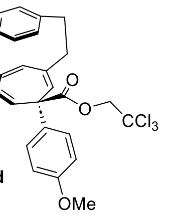

3ad

2,2,2-trichloroethyl (Z)-16-(4-methoxyphenyl)-1(1,4)-cycloheptana-4(1,4)-benzenacyclohexaphane-11(17),12,14-triene-16-carboxylate (3ad)

Prepared according to general procedure A. [2.2]Paracyclophane (125 mg, 0.6 mmol, 3.0 equiv), MS 4Å (100 wt%), $Rh_2(OBz)_4$ (1.4 mg, 0.0002 mmol, 0.001 equiv), and 2,2,2-trichloroethyl 2-diazo-2-(4-methoxyphenyl)acetate (64.7 mg, 0.2 mmol, 1.0 equiv) were used. The crude mixture was then dry loaded on silica, followed by flash chromatography (gradient 5-40% DCM in hexane) afforded **3ad** as a white crystalline solid (38.0 mg, 39% yield).

R_f (1H/2DCM) = 0.60

^{1}H NMR (400 MHz, $CDCl_3$) δ 6.99 (d, *J* = 8.9 Hz, 1H), 6.91 – 6.86 (m, 1H), 6.62 – 6.58 (m, 1H), 6.51 (d, *J* = 8.9 Hz, 1H), 5.79 (s, 1H), 5.27 (t, *J* = 1.0 Hz, 1H), 4.70 (s, 1H), 3.66 (s, 2H), 3.00 – 2.86 (m, 3H), 2.63 – 2.49 (m, 1H).

^{13}C NMR (101 MHz, $CDCl_3$) δ 173.4, 158.3, 139.3, 136.0, 134.0, 132.9, 131.3, 130.0, 129.1, 126.7, 112.0, 94.9, 74.4, 55.5, 55.0, 37.3, 33.4.

HRMS (+p APCI) calcd. for $[C_{27}H_{26}O_3{}^{35}Cl_3]$ ($[M+H]^+$) 503.0942 found 503.0948

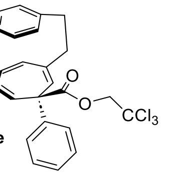

3ae

2,2,2-trichloroethyl (Z)-16-phenyl-1(1,4)-cycloheptana-4(1,4)-benzenacyclohexaphane-11(17),12,14-triene-16-carboxylate (3ae)

Prepared according to general procedure A. To an 8 ml vial was charged with [2.2]Paracyclophane (125 mg, 0.6 mmol, 3.0 equiv), MS 4Å (100 wt%), $Rh_2(OBz)_4$ (1.4 mg, 0.0002 mmol, 0.001 equiv), and 2,2,2-trichloroethyl 2-diazo-2-phenylacetate (58.7 mg, 0.2 mmol, 1.0 equiv) were used. The crude mixture was then dry loaded on silica, followed by flash chromatography (gradient 10-33% DCM in hexane) afforded **3ae** as a white crystalline solid (35.8 mg, 37% yield).

R_f (3H/2DCM) = 0.51

^{1}H NMR (400 MHz, $CDCl_3$) δ 7.13 – 7.06 (m, 2H), 7.05 – 6.93 (m, 3H), 6.89 (s, 2H), 6.60 (s, 2H), 5.80 (s, 2H), 5.26 (s, 2H), 4.70 (s, 2H), 3.00 – 2.88 (m, 6H), 2.64 – 2.50 (m, 2H).

^{13}C NMR (201 MHz, $CDCl_3$) δ 173.3, 139.6, 139.4, 136.2, 134.2, 133.0, 130.1, 128.3, 126.9, 126.7, 126.6, 94.9, 74.6, 56.3, 37.4, 33.5.

HRMS (+p APCI) calcd. for $[C_{26}H_{24}O_2{}^{35}Cl_3]$ ($[M+H]^+$) 473.0836 found 473.0839

2,2,2-trichloroethyl (Z)-16-(4-nitrophenyl)-1(1,4)-cycloheptana-4(1,4)-benzenacyclohexaphane-11(17),12,14-triene-16-carboxylate (3af)

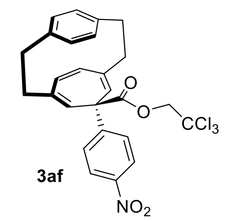

Prepared according to general procedure A. To an 8 ml vial was charged with [2.2]Paracyclophane (125 mg, 0.6 mmol, 3.0 equiv), MS 4Å (100 wt%), $Rh_2(OBz)_4$ (1.4 mg, 0.0002 mmol, 0.001 equiv), and 2,2,2-trichloroethyl 2-diazo-2-(4-nitrophenyl)acetate (67.7 mg, 0.2 mmol, 1.0 equiv) were used. The crude mixture was then dry loaded on silica, followed by flash chromatography (gradient 10-50% DCM in hexane) afforded **3af** as a yellow crystalline solid (53.0 mg, 51% yield).

$\mathbf{R_f}$ (2DCM/1H) = 0.5

^{1}H NMR (400 MHz, $CDCl_3$) δ 7.83 (d, J = 8.8 Hz, 2H), 7.26 (d, J = 8.9 Hz, 2H), 6.89 (s, 2H), 6.60 (s, 2H), 5.79 (s, 2H), 5.26 (s, 2H), 4.72 (s, 2H), 3.06 – 2.84 (m, 6H), 2.69 – 2.49 (m, 2H).

^{13}C NMR (101 MHz, $CDCl_3$) δ 171.9, 147.5, 146.8, 139.2, 137.0, 134.3, 132.8, 130.0, 129.0, 125.6, 121.8, 94.5, 74.6, 56.2, 37.2, 33.3.

HRMS (+p APCI) calcd. for [$C_{26}H_{23}O_4N^{35}Cl_3$] ([M+H]$^+$) 518.0687 found 518.0690

2,2,2-trichloroethyl (Z)-16-(4-(trifluoromethyl)phenyl)-1(1,4)-cycloheptana-4(1,4)-benzenacyclohexaphane-11(17),12,14-triene-16-carboxylate (3ag)

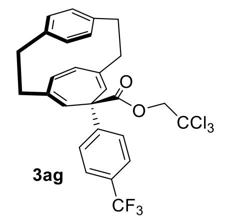

Prepared according to general procedure A. To an 8 ml vial was charged with [2.2]Paracyclophane (125 mg, 0.6 mmol, 3.0 equiv), MS 4Å (100 wt%), and $Rh_2(OBz)_4$ (1.4 mg, 0.0002 mmol, 0.001 equiv), and 2,2,2-trichloroethyl 2-diazo-2-(4-(trifluoromethyl)phenyl)acetate (72.3 mg, 0.2 mmol, 1.0 equiv) were used. The crude mixture was then dry loaded on silica, followed by flash chromatography (gradient 10-33% DCM in hexane) afforded **3ag** as a white crystalline solid (59.0 mg, 55% yield).

$\mathbf{R_f}$ (3H/2DCM) = 0.6

^{1}H NMR (400 MHz, $CDCl_3$) δ 7.22 (s, 4H), 6.90 (s, 2H), 6.61 (d, J = 1.7 Hz, 2H), 5.80 (s, 2H), 5.26 (s, 2H), 4.71 (s, 2H), 3.03 – 2.88 (m, 6H), 2.66 – 2.52 (m, 2H).

^{13}C NMR (101 MHz, $CDCl_3$) δ 172.5, 143.8, 139.2, 136.7, 134.2, 132.8, 130.0, 128.9 (q, J = 32.4 Hz), 128.4, 125.9, 124.1 (q, J = 272.0 Hz), 123.6 (q, J = 3.8 Hz), 94.6, 74.5, 56.1, 37.2, 33.3.

^{19}F NMR (376 MHz, $CDCl_3$) δ -62.5.

HRMS (+p APCI) calcd. for [$C_{27}H_{23}O_2{}^{35}Cl_3F_3$] ([M+H]$^+$) 541.0710 found 541.0716

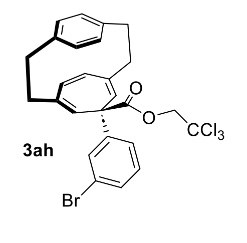

2,2,2-trichloroethyl (Z)-16-(3-bromophenyl)-1(1,4)-cycloheptana-4(1,4)-benzenacyclohexaphane-11(17),12,14-triene-16-carboxylate (3ah)

Prepared according to general procedure A. To an 8 ml vial was charged with [2.2]Paracyclophane (125 mg, 0.6 mmol, 3.0 equiv), MS 4Å (100 wt%), and $Rh_2(OBz)_4$ (1.4 mg, 0.0002 mmol, 0.001 equiv), and 2,2,2-trichloroethyl 2-(3-bromophenyl)-2-diazoacetate (74.5 mg, 0.2 mmol, 1.0 equiv) were used. The crude mixture was then dry loaded on silica, followed by flash chromatography (gradient 10-40% DCM in hexane) afforded **3ah** as a white crystalline solid (52.7 mg, 47% yield).

R_f (3H/2DCM) = 0.50

^{1}H NMR (400 MHz, $CDCl_3$) δ 7.23 (t, J = 1.9 Hz, 1H), 7.14 (d, J = 7.8 Hz, 1H), 7.02 (d, J = 8.1 Hz, 1H), 6.90 (s, 2H), 6.83 (t, J = 7.9 Hz, 1H), 6.60 (s, 2H), 5.76 (s, 2H), 5.29 (s, 2H), 4.72 (s, 2H), 3.04 – 2.85 (m, 6H), 2.66 – 2.50 (m, 2H).

^{13}C NMR (101 MHz, $CDCl_3$) δ 172.6, 142.0, 139.2, 136.6, 134.2, 132.8, 131.2, 130.0, 130.0, 128.1, 126.7, 125.8, 121.1, 94.7, 74.5, 56.0, 37.3, 33.3.

HRMS (+p APCI) calcd. for $[C_{26}H_{23}O_2{}^{79}Br^{35}Cl_3]$ ($[M+H]^+$) 550.9942 found 550.9947

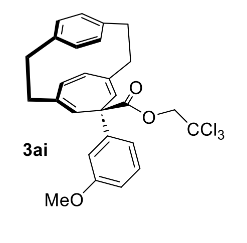

2,2,2-trichloroethyl (Z)-16-(3-methoxyphenyl)-1(1,4)-cycloheptana-4(1,4)-benzenacyclohexaphane-11(17),12,14-triene-16-carboxylate (3ai)

Prepared according to general procedure A. [2.2]Paracyclophane (125 mg, 0.6 mmol, 3.0 equiv), MS 4Å (100 wt%), $Rh_2(OBz)_4$ (1.4 mg, 0.0002 mmol, 0.001 equiv), and 2,2,2-trichloroethyl 2-diazo-2-(3-methoxyphenyl)acetate (64.7 mg, 0.2 mmol, 1.0 equiv) were used The crude mixture was then dry loaded on silica, followed by flash chromatography (gradient 10-40% DCM in hexane) afforded **3ai** as a white crystalline solid (41.3 mg, 41% yield).

R_f (2H/3DCM) = 0.41

^{1}H NMR (400 MHz, $CDCl_3$) δ 6.92 – 6.84 (m, 3H), 6.71 – 6.62 (m, 2H), 6.62 – 6.57 (m, 2H), 6.55 (ddd, J = 8.1, 2.6, 1.0 Hz, 1H), 5.77 (s, 2H), 5.27 (d, J = 1.0 Hz, 2H), 4.70 (s, 2H), 3.64 (s, 3H), 3.00 – 2.86 (m, 6H), 2.64 – 2.49 (m, 2H).

^{13}C NMR (201 MHz, $CDCl_3$) δ 173.2, 158.3, 141.3, 139.4, 136.3, 134.1, 133.0, 130.1, 127.5, 126.4, 121.1, 114.8, 111.9, 95.0, 74.6, 56.3, 55.2, 37.4, 33.5.

HRMS (+p APCI) calcd. for $[C_{27}H_{26}O_3{}^{35}Cl_3]$ ($[M+H]^+$) 503.0942 found 503.0942

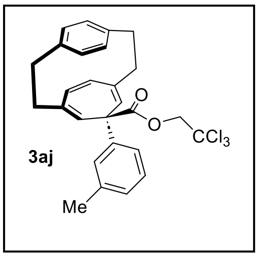

2,2,2-trichloroethyl (Z)-16-(m-tolyl)-1(1,4)-cycloheptana-4(1,4)-benzenacyclohexaphane-11(17),12,14-triene-16-carboxylate (3aj)

Prepared according to general procedure A. [2.2]Paracyclophane (125 mg, 0.6 mmol, 3.0 equiv), MS 4Å (100 wt%), $Rh_2(OBz)_4$ (1.4 mg, 0.0002 mmol, 0.001 equiv), and 2,2,2-trichloroethyl 2-diazo-2-(m-tolyl)acetate (61.5 mg, 0.2 mmol, 1.0 equiv) were used. The crude mixture was then dry loaded on silica, followed by flash chromatography (gradient 10-40% DCM in hexane) afforded **3aj** as a white crystalline solid (41.5 mg, 42% yield).

R_f (2H/1DCM) = 0.35

^{1}H NMR (400 MHz, $CDCl_3$) δ 6.96 – 6.77 (m, 6H), 6.62 (s, 2H), 5.80 (s, 2H), 5.28 (s, 2H), 4.71 (s, 2H), 3.01 – 2.89 (m, 6H), 2.65 – 2.51 (m, 2H), 2.15 (s, 3H).

^{13}C NMR (101 MHz, $CDCl_3$) δ 173.3, 139.3, 139.3, 136.1, 136.0, 134.0, 132.9, 130.0, 128.9, 127.7, 126.5, 126.4, 125.2, 94.9, 74.4, 56.2, 37.3, 33.4, 21.5.

HRMS (+p APCI) calcd. for [$C_{27}H_{26}O_2{}^{35}Cl_3$] ([M+H]$^+$) 487.0993 found 487.0995

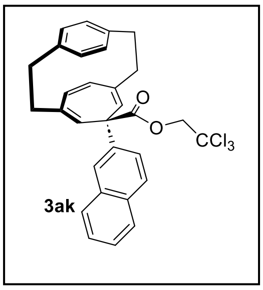

2,2,2-trichloroethyl (Z)-16-(naphthalen-2-yl)-1(1,4)-cycloheptana-4(1,4)-benzenacyclohexaphane-11(17),12,14-triene-16-carboxylate (3ak)

Prepared according to general procedure A. [2.2]Paracyclophane (125 mg, 0.6 mmol, 3.0 equiv), MS 4Å (100 wt%), $Rh_2(OBz)_4$ (1.4 mg, 0.0002 mmol, 0.001 equiv), and 2,2,2-trichloroethyl 2-diazo-2-(naphthalen-2-yl)acetate (68.7 mg, 0.2 mmol, 1.0 equiv) were used. The crude mixture was then dry-loaded on silica, followed by flash chromatography (gradient 10%-40% DCM in hexane) afforded **3ak** as a white solid (59.7 mg, 57% yield).

R_f (2H/1DCM) = 0.33

^{1}H NMR (400 MHz, $CDCl_3$) δ 7.68 – 7.60 (m, 2H), 7.54 (d, J = 1.9 Hz, 1H), 7.48 (d, J = 8.7 Hz, 1H), 7.38 – 7.31 (m, 2H), 7.28 (dd, J = 8.7, 1.9 Hz, 1H), 7.05 – 6.83 (m, 2H), 6.75 – 6.52 (m, 2H), 5.92 (d, J = 1.0 Hz, 2H), 5.24 (s, 2H), 4.70 (s, 2H), 3.09 – 2.91 (m, 6H), 2.73 – 2.53 (m, 2H).

^{13}C NMR (101 MHz, CDCl3) δ 173.1, 139.3, 136.9, 136.3, 134.1, 132.9, 132.5, 132.3, 130.0, 127.9, 127.3, 127.1, 126.5, 126.5, 126.1, 125.6, 125.5, 94.8, 74.5, 56.3, 37.4, 33.4.

HRMS (+p APCI) calcd. for [$C_{30}H_{26}O_2{}^{35}Cl_3$] ([M+H]$^+$) 523.0993 found 523.0997

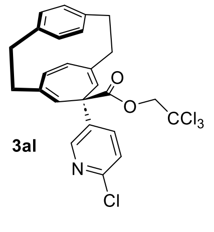

2,2,2-trichloroethyl (Z)-16-(6-chloropyridin-3-yl)-1(1,4)-cycloheptana-4(1,4)-benzenacyclohexaphane-11(17),12,14-triene-16-carboxylate (3al)

Prepared according to general procedure A. [2.2]Paracyclophane (125 mg, 0.6 mmol, 3.0 equiv), MS 4Å (100 wt%), $Rh_2(OBz)_4$ (1.4 mg, 0.0002 mmol, 0.001 equiv), and 2,2,2-trichloroethyl 2-(6-chloropyridin-3-yl)-2-diazoacetate (65.8 mg, 0.2 mmol, 1.0 equiv) were used. The crude mixture was then dry loaded on silica, followed by flash chromatography (gradient 0-10% ethyl acetate in hexane) afforded **3al** as a white crystalline solid (36.0 mg, 35% yield).

$\mathbf{R_f}$ (9H/1EA) = 0.33

^{1}**H NMR** (400 MHz, $CDCl_3$) δ 8.05 (d, *J* = 2.6 Hz, 1H), 7.35 (dd, *J* = 8.4, 2.6 Hz, 1H), 6.92 (d, *J* = 8.4 Hz, 1H), 6.89 (d, *J* = 1.5 Hz, 2H), 6.59 (s, 2H), 5.73 (s, 2H), 5.28 (s, 2H), 4.73 (s, 2H), 3.01 – 2.85 (m, 6H), 2.63 – 2.49 (m, 2H).

^{13}**C NMR** (101 MHz, $CDCl_3$) δ 172.0, 149.6, 149.3, 139.2, 138.2, 137.1, 134.4, 132.8, 130.0, 124.9, 122.3, 94.5, 74.5, 53.9, 37.2, 33.2.

HRMS (+p APCI) calcd. for [$C_{25}H_{22}O_2N^{35}Cl_4$] ($[M+H]^+$) 508.0399 found 508.0404

7. Rhodium catalyzed enantioselective cyclopropanation of 4,16-substituted [2.2]paracyclophane and characterization of product

General Procedure B: Desymmetrization of pseudo-para disubstituted [2.2]paracyclophane

To a flame-dried clean 16.0 ml vials equipped with a magnetic stir-bar was evacuated and back-filled with nitrogen 3 times. After cooling down to room temperature, $Rh_2(R\text{-}TPPTTL)_4$ (0.5 mol %) followed by 4,16-substituted [2.2]paracyclophane (0.2 mmol, 1.0 equiv), and MS 4Å (100 wt%) were then added. The vial was once again evacuated and purged with argon (3-5 times) and dried dichloromethane (2.0 mL) was added. The vial and its contents were then set to stir at 40 ºC by heating block under an nitrogen atmosphere. The diazo solution was prepared by adding diazo compound (0.3 mmol, 1.5 equiv) to the other 8 ml vial. The vial was evacuated and purged with argon (2-3 times) and of dry dichloromethane (2.0 mL) was then added to obtain a 0.15 M solution of the diazo compound. The 0.15 M solution was transferred into a plastic syringe (3 mL, 9.83 mm diameter). The 0.15 M solution of diazo was then added slowly to a catalyst and trap solution within 3 hours by using a well-calibrated syringe pump (0.667 mL/h). After complete addition (3 hours later), the residual diazo compound in the 3.0 mL plastic syringe was rinsed with 0.5 mL dry dichloromethane and transferred dropwise into the stirring reaction mixture of vial-A. The mixture was stirred for an additional 15 min and then concentrated under reduced pressure. The mixture was then stirred overnight before filtered through celite to remove dust from MS 4Å then dried load on silica and purified by flash chromatography to obtain the purified product.

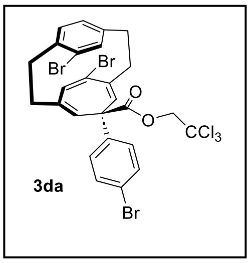

2,2,2-trichloroethyl (S,E)-12,43-dibromo-16-(4-bromophenyl)-1(1,4)-cycloheptana-4(1,4)-benzenacyclohexaphane-11(17),12,14-triene-16-carboxylate (3da)

Prepared according to general procedure B. 4,16-Dibromo[2.2]paracyclophane (1.00 g, 2.73 mmol, 1.0 equiv), MS 4Å (100 wt%), Rh_2(*S*-TPPTTL)$_4$ (33.7 mg, 0.0137 mmol, 0.0005 equiv), 2,2,2-trichloroethyl 2-(4-bromophenyl)-2-diazoacetate (1.53 g, 4.10 mmol, 1.5 equiv) were used. The crude mixture was then dry-loaded on silica, followed by flash chromatography (gradient 5%-30% DCM in hexane) afforded **3da** as an off-white solid (1.38 g, 71% yield, 95% ee). The product can be recrystallized in hot hexane to get 99% ee.

R$_f$ (3H/1DCM) = 0.26

^{1}H NMR (400 MHz, CDCl$_3$) δ 7.20 (dd, *J* = 7.7, 1.7 Hz, 1H), 7.15 (d, *J* = 8.6 Hz, 2H), 6.95 (d, *J* = 8.7 Hz, 2H), 6.87 (d, *J* = 7.7 Hz, 1H), 6.76 (d, *J* = 1.6 Hz, 1H), 6.38 (d, *J* = 1.0 Hz, 1H), 5.78 (s, 1H), 5.68 (s, 1H), 4.76 (d, *J* = 11.9 Hz, 1H), 4.68 (d, *J* = 11.9 Hz, 1H), 3.32 (ddd, *J* = 13.4, 9.9, 5.9 Hz, 1H), 3.27 – 3.16 (m, 1H), 3.09 – 2.91 (m, 3H), 2.86 – 2.74 (m, 2H), 2.34 (ddd, *J* = 13.8, 10.2, 5.9 Hz, 1H).

^{13}C NMR (101 MHz, CDCl$_3$) δ 171.6, 140.8, 138.5, 138.2, 137.8, 136.7, 135.9, 134.7, 131.0, 130.2, 129.3, 129.3, 127.9, 127.7, 127.0, 126.0, 121.3, 94.6, 74.5, 55.6, 37.0, 35.4, 32.3, 31.3.

HRMS (+p APCI) calcd for $C_{26}H_{21}O_2{}^{79}Br_3{}^{35}Cl_3$ $[M+H]^+$ 706.8152 found 706.8151

SFC: (ChiralCel OJ-3, 5% (50% methanol in isopropanol with 0.2% Formic Acid), 2.5 mL/min, 1.0 mg/ml, 10 min, λ = 230 nm) retention times of 4.25 (minor) and 5.56 min (major) 95% ee.

$[\alpha]^{20}_D$: -35.2° (c = 0.61 g/100 ml, $CHCl_3$, 95% ee)

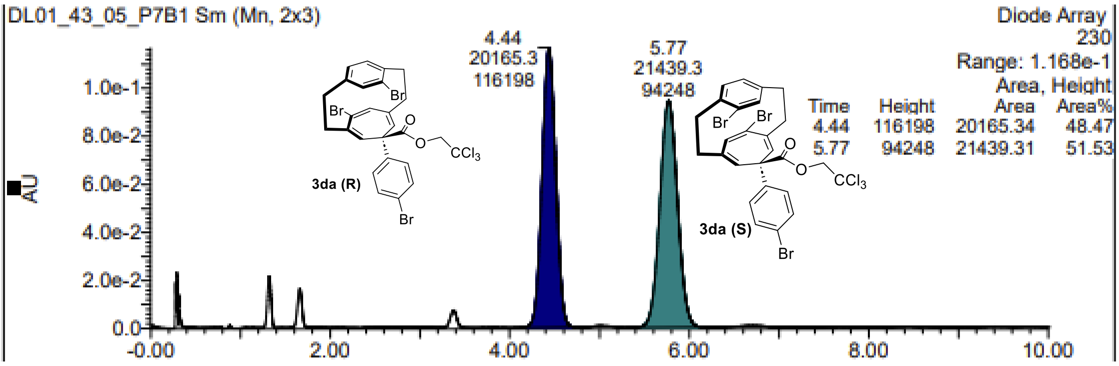

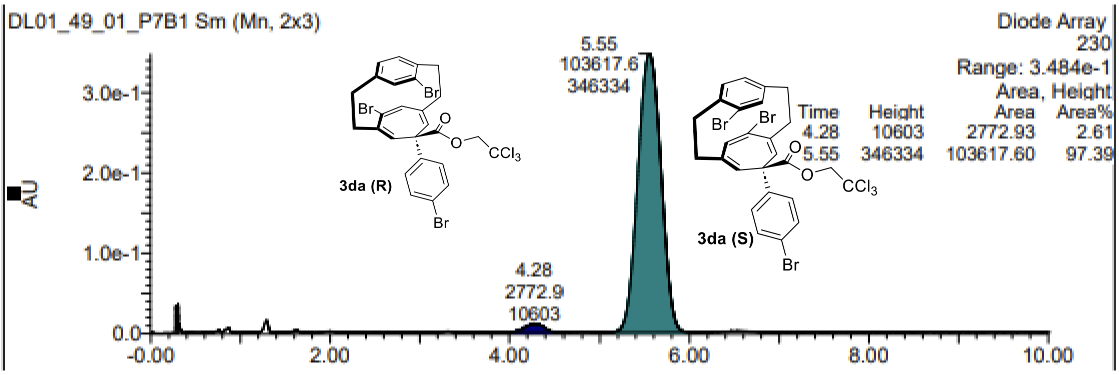

Figure S8. SFC trace for 3da (racemic – top /chiral – bottom)

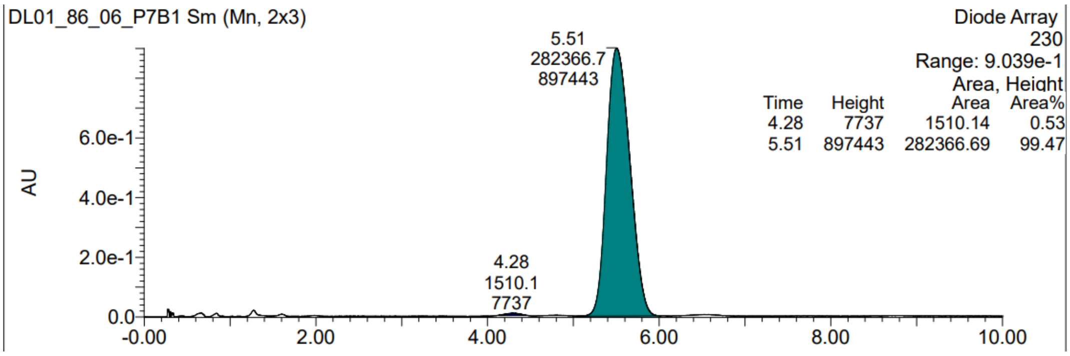

Figure S9. SFC trace for 3da SFC trace after recrystallization in hot hexane

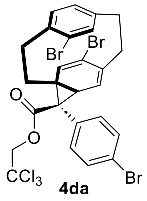

2,2,2-trichloroethyl (11S,17S,Z)-13,42-dibromo-17-(4-bromophenyl)-1(1,4)-bicyclo[4.1.0]heptana-4(1,4)-benzenacyclohexaphane-12,14-diene-17-carboxylate (4da) (*minor regio-isomer*)

Prepared according to general procedure B with modifying stochiometry. To an 16 ml vial was charged with 4,16-dibromo[2.2]paracyclophane (146.4 mg, 0.4 mmol, 2.0 equiv), MS 4Å (100 wt%), $Rh_2(OBz)_4$ (2.46 mg, 0.0001 mmol, 0.01 equiv), and 2,2,2-trichloroethyl 2-([1,1'-biphenyl]-4-yl)-2-diazoacetate (74.5 mg, 0.2 mmol, 1.0 equiv) were used. The crude mixture was then dry-loaded on silica, followed by flash chromatography (gradient 5%-30% DCM in hexane) afforded **3da** (14.0 mg, 20%) a white solid and **4da** (7.0 mg, 10%) as a clear oil which solidifies upon high-vacuum after a few days. **Note:** *The structure of minor-regioisomer was confirmed by COSEY experiment.*

R_f (3H/1DCM) = 0.22

^{1}H NMR (400 MHz, $CDCl_3$) δ 7.31 (d, *J* = 7.8 Hz, 1H), 7.22 (d, *J* = 8.1 Hz, 2H), 7.01 (s, 1H), 6.84 (d, *J* = 7.8 Hz, 1H), 6.81 (d, *J* = 8.1 Hz, 2H), **5.37** (s, 1H), **5.29** (d, *J* = 7.1 Hz, 1H), 4.84 (d, *J* = 11.9 Hz, 1H), 4.55 (d, *J* = 11.9 Hz, 1H), **3.42** (d, *J* = 7.0 Hz, 1H), 3.28 (dt, *J* = 14.5, 8.0 Hz, 1H), 3.08 – 2.87 (m, 3H), 2.70 (ddd, *J* = 13.8, 9.4, 2.0 Hz, 1H), 2.36 (ddd, *J* = 15.0, 8.3, 3.9 Hz, 1H), 2.26 – 2.15 (m, 2H). (*Highlighted signals showed correlation on COSEY)*

^{13}C NMR (101 MHz, CDCl$_3$) δ 171.0, 141.5, 138.3, 137.0, 134.9, 134.7, 134.2, 133.5, 132.5, 131.1, 130.5, 129.8, 125.7, 122.9, 121.2, 94.8, 74.8, 44.9, 38.6, 34.9, 33.2, 33.0, 33.0, 31.3.

HRMS (+p APCI) calcd for $C_{26}H_{21}O_2{}^{79}Br_3{}^{35}Cl_3$ $[M+H]^+$ 706.8152 found 706.8149

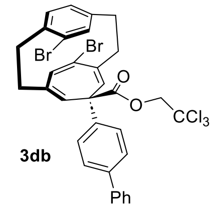

2,2,2-trichloroethyl (S,E)-16-([1,1'-biphenyl]-4-yl)-12,43-dibromo-1(1,4)-cycloheptana-4(1,4)-benzenacyclohexaphane-11(17),12,14-triene-16-carboxylate (3db)

Prepared according to general procedure B. 4,16-dibromo[2.2]paracyclophane (73.2 mg, 0.2 mmol, 1.0 equiv), MS 4Å (100 wt%), and Rh_2(*S*-TPPTTL)$_4$ (2.46 mg, 0.0001 mmol, 0.005 equiv), and 2,2,2-trichloroethyl 2-([1,1'-biphenyl]-4-yl)-2-diazoacetate (111.0 mg, 0.3 mmol, 1.5 equiv) were used. The crude mixture was then dry-loaded on silica, followed by flash chromatography (gradient 5%-30% DCM in hexane) afforded **3db** as a white solid (60.5 mg, 43% yield, 88% ee).

R_f (2H/1DCM) = 0.21

^{1}H NMR (400 MHz, CDCl$_3$) δ 7.51 – 7.45 (m, 2H), 7.40 – 7.34 (m, 2H), 7.31 – 7.25 (m, 3H), 7.21 (dd, J = 7.7, 1.7 Hz, 1H), 7.13 (d, J = 8.5 Hz, 2H), 6.87 (d, J = 7.7 Hz, 1H), 6.78 (d, J = 1.6 Hz, 1H), 6.44 (s, 1H), 5.83 (s, 1H), 5.70 (s, 1H), 4.78 (d, J = 11.9 Hz, 1H), 4.70 (d, J = 11.9 Hz, 1H), 3.34 (ddd, J = 13.3, 9.9, 6.0 Hz, 1H), 3.26 (ddd, J = 14.2, 8.1, 4.2 Hz, 1H), 3.12 – 2.90 (m, 3H), 2.89 – 2.76 (m, 2H), 2.37 (ddd, J = 13.8, 10.2, 5.9 Hz, 1H).

^{13}C NMR (101 MHz, CDCl$_3$) δ 172.2, 141.0, 140.7, 140.1, 138.6, 138.3, 137.8, 136.9, 135.8, 134.6, 131.1, 129.4, 128.8, 128.5, 128.2, 127.8, 127.5, 127.3, 127.0, 126.2, 125.9, 94.9, 74.6, 55.9, 37.2, 35.6, 32.4, 31.5.

HRMS (+p APCI) calcd. for $[C_{32}H_{26}O_2{}^{79}Br_2{}^{35}Cl_3]$ ($[M+H]^+$) 704.9360 found 704.9357

SFC: (ChiralCel OJ-3, 10% (50% methanol in isopropanol with 0.2% Formic Acid), 2.5 mL/min, 1.0 mg/ml, 15 min, λ = 230 nm) retention times of 6.59 (minor) and 9.42 min (major) 88% ee.

$[\alpha]^{20}_D$: -38.1° (c = 0.51 g/100 ml, $CHCl_3$, 88% ee)

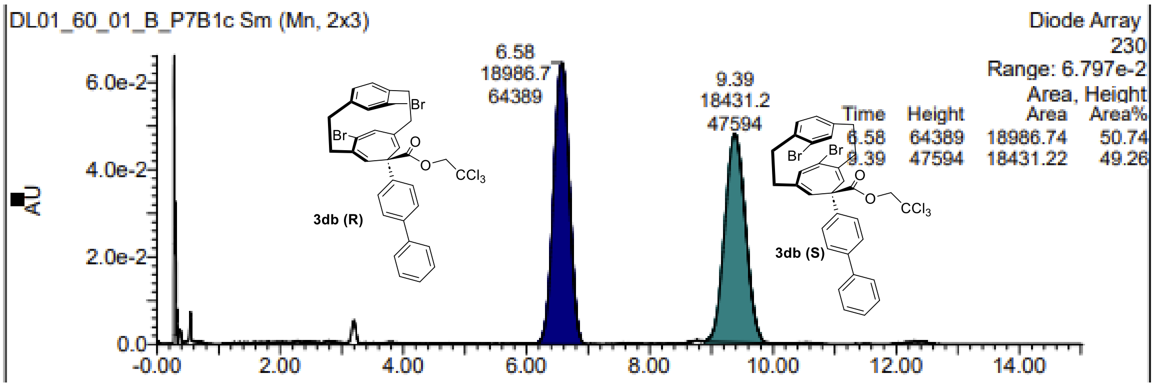

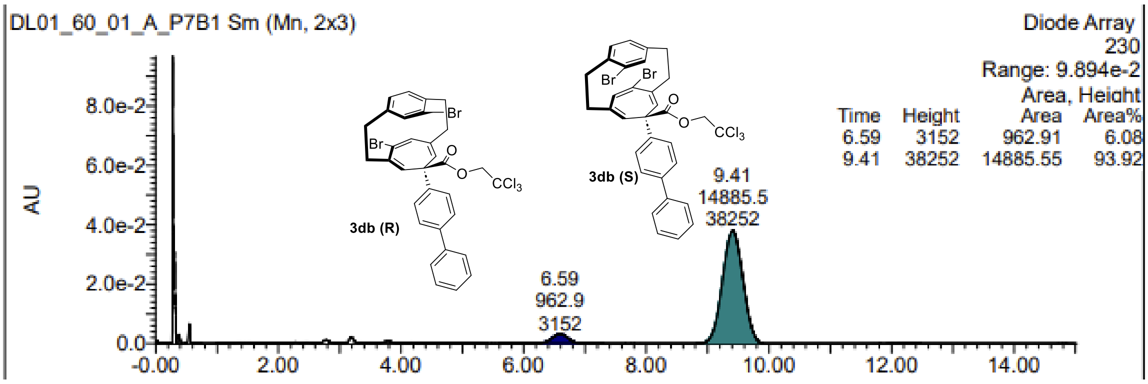

Figure S10. SFC trace for 3db (racemic – top /chiral – bottom)

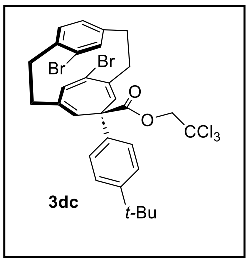

2,2,2-trichloroethyl (S,E)-12,43-dibromo-16-(4-(tert-butyl)phenyl)-1(1,4)-cycloheptana-4(1,4)-benzenacyclohexaphane-11(17),12,14-triene-16-carboxylate (3dc)

Prepared according to general procedure B. To an 16 ml vial was charged with 4,16-dibromo[2.2]paracyclophane (73.2 mg, 0.2 mmol, 1.0 equiv), MS 4Å (100 wt%), Rh_2(*S*-TPPTTL)$_4$ (2.46 mg, 0.0001 mmol, 0.005 equiv), and 2,2,2-trichloroethyl 2-(4-(tert-butyl)phenyl)-2-diazoacetate (105.0 mg, 0.3 mmol, 1.5 equiv) were used. The crude mixture was then dry-loaded on silica, followed by flash chromatography (gradient 5%-25% DCM in hexane) afforded **3dc** as a white solid (32.1 mg, 23% yield, 84% ee).

R$_f$ (2H/1DCM) = 0.37

^{1}H NMR (400 MHz, CDCl$_3$) δ 7.21 (d, *J* = 7.7 Hz, 1H), 7.01 (d, *J* = 8.7 Hz, 2H), 6.96 (d, *J* = 8.6 Hz, 2H), 6.86 (d, *J* = 7.6 Hz, 1H), 6.77 (s, 1H), 6.40 (s, 1H), 5.80 (s, 1H), 5.68 (s, 1H), 4.76 (d, *J* = 11.9 Hz, 1H), 4.68 (d, *J* = 11.9 Hz, 1H), 3.32 (ddd, *J* = 13.3, 9.8, 6.0 Hz, 1H), 3.27 – 3.17 (m, 1H), 3.09 – 2.91 (m, 3H), 2.86 – 2.74 (m, 2H), 2.34 (ddd, *J* = 13.8, 10.1, 6.0 Hz, 1H), 1.18 (s, 9H).

^{13}C NMR (101 MHz, CDCl$_3$) δ 172.3, 150.1, 140.9, 138.5, 138.1, 136.8, 135.4, 135.3, 134.2, 131.0, 129.3, 128.5, 127.7, 127.5, 127.2, 126.0, 123.9, 94.8, 74.3, 55.7, 37.0, 35.5, 34.3, 32.3, 31.4, 31.2.

HRMS (+p APCI) calcd. for [$C_{30}H_{30}O_2{}^{79}Br_2{}^{35}Cl_3$] ([M+H]$^+$) 684.9673 found 684.9667

SFC: (ChiralCel OJ-3, 5% (50% methanol in isopropanol with 0.2% Formic Acid), 2.5 mL/min, 1.0 mg/ml, 5 min, λ = 230 nm) retention times of 1.92 (minor) and 2.41 min (major) 84% ee.

$[\alpha]^{20}_D$: -27.7° (c = 0.40 g/100 ml, $CHCl_3$, 84% ee)

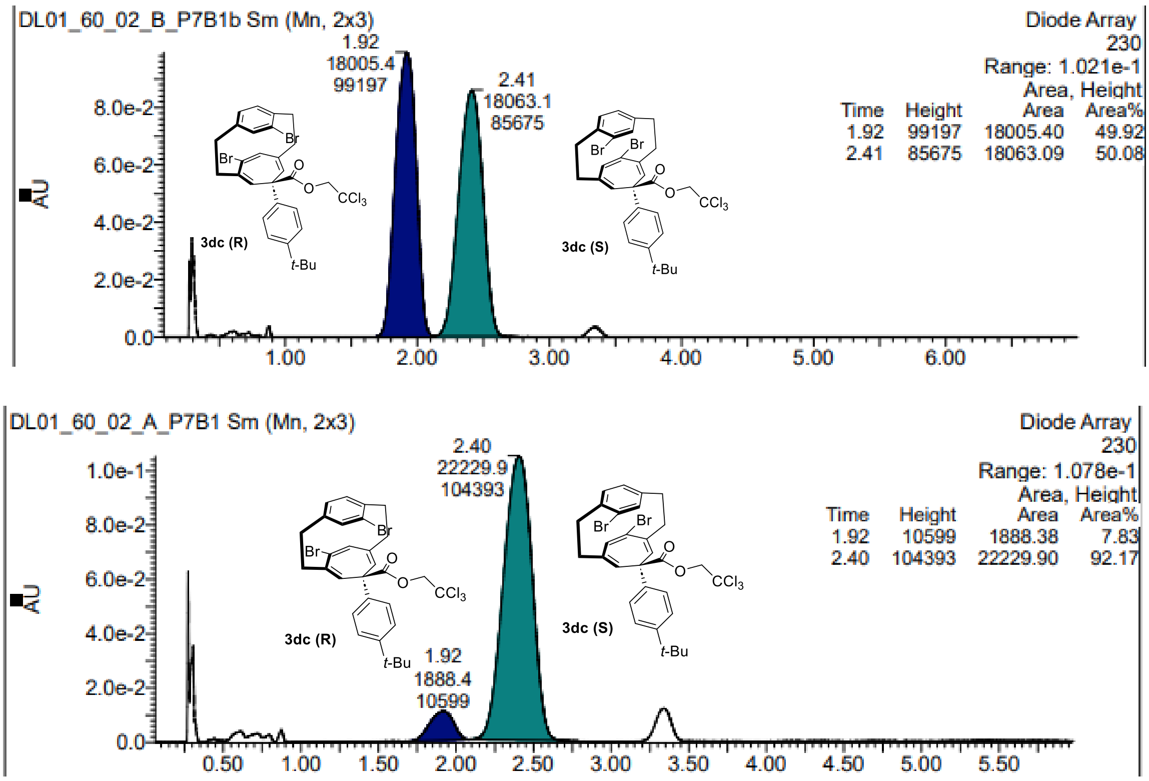

Figure S11. SFC trace for 3dc (racemic – top /chiral – bottom)

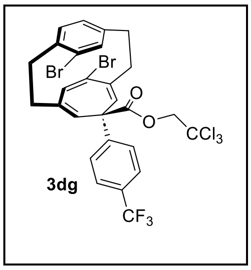

2,2,2-trichloroethyl (S,E)-12,43-dibromo-16-(4-(trifluoromethyl)phenyl)-1(1,4)-cycloheptana-4(1,4)-benzenacyclohexaphane-11(17),12,14-triene-16-carboxylate (3dg)

Prepared according to general procedure B. 4,16-dibromo[2.2]paracyclophane (73.2 mg, 0.2 mmol, 1.0 equiv), MS 4Å (100 wt%), $Rh_2(R\text{-}TPPTTL)_4$ (2.46 mg, 0.0001 mmol, 0.005 equiv), and 2,2,2-trichloroethyl 2-(4-(tert-butyl)phenyl)-2-diazoacetate (105.0 mg, 0.3 mmol, 1.5 equiv) were used. The crude mixture was then dry-loaded on silica, followed by flash chromatography (gradient 5%-25% DCM in hexane) afforded **3dg** as a white solid (77.9 mg, 56% yield, -94% ee).

R_f (3H/1DCM) = 0.36

^{1}H NMR (600 MHz, $CDCl_3$) δ 7.29 (d, J = 8.3 Hz, 2H), 7.21 (dd, J = 7.7, 1.7 Hz, 3H), 6.87 (d, J = 7.6 Hz, 1H), 6.77 (d, J = 2.0 Hz, 1H), 6.41 (s, 1H), 5.81 (s, 1H), 5.68 (s, 1H), 4.76 (d, J = 11.9 Hz, 1H), 4.69 (d, J = 11.9 Hz, 1H), 3.34 (ddd, J = 13.4, 9.9, 5.9 Hz, 1H), 3.24 (ddd, J = 14.4, 8.7, 3.9 Hz, 1H), 3.10 – 2.95 (m, 3H), 2.87 – 2.77 (m, 2H), 2.36 (ddd, J = 13.8, 10.2, 5.9 Hz, 1H).

^{13}C NMR (151 MHz, CDCl$_3$) δ 171.4, 142.9, 140.8, 138.5, 138.3, 136.7, 136.2, 135.0, 131.0, 129.5 (q, J = 32.4 Hz), 129.3, 127.9, 127.8, 127.7, 126.8, 126.0, 124.03 (q, J = 3.8 Hz), 124.01 (q, J = 272.1 Hz), 94.5, 74.5, 56.0, 37.0, 35.4, 32.3, 31.3.

^{19}F NMR (376 MHz, CDCl$_3$) δ -62.53.

HRMS (+p APCI) cal $C_{27}H_{21}O_2{}^{79}Br_2{}^{35}Cl_3F_3$ [M+H]$^+$ 696.8921 found 696.8922

SFC: (CEL 1, 5% (50% methanol in isopropanol with 0.2% Formic Acid), 2.5 mL/min, 1.0 mg/ml, 10 min, λ = 230 nm) retention times of 5.00 (major) and 5.69 min (minor) -94% ee.

[α]$^{20}_{D}$: +28.7° (c = 0.35 g/100 ml, CHCl$_3$, -94% ee)

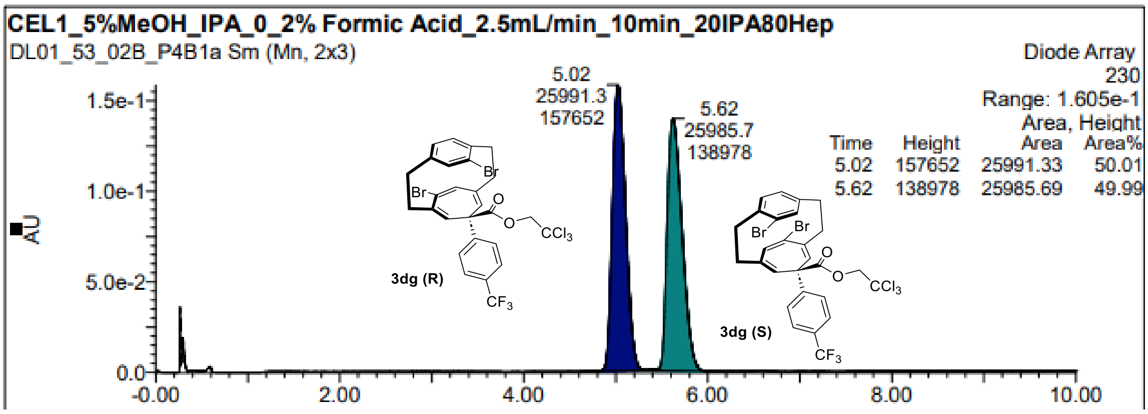

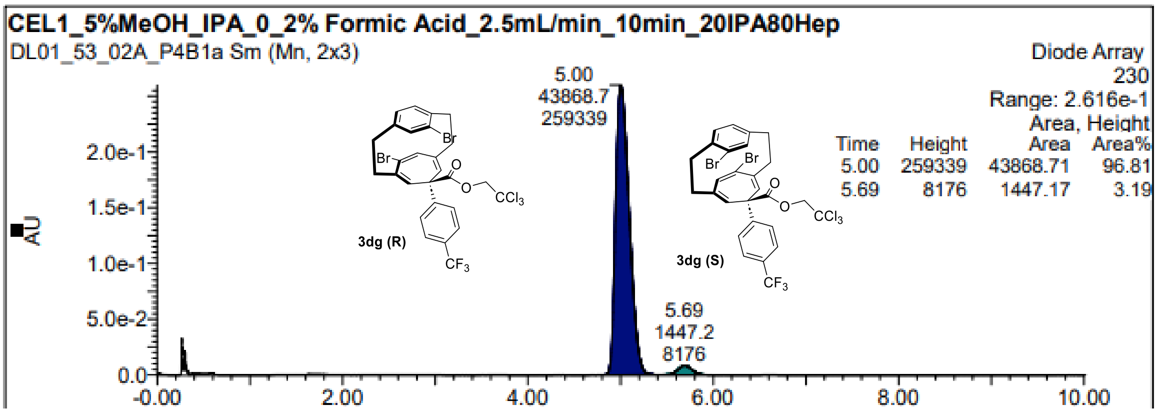

Figure S12. SFC trace for 3dg (racemic – top /chiral – bottom)

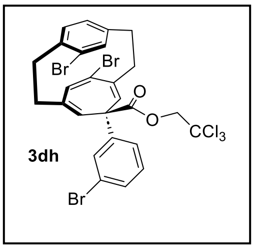

2,2,2-trichloroethyl (S,E)-12,43-dibromo-16-(3-bromophenyl)-1(1,4)-cycloheptana-4(1,4)-benzenacyclohexaphane-11(17),12,14-triene-16-carboxylate (3dh)

Prepared according to general procedure B. 4,16-dibromo[2.2]paracyclophane (73.2 mg, 0.2 mmol, 1.0 equiv), MS 4Å (100 wt%), and Rh$_2$(S-TPPTTL)$_4$ (2.46 mg, 0.0001 mmol, 0.005 equiv), 2,2,2-trichloroethyl 2-(3-bromophenyl)-2-diazoacetate (112.0

mg, 0.3 mmol, 1.5 equiv) were used. The crude mixture was then dry-loaded on silica, followed by flash chromatography (gradient 5%-25% DCM in hexane) afforded **3dh** as a white solid (95.6 mg, 67% yield, 95% ee).

R_f (3H/1DCM) = 0.32

^{1}H NMR (400 MHz, CDCl$_3$) δ δ 7.25 – 7.16 (m, 3H), 7.00 (dt, *J* = 7.9, 1.4 Hz, 1H), 6.93 – 6.84 (m, 2H), 6.76 (d, *J* = 1.6 Hz, 1H), 6.36 (s, 1H), 5.76 (s, 1H), 5.71 (s, 1H), 4.76 (d, *J* = 11.9 Hz, 1H), 4.71 (d, *J* = 11.9 Hz, 1H), 3.39 – 3.19 (m, 2H), 3.10 – 2.91 (m, 3H), 2.87 – 2.75 (m, 2H), 2.36 (ddd, *J* = 13.8, 10.2, 5.9 Hz, 1H).

^{13}C NMR (101 MHz, CDCl$_3$) δ (101 MHz, CDCl$_3$) δ 171.6, 141.0, 140.9, 138.5, 138.2, 136.7, 136.1, 134.9, 131.0, 130.8, 130.5, 129.3, 128.5, 127.8, 127.8, 126.7, 126.3, 126.0, 121.4, 94.6, 74.5, 55.8, 37.0, 35.5, 32.3, 31.3.

HRMS (+p APCI) calcd. for [$C_{26}H_{21}O_2{}^{79}Br_3{}^{35}Cl_3$] ([M+H]$^+$) 706.8152 found 706.8153

SFC: (ChiralCel OJ-3, 5% (50% methanol in isopropanol with 0.2% Formic Acid), 2.5 mL/min, 1.0 mg/ml, 10 min, λ = 230 nm) retention times of 4.11 (minor) and 6.80 min (major) 95% ee.

[α]$^{20}_D$: -25.7° (c = 0.31 g/100 ml, CHCl$_3$, 95% ee)

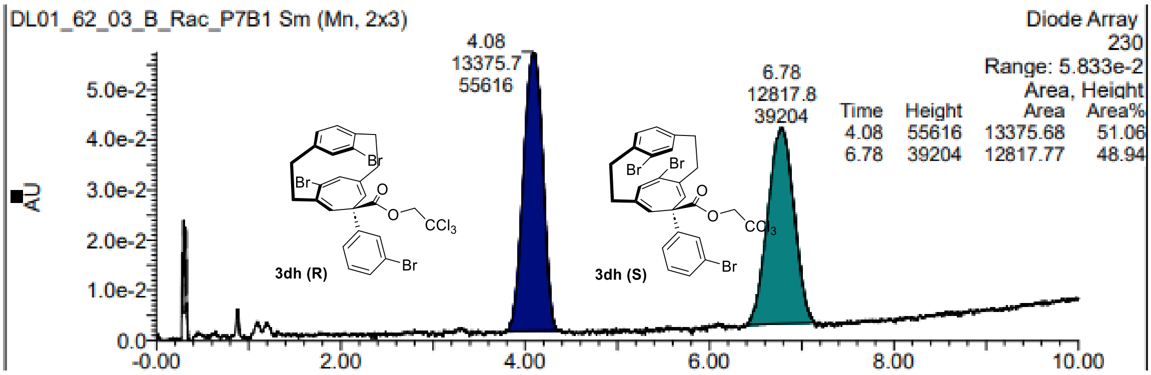

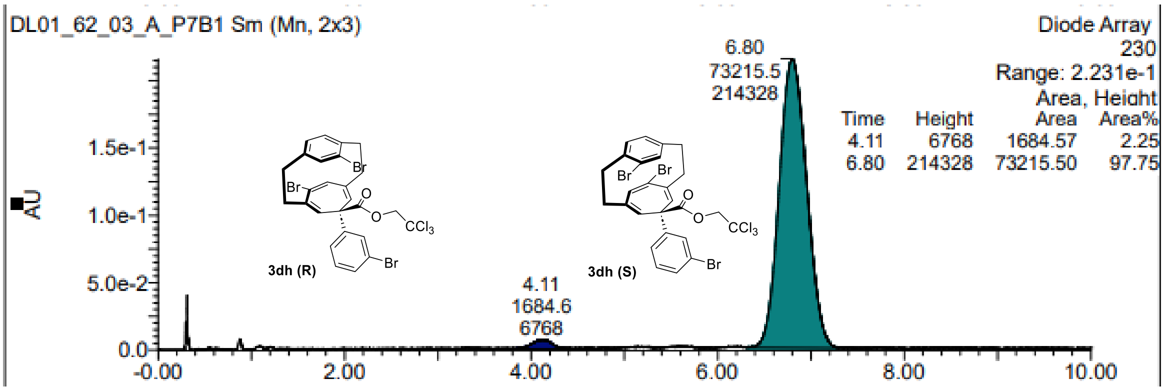

Figure S13. SFC trace for 3dh (racemic – top /chiral – bottom)

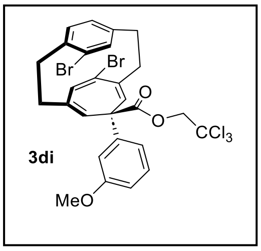

2,2,2-trichloroethyl (S,E)-12,43-dibromo-16-(3-methoxyphenyl)-1(1,4)-cycloheptana-4(1,4)-benzenacyclohexaphane-11(17),12,14-triene-16-carboxylate (3di)

Prepared according to general procedure B. 4,16-dibromo[2.2]paracyclophane (73.2 mg, 0.2 mmol, 1.0 equiv), MS 4Å (100 wt%), Rh_2(*S*-TPPTTL)$_4$ (2.46 mg, 0.0001 mmol, 0.005 equiv), and 2,2,2-trichloroethyl 2-(3-methoxyphenyl)-2-diazoacetate (97.1 mg, 0.3 mmol, 1.5 equiv) were used. The crude mixture was then dry-loaded on silica, followed by flash chromatography (gradient 5%-25% DCM in hexane) afforded **3di** as a white solid (68.1 mg, 52% yield, 96% ee).

R_f (3H/1DCM) = 0.25

^{1}H NMR (400 MHz, $CDCl_3$) δ 7.21 (dd, J = 7.7, 1.8 Hz, 1H), 6.94 (t, J = 7.9 Hz, 1H), 6.87 (d, J = 7.7 Hz, 1H), 6.77 (d, J = 1.8 Hz, 1H), 6.71 – 6.56 (m, 3H), 6.40 (s, 1H), 5.79 (s, 1H), 5.69 (s, 1H), 4.77 (d, J = 11.9 Hz, 1H), 4.69 (d, J = 11.9 Hz, 1H), 3.67 (s, 3H), 3.38 – 3.18 (m, 2H), 3.08 – 2.92 (m, 3H), 2.87 – 2.74 (m, 2H), 2.34 (ddd, J = 13.7, 10.2, 6.0 Hz, 1H).

^{13}C NMR (101 MHz, $CDCl_3$) δ 172.0, 158.4, 140.9, 140.2, 138.5, 138.1, 136.7, 135.7, 134.4, 131.0, 129.3, 128.3, 127.9, 127.7, 127.2, 126.0, 120.5, 113.9, 112.7, 94.8, 74.4, 56.0, 55.2, 37.0, 35.5, 32.3, 31.3.

HRMS (+p APCI) calcd. for [$C_{27}H_{24}O_3{}^{79}Br_2{}^{35}Cl_3$] ([M+H]$^+$) 658.9152 found 658.9152

SFC: (ChiralCel OJ-3, 5% (50% methanol in isopropanol with 0.2% Formic Acid), 2.5 mL/min, 1.0 mg/ml, 10 min, λ = 230 nm) retention times of 3.30 (minor) and 5.92 min (major) 96% ee.

$[\alpha]^{20}_D$: -27.3° (c = 0.24 g/100 ml, $CHCl_3$, 96% ee)

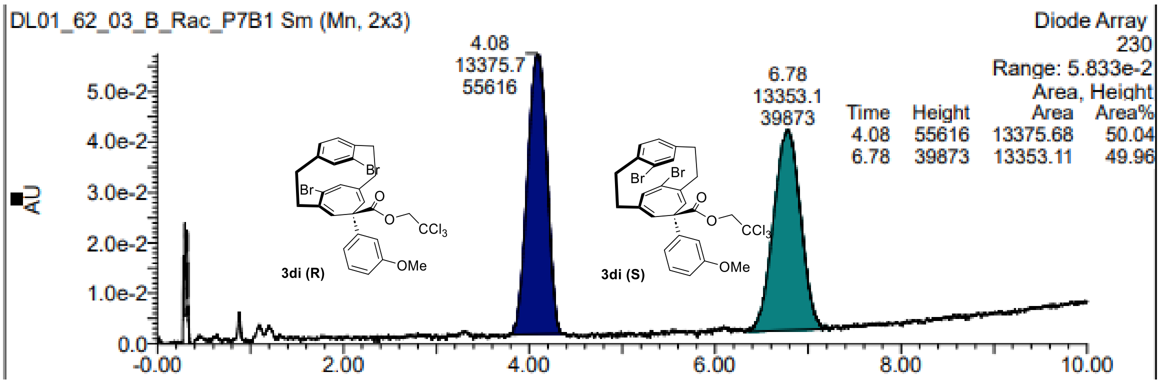

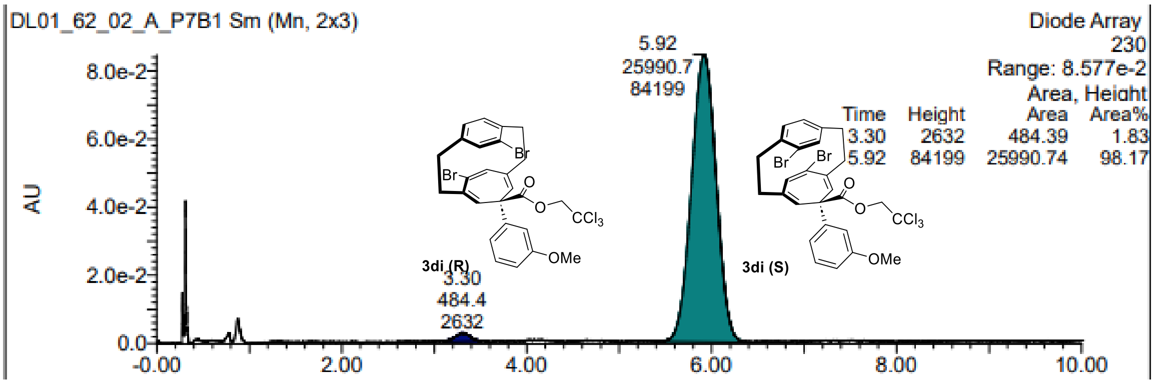

Figure S14. SFC trace for 3di (racemic – top /chiral – bottom)

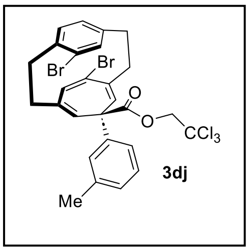

2,2,2-trichloroethyl (S,E)-12,43-dibromo-16-(m-tolyl)-1(1,4)-cycloheptana-4(1,4)-benzenacyclohexaphane-11(17),12,14-triene-16-carboxylate (3dj)

Prepared according to general procedure B. 4,16-dibromo[2.2]paracyclophane (73.2 mg, 0.2 mmol, 1.0 equiv), MS 4Å (100 wt%), and Rh_2(*S*-TPPTTL)$_4$ (2.46 mg, 0.0001 mmol, 0.005 equiv), and 2,2,2-trichloroethyl 2-diazo-2-(m-tolyl)acetate (92.3 mg, 0.3 mmol, 1.5 equiv) were used. The crude mixture was then dry-loaded on silica, followed by flash chromatography (gradient 5%-20% DCM in hexane) afforded **3dj** as a white solid (95.0 mg, 74% yield, 95% ee).

R_f (3H/1DCM) = 0.25

^{1}H NMR (800 MHz, $CDCl_3$) δ 7.20 (dt, *J* = 7.6, 1.4 Hz, 1H), 6.92 – 6.89 (m, 1H), 6.86 (dd, *J* = 8.1, 3.8 Hz, 4H), 6.77 (d, *J* = 1.7 Hz, 1H), 6.42 (s, 1H), 5.78 (s, 1H), 5.66 (s, 1H), 4.75 (dd, *J* = 11.9, 1.1 Hz, 1H), 4.69 (dd, *J* = 11.8, 1.1 Hz, 1H), 3.35 – 3.29 (m, 1H), 3.27 – 3.22 (m, 1H), 3.07 – 2.95 (m, 3H), 2.84 – 2.78 (m, 2H), 2.34 (ddd, *J* = 13.9, 10.2, 6.0 Hz, 1H), 2.17 (s, 3H).

^{13}C NMR (201 MHz, $CDCl_3$) δ 172.4, 141.0, 138.6, 138.5, 138.1, 136.9, 136.6, 135.6, 134.3, 131.1, 129.4, 128.8, 128.6, 128.2, 127.8, 127.5, 127.0, 126.2, 125.0, 94.9, 74.5, 56.1, 37.1, 35.6, 32.5, 31.5, 21.5.

HRMS (+p APCI) calcd. for [$C_{27}H_{24}O_2{}^{79}Br_2{}^{35}Cl_3$] ($[M+H]^+$) 642.9203 found 642.9200

HPLC (Chiralpak ADH column, 1% i-propanol in hexane, 1.0 mLmin^{-1}, 1.0 mgmL^{-1}, 15 min, UV 230 nm) retention times of 5.19 min (minor) and 6.28 min (major), 95% ee.

$[\alpha]^{20}_D$: -29.2° (c = 1.26 g/100 ml, $CHCl_3$, 95% ee)

```
Acq. Method      : ADH_60min_1.0ML_1.0%.M
Analysis Method : C:\Chem32\1\Data\02-Dec-2023\02-Dec-2023 2023-12-05 20-30-48\ADH_flush.M (
                  Sequence Method)
Last changed     : 6/26/2017 5:04:52 PM by SYSTEM
Additional Info : Peak(s) manually integrated
```

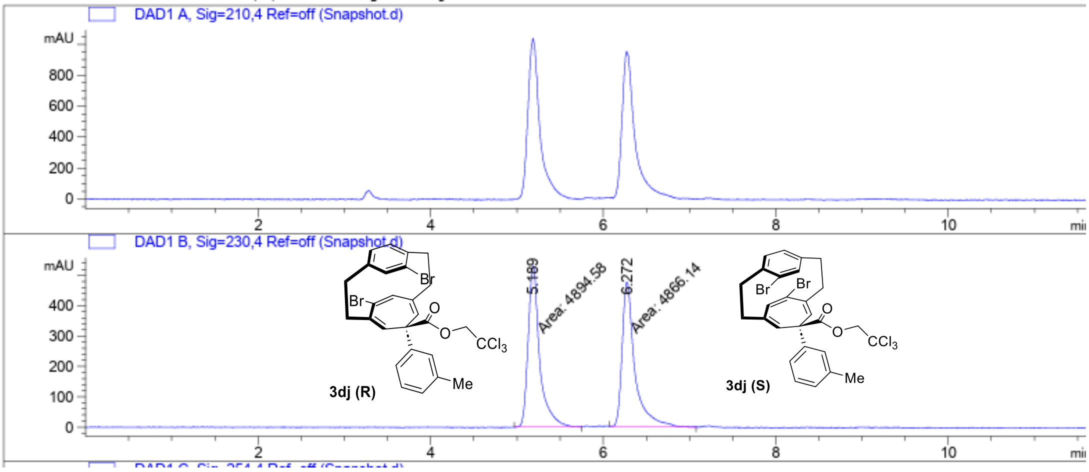

Signal 2: DAD1 B, Sig=230,4 Ref=off

Peak #	RetTime [min]	Type	Width [min]	Area [mAU*s]	Height [mAU]	Area %
1	5.189	MM	0.1539	4894.58496	530.12305	50.1457
2	6.272	MM	0.1703	4866.14453	476.16986	49.8543

Totals : 9760.72949 1006.29291

```
                                          Inj Volume : 10.000 µl
Method           : C:\Chem32\1\Data\02-Dec-2023\02-Dec-2023 2023-12-05 20-30-48\ADH_15min_1.0ML_1
                   .0%.M (Sequence Method)
Last changed     : 11/18/2022 12:52:25 PM by SYSTEM
Additional Info : Peak(s) manually integrated
```

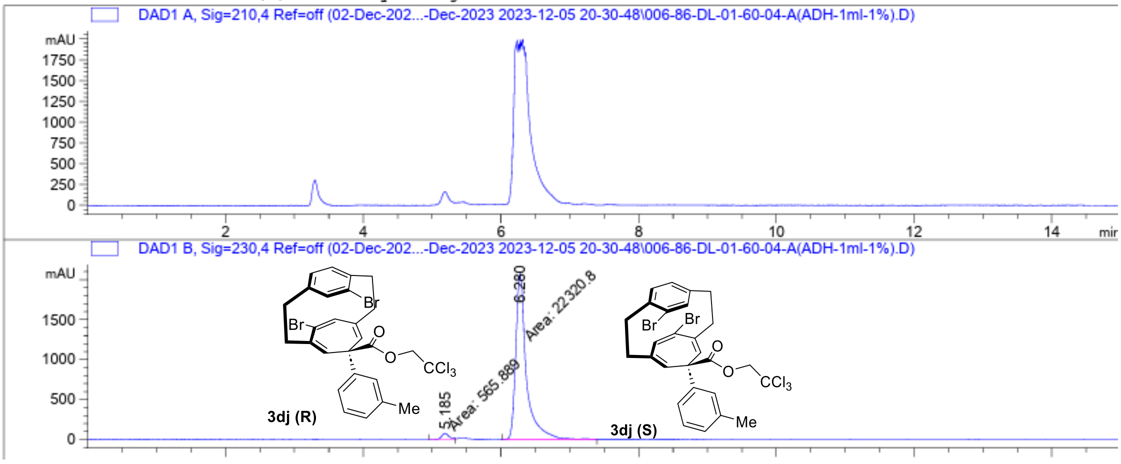

Signal 2: DAD1 B, Sig=230,4 Ref=off

Peak #	RetTime [min]	Type	Width [min]	Area [mAU*s]	Height [mAU]	Area %
1	5.185	MF	0.1257	565.88892	75.05724	2.4726
2	6.280	MM	0.1789	2.23208e4	2079.55103	97.5274

Totals : 2.28867e4 2154.60826

Figure S15. SFC trace for 3dj(racemic – top /chiral – bottom)

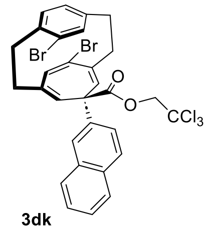

3dk

2,2,2-trichloroethyl (S,E)-12,43-dibromo-16-(naphthalen-2-yl)-1(1,4)-cycloheptana-4(1,4)-benzenacyclohexaphane-11(17),12,14-triene-16-carboxylate (3dk)

Prepared according to general procedure B. 4,16-dibromo[2.2]paracyclophane (73.2 mg, 0.2 mmol, 1.0 equiv), MS 4Å (100 wt%), Rh_2(*S*-TPPTTL)$_4$ (2.46 mg, 0.0001 mmol, 0.005 equiv), and 2,2,2-trichloroethyl 2-diazo-2-(naphthalen-2-yl)acetate (103.0 mg, 0.3 mmol, 1.5 equiv) were used. The crude mixture was then dry-loaded on silica, followed by flash chromatography (gradient 5%-25% DCM in hexane) afforded **3dk** as a white solid (37.5 mg, 28% yield, 82% ee).

R_f (2H/1DCM) = 0.34

^{1}H NMR (400 MHz, CDCl$_3$) δ 7.71 – 7.61 (m, 2H), 7.52 (dd, *J* = 5.3, 3.4 Hz, 2H), 7.40 – 7.33 (m, 2H), 7.21 (ddd, *J* = 8.0, 6.8, 1.8 Hz, 2H), 6.86 (d, *J* = 7.7 Hz, 1H), 6.81 (d, *J* = 1.6 Hz, 1H), 6.53 (s, 1H), 5.89 (s, 1H), 5.63 (s, 1H), 4.73 (d, *J* = 11.9 Hz, 1H), 4.67 (d, *J* = 11.9 Hz, 1H), 3.40 – 3.25 (m, 2H), 3.10 (ddd, *J* = 13.6, 9.8, 3.5 Hz, 1H), 3.06 – 2.95 (m, 2H), 2.93 – 2.76 (m, 2H), 2.36 (ddd, *J* = 13.7, 10.2, 5.9 Hz, 1H).

^{13}C NMR (101 MHz, CDCl$_3$) δ 172.1, 140.9, 138.5, 138.0, 136.8, 135.9, 135.8, 134.6, 132.7, 132.4, 131.0, 129.3, 128.6, 127.9, 127.8, 127.4, 127.3, 126.8, 126.7, 126.1, 125.8, 94.7, 74.5, 56.1, 37.1, 35.5, 32.3, 31.3.*(missing 2 carbons)*

HRMS (+p APCI) calcd. for [$C_{30}H_{24}O_2{}^{79}Br_2{}^{35}Cl_3$] ([M+H]$^+$) 678.9203 found 678.9199

SFC: (ChiralCel OJ-3, 5% (50% methanol in isopropanol with 0.2% Formic Acid), 2.5 mL/min, 1.0 mg/ml, 25 min, λ = 230 nm) retention times of 8.30 (minor) and 15.53 min (major) 82% ee.

[α]$^{20}_D$: -9.31° (c = 0.26 g/100 ml, CHCl$_3$, 82% ee)

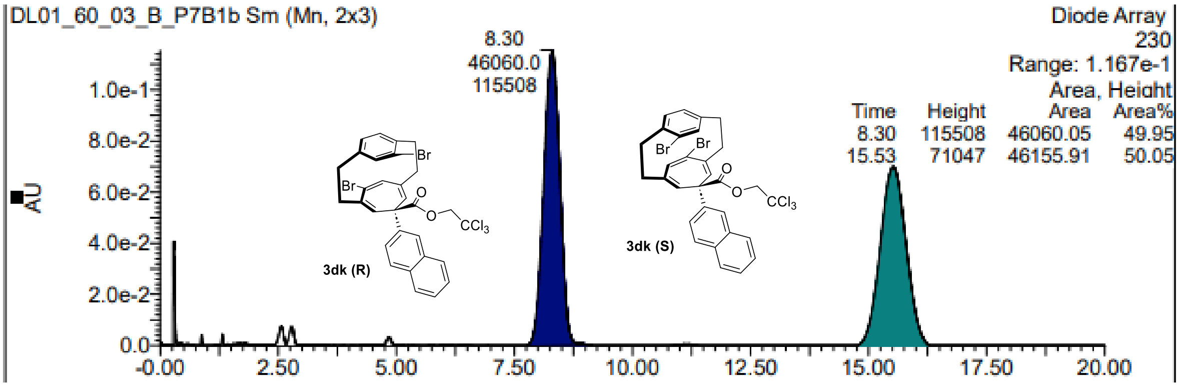

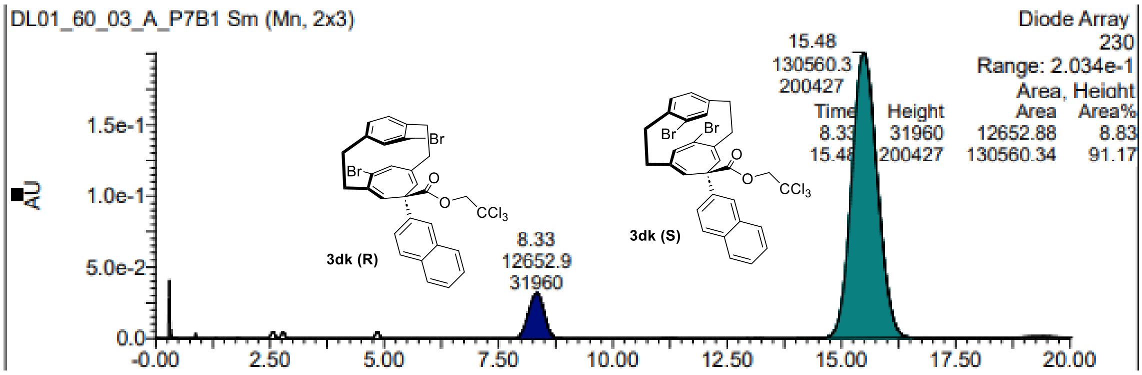

Figure S16. SFC trace for 3dk (racemic – top /chiral – bottom)

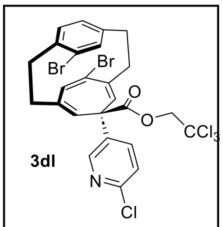

2,2,2-trichloroethyl (S,E)-12,43-dibromo-16-(6-chloropyridin-3-yl)-1(1,4)-cycloheptana-4(1,4)-benzenacyclohexaphane-11(17),12,14-triene-16-carboxylate (3dl)

Prepared according to general procedure B. 4,16-dibromo[2.2]paracyclophane (73.2 mg, 0.2 mmol, 1.0 equiv), MS 4Å (100 wt%), Rh_2(*S*-TPPTTL)$_4$ (2.46 mg, 0.0001 mmol, 0.005 equiv), and 2,2,2-trichloroethyl 2-(6-chloropyridin-3-yl)-2-diazoacetate (98.7 mg, 0.3 mmol, 1.5 equiv) were used. The crude mixture was then dry-loaded on silica, followed by flash chromatography (gradient 5%-20% DCM in hexane) afforded **3dl** as a white solid (90.8 mg, 68% yield, 97% ee). ***Note:*** *Racemic sample was prepared using Rh_2(R/S-TPPTTL)$_4$ as an achiral catalyst $Rh_2(OBz)_4$ resulted in trace amount of product.*

R_f (1H/1DCM) = 0.40

^{1}H NMR (800 MHz, CDCl$_3$) δ 8.09 (d, *J* = 2.3 Hz, 1H), 7.35 (dd, *J* = 8.5, 2.7 Hz, 1H), 7.20 (d, *J* = 7.6 Hz, 1H), 7.00 (d, *J* = 8.4 Hz, 1H), 6.87 (d, *J* = 7.6 Hz, 1H), 6.76 (s, 1H), 6.35 (s, 1H), 5.76 (s, 1H), 5.73 (s, 1H), 4.78 (d, *J* = 11.9 Hz, 1H), 4.71 (d, *J* = 11.8 Hz, 1H), 3.33 (ddd, *J* = 13.3, 10.0, 5.9 Hz, 1H), 3.23 (ddd, *J* = 13.1, 9.1, 3.6 Hz, 1H), 3.04 (ddd, *J* = 13.8, 10.0, 3.6 Hz, 1H), 3.02 – 2.95 (m, 2H), 2.85 – 2.78 (m, 2H), 2.36 (ddd, *J* = 13.9, 10.3, 5.9 Hz, 1H).

^{13}C NMR (201 MHz, CDCl$_3$) δ 171.08, 150.42, 149.01, 140.91, 138.58, 138.55, 137.81, 136.84, 136.83, 135.63, 133.95, 131.12, 129.47, 127.96, 126.80, 126.08, 125.88, 122.78, 94.53, 74.69, 53.94, 37.16, 35.52, 32.32, 31.30.

HRMS (+p APCI) calcd. for [$C_{25}H_{20}O_2N^{79}Br_2{}^{35}Cl_4$] ([M+H]$^+$) 663.8609 found 663.8612

HPLC (Chiralpak ADH column, 1% i-propanol in hexane, 1.0 mLmin^{-1}, 1.0 mgmL^{-1}, 15 min, UV 230 nm) retention times of 21.61 min (minor) and 28.69 min (major), 97% ee.

$[\alpha]^{20}_D$: -31.9° (c = 0.96 g/100 ml, CHCl$_3$, 97% ee)

```
Method          : C:\Chem32\1\Data\10-Dec-2023\10-Dec-2023 2023-12-10 17-51-15\ADH_60min_1.0ML_1
                  .0%.M (Sequence Method)
Last changed    : 6/29/2017 2:30:56 PM by SYSTEM
Additional Info : Peak(s) manually integrated
```

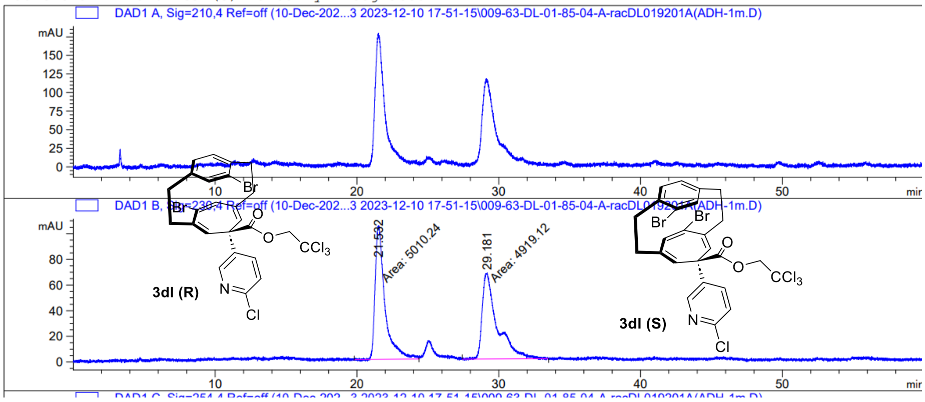


```
Signal 2: DAD1 B, Sig=230,4 Ref=off

Peak RetTime Type  Width     Area      Height     Area
  #   [min]        [min]   [mAU*s]     [mAU]       %
----|-------|----|-------|----------|----------|--------|
   1  21.532 MM    0.8030 5010.23633  103.98847  50.4588
   2  29.181 MM    1.2243 4919.12305   66.96335  49.5412

Totals :                  9929.35938  170.95182
```

```
Method          : C:\Chem32\1\Data\10-Dec-2023\10-Dec-2023 2023-12-11 07-53-06\ADH_60min_1.0ML_1
                  .0%.M (Sequence Method)
Last changed    : 6/29/2017 2:30:56 PM by SYSTEM
Additional Info : Peak(s) manually integrated
```

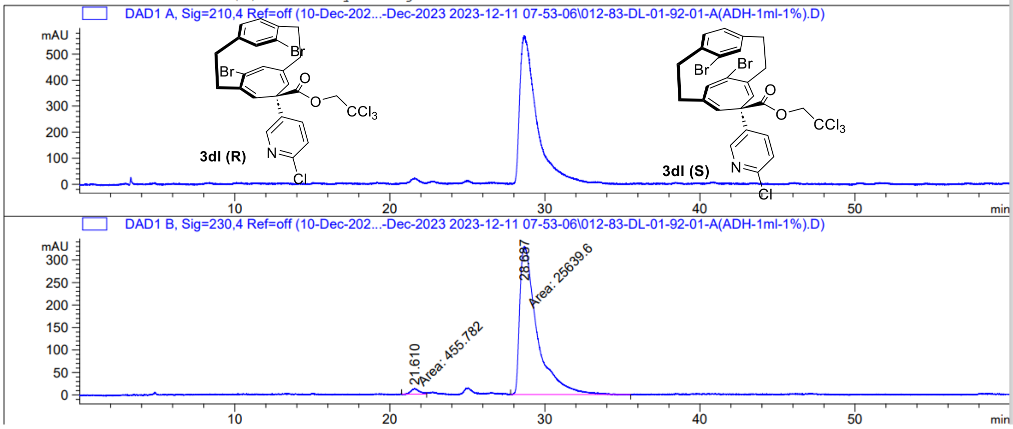


```
Signal 2: DAD1 B, Sig=230,4 Ref=off

Peak RetTime Type  Width     Area      Height     Area
  #   [min]        [min]   [mAU*s]     [mAU]       %
----|-------|----|-------|----------|----------|--------|
   1  21.610 MM    0.6082  455.78214   12.48947   1.7466
   2  28.687 MM    1.2973 2.56396e4  329.39255  98.2534

Totals :                  2.60954e4  341.88202
```

Figure S17. SFC trace for 3dl (racemic – top /chiral – bottom)

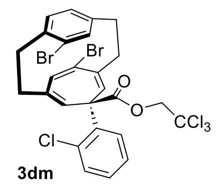
3dm

2,2,2-trichloroethyl (S,E)-12,43-dibromo-16-(2-chlorophenyl)-1(1,4)-cycloheptana-4(1,4)-benzenacyclohexaphane-11(17),12,14-triene-16-carboxylate (3dm)

Prepared according to general procedure B. 4,16-dibromo[2.2]paracyclophane (73.2 mg, 0.2 mmol, 1.0 equiv), MS 4Å (100 wt%), Rh_2(*S*-TPPTTL)$_4$ (2.46 mg, 0.0001 mmol, 0.005 equiv), and 2,2,2-trichloroethyl 2-(2-chlorophenyl)-2-diazoacetate (98.4 mg, 0.3 mmol, 1.5 equiv) were used. The crude mixture was then dry-loaded on silica, followed by flash chromatography (gradient 5%-20% DCM in hexane) afforded **3dm** as a clear viscous liquid which will slowly solidify into white solid after several days (99.9 mg, 70% yield, 95% ee). **Note:** *NMR showed hinder rotation – analyzed as a mixture of two rotamer – efforts to deconvolute ^{1}H-NMR at higher temperatures were unsuccessful. Racemic sample was made from Rh_2(R/S-TPPTTL)$_4$ (as a reaction with a chiral $Rh_2(OBz)_4$ resulted in lower yield and more complicated reaction mixture)*

R_f (1H/1DCM) = 0.80

^{1}H NMR (800 MHz, $CDCl_3$) δ δ 7.24 – 7.16 (m, 1H) (mixture of two rotamers), 7.12 (d, *J* = 7.6 Hz, 1H) (mixture of two rotamers), 7.03 (td, *J* = 7.5, 1.7 Hz, 1H) (mixture of two rotamers), 7.02 – 6.94 (m, 1H) (mixture of two rotamers), 6.94 – 6.88 (m, 1H) (mixture of two rotamers), 6.88 – 6.81 (m, 2H) (mixture of two rotamers), 6.44 (s, 0.5H) (major rotamer), 5.97 (s, 0.44H) (minor rotamer), 5.79 (s, 0.44H) (minor rotamer), 5.63 (s, 0.5H) (major rotamer), 5.58 (s, 0.44H) (minor rotamer), 5.03 (s, 0.5H) (major rotamer), 4.84 (d, *J* = 12.0 Hz, 0.44H) (minor rotamer, -CH_2Cl_3), 4.78 (d, *J* = 11.9 Hz, 0.5H) (major rotamer, -CH_2Cl_3), 4.73 (d, *J* = 12.0 Hz, 0.5H) (major rotamer, -CH_2Cl_3), 4.69 (d, *J* = 11.9 Hz, 0.44H) (minor rotamer, -CH_2Cl_3), 3.42 – 3.28 (m, 1.5H) (mixture of two rotamers), 3.12 – 2.90 (m, 3.5H) (mixture of two rotamers), 2.80 (q, *J* = 19.1 Hz, 2H) (mixture of two rotamers), 2.54 (s, 0.44H) (minor rotamer), 2.28 (d, *J* = 15.4 Hz, 0.5H) (major rotamer). (*Total 20H*)

^{13}C NMR (201 MHz, $CDCl_3$) δ 171.60, 141.50, 140.65, 139.04, 138.21, 137.70, 136.88, 136.72, 136.64, 136.43, 134.10, 133.80, 133.59, 133.42, 132.08, 131.45, 131.29, 130.77, 130.76, 129.15, 128.53, 127.45, 126.41, 126.27, 126.02, 125.26, 125.16, 125.00, 123.91, 119.32, 118.38, 94.69, 74.92, 54.63, 54.55, 37.11, 36.83, 35.41, 35.31, 32.54, 31.73, 31.52. *(mixture of two rotamers due to hinder rotation around C-C bond of o-Cl-Ph group and cycloheptatriene)*

HRMS (+p APCI) calcd. for [$C_{26}H_{21}O_2{}^{79}Br_2{}^{35}Cl_4$] ([M+H]$^+$) 662.8657 found 662.8657

HPLC (Chiralpak ADH column, 1% i-propanol in hexane, 1.0 mLmin^{-1}, 1.0 mgmL^{-1}, 15 min, UV 230 nm) retention times of 6.35 min (minor) and 7.61 min (major), 95% ee.

$[\alpha]^{20}_D$: -2.46° (c = 1.47 g/100 ml, $CHCl_3$, 95% ee)

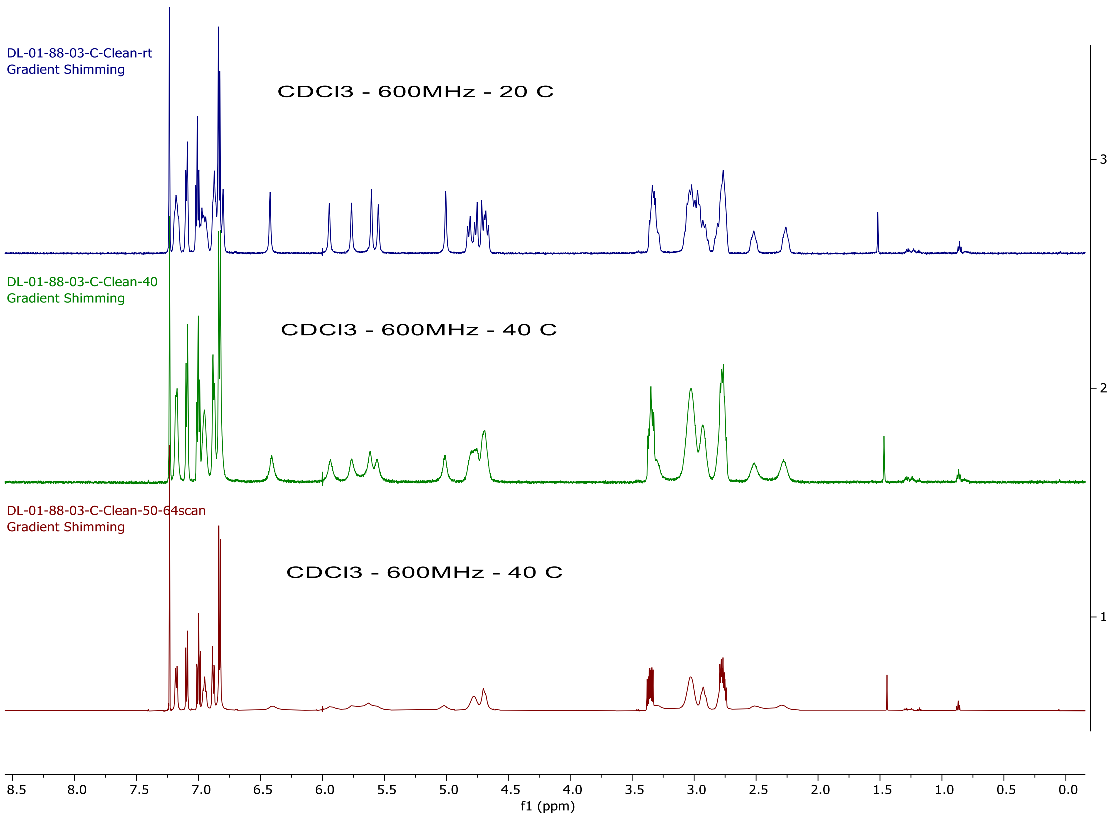

Figure S18. Varied temperajure ^{1}H-NMR ($CDCl_3$) of **3dm** *(Higher temperature helps only deconvolute aromatic region)*

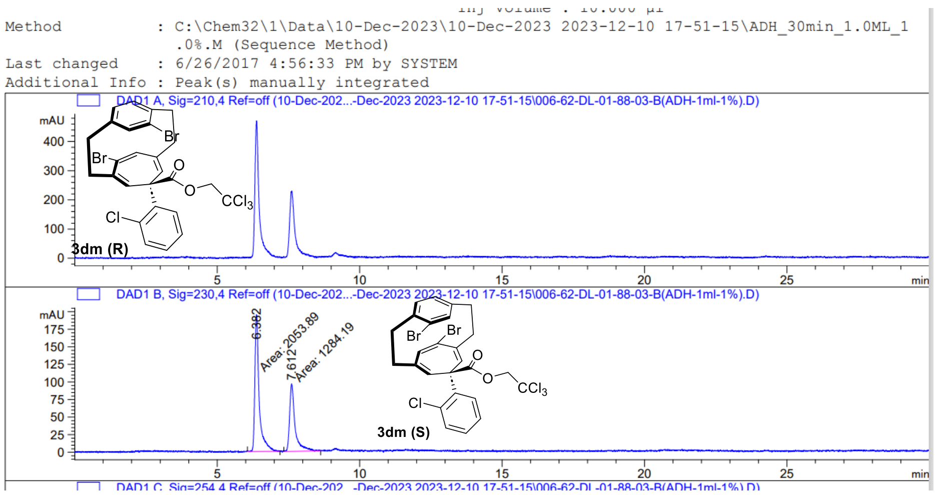

Signal 2: DAD1 B, Sig=230,4 Ref=off

Peak #	RetTime [min]	Type	Width [min]	Area [mAU*s]	Height [mAU]	Area %
1	6.382	MM	0.1759	2053.89355	194.64998	61.5292
2	7.612	MM	0.2234	1284.18677	95.82401	38.4708

Totals : 3338.08032 290.47398

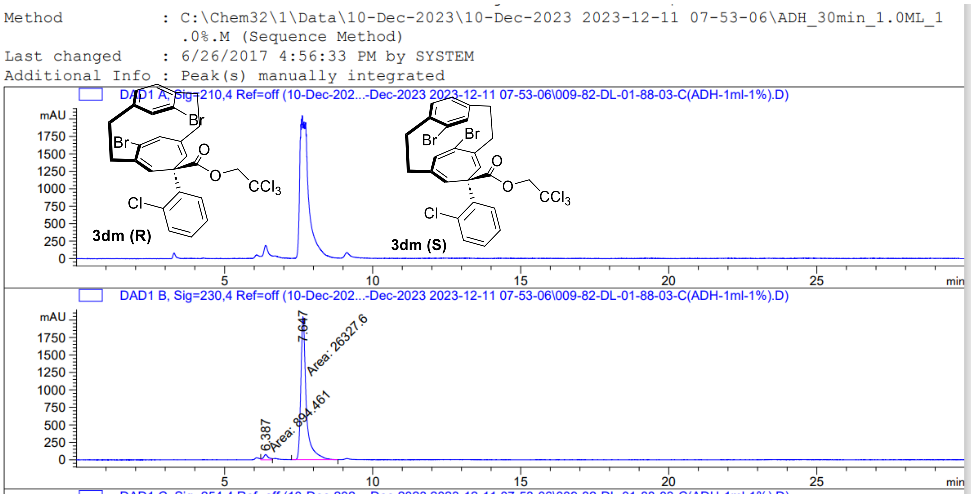

Signal 2: DAD1 B, Sig=230,4 Ref=off

Peak #	RetTime [min]	Type	Width [min]	Area [mAU*s]	Height [mAU]	Area %
1	6.382	MM	0.1759	2053.89355	194.64998	61.5292
2	7.612	MM	0.2234	1284.18677	95.82401	38.4708

Totals : 3338.08032 290.47398

Figure S19. SFC trace for 3dm (racemic – top /chiral – bottom)

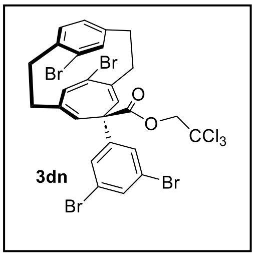

2,2,2-trichloroethyl (S,E)-12,43-dibromo-16-(3,5-dibromophenyl)-1(1,4)-cycloheptana-4(1,4)-benzenacyclohexaphane-11(17),12,14-triene-16-carboxylate (3dn)

Prepared according to general procedure B. 4,16-dibromo[2.2]paracyclophane (73.2 mg, 0.2 mmol, 1.0 equiv), MS 4Å (100 wt%), Rh_2(*S*-TPPTTL)$_4$ (2.46 mg, 0.0001 mmol, 0.005 equiv), and 2,2,2-trichloroethyl 2-(3,5-dibromophenyl)-2-diazoacetate (135 mg, 0.3 mmol, 1.5 equiv) were used. The crude mixture was then dry-loaded on silica, followed by flash chromatography (gradient 5%-25% DCM in hexane) afforded **3dn** as a white solid (122.9 mg, 78% yield, 91.5% ee).

R$_f$ (3H/1DCM) = 0.38

^{1}H NMR (400 MHz, CDCl$_3$) δ 7.38 (t, *J* = 1.8 Hz, 1H), 7.21 (dd, *J* = 7.7, 1.7 Hz, 1H), 7.13 (d, *J* = 1.7 Hz, 2H), 6.88 (d, *J* = 7.7 Hz, 1H), 6.75 (d, *J* = 1.7 Hz, 1H), 6.32 (s, 1H), 5.74 (s, 1H), 5.71 (s, 1H), 4.77 (d, *J* = 11.9 Hz, 1H), 4.73 (d, *J* = 11.9 Hz, 1H), 3.38 – 3.20 (m, 2H), 3.08 – 2.94 (m, 3H), 2.86 – 2.77 (m, 2H), 2.38 (ddd, *J* = 13.9, 10.2, 5.8 Hz, 1H).

^{13}C NMR (101 MHz, CDCl$_3$) δ 171.0, 142.8, 140.8, 138.5, 138.2, 136.7, 136.6, 135.3, 133.1, 131.0, 129.6, 129.3, 128.0, 127.2, 126.0, 125.9, 121.7, 94.5, 74.6, 55.6, 37.1, 35.5, 32.2, 31.2.

HRMS (+p APCI) calcd. for [$C_{26}H_{20}O_2{}^{79}Br_4{}^{35}Cl_3$] ([M+H]$^+$) 784.7257 found 784.7257

SFC: (ChiralCel OJ-3, 5% (50% methanol in isopropanol with 0.2% Formic Acid), 2.5 mL/min, 1.0 mg/ml, 10 min, λ = 230 nm) retention times of 4.27 (minor) and 5.21 min (major) 91.5% ee.

$[\alpha]^{20}_{D}$: -6.41° (c = 0.32 g/100 ml, $CHCl_3$, 91.5% ee)

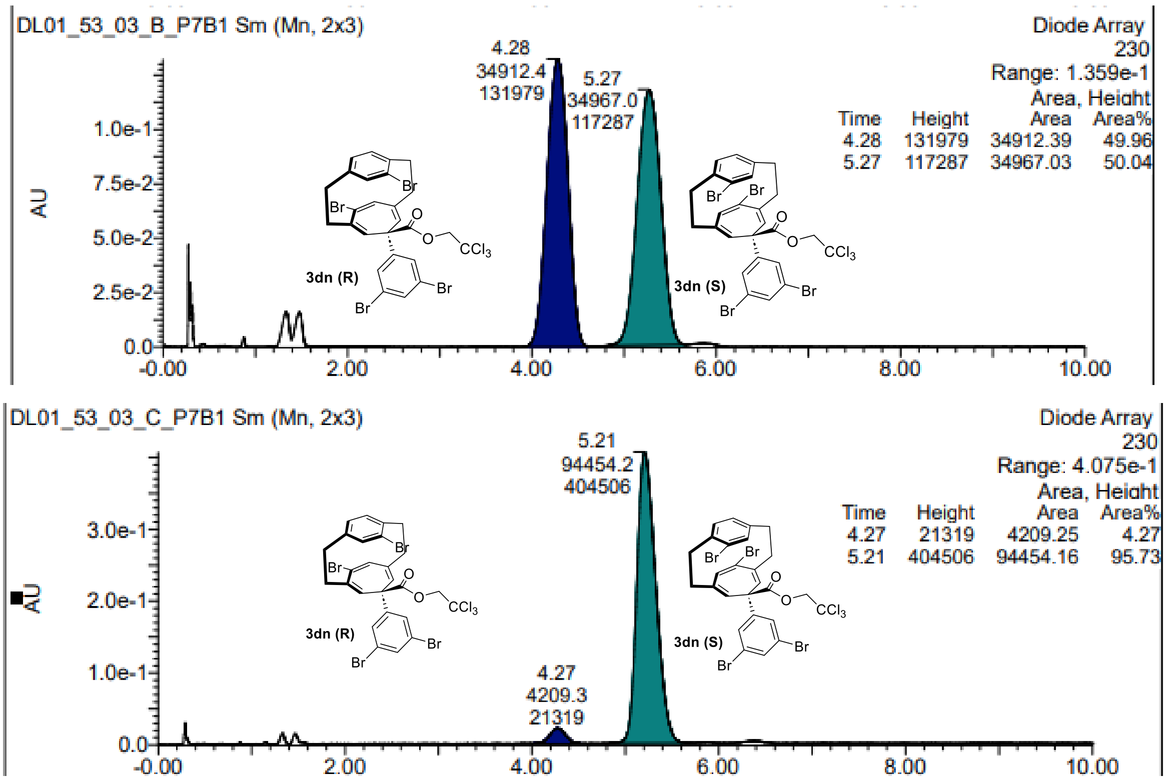

Figure S20. SFC trace for 3dn (racemic – top /chiral – bottom)

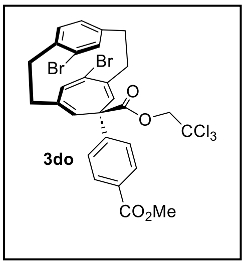

2,2,2-trichloroethyl (S,E)-12,43-dibromo-16-(4-(methoxycarbonyl)phenyl)-1(1,4)-cycloheptana-4(1,4)-benzenacyclohexaphane-11(17),12,14-triene-16-carboxylate (3do)

Prepared according to general procedure B. 4,16-dibromo[2.2]paracyclophane (73.2 mg, 0.2 mmol, 1.0 equiv), MS 4Å (100 wt%), Rh_2(*S*-TPPTTL)$_4$ (2.46 mg, 0.0001 mmol, 0.005 equiv), and methyl 4-(1-diazo-2-oxo-2-(2,2,2-trichloroethoxy)ethyl)benzoate (105 mg, 0.3 mmol, 1.5 equiv) were used. The crude mixture was then dry-loaded on silica, followed by flash chromatography (gradient 5%-20% DCM in hexane) afforded **3do** as a white solid (94.5 mg, 69% yield, 98% ee).

R_f (3H/1DCM) = 0.3

^{1}H NMR (800 MHz, $CDCl_3$) δ 7.70 (d, *J* = 7.5 Hz, 2H), 7.20 (d, *J* = 7.6 Hz, 1H), 7.16 (dd, *J* = 8.5, 1.2 Hz, 2H), 6.86 (d, *J* = 7.6 Hz, 1H), 6.77 (d, *J* = 1.7 Hz, 1H), 6.40 (s, 1H), 5.81 (s, 1H), 5.66 (s, 1H), 4.75 (d, *J* = 11.9 Hz, 1H), 4.68 (d, *J* = 12.0 Hz, 1H), 3.83 (s, 3H), 3.36 – 3.30 (m, 1H), 3.26 – 3.21 (m, 1H),

3.06 (ddd, J = 13.8, 10.0, 3.7 Hz, 1H), 3.03 – 2.94 (m, 2H), 2.85 – 2.78 (m, 2H), 2.36 (ddd, J = 14.1, 10.3, 5.9 Hz, 1H).

^{13}C NMR (201 MHz, $CDCl_3$) δ 171.5, 166.8, 144.2, 140.9, 138.5, 138.2, 136.7, 136.1, 134.9, 131.0, 129.3, 129.2, 128.4, 127.8, 127.8, 127.7, 127.0, 126.0, 94.5, 74.5, 56.1, 52.0, 37.0, 35.4, 32.3, 31.3.

HRMS (+p APCI) calcd. for [$C_{28}H_{24}O_4{}^{79}Br_2{}^{35}Cl_3$] ([M+H]$^+$) 686.9101 found 686.9099

HPLC (Chiralpak ADH column, 1% i-propanol in hexane, 1.0 mLmin^{-1}, 1.0 mgmL^{-1}, 15 min, UV 230 nm) retention times of 11.84 min (minor) and 18.69 min (major), 98% ee.

$[\alpha]^{20}_D$: -28.8° (c = 0.99 g/100 ml, $CHCl_3$, 98% ee)

```
Method          : C:\Chem32\1\Data\10-Dec-2023\10-Dec-2023 2023-12-12 21-39-04\ADH_30min_1.0ML_1
                  .0%.M (Sequence Method)
Last changed    : 6/26/2017 4:56:33 PM by SYSTEM
Additional Info : Peak(s) manually integrated
```

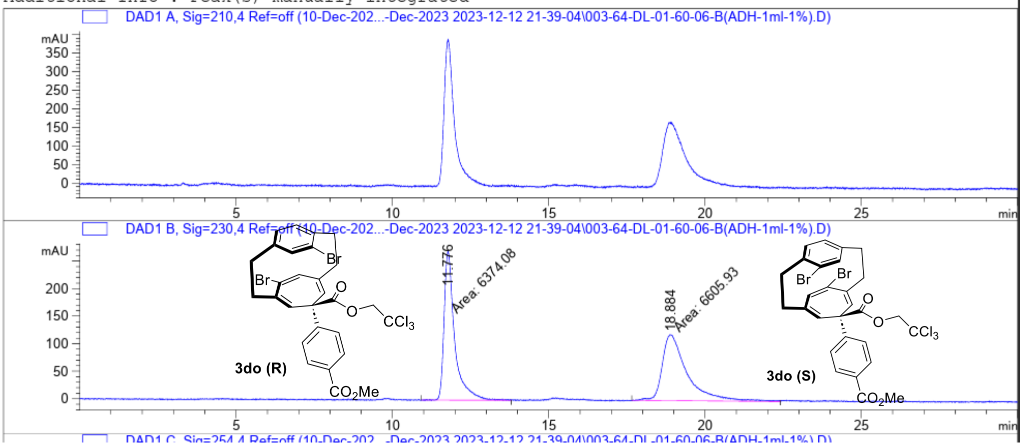

Signal 2: DAD1 B, Sig=230,4 Ref=off

Peak #	RetTime [min]	Type	Width [min]	Area [mAU*s]	Height [mAU]	Area %
1	11.776	MM	0.3931	6374.07764	270.24695	49.1069
2	18.884	MM	0.9191	6605.93457	119.79194	50.8931
Totals :				1.29800e4	390.03889	

```
Method          : C:\Chem32\1\Data\10-Dec-2023\10-Dec-2023 2023-12-12 21-39-04\ADH_30min_1.0ML_1
                  .0%.M (Sequence Method)
Last changed    : 6/26/2017 4:56:33 PM by SYSTEM
Additional Info : Peak(s) manually integrated
```

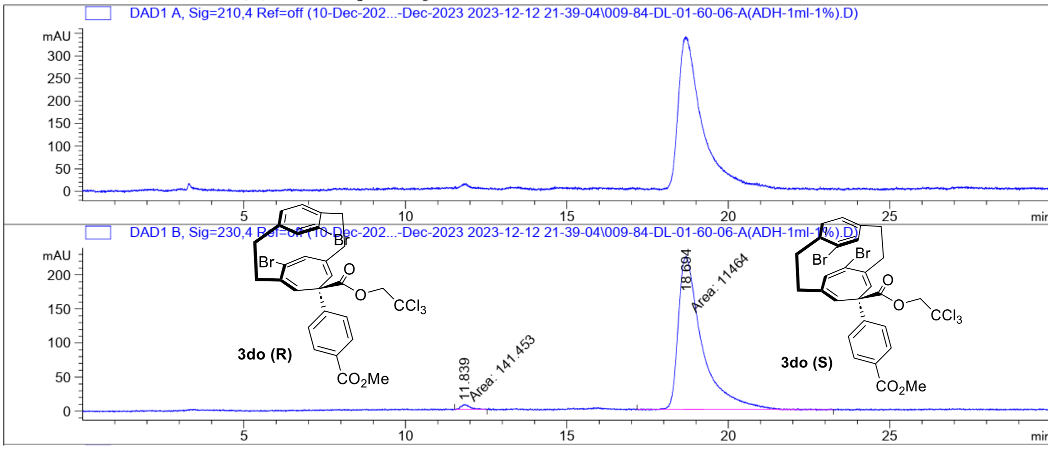

Signal 2: DAD1 B, Sig=230,4 Ref=off

Peak #	RetTime [min]	Type	Width [min]	Area [mAU*s]	Height [mAU]	Area %
1	11.839	MM	0.3243	141.45314	7.27009	1.2188
2	18.694	MM	0.8490	1.14640e4	225.05014	98.7812
Totals :				1.16055e4	232.32023	

Figure S21. SFC trace for 3do (racemic – top /chiral – bottom)

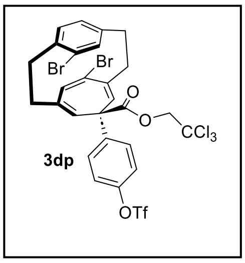

2,2,2-trichloroethyl (S,E)-12,43-dibromo-16-(4-(((trifluoromethyl)sulfonyl)oxy)phenyl)-1(1,4)-cycloheptana-4(1,4)-benzenacyclohexaphane-11(17),12,14-triene-16-carboxylate (3dp)

Prepared according to general procedure B. 4,16-dibromo[2.2]paracyclophane (73.2 mg, 0.2 mmol, 1.0 equiv), MS 4Å (100 wt%), Rh_2(*S*-TPPTTL)$_4$ (2.46 mg, 0.0001 mmol, 0.005 equiv), and 2,2,2-trichloroethyl 2-diazo-2-(4-(((trifluoromethyl)sulfonyl)oxy)phenyl)acetate (132 mg, 0.3 mmol, 1.5 equiv) were used. The crude mixture was then dry-loaded on silica, followed by flash chromatography (gradient 5%-20% DCM in hexane) afforded **3dp** as a white solid (117.6 mg, 76% yield, 95% ee).

R_f (3H/1DCM) = 0.3

^{1}H NMR (800 MHz, $CDCl_3$) δ 7.20 (dd, *J* = 7.6, 1.7 Hz, 1H), 7.16 (d, *J* = 9.0 Hz, 2H), 6.94 (d, *J* = 9.0 Hz, 2H), 6.87 (d, *J* = 7.6 Hz, 1H), 6.38 (s, 1H), 5.80 (s, 1H), 5.70 (s, 1H), 4.77 (d, *J* = 11.9 Hz, 1H), 4.71 (d, *J* = 11.9 Hz, 1H), 3.33 (ddd, *J* = 13.4, 10.0, 5.9 Hz, 1H), 3.23 (dddd, *J* = 14.5, 9.1, 3.7, 1.4 Hz, 1H), 3.05 (ddd, *J* = 13.9, 10.0, 3.8 Hz, 1H), 3.03 – 2.95 (m, 2H), 2.85 – 2.79 (m, 2H), 2.40 – 2.34 (m, 1H).

^{13}C NMR (201 MHz, $CDCl_3$) δ 171.5, 148.7, 141.0, 139.5, 138.6, 138.4, 136.8, 136.4, 135.2, 131.1, 129.6, 129.5, 127.9, 127.8, 127.1, 126.1, 120.0, 118.8 (q, *J* = 321 Hz), 94.6, 74.6, 55.7, 37.1, 35.5, 32.4, 31.4.

^{19}F NMR (753 MHz, $CDCl_3$) δ -72.77.

HRMS (+p APCI) calcd. for [$C_{27}H_{21}O_5{}^{79}Br_2{}^{35}Cl_3F_3{}^{32}S$] ($[M+H]^+$) 776.8489 found 776.8485

HPLC (Chiralpak ADH column, 1% i-propanol in hexane, 1.0 $mLmin^{-1}$, 1.0 $mgmL^{-1}$, 15 min, UV 230 nm) retention times of 6.93 min (minor) and 7.85 min (major), 95% ee.

$[\alpha]^{20}_D$: -22.0° (c = 0.77 g/100 ml, $CHCl_3$, 95% ee)

```
Method          : C:\Chem32\1\Data\10-Dec-2023\10-Dec-2023 2023-12-10 17-51-15\ADH_30min_1.0ML_1
                  .0%.M (Sequence Method)
Last changed    : 6/26/2017 4:56:33 PM by SYSTEM
Additional Info : Peak(s) manually integrated
```

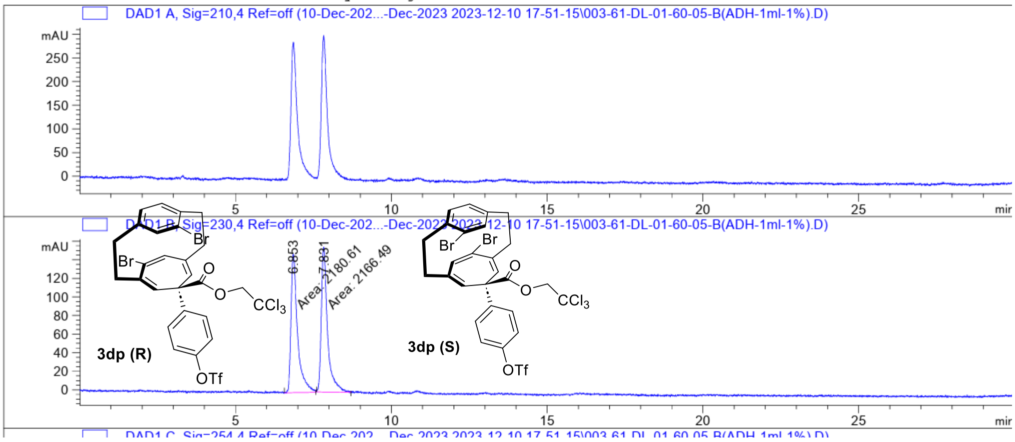

Signal 2: DAD1 B, Sig=230,4 Ref=off

Peak #	RetTime [min]	Type	Width [min]	Area [mAU*s]	Height [mAU]	Area %
1	6.853	MM	0.2436	2180.60840	149.20715	50.1624
2	7.831	MM	0.2312	2166.48804	156.15683	49.8376
Totals :				4347.09644	305.36398	

```
Inj Volume : 10.000 µl
Method          : C:\Chem32\1\Data\10-Dec-2023\10-Dec-2023 2023-12-11 07-53-06\ADH_30min_1.0ML_1
                  .0%.M (Sequence Method)
Last changed    : 6/26/2017 4:56:33 PM by SYSTEM
Additional Info : Peak(s) manually integrated
```

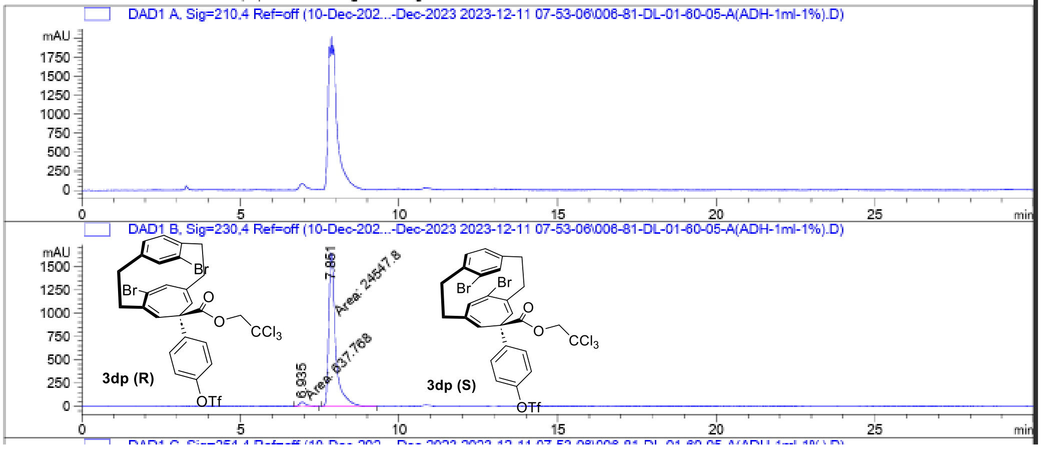

Signal 2: DAD1 B, Sig=230,4 Ref=off

Peak #	RetTime [min]	Type	Width [min]	Area [mAU*s]	Height [mAU]	Area %
1	6.935	MM	0.2446	637.76831	43.44785	2.5323
2	7.851	MM	0.2481	2.45478e4	1648.75085	97.4677
Totals :				2.51856e4	1692.19871	

Figure S22. SFC trace for 3dp (racemic – top /chiral – bottom)

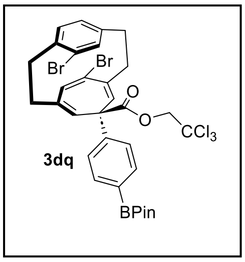

2,2,2-trichloroethyl (S,E)-12,43-dibromo-16-(4-(4,4,5,5-tetramethyl-1,3,2-dioxaborolan-2-yl)phenyl)-1(1,4)-cycloheptana-4(1,4)-benzenacyclohexaphane-11(17),12,14-triene-16-carboxylate (3dq)

Prepared according to general procedure B. 4,16-dibromo[2.2]paracyclophane (73.2 mg, 0.2 mmol, 1.0 equiv), MS 4Å (100 wt%), $Rh_2(S\text{-TPPTTL})_4$ (2.46 mg, 0.0001 mmol, 0.005 equiv), and 2,2,2-trichloroethyl 2-diazo-2-(4-(4,4,5,5-tetramethyl-1,3,2-dioxaborolan-2-yl)phenyl)acetate (126 mg, 0.3 mmol, 1.5 equiv) were used. The crude mixture was then dry-loaded on silica, followed by flash chromatography (gradient 5%-20% DCM in hexane) afforded **3dq** as a white solid (56.7 mg, 37% yield, 90% ee).

R_f (1H/1DCM) = 0.25

^{1}H NMR (800 MHz, $CDCl_3$) δ 7.46 (d, J = 8.3 Hz, 2H), 7.20 (d, J = 7.5 Hz, 1H), 7.07 (d, J = 8.6 Hz, 2H), 6.86 (d, J = 7.6 Hz, 1H), 6.77 (s, 1H), 6.41 (s, 1H), 5.80 (s, 1H), 5.65 (s, 1H), 4.72 (d, J = 11.9 Hz, 1H), 4.65 (d, J = 11.9 Hz, 1H), 3.35 – 3.29 (m, 1H), 3.23 (ddd, J = 9.1, 3.9, 1.5 Hz, 1H), 3.04 (ddd, J = 13.4, 9.8, 3.4 Hz, 1H), 3.02 – 2.94 (m, 2H), 2.84 – 2.77 (m, 2H), 2.32 (ddd, J = 14.1, 10.3, 6.0 Hz, 1H), 1.28 (d, J = 3.5 Hz, 12H).

^{13}C NMR (201 MHz, $CDCl_3$) δ 172.08, 142.02, 141.00, 138.64, 138.24, 136.87, 135.84, 134.56, 133.69, 131.08, 129.39, 128.42, 127.84, 127.35, 127.16, 126.15, 94.83, 83.82, 74.66, 56.36, 37.19, 35.62, 32.43, 31.44, 25.05, 24.96. (missing 1 quaternary carbon of BPin group)

HRMS (+p APCI) calcd. for $[C_{32}H_{33}{}^{11}BO_4{}^{79}Br_2{}^{35}Cl_3]$ ($[M+H]^+$) 754.9904 found 754.9892

HPLC (SSWhelk column, 1% i-propanol in hexane, 1.0 $mLmin^{-1}$, 1.0 $mgmL^{-1}$, 15 min, UV 230 nm) retention times of 20.3 min (minor) and 23.0 min (major), 90% ee.

$[\alpha]^{20}_D$: -27.2° (c = 1.21 g/100 ml, $CHCl_3$, 90% ee)

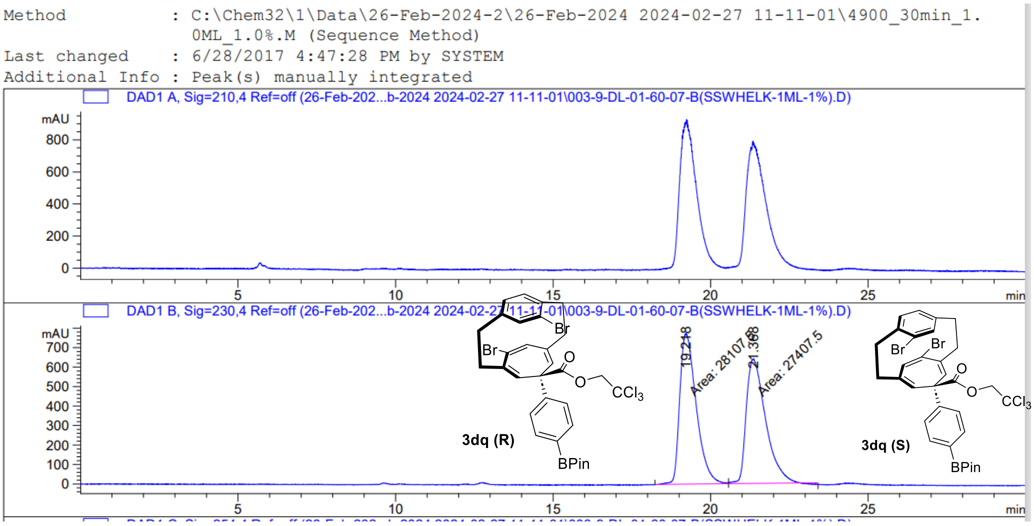

Signal 2: DAD1 B, Sig=230,4 Ref=off

Peak #	RetTime [min]	Type	Width [min]	Area [mAU*s]	Height [mAU]	Area %
1	19.218	MF	0.6057	2.81075e4	773.43671	50.6305
2	21.368	FM	0.7138	2.74075e4	639.94604	49.3695
Totals :				5.55150e4	1413.38275	

Method : C:\Chem32\1\Data\26-Feb-2024-2\26-Feb-2024 2024-02-26 14-14-51\4900_30min_1.0ML_1.0%.M (Sequence Method)
Last changed : 6/28/2017 4:47:28 PM by SYSTEM
Additional Info : Peak(s) manually integrated

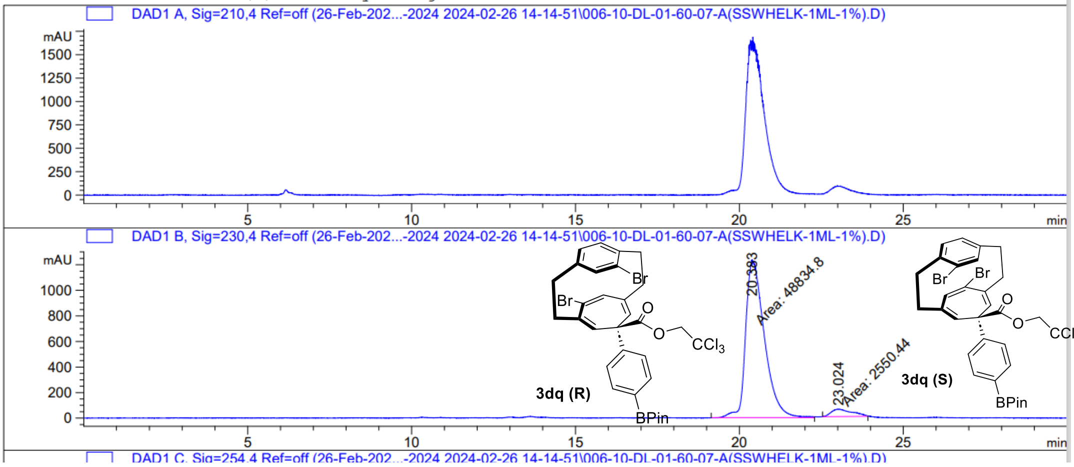

Signal 2: DAD1 B, Sig=230,4 Ref=off

Peak #	RetTime [min]	Type	Width [min]	Area [mAU*s]	Height [mAU]	Area %
1	20.383	MM	0.6589	4.88348e4	1235.33179	95.0366
2	23.024	MM	0.7287	2550.43799	58.33303	4.9634
Totals :				5.13852e4	1293.66481	

Figure S23. SFC trace for 3dq (racemic – top /chiral – bottom)

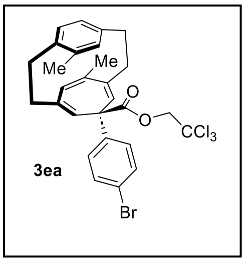

2,2,2-trichloroethyl (S,Z)-16-(4-bromophenyl)-12,43-dimethyl-1(1,4)-cycloheptana-4(1,4)-benzenacyclohexaphane-11(17),12,14-triene-16-carboxylate (3ea)

Prepared according to general procedure B. 4,16-dimethyl[2.2]paracyclophane (47.2 mg, 0.2 mmol, 1.0 equiv), MS 4Å (100 wt%), Rh_2(*S*-TPPTTL)$_4$ (2.46 mg, 0.0001 mmol, 0.005 equiv), and 2,2,2-trichloroethyl 2-(4-bromophenyl)-2-diazoacetate (112 mg, 0.3 mmol, 1.5 equiv) were used. The crude mixture was then dry-loaded on silica, followed by flash chromatography (gradient 5%-25% DCM in hexane) afforded **3ea** as a white solid (85.7 mg, 74% yield, 87% ee).

R_f (3H/1DCM) = 0.33

^{1}H NMR (400 MHz, CDCl$_3$) δ 7.08 (d, J = 8.7 Hz, 2H), 6.96 (d, J = 8.6 Hz, 2H), 6.82 (dd, J = 7.6, 1.8 Hz, 1H), 6.68 (d, J = 7.6 Hz, 1H), 6.39 – 6.30 (m, 1H), 6.00 (s, 1H), 5.55 (s, 1H), 4.95 (d, J = 1.4 Hz, 1H), 4.73 (d, J = 11.9 Hz, 1H), 4.65 (d, J = 11.9 Hz, 1H), 3.12 – 2.93 (m, 3H), 2.91 – 2.78 (m, 2H), 2.77 – 2.67 (m, 2H), 2.36 (ddd, J = 13.7, 10.3, 5.7 Hz, 1H), 2.07 (s, 3H), 1.41 (d, J = 1.4 Hz, 3H).

^{13}C NMR (101 MHz, CDCl$_3$) δ 172.7, 139.6, 138.7, 138.2, 138.1, 137.9, 136.2, 135.8, 134.4, 133.5, 130.0, 129.7, 129.4, 126.6, 123.9, 123.3, 120.5, 94.8, 74.3, 55.2, 36.1, 35.6, 31.9, 29.7, 22.5, 20.3.

HRMS (+p APCI) calcd. for [$C_{28}H_{27}O_2{}^{79}Br^{35}Cl_3$] ([M+H]$^+$) 579.0255 found 579.0256.

SFC: (ChiralCel OJ-3, 5% (50% methanol in isopropanol with 0.2% Formic Acid), 2.5 mL/min, 1.0 mg/ml, 10 min, λ = 230 nm) retention times of 2.15 (minor) and 2.72 min (major) 87% ee.

[α]$^{20}_D$: -31.7° (c = 0.29 g/100 ml, CHCl$_3$, 87% ee)

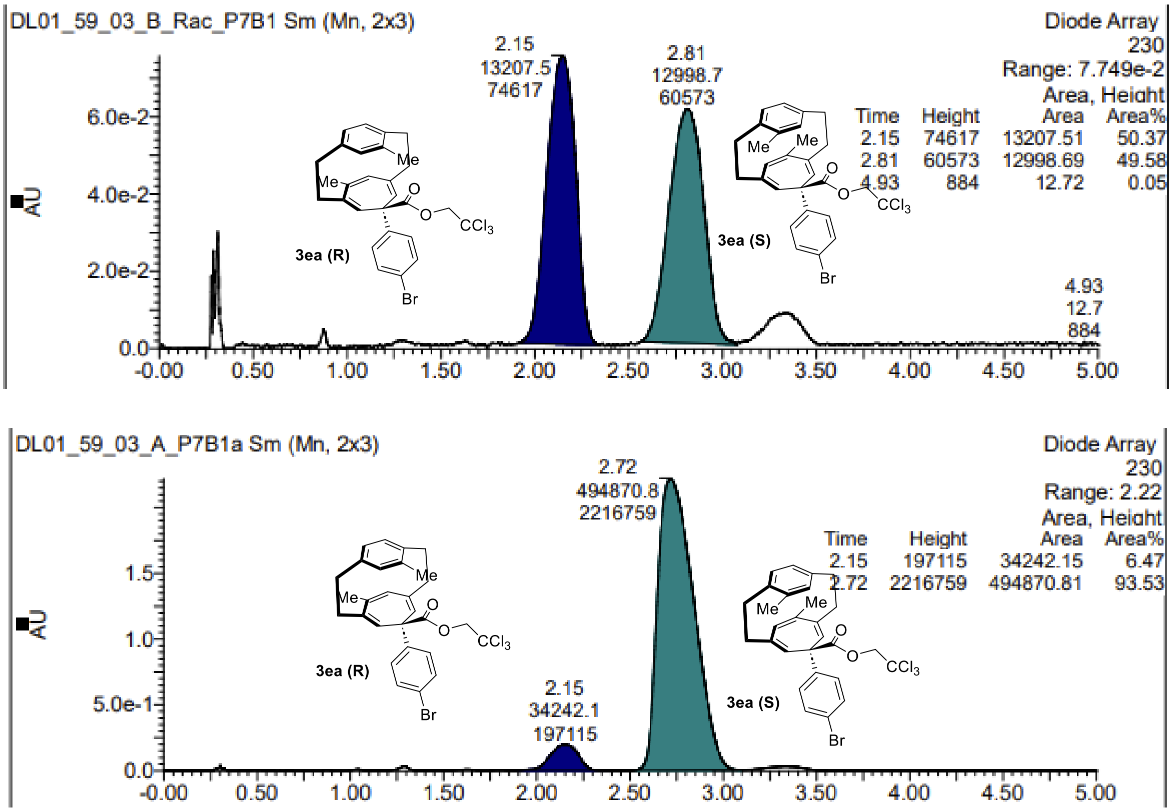

Figure S24. SFC trace for 3ea (racemic – top /chiral – bottom)

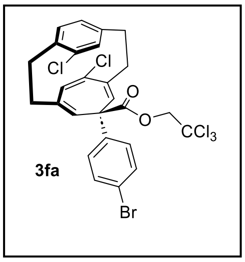

2,2,2-trichloroethyl (S,E)-16-(4-bromophenyl)-12,43-dichloro-1(1,4)-cycloheptana-4(1,4)-benzenacyclohexaphane-11(17),12,14-triene-16-carboxylate (3fa)

Prepared according to general procedure B. 4,16-dichloro[2.2]paracyclophane (1.00 g, 3.61 mmol, 1.0 equiv), MS 4Å (100 wt%), and Rh_2(*S*-TPPTTL)$_4$ (44.5 mg, 0.018 mmol, 0.005 equiv). 2,2,2-trichloroethyl 2-(4-bromophenyl)-2-diazoacetate (2.02 g, 5.41 mmol, 1.5 equiv) were used. The crude mixture was then dry-loaded on silica, followed by flash chromatography (gradient 5%-25% DCM in hexane) afforded **3fa** as a white solid (1.42 g, 63% yield, 90% ee). (*Note*: 0.2 mmol scale resulted in 60.6 mg, 49% yield, 90% ee; 2.0 g scale resulted in 3.97 g, 89% yield, 90% ee)

R_f (3H/1DCM) = 0.25

^{1}H NMR (400 MHz, $CDCl_3$) δ 7.14 (d, J = 8.6 Hz, 2H), 7.10 (dd, J = 7.7, 1.7 Hz, 1H), 6.95 (d, J = 8.6 Hz, 2H), 6.85 (d, J = 7.7 Hz, 1H), 6.55 (d, J = 1.7 Hz, 1H), 6.23 (s, 1H), 5.78 (s, 1H), 5.41 (s, 1H), 4.75 (d, J = 11.9 Hz, 1H), 4.68 (d, J = 11.9 Hz, 1H), 3.35 (ddd, J = 13.3, 9.9, 5.4 Hz, 1H), 3.22 (ddd, J = 14.4, 8.3, 4.4 Hz, 1H), 3.11 – 2.90 (m, 3H), 2.83 – 2.68 (m, 2H), 2.38 (ddd, J = 13.6, 10.2, 5.5 Hz, 1H).

^{13}C NMR (101 MHz, $CDCl_3$) δ 171.7, 140.8, 138.0, 136.8, 135.8, 135.0, 134.6, 134.5, 134.4, 133.8, 131.3, 130.2, 129.3, 128.5, 126.8, 126.6, 121.3, 94.6, 74.5, 55.6, 35.6, 35.5, 31.6, 30.1.

HRMS (+p APCI) calcd. for [$C_{26}H_{21}O_2{}^{79}Br^{35}Cl_5$] ([M+H]$^+$) 618.9162 found 618.9165

SFC: (ChiralCel OJ-3, 2% (50% methanol in isopropanol with 0.2% Formic Acid), 2.5 mL/min, 1.0 mg/ml, 10 min, λ = 230 nm) retention times of 6.34 (minor) and 8.65 min (major) 90% ee.

[α]$^{20}_D$: -55.8° (c = 0.51 g/100 ml, $CHCl_3$, 90% ee)

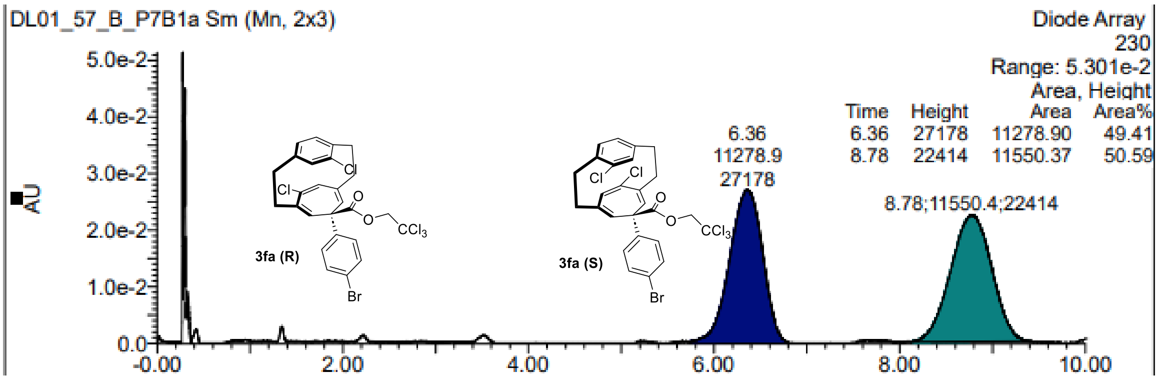

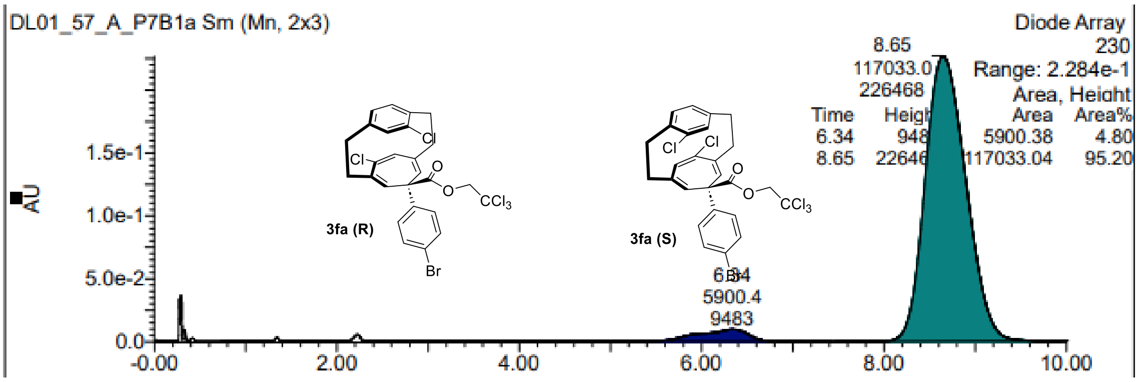

Figure S25. SFC trace for 3fa (racemic – top /chiral – bottom)

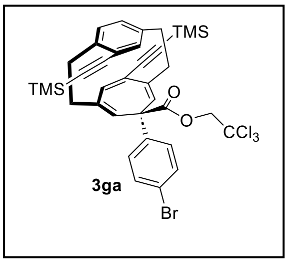

2,2,2-trichloroethyl (S,E)-16-(4-bromophenyl)-12,43-bis((trimethylsilyl)ethynyl)-1(1,4)-cycloheptana-4(1,4)-benzenacyclohexaphane-11(17),12,14-triene-16-carboxylate (3ga)

Prepared according to general procedure B. 12,43-bis((trimethylsilyl)ethynyl)-1,4(1,4)-dibenzenacyclohexaphane (95.4 mg, 0.2 mmol, 1.0 equiv), MS 4Å (100 wt%), Rh_2(*S*-TPPTTL)$_4$ (2.46 mg, 0.0001 mmol, 0.005 equiv), and 2,2,2-trichloroethyl 2-(4-bromophenyl)-2-diazoacetate (112 mg, 0.3 mmol, 1.5 equiv) were used. The crude mixture was then dry-loaded on silica, followed by flash chromatography (gradient 5%-25% DCM in hexane) afforded **3ga** as a white solid (75.4 mg, 50% yield, 94% ee).

R_f (2H/1DCM) = 0.34

^{1}H NMR (600 MHz, CDCl$_3$) δ 7.11 (d, *J* = 6.7 Hz, 3H), 6.96 (d, *J* = 6.8 Hz, 2H), 6.83 (d, *J* = 7.7 Hz, 1H), 6.68 (d, *J* = 2.1 Hz, 1H), 6.30 (s, 1H), 5.73 (s, 1H), 5.53 (s, 1H), 4.78 (d, *J* = 12.0 Hz, 1H), 4.55 (d, *J* = 12.0 Hz, 1H), 3.40 (ddd, *J* = 12.4, 10.0, 6.1 Hz, 1H), 3.22 (ddd, *J* = 13.9, 9.0, 4.1 Hz, 1H), 3.06 (ddd, *J* = 13.6, 9.9, 3.4 Hz, 1H), 3.01 – 2.91 (m, 2H), 2.77 (ddd, *J* = 14.9, 9.0, 2.8 Hz, 3H), 2.38 (ddd, *J* = 14.8, 10.0, 6.0 Hz, 1H), 0.27 (s, 9H), 0.20 (s, 9H).

^{13}C NMR (151 MHz, CDCl$_3$) δ 171.9, 142.0, 141.3, 138.8, 138.4, 136.9, 136.1, 134.7, 130.4, 130.0, 129.7, 129.7, 128.3, 125.9, 125.5, 123.8, 120.9, 105.8, 105.5, 97.5, 97.4, 94.6, 74.5, 55.5, 36.3, 36.1, 31.8, 31.3, 0.1, 0.0.

HRMS (+p APCI) calcd. for [$C_{36}H_{39}O_2{}^{79}Br^{35}Cl_3{}^{28}Si_2$] ([M+H]$^+$) 743.0732 found 743.0747.

HPLC (Chiralpak OD column, 0.5% i-propanol in hexane, 0.5 mLmin^{-1}, 1.0 mgmL^{-1}, 15 min, UV 230 nm) retention times of 6.92 min (minor) and 8.86 min (major), 94% ee.

[α]$^{20}_{D}$: -63.4° (c = 0.51 g/100 ml, CHCl$_3$, 94% ee)

```
Method          : C:\Chem32\1\Data\15-Dec-2023\15-Dec-2023 2023-12-23 10-31-50\OD_30min_0.50ML_0
                  .5%.M (Sequence Method)
Last changed    : 6/26/2017 4:13:55 PM by SYSTEM
Additional Info : Peak(s) manually integrated
```

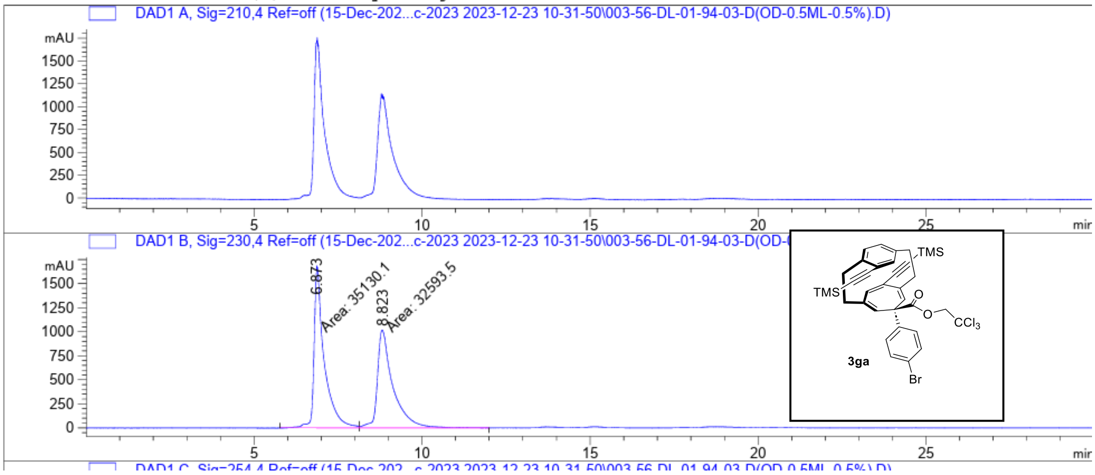


```
Signal 1: DAD1 A, Sig=210,4 Ref=off


Signal 2: DAD1 B, Sig=230,4 Ref=off

Peak RetTime Type  Width     Area      Height     Area
  #   [min]        [min]   [mAU*s]     [mAU]        %
----|-------|----|-------|----------|----------|--------|
   1   6.873 MF    0.3471 3.51301e4 1686.73999  51.8728
   2   8.823 FM    0.5332 3.25935e4 1018.75580  48.1272

Totals :                  6.77236e4  2705.49579
```

```
Method          : C:\Chem32\1\Data\15-Dec-2023\15-Dec-2023 2023-12-23 10-31-50\OD_30min_0.50ML_0
                  .5%.M (Sequence Method)
Last changed    : 6/26/2017 4:13:55 PM by SYSTEM
Additional Info : Peak(s) manually integrated
```

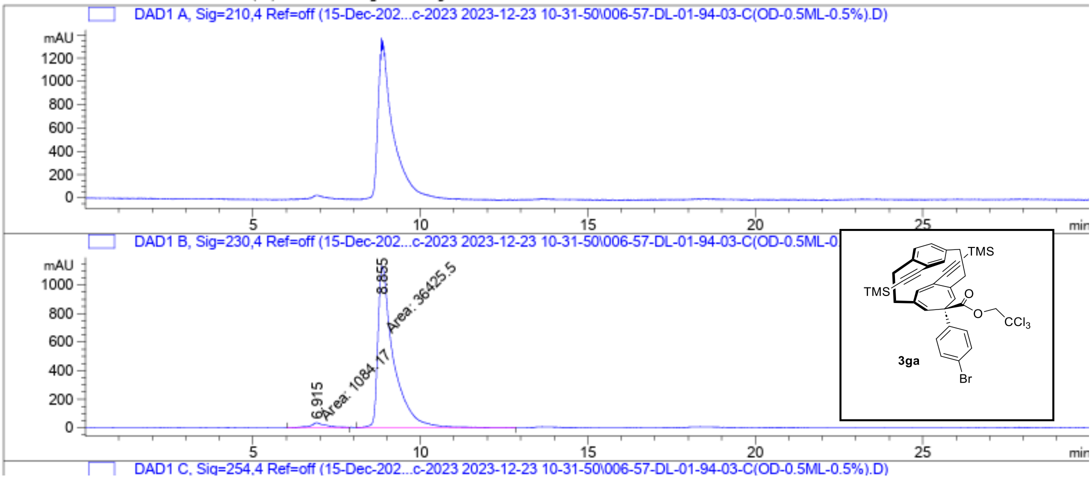


```
Signal 1: DAD1 A, Sig=210,4 Ref=off


Signal 2: DAD1 B, Sig=230,4 Ref=off

Peak RetTime Type  Width     Area      Height     Area
  #   [min]        [min]   [mAU*s]     [mAU]        %
----|-------|----|-------|----------|----------|--------|
   1   6.873 MF    0.3471 3.51301e4 1686.73999  51.8728
   2   8.823 FM    0.5332 3.25935e4 1018.75580  48.1272

Totals :                  6.77236e4  2705.49579
```

Figure S26. SFC trace for 3ga (racemic – top /chiral – bottom)

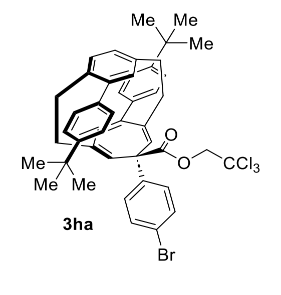

2,2,2-trichloroethyl (S,E)-16-(4-bromophenyl)-12,43-bis(4-(tert-butyl)phenyl)-1(1,4)-cycloheptana-4(1,4)-benzenacyclohexaphane-11(17),12,14-triene-16-carboxylate (3ha)

Prepared according to general procedure B. 4,16- bis(4-(tert-butyl)phenyl)[2.2]paracyclophane (94.5 mg, 0.2 mmol, 1.0 equiv), MS 4Å (100 wt%), Rh_2(*S*-TPPTTL)$_4$ (2.46 mg, 0.0001 mmol, 0.005 equiv), and 2,2,2-trichloroethyl 2-(4-bromophenyl)-2-diazoacetate (112 mg, 0.3 mmol, 1.5 equiv) were used. The crude mixture was then dry-loaded on silica, followed by flash chromatography (gradient 5%-25% DCM in hexane) afforded **3ha** as a white solid (8.9 mg, 7% yield, 78% ee).

R_f (2H/1DCM) = 0.34

^{1}H NMR (400 MHz, $CDCl_3$) δ 7.42 (d, *J* = 8.4 Hz, 2H), 7.36 – 7.29 (m, 4H), 7.18 (d, *J* = 7.8 Hz, 1H), 7.12 (d, *J* = 8.9 Hz, 2H), 7.08 (d, *J* = 9.0 Hz, 2H), 6.98 (dd, *J* = 7.8, 1.8 Hz, 1H), 6.94 (d, *J* = 8.3 Hz, 2H), 6.82 (d, *J* = 1.8 Hz, 1H), 6.26 (s, 1H), 5.68 (s, 1H), 5.61 (s, 1H), 4.77 (d, *J* = 11.8 Hz, 1H), 4.72 (d, *J* = 11.8 Hz, 1H), 3.28 (dt, *J* = 13.5, 8.0 Hz, 1H), 2.94 (ddd, *J* = 13.3, 8.6, 4.2 Hz, 1H), 2.87 – 2.71 (m, 3H), 2.63 – 2.48 (m, 2H), 2.30 (dt, *J* = 13.6, 8.1 Hz, 1H), 1.40 (s, 9H), 1.35 (s, 9H).

^{13}C NMR (101 MHz, $CDCl_3$) δ 172.5, 149.9, 149.6, 142.9, 141.6, 139.3, 139.3, 138.3, 138.2, 136.9, 136.6, 136.3, 133.8, 131.5, 131.5, 129.9, 129.7, 129.5, 129.3, 128.5, 127.6, 126.9, 125.3, 125.1, 120.9, 94.7, 74.6, 55.8, 36.8, 35.8, 34.5, 34.5, 33.0, 31.5, 31.5, 31.4.

HRMS (+p APCI) calcd. for [$C_{46}H_{47}O_2{}^{79}Br^{35}Cl_3$] ([M+H]$^+$) 815.1820 found 815.1815

SFC: (CEL1, 10% (50% methanol in isopropanol with 0.2% Formic Acid), 2.5 mL/min, 1.0 mg/ml, 10 min, λ = 230 nm) retention times of 5.87 (minor) and 10.53 min (major) 78% ee.

$[\alpha]^{20}_D$: -9.10° (c = 0.31 g/100 ml, $CHCl_3$, 78% ee)

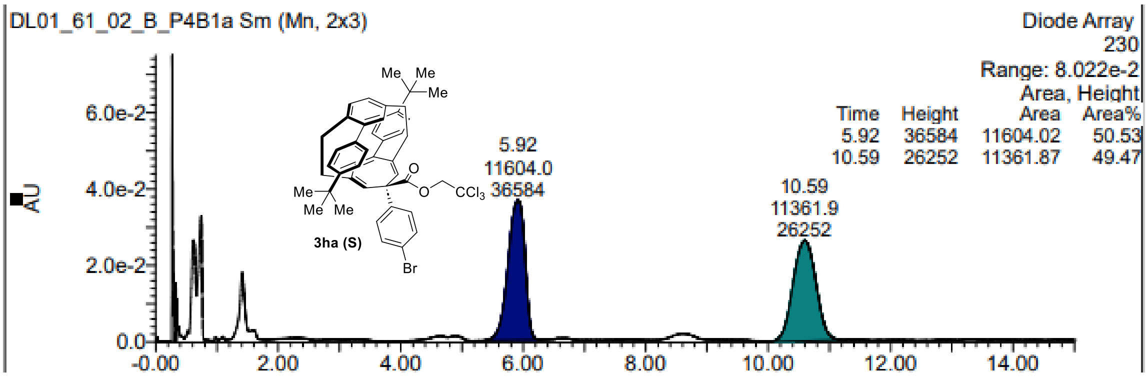

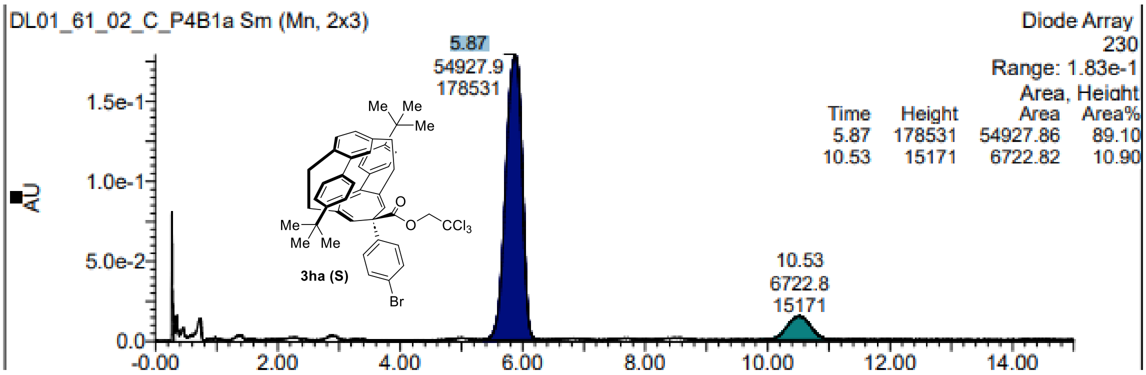

Figure S27. SFC trace for 3ha (racemic – top /chiral – bottom)

8. Second cyclopropanation of

8.1. Regio-selectivity optimization

Table S7. Catalyst screening for subsequent cyclopropanation on 3da

Note: *A second cyclopropanation also has site-selectivity challenging, as the 2nd cyclopropanation can occur at C1-C2 or C2-C3.*

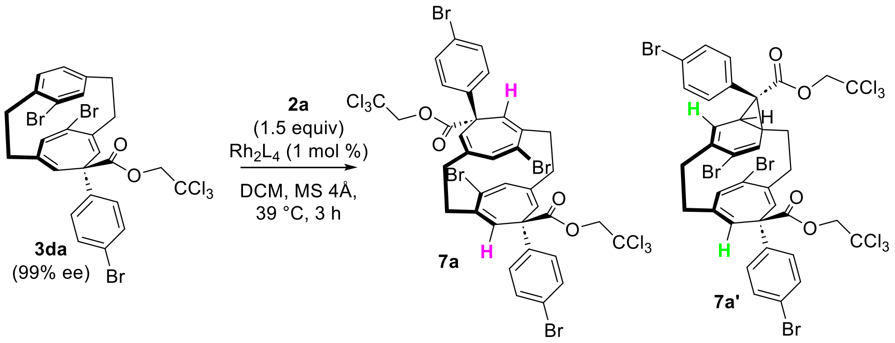

Entry[a]	Catalyst	Yield, %[b]	r.r.[c]	ee of 3da[e]
1[d]	Rh_2(*S*-TPPTTL)$_4$	29	1.5:1	97%
2	Rh_2(*R*-TPPTTL)$_4$	96	14:1	n.d.
3	$Rh_2(OBz)_4$	63	3:1	n.d.

[a]Reaction conditions: **3da** (0.1 mmol), **2a** (2.0 equiv), Rh_2L_4 (1 mol%), DCM (0.05 M), MS 4Å, 3-hour slow additions. [b]Isolated yields. [c]r.r. determined by crude NMR 6.23 ppm (s, 2H) for **7a** and 6.35 ppm (s, 1H) for **7a'**.[d] ee of 3da was 95%. [e]ee of recovered **3da**

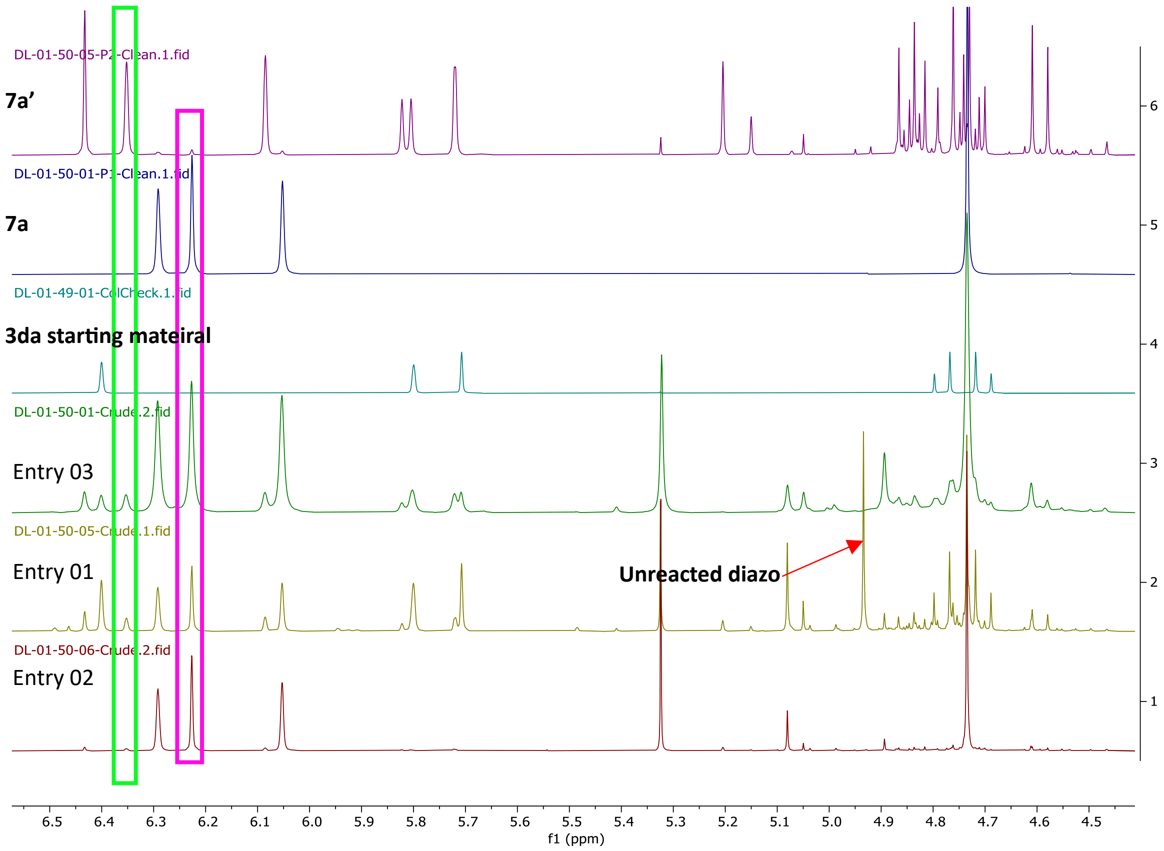

Figure S28. Crude NMR of **3da** cyclopropanation for r.r. determination. (highlighted in pink is signal belong to **7a**, while green is of **7a'**)

8.2. Cyclopropanation of 3da with different diazo

General procedure C for second Buchner reaction on **3da**:

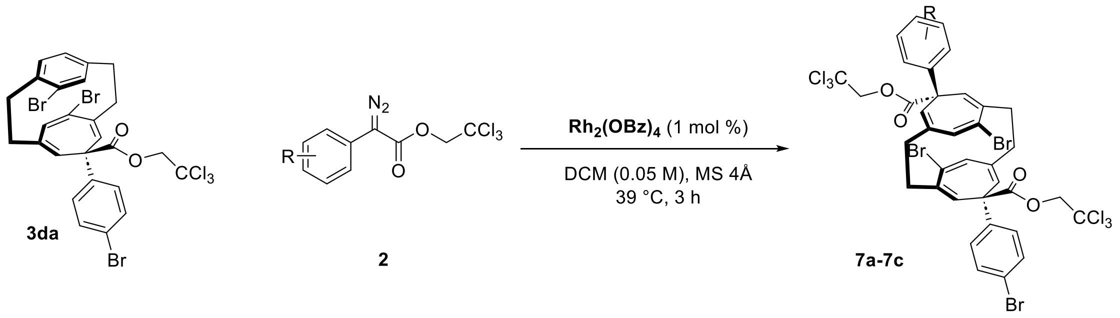

To a flame-dried clean 16.0 ml vials equipped with a magnetic stir-bar was evacuated and back-filled with nitrogen 3 times. After cooling down to room temperature, $Rh_2(OBz)_4$ (1 mol %) followed by **3da** (142 mg, 0.2 mmol, 1.0 equiv), and MS 4Å (100 wt%) were then added. The vial was once again evacuated and purged with argon (3-5 times) and dried dichloromethane (2.0 mL) was added. The vial and its contents were then set to stir at 40 °C by heating block under an nitrogen atmosphere. The diazo solution was prepared by adding diazo compound (0.3 mmol, 1.5 equiv) to the other 8 ml vial. The vial was evacuated and purged with argon (2-3 times) and of dry dichloromethane (2.0 mL) was then added to obtain a 0.15 M solution of the diazo compound. The 0.15 M solution was transferred into a plastic

syringe (3 mL, 9.83 mm diameter). The 0.15 M solution of diazo was then added slowly to a catalyst and trap solution within 3 hours by using a well-calibrated syringe pump (0.667 mL/h). After complete addition (3 hours later), the residual diazo compound in the 3.0 mL plastic syringe was rinsed with 0.5 mL dry dichloromethane and transferred dropwise into the stirring reaction mixture of vial-A. The mixture was stirred for an additional 15 min and then concentrated under reduced pressure. The mixture was then stirred overnight before filtered through celite to remove dust from MS 4Å then dried load on silica and purified by flash chromatography to obtain the purified product.

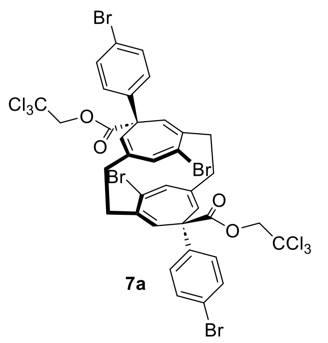

bis(2,2,2-trichloroethyl) (6R,15E,17E)-15,17-dibromo-6,13-bis(4-bromophenyl)tricyclo[9.3.2.24,8]octadeca-1(14),4,7,11,15,17-hexaene-6,13-dicarboxylate (7a)

Prepared according to general procedures **C**, **3da** (142 mg, 0.2 mmol, 1.0 equiv), $Rh_2(OBz)_4$ (1.23 mg, 0.0002 mmol, 0.01 equiv), and 2,2,2-trichloroethyl 2-(4-bromophenyl)-2-diazoacetate (111 mg, 0.3 mmol, 1.5 equiv) were used. The crude mixture was purified by flash chromatography (gradient 0%-10% Et_2O in hexane) afforded **7a** as a white solid (132 mg, 63%).

R_f (9H/1Et_2O) = 0.45

^{1}H NMR (400 MHz, $CDCl_3$) δ 7.20 (d, J = 8.6 Hz, 4H), 7.03 (d, J = 8.6 Hz, 4H), 6.27 (s, 2H), 6.20 (s, 2H), 6.03 (s, 2H), 4.71 (s, 4H), 3.15 – 2.92 (m, 4H), 2.75 – 2.59 (m, 2H), 2.45 – 2.33 (m, 2H).

^{13}C NMR (101 MHz, $CDCl_3$) δ 171.7, 137.6, 136.7, 135.4, 135.3, 130.4, 129.6, 127.8, 125.1, 125.1, 121.6, 94.6, 74.7, 55.5, 34.1, 34.0.

HRMS (+p APCI) calcd. for [$C_{36}H_{27}O_4{}^{79}Br_4{}^{35}Cl_6$] ([M+H]$^+$) 1048.6769 found 1048.6767.

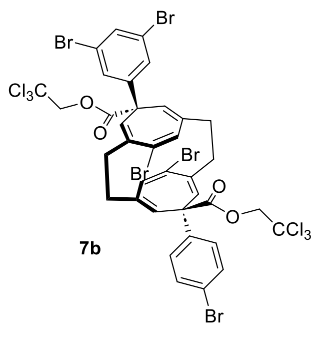

bis(2,2,2-trichloroethyl) (6R,15E,17E)-15,17-dibromo-6-(4-bromophenyl)-13-(3,5-dibromophenyl)tricyclo[9.3.2.24,8]octadeca-1(14),4,7,11,15,17-hexaene-6,13-dicarboxylate (7b)

Prepared according to general procedures **C**, **3da** (142 mg, 0.2 mmol, 1.0 equiv), $Rh_2(OBz)_4$ (1.23 mg, 0.0002 mmol, 0.01 equiv), and 2,2,2-trichloroethyl 2-diazo-2-(3,5-dibromophenyl)acetate (135 mg, 0.3 mmol, 1.5 equiv) were used. The crude mixture was purified by flash chromatography (gradient 0%-10% Et_2O in hexane) afforded **7b** as a white solid (151.0 mg, 67%, 99% ee).

R_f (9H/1Et_2O) = 0.35

^{1}H NMR (800 MHz, $CDCl_3$) δ 7.42 (s, 1H), 7.23 – 7.18 (m, 4H), 7.03 (d, J = 6.8 Hz, 2H), 6.28 (s, 1H), 6.26 (s, 1H), 6.23 (s, 1H), 6.20 (s, 1H), 6.04 (s, 1H), 5.97 (s, 1H), 4.76 (d, J = 10.2 Hz, 1H), 4.74 – 4.71 (m, 3H), 3.15 – 3.00 (m, 4H), 2.76 – 2.67 (m, 2H), 2.46 – 2.35 (m, 2H).

^{13}C NMR (201 MHz, $CDCl_3$) δ 171.6, 171.0, 142.6, 137.5, 136.8, 136.7, 136.1, 135.9, 135.3, 135.2, 133.4, 130.5, 129.9, 129.6, 128.2, 127.8, 125.244, 125.236, 124.7, 124.4, 121.9, 121.6, 94.6, 94.5, 74.8, 74.7, 55.5, 55.4, 34.1, 34.05, 34.01, 33.9.

HRMS (+p APCI) calcd. for [$C_{36}H_{26}O_4{}^{79}Br_5{}^{35}Cl_6$] ([M+H]$^+$) 1126.5874 found 1126.5855

HPLC (Chiralpak ADH column, 0.5% i-propanol in hexane, 1.0 mLmin^{-1}, 1.0 mgmL^{-1}, 30 min, UV 230 nm) retention times of 18.69 min (major) and 24.36 min (minor), 99% ee.

$[\alpha]^{20}_{D}$: -12.4° (c = 0.87 g/100 ml, $CHCl_3$, 99% ee)

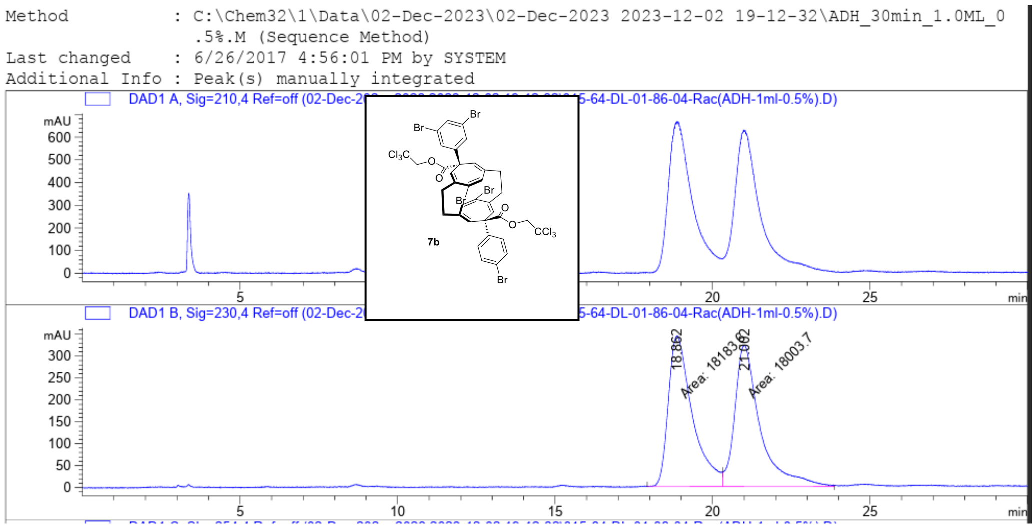

Signal 2: DAD1 B, Sig=230,4 Ref=off

Peak #	RetTime [min]	Type	Width [min]	Area [mAU*s]	Height [mAU]	Area %
1	18.862	MF	0.8819	1.81836e4	343.62564	50.2486
2	21.002	FM	0.9335	1.80037e4	321.44489	49.7514

Totals : 3.61873e4 665.07053

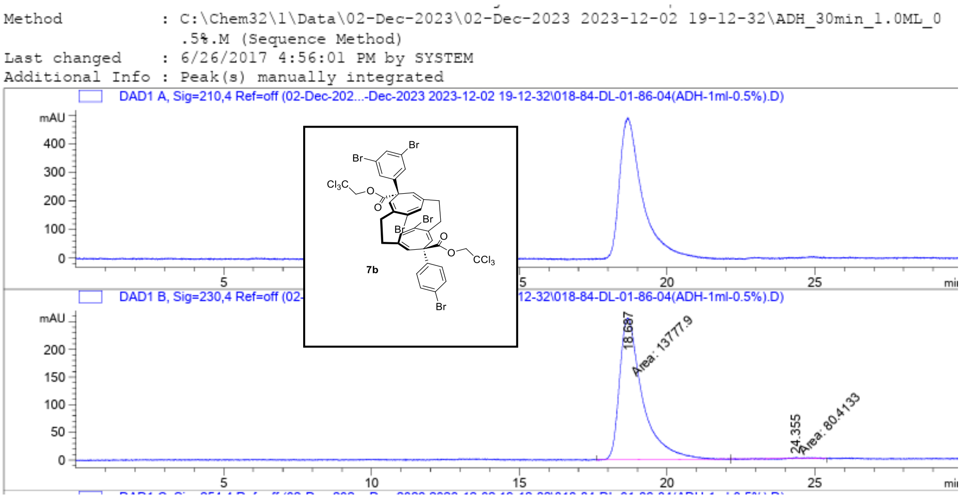

Signal 2: DAD1 B, Sig=230,4 Ref=off

Peak #	RetTime [min]	Type	Width [min]	Area [mAU*s]	Height [mAU]	Area %
1	18.687	MF	0.8974	1.37779e4	255.87688	99.4197
2	24.355	FM	0.5020	80.41326	2.66960	0.5803
Totals :				1.38583e4	258.54648	

Figure S29. SFC trace for 7b (racemic – top /chiral – bottom)

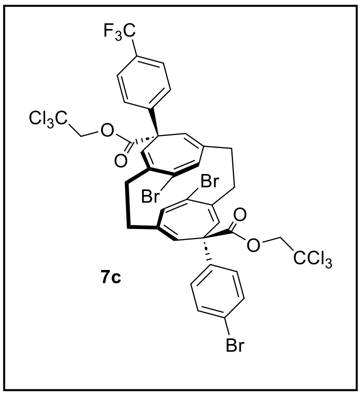

bis(2,2,2-trichloroethyl) (6R,15E,17E)-15,17-dibromo-6-(4-bromophenyl)-13-(4-(trifluoromethyl)phenyl)tricyclo[9.3.2.24,8]octadeca-1(14),4,7,11,15,17-hexaene-6,13-dicarboxylate (7c)

Prepared according to general procedures **C**, **3da** (142 mg, 0.2 mmol, 1.0 equiv), $Rh_2(OBz)_4$ (1.23 mg, 0.0002 mmol, 0.01 equiv), and 2,2,2-trichloroethyl 2-diazo-2-(4-(trifluoromethyl)phenyl)acetate (108 mg, 0.3 mmol, 1.5 equiv) were used. The crude mixture was purified by flash chromatography (gradient 0%-10% Et_2O in hexane) afforded **7c** as a white solid (157.1 mg, 75%, 98% ee).

R_f (9H/1Et_2O) = 0.35

^{1}H NMR (800 MHz, $CDCl_3$) δ 7.34 (d, J = 8.1 Hz, 2H), 7.28 (d, J = 8.1 Hz, 2H), 7.21 (d, J = 6.6 Hz, 2H), 7.03 (d, J = 6.7 Hz, 2H), 6.30 (s, 1H), 6.27 (s, 1H), 6.22 (s, 1H), 6.20 (s, 1H), 6.06 (s, 1H), 6.03 (s, 1H), 4.73 – 4.69 (m, 4H), 3.12 – 3.00 (m, 4H), 2.77 – 2.65 (m, 2H), 2.44 – 2.37 (m, 2H).

^{13}C NMR (201 MHz, CDCl$_3$) δ 171.7, 171.5, 142.7, 137.6, 136.8, 136.8, 135.7, 135.6, 135.4, 135.3, 130.5, 129.9 (q, *J* = 32.3 Hz), 129.6, 128.2, 127.9, 127.8, 125.2, 125.2, 125.0, 124.9, 124.3 (q, *J* = 3.7 Hz), 124.1 (q, *J* = 272.3 Hz), 121.6, 94.65, 94.57, 74.6. 74.68, 55.8, 55.5, 34.11, 34.09, 33.99, 33.98.

^{19}F NMR (753 MHz, CDCl$_3$) δ -62.54.

HRMS (+p APCI) calcd. for [$C_{37}H_{27}O_4{}^{79}Br_3{}^{35}Cl_6F_3$] ([M+H]$^+$) 1038.7537 found 1038.7522.
HPLC (Chiralpak ADH column, 0.5% i-propanol in hexane, 1.0 mLmin^{-1}, 1.0 mgmL^{-1}, 30 min, UV 230 nm) retention times of 20.14 min (major) and 23.31 min (minor), 98% ee.

[α]$^{20}_{D}$: -8.80° (c = 0.95 g/100 ml, CHCl$_3$, 98% ee)

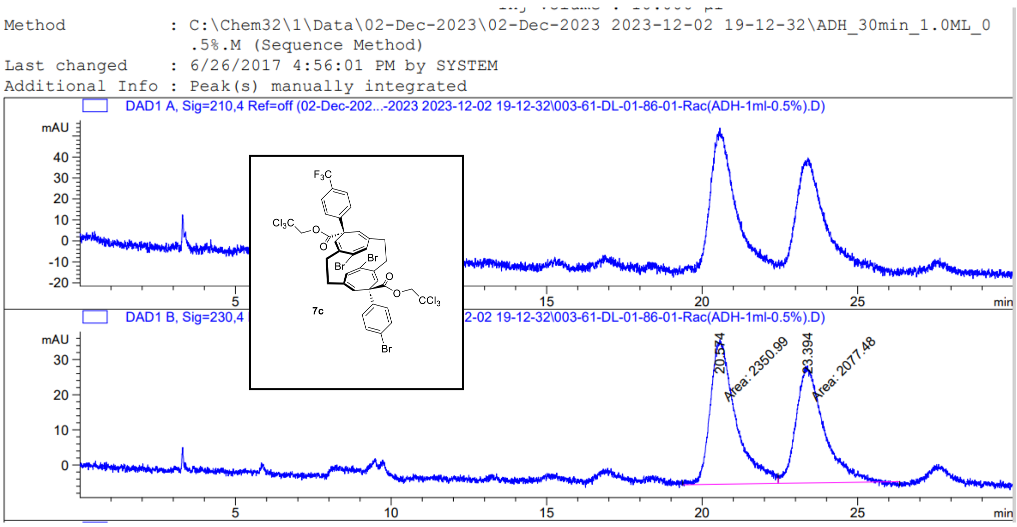

Signal 2: DAD1 B, Sig=230,4 Ref=off

Peak #	RetTime [min]	Type	Width [min]	Area [mAU*s]	Height [mAU]	Area %
1	20.574	MF	0.9502	2350.98682	41.23712	53.0881
2	23.394	FM	1.0385	2077.47681	33.34246	46.9119
Totals :				4428.46362	74.57958	

```
Method          : C:\Chem32\1\Data\02-Dec-2023\02-Dec-2023 2023-12-05 20-30-48\ADH_30min_1.0ML_0
                  .5%.M (Sequence Method)
Last changed    : 6/26/2017 4:56:01 PM by SYSTEM
Additional Info : Peak(s) manually integrated
```

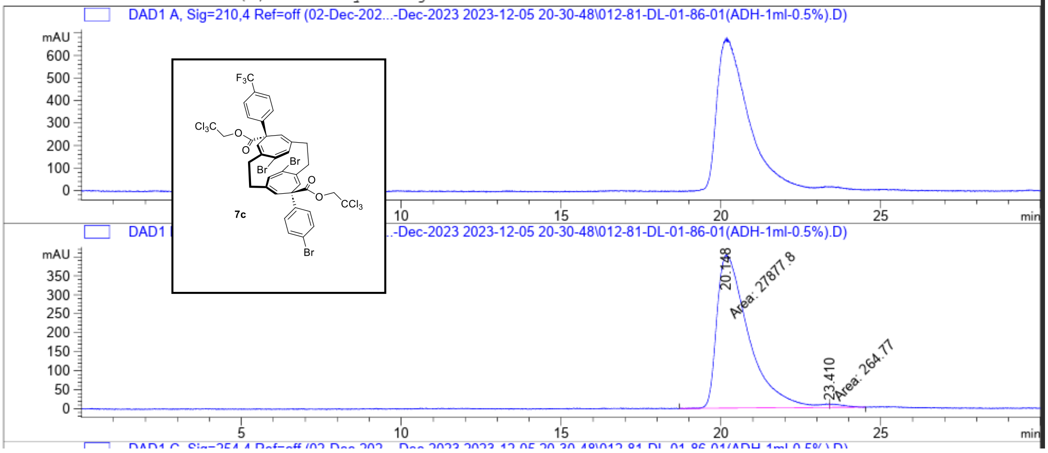


```
Signal 2: DAD1 B, Sig=230,4 Ref=off

Peak RetTime Type  Width     Area      Height     Area
  #   [min]        [min]   [mAU*s]     [mAU]       %
----|-------|----|-------|----------|----------|--------|
   1  20.148 MF    1.1506 2.78778e4  403.81259  99.0592
   2  23.410 FM    0.4678  264.77014   9.43327   0.9408

Totals :                  2.81426e4  413.24586
```

Figure S30. SFC trace for 7c (racemic – top /chiral – bottom)

9. Double cyclopropanation

9.1. Catalyst screening

Table S8. Catalyst screening for double cyclopropanation of 1d

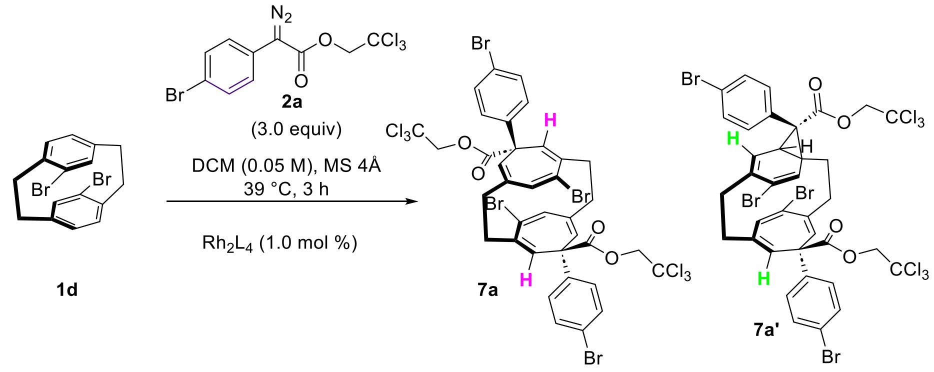

Entry[a]	Catalyst	Yield, %[b]	r.r.[c]
1	$Rh_2(OAc)_4$	n.d.	1.2:1
2	$Rh_2(OBz)_4$	39	1.5:1
3	Rh_2(R/S-TPPTTL)$_4$	85	7:1
4	Rh_2(R/S-NTTL)$_4$	n.d.	n.d.
5	Rh_2(R/S-DOSP)$_4$	n.d.	1:1

[a]Reaction conditions: **1d** (0.2 mmol), **2a** (3.0 equiv), Rh_2L_4 (1 mol%), DCM (0.05 M), MS 4Å, 3-hour slow additions. [b]Isolated yields. [c]r.r. was determined by crude NMR 6.23 ppm (s, 2H) for **7a** and 6.35 ppm (s, 1H) for **7a'**

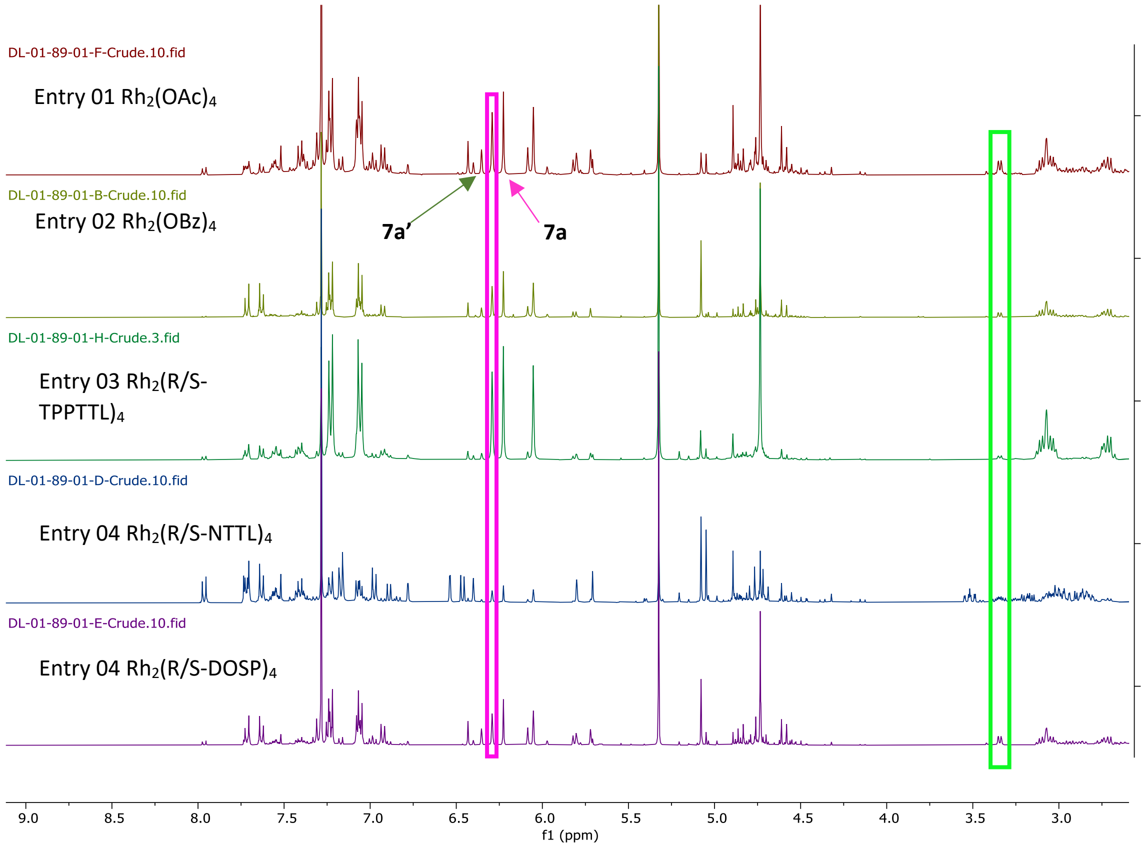

Figure S31. Crude NMR of **1d** cyclopropanation for r.r. determination. (highlighted in pink is signal belong to **7a**, while green is of **7a'**)

9.2. Diazo survey

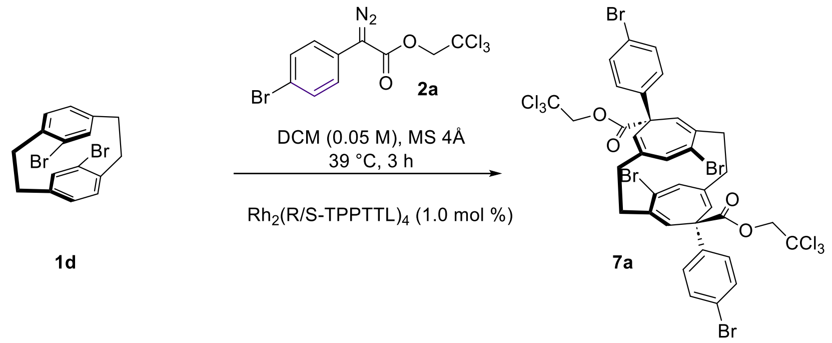

To an 16 ml vial was charged with 4,16-dibromo[2.2]paracyclophane (73.2 mg, 0.2 mmol, 1.0 equiv), MS 4Å (100 wt%), and Rh_2(*R/S*-TPPTTL)$_4$ (5.0 mg, 0.0002 mmol, 0.001 equiv). The vial was vacuumed and backfilled with nitrogen 3 times, before 2 ml of dry DCM was added. The mixture was then brought up to reflux (40 ºC) by heating block. A solution of 2,2,2-trichloroethyl 2-(4-bromophenyl)-2-diazoacetate (223 mg, 0.6 mmol, 3.0 equiv) in 1 ml of dry DCM was added dropwise to the above 8 ml vial within 1 hour by a well-calibrated syringe pump. After 3 hours, the mixture was

filtered through celite and washed by DCM to remove MS 4Å. The crude mixture was then dry-loaded on silica, followed by flash chromatography (gradient 0%-6% ether in hexane) afforded as **7a** as a white solid (178.8 mg, 85%).

R_f (9H/1Et_2O) = 0.45

^{1}H NMR (400 MHz, $CDCl_3$) δ 7.20 (d, J = 8.6 Hz, 4H), 7.03 (d, J = 8.6 Hz, 4H), 6.27 (s, 2H), 6.20 (s, 2H), 6.03 (s, 2H), 4.71 (s, 4H), 3.15 – 2.92 (m, 4H), 2.75 – 2.59 (m, 2H), 2.45 – 2.33 (m, 2H).

^{13}C NMR (101 MHz, $CDCl_3$) δ 171.7, 137.6, 136.7, 135.4, 135.3, 130.4, 129.6, 127.8, 125.1, 125.1, 121.6, 94.6, 74.7, 55.5, 34.1, 34.0.

HRMS (+p APCI) calcd. for [$C_{36}H_{27}O_4{}^{79}Br_4{}^{35}Cl_6$] ([M+H]$^+$) 1048.6769 found 1048.6767.

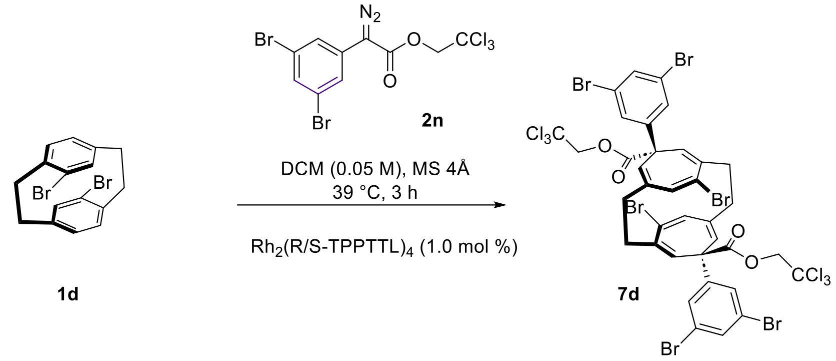

To an 16 ml vial was charged with 4,16-dibromo[2.2]paracyclophane (73.2 mg, 0.2 mmol, 1.0 equiv), MS 4Å (100 wt%), and Rh_2(*R/S*-TPPTTL)$_4$ (5.0 mg, 0.0002 mmol, 0.001 equiv). The vial was vacuumed and backfilled with nitrogen 3 times, before 2 ml of dry DCM was added. The mixture was then brought up to reflux (40 °C) by heating block. A solution of 2,2,2-trichloroethyl 2-diazo-2-(3,5-dibromophenyl)acetate (271 mg, 0.6 mmol, 3.0 equiv) in 1 ml of dry DCM was added dropwise to the above 8 ml vial within 1 hour by a well-calibrated syringe pump. After 3 hours, the solvent was removed, then hot toluene was then added to dissolve product, followed by filtering through celite and washed by DCM to remove MS 4Å. Because of low solubility, the product was purified by recrystallization instead of by flash chromatography. The crude mixture was dissolved in hot toluene, then upon cooling down solid product will form. The solid was collected, and the recrystallization process was repeated 2 more times to afford as **7d** as a white solid (113.9 mg, 47%). *Note: Yield could be higher with more careful recrystallization.*

R_f (9H/1Et_2O) = 0.42

^{1}H NMR (400 MHz, $CDCl_3$) δ 7.43 (t, J = 1.8 Hz, 2H), 7.22 (d, J = 1.8 Hz, 4H), 6.26 (s, 2H), 6.24 (s, 2H), 5.99 (s, 2H), 4.80 – 4.70 (m, 4H), 3.16 – 3.00 (m, 4H), 2.72 (dt, J = 11.7, 5.6 Hz, 2H), 2.43 (h, J = 7.4 Hz, 2H).

^{13}C NMR (101 MHz, CDCl$_3$) δ 171.0, 142.5, 136.7, 136.0, 135.9, 133.4, 129.9, 128.1, 124.8, 124.5, 122.0, 94.5, 74.8, 55.5, 34.0, 34.0.

HRMS (+p APCI) calcd. for [$C_{36}H_{25}O_4{}^{79}Br_4{}^{81}Br_2{}^{35}Cl_6$] ([M+H]$^+$) 1208.4911 found 1208.4935.

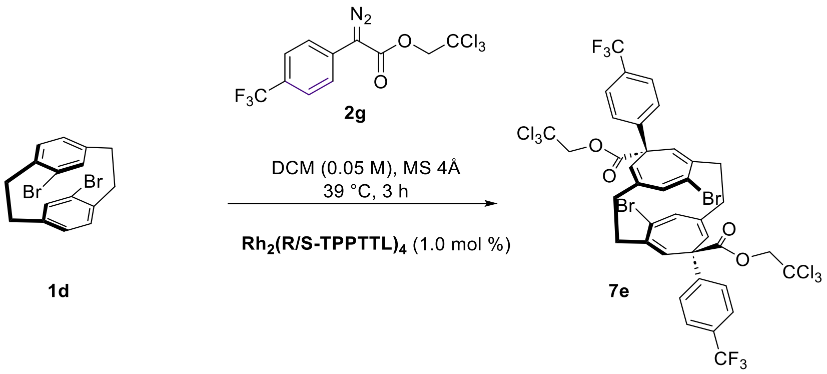

To an 16 ml vial was charged with 4,16-dibromo[2.2]paracyclophane (73.2 mg, 0.2 mmol, 1.0 equiv), MS 4Å (100 wt%), and Rh_2(*R/S*-TPPTTL)$_4$ (5.0 mg, 0.0002 mmol, 0.001 equiv). The vial was vacuumed and backfilled with nitrogen 3 times, before 2 ml of dry DCM was added. The mixture was then brought up to reflux (40 ºC) by heating block. A solution of 2,2,2-trichloroethyl 2-diazo-2-(4-(trifluoromethyl)phenyl)acetate (217 mg, 0.6 mmol, 3.0 equiv) in 1 ml of dry DCM was added dropwise to the above 8 ml vial within 1 hour by a well-calibrated syringe pump. After 3 hours, the mixture was filtered through celite and washed by DCM to remove MS 4Å. The crude mixture was then dry-loaded on silica, followed by flash chromatography (gradient 0%-15% ether in hexane) afforded an **7e** as a white solid (131.7 mg, 64%).

R_f (9H/1Et$_2$O) = 0.40

^{1}H NMR (600 MHz, CDCl$_3$) δ 7.35 (d, *J* = 8.1 Hz, 4H), 7.29 (d, *J* = 8.2 Hz, 4H), 6.30 (s, 2H), 6.21 (s, 2H), 6.07 (s, 2H), 4.71 (s, 4H), 3.15 – 3.01 (m, 4H), 2.72 (dt, *J* = 11.4, 5.7 Hz, 2H), 2.41 (dt, *J* = 14.0, 6.2 Hz, 2H).

^{19}F NMR (565 MHz, Chloroform-*d*) δ -62.55.

^{13}C NMR (151 MHz, CDCl$_3$) δ 171.4, 142.7, 136.9, 135.6, 135.5, 129.9 (q, *J* = 32.3 Hz), 128.2, 127.9, 125.0, 125.0, 124.3 (q, *J* = 3.8 Hz), 124.1 (q, *J* = 272.0 Hz), 94.6, 74.7, 55.8, 34.1, 34.0.

HRMS (+p APCI) calcd. for [$C_{38}H_{27}O_4{}^{79}Br_2{}^{35}Cl_6F_6$] ([M+H]$^+$) 1028.8306 found 1028.8297.

10. [2.2]paracyclophane-based dirhodium catalyst synthesis

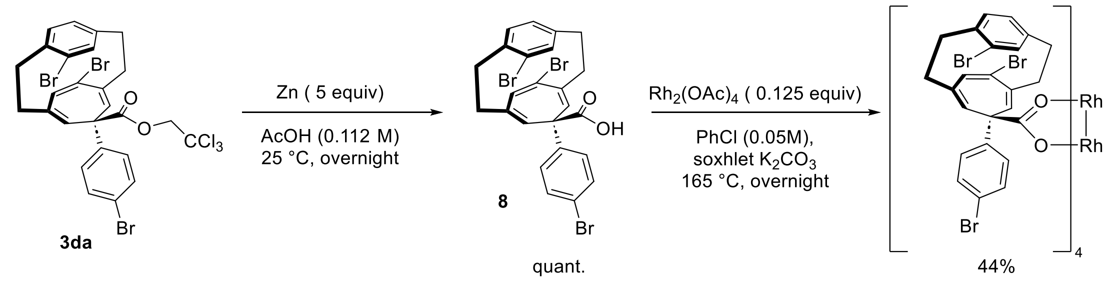

3da

8

quant.
ee = 95%
1st Recrystalization
98.7% ee (76% recover)

44%

9

8

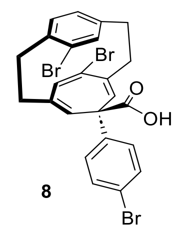

To a flame dry 16 ml vial, was charged with **3da** (500.0 mg, 703.7 μmol, 1 equiv), zinc (230.0 mg, 3.519 mmol, 5 equiv), then acetic acid (6.283 mL). The mixture was stirred at room temperature for 18 h. Then, it was partitioned between EtOAc and H_2O. The aqueous phase was extracted with 3 times EtOAc. The combined organic phase was then washed 3 times with H2O 25 ml, then brine 30 ml, and then dried with Na_2SO_4. The EtOAc solution was filtered and concentrated under vacuum. Further purification was achieved with flash column chromatography using [0-10% EA in DCM] to afford **8** as a white solid (414 mg, quantitative yield, 95% ee). Recrystallization was carried out in hot heptane to give **8** (311.1 mg, 76%, 99% ee).

R_f (9DCM/1EA) = 0.40

^{1}H NMR (600 MHz, $CDCl_3$) δ 7.20 (dd, *J* = 7.7, 1.7 Hz, 1H), 7.16 (d, *J* = 8.7 Hz, 2H), 6.92 (d, *J* = 8.7 Hz, 2H), 6.86 (d, *J* = 7.7 Hz, 1H), 6.74 (dd, *J* = 1.7, 0.8 Hz, 1H), 6.31 (s, 1H), 5.69 (s, 1H), 5.65 (s, 1H), 3.31 (ddd, *J* = 13.4, 10.0, 5.6 Hz, 1H), 3.24 – 3.17 (m, 1H), 3.06 – 2.91 (m, 3H), 2.83 – 2.73 (m, 2H), 2.39 – 2.29 (m, 1H).

^{13}C NMR (151 MHz, $CDCl_3$) δ 178.7, 140.8, 138.5, 138.2, 138.1, 136.6, 135.8, 134.6, 131.0, 130.2, 129.2, 128.2, 127.8, 127.1, 125.9, 121.3, 55.4, 36.9, 35.3, 32.3, 31.3. (missing 1 carbon)

HRMS (+p APCI) calcd. for [$C_{24}H_{20}O_2{}^{79}Br_3$] ([M+H]$^+$) 576.9008 found 576.9016
SFC: (ChiralCel OJ3, 10% (50% methanol in isopropanol with 0.2% Formic Acid), 2.5 mL/min, 1.0 mg/ml, 15 min, λ = 230 nm) retention times of 3.46 (minor) and 7.74 min (major) 99% ee (after recrystallization).

$[\alpha]^{20}_D$: -37.7° (c = 0.31 g/100 ml, $CHCl_3$, 99% ee)

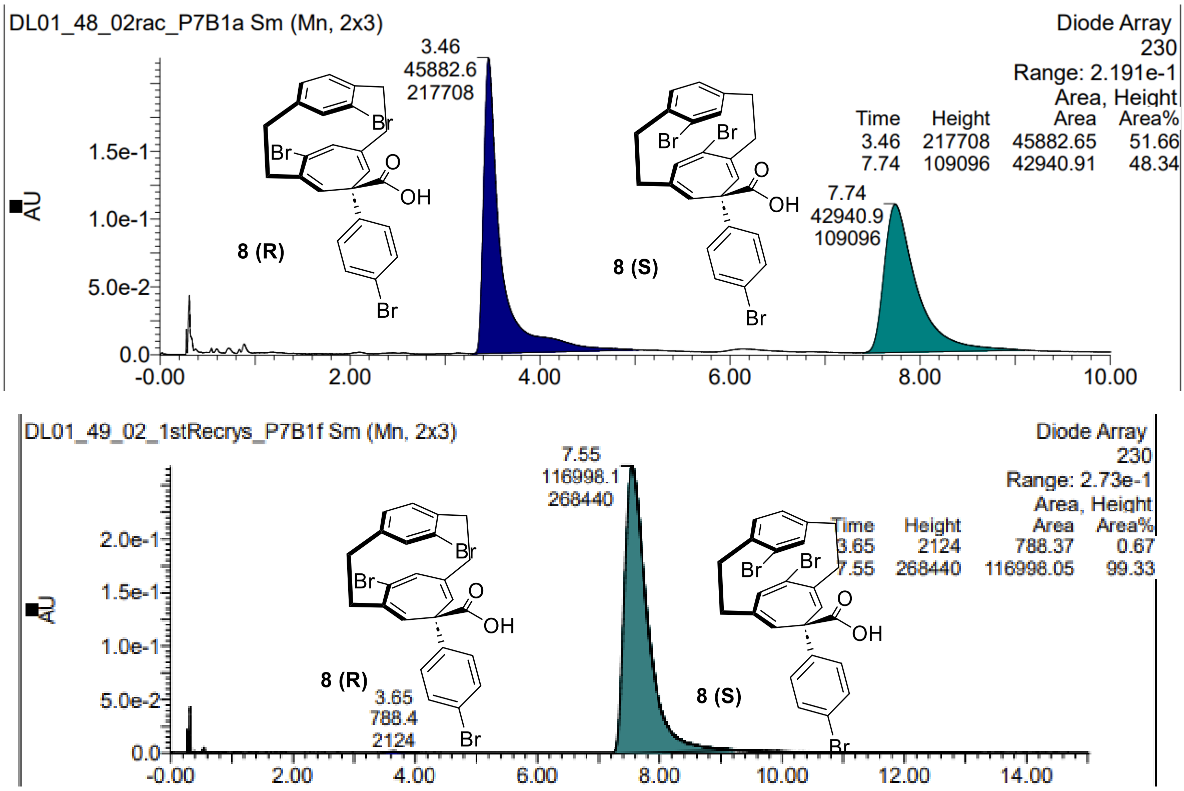

Figure S32. SFC trace for **8** (racemic – top /chiral – bottom)

Dirhodium tetracarboxylate 9

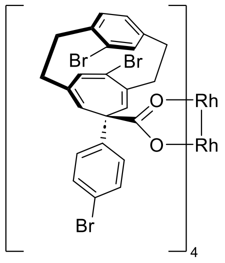

9

To a 25 ml round-bottom-flask equipped with a magnetic stir-bar was added **8** (232 mg, 400 μmol, 8 equiv) and dirhodium tetraacetate (22.1 mg, 0.0500 mmol, 1 equiv). Chlorobenzene (12.0 mL) was added. The flask was fitted with a Soxhlet packed with K_2CO_3 anhydrous. The Soxhlet was also fitted with a condenser. The reaction mixture was set to stir overnight at reflux (150-170 °C) under an inert atmosphere. The solvent was removed under vacuum. The crude green material was then dry-loaded on silica and purified by silica-gel-chromatography on a gradient (0-11% ethyl acetate in hexane) to afford **9** as a green solid (54.3 mg, 44%)

R_f (6H/1EA) = 0.40

^{1}H NMR (600 MHz, C_6D_6) δ 7.32 (d, J = 8.3 Hz, 2H), 6.98 (s, 1H), 6.81 (d, J = 8.1 Hz, 2H), 6.48 (d, J = 7.7 Hz, 1H), 6.18 (s, 1H), 5.47 (s, 1H), 5.20 (s, 1H), 3.40 – 3.21 (m, 2H), 2.97 (ddd, J = 13.9, 10.1, 3.6 Hz, 1H), 2.81 (t, J = 7.3 Hz, 2H), 2.64 (dt, J = 14.6, 8.6 Hz, 1H), 2.45 (ddd, J = 14.0, 10.5, 3.7 Hz, 1H), 2.10 (ddd, J = 14.5, 10.4, 5.2 Hz, 1H).

^{13}C NMR (151 MHz, C_6D_6) δ 193.6, 141.3, 140.2, 138.5, 138.0, 137.2, 135.5, 133.9, 131.2, 130.7, 130.0, 129.9, 129.8, 129.1, 126.6, 121.0, 57.9, 37.6, 35.8, 32.6, 31.3. (missing 1 carbon)

HRMS (p ESI) calcd. for [$C_{96}H_{72}O_8{}^{79}Br_9{}^{81}Br_3{}^{103}Rh_2$] ([M]$^+$) 2511.3471 found 2511.3655 (Δ = 7.3 ppm)

11. Dirhodium activity

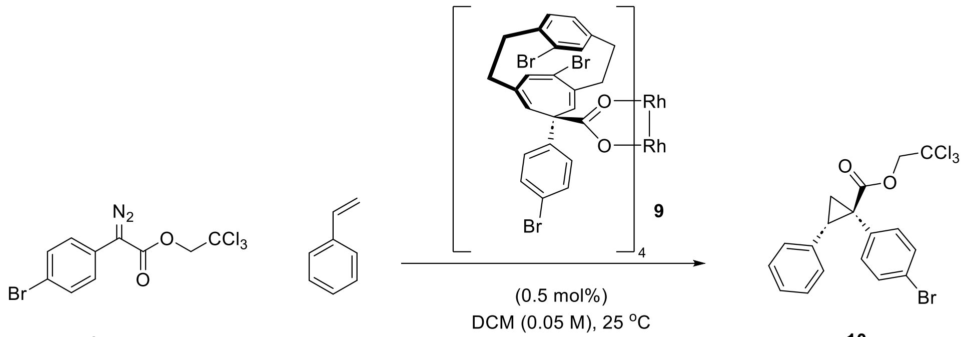

2a 5 equiv **10**
86%; 81% ee

To a flame dried 8 ml vial was charged with magnetic stir bar. **9** (1.26 mg, 0.5 μmol,0.005 equiv), styrene (57.3 μL, 52.1 mg, 0.5 mmol, 5 equiv), MS 4Å, and 1.0 ml DCM was introduced to the vial. A solution of 2,2,2-trichloroethyl 2-(4-bromophenyl)-2-diazoacetate (37.2 mg, 0.10 mmol, 1.0 equiv) in 1.0 ml of DCM was added dropwise in 5 mins. The solution was let stir further 10 min. The reaction mixture was then filtered and concentrated under reduced pressure. The crude reaction mixture was then purified by flash chromatography (gradient 0-10% ether in hexane) to afford **10** (38.6 mg, 86%) as a clear oil which solidifies upon high vacuum.

R_f (10% Et_2O in hexane) = 0.55

^{1}H NMR (400 MHz, $CDCl_3$) δ 7.26 (d, J = 8.5 Hz, 2H), 7.16 – 7.04 (m, 3H), 6.94 (d, J = 8.5 Hz, 2H), 6.84 – 6.75 (m, 2H), 4.83 (d, J = 11.9 Hz, 1H), 4.64 (d, J = 11.9 Hz, 1H), 3.22 (dd, J = 9.4, 7.5 Hz, 1H), 2.28 (dd, J = 9.4, 5.2 Hz, 1H), 1.97 (dd, J = 7.5, 5.2 Hz, 1H). (*the obtain NMR matched the reported literature*)[17]

HPLC The enantiopurity was determined to be 81% ee by chiral HPLC analysis (Chiracel AD-H, 1.0% IPA/Hexanes, 1.0 mL/min, λ=230 nm, retention time of 6.74 min (major) and 8.43 min (minor).

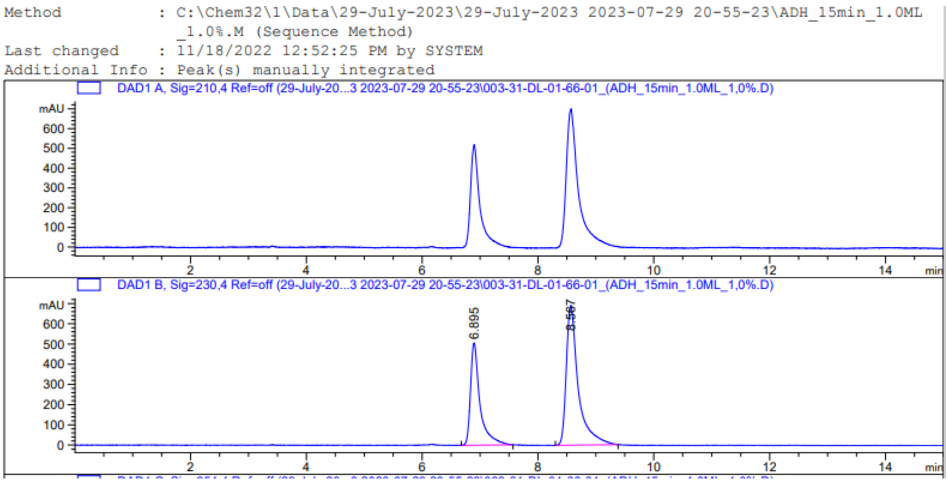

Signal 2: DAD1 B, Sig=230,4 Ref=off

Peak #	RetTime [min]	Type	Width [min]	Area [mAU*s]	Height [mAU]	Area %
1	6.895	BV R	0.1520	5739.66699	505.56860	36.5639
2	8.567	VV R	0.1776	9957.94824	691.02069	63.4361

Totals : 1.56976e4 1196.58929

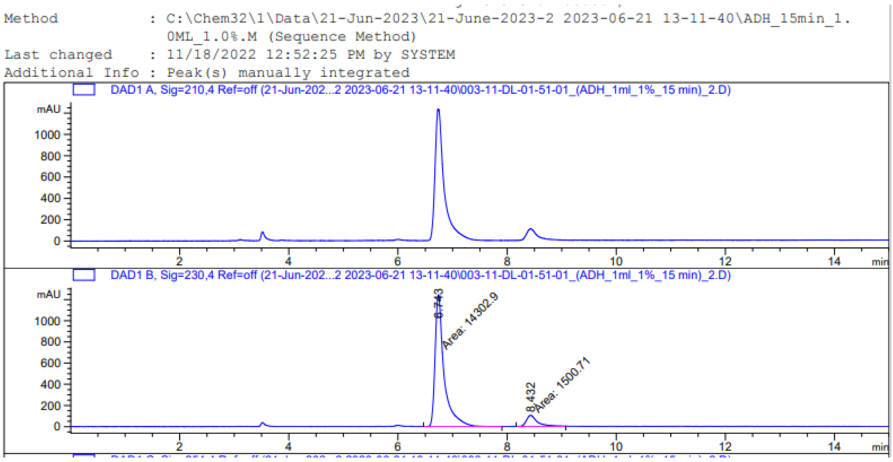

Signal 2: DAD1 B, Sig=230,4 Ref=off

Peak #	RetTime [min]	Type	Width [min]	Area [mAU*s]	Height [mAU]	Area %
1	6.743	MM	0.1912	1.43029e4	1247.06909	90.5040
2	8.432	MM	0.2364	1500.71448	105.79115	9.4960

Totals : 1.58037e4 1352.86024

Figure S33. SFC trace for **10** (racemic – top /chiral – bottom)

12. Copies of NMR spectra

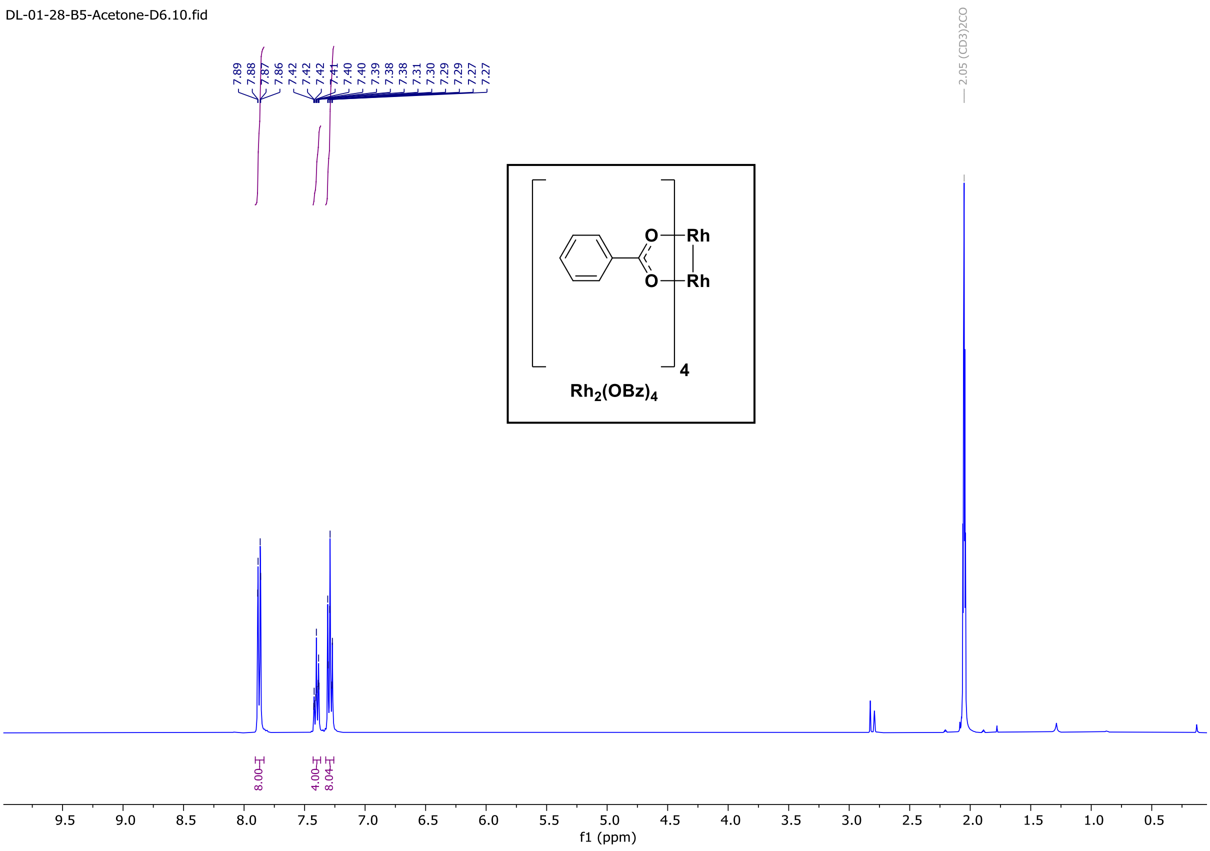

Figure S34. ^{1}H-NMR of $Rh_2(OBz)_4$

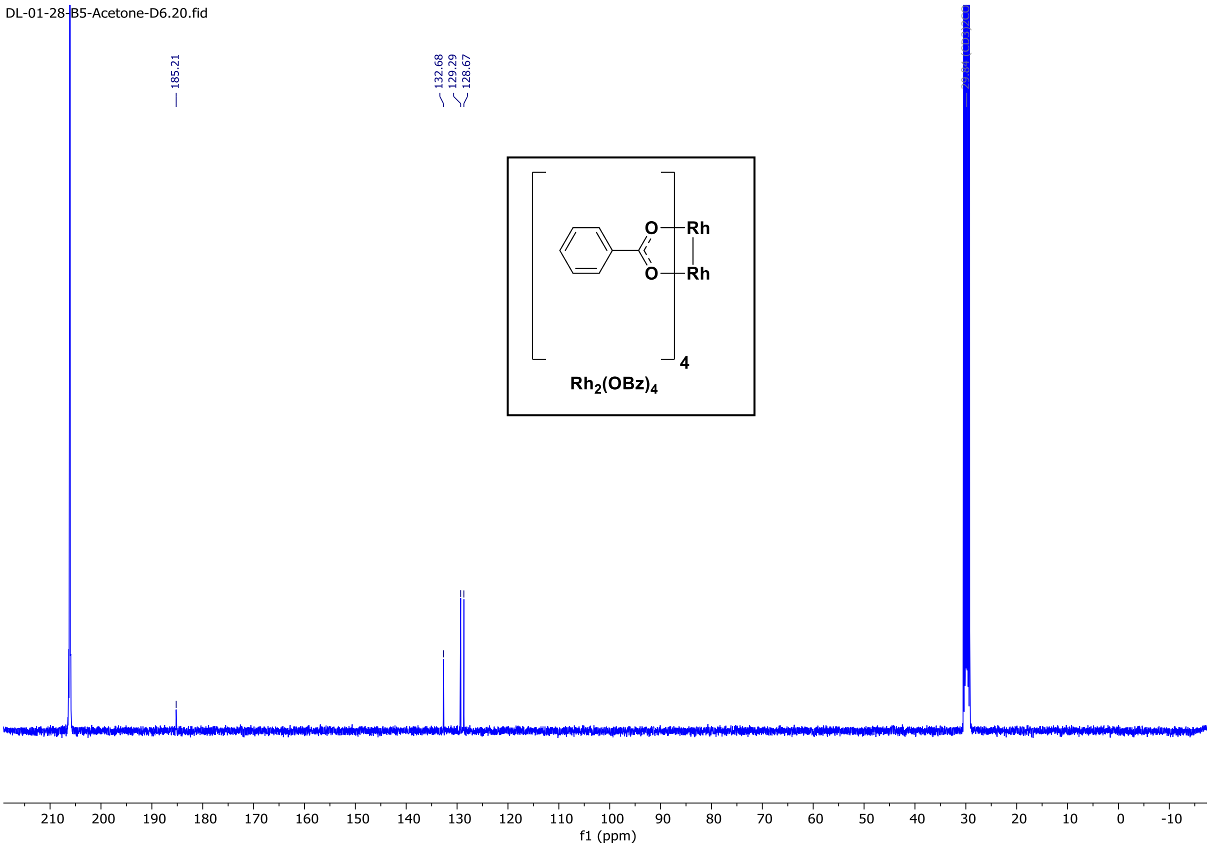

Figure S35. ^{13}C-NMR of $Rh_2(OBz)_4$

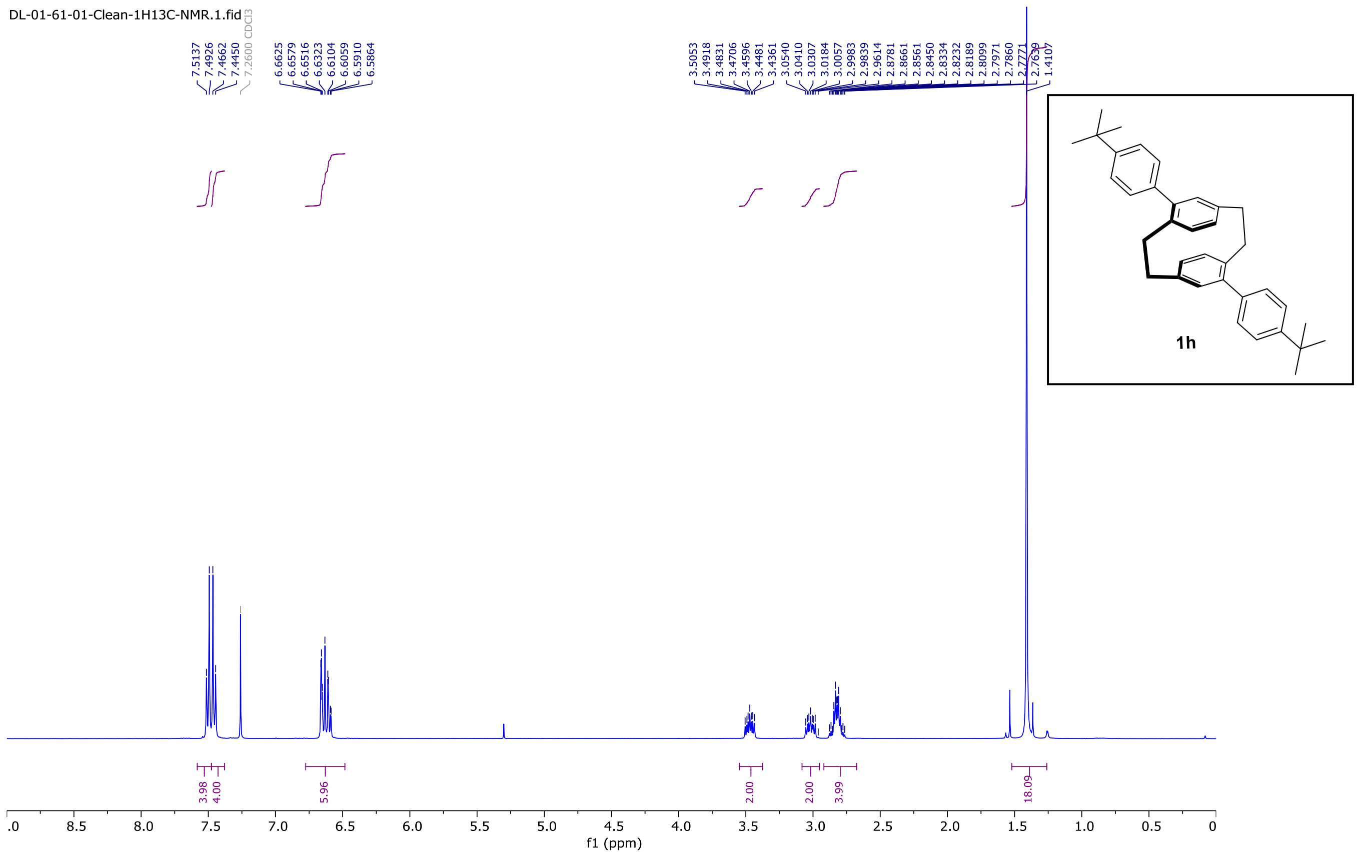

Figure S36. **^{1}H-NMR of 1h**

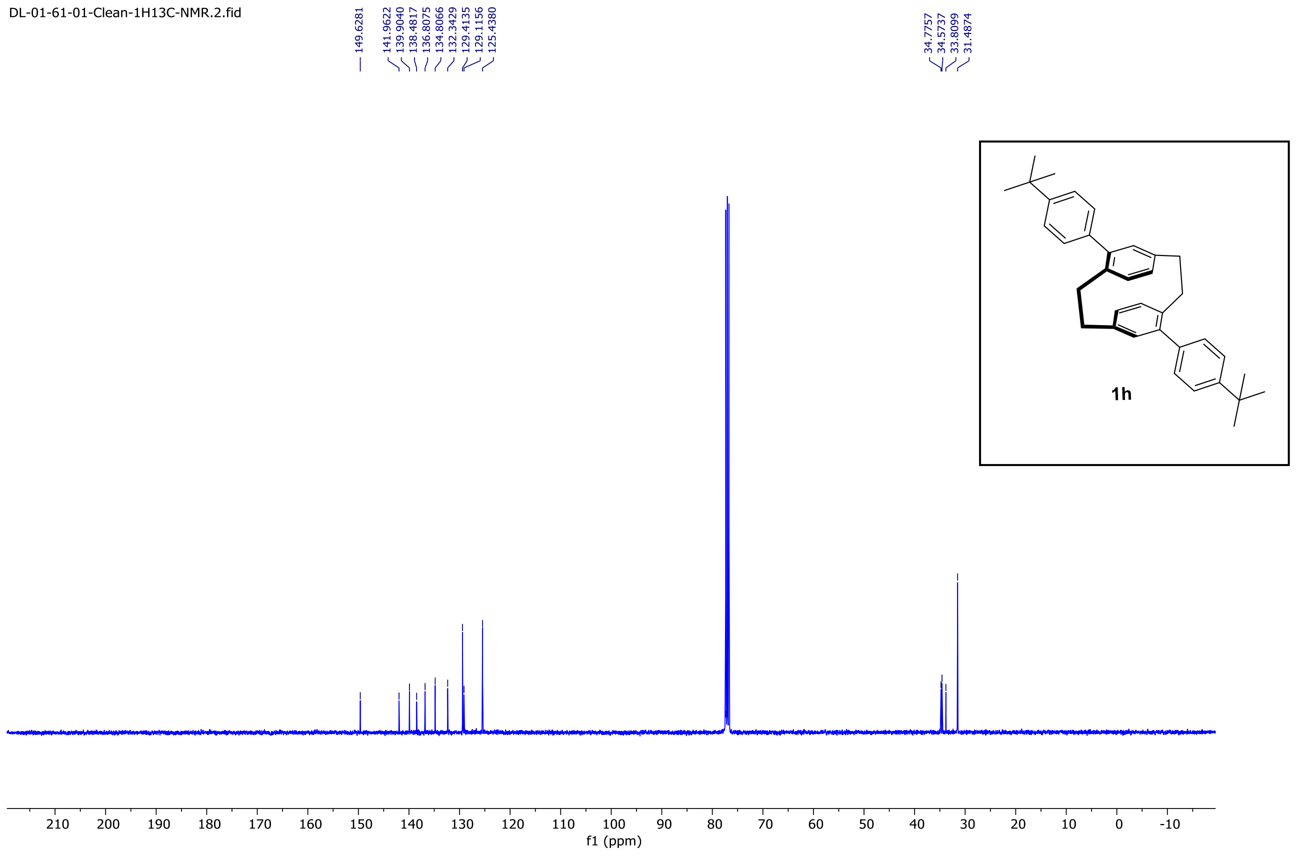

Figure S37. **^{13}C-NMR of 1h**

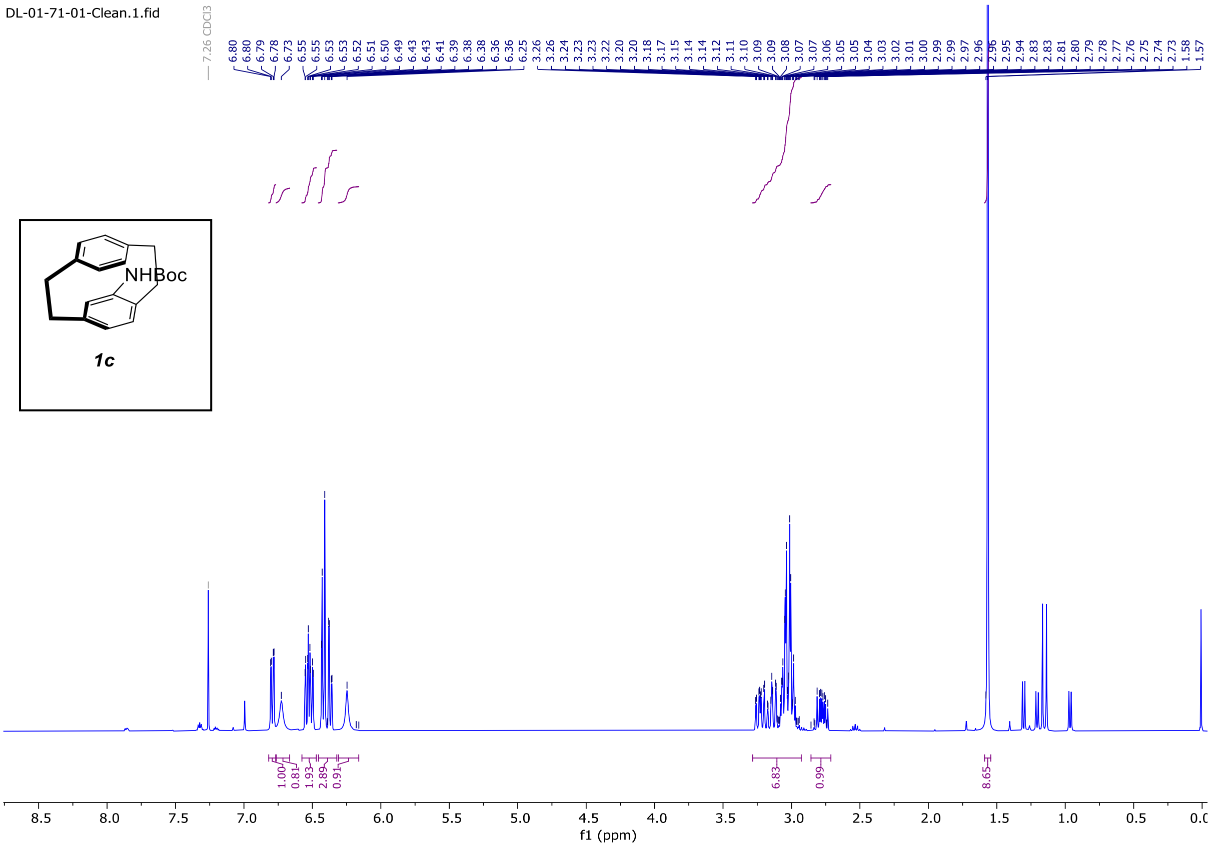

Figure S38. ^{1}**H-NMR of 1c**

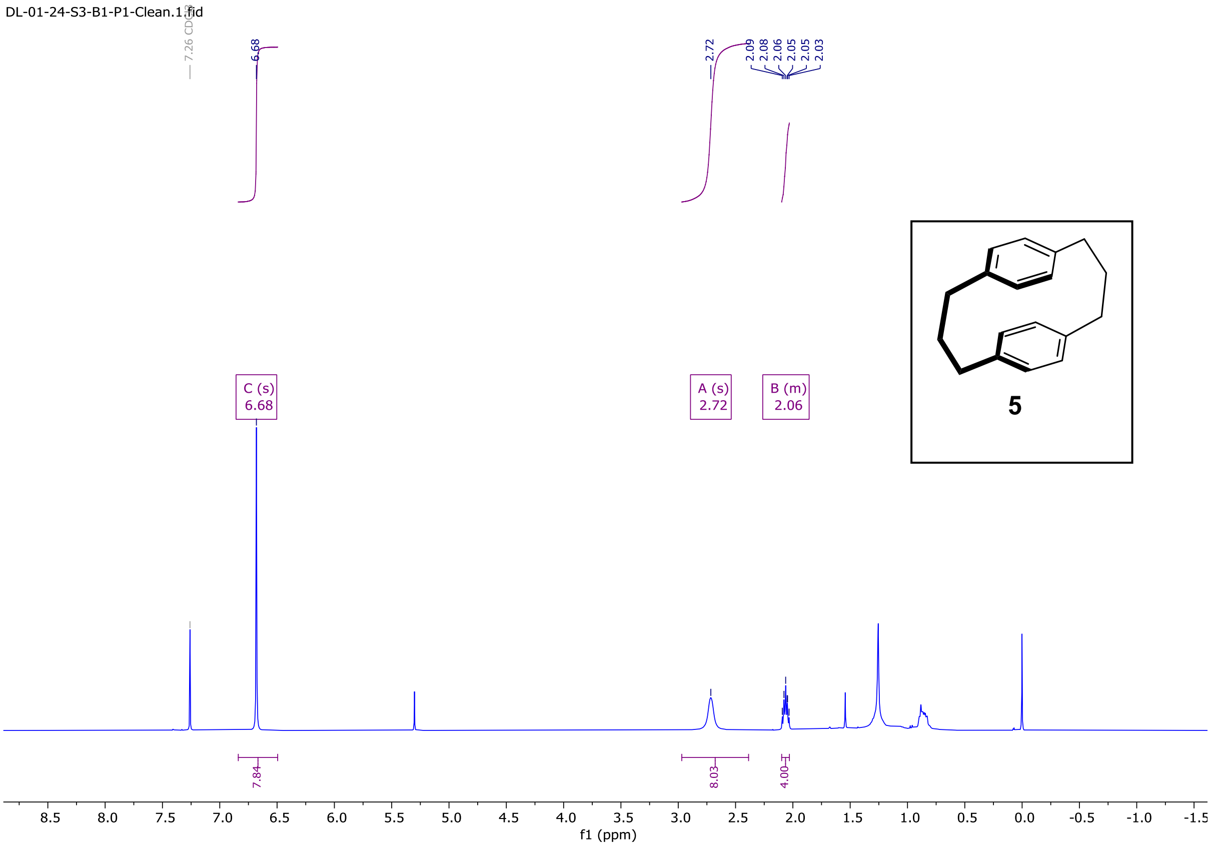

Figure S39. ^{1}**H-NMR of 5**

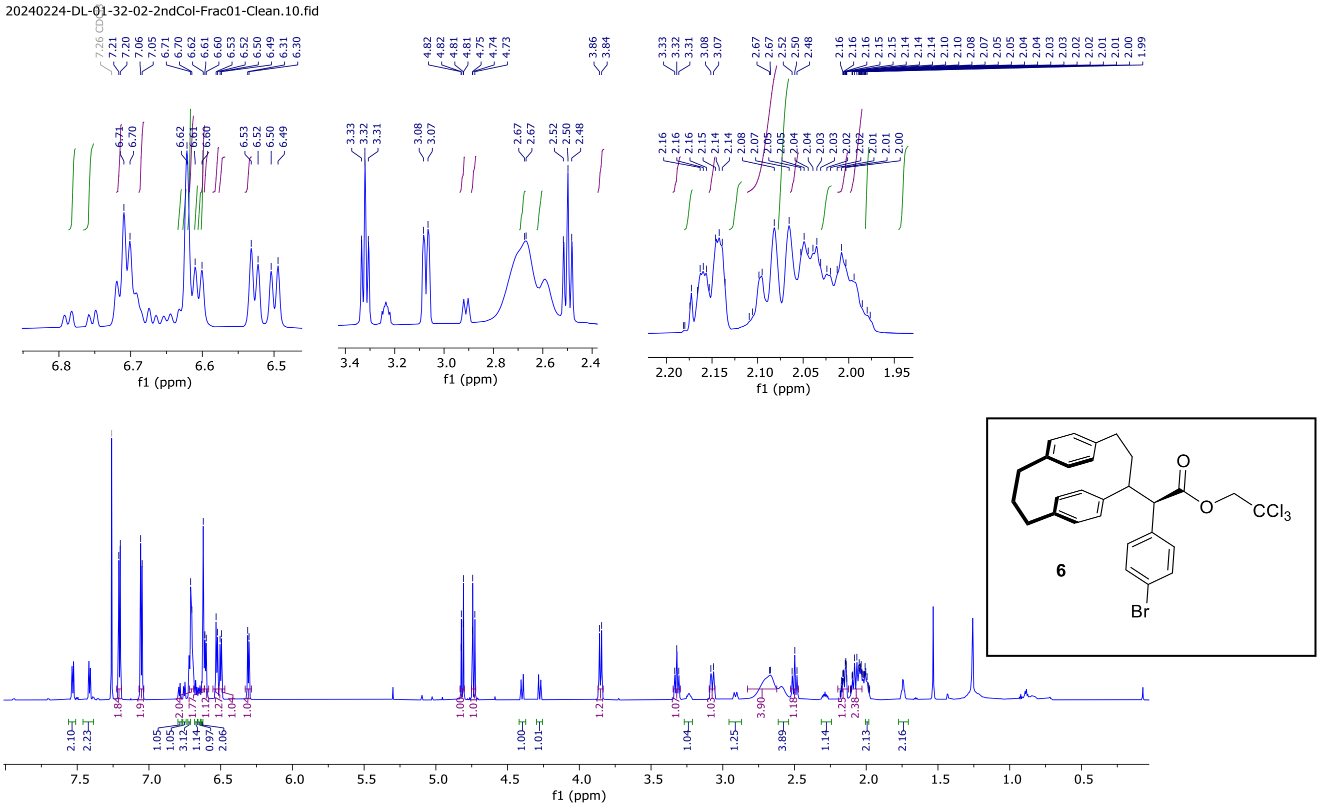

Figure S40. ^{1}H-NMR of 6

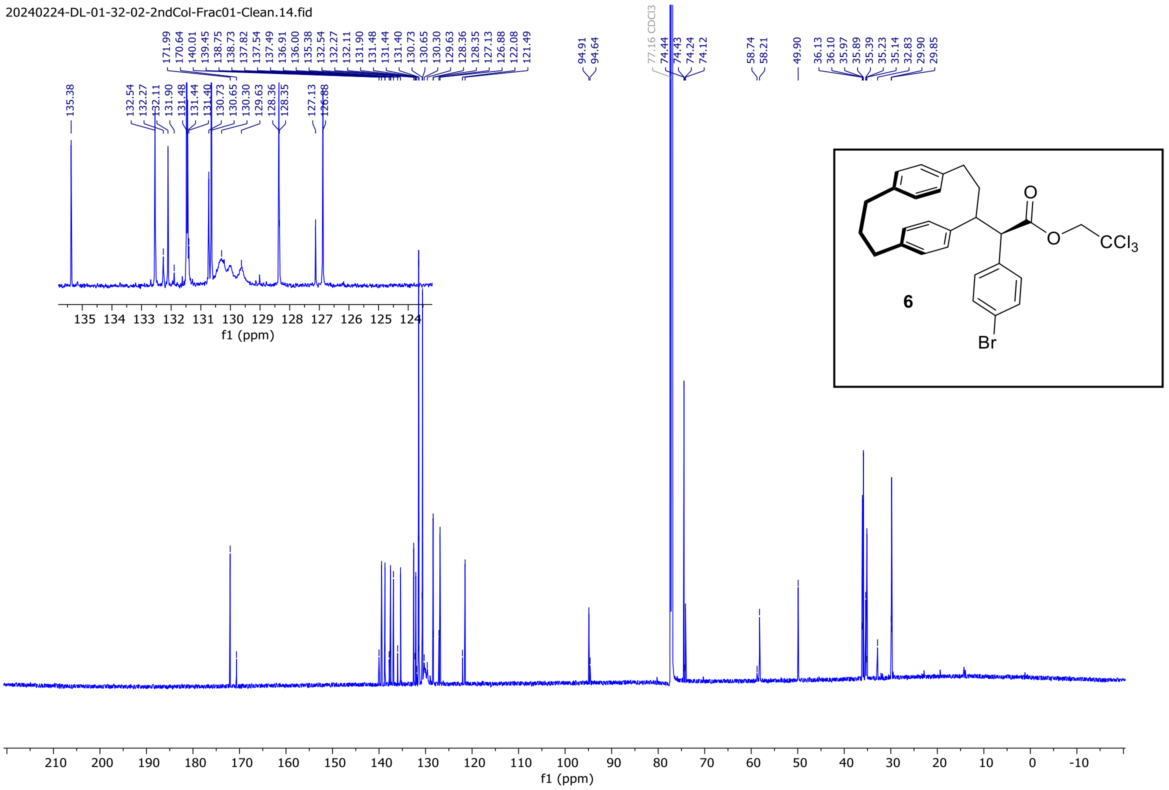

Figure S41. ^{13}C-NMR of 6

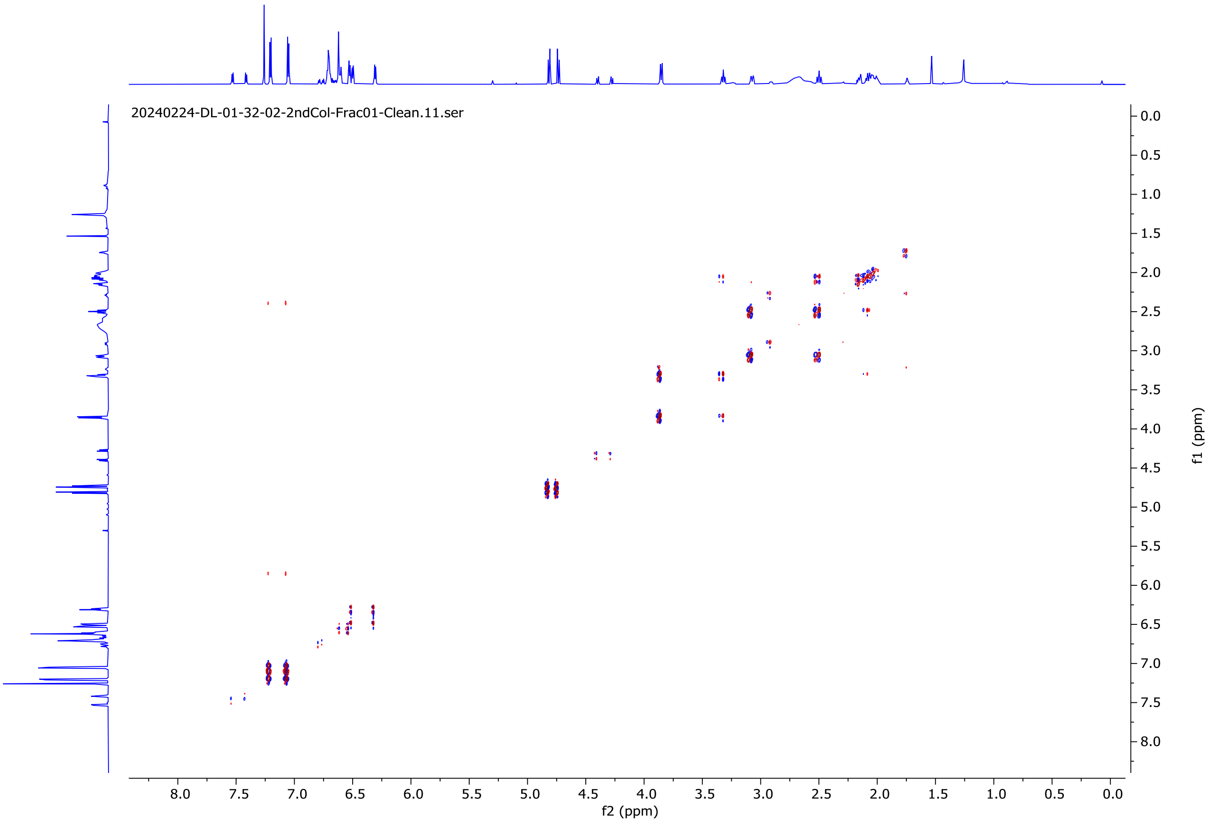

Figure S42. COSEY of 6

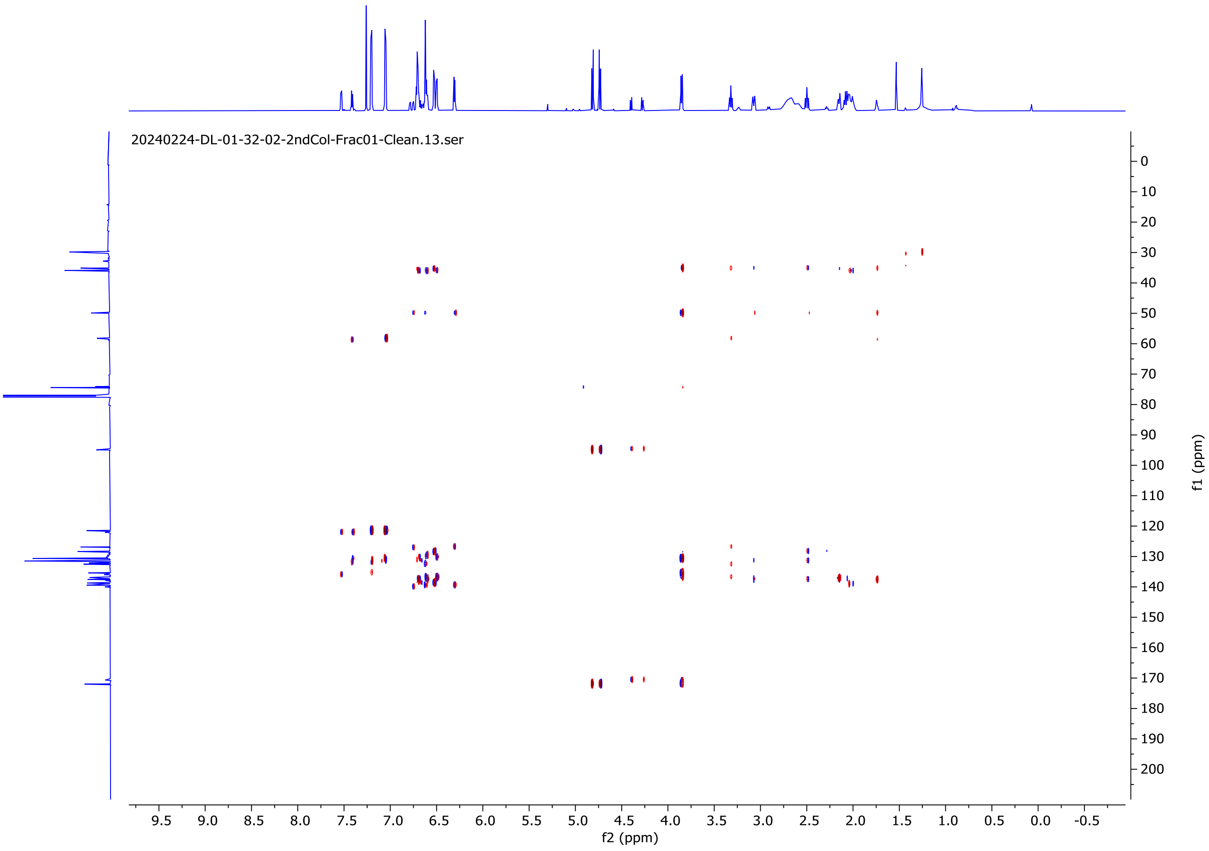

Figure S43. HMBC of 6

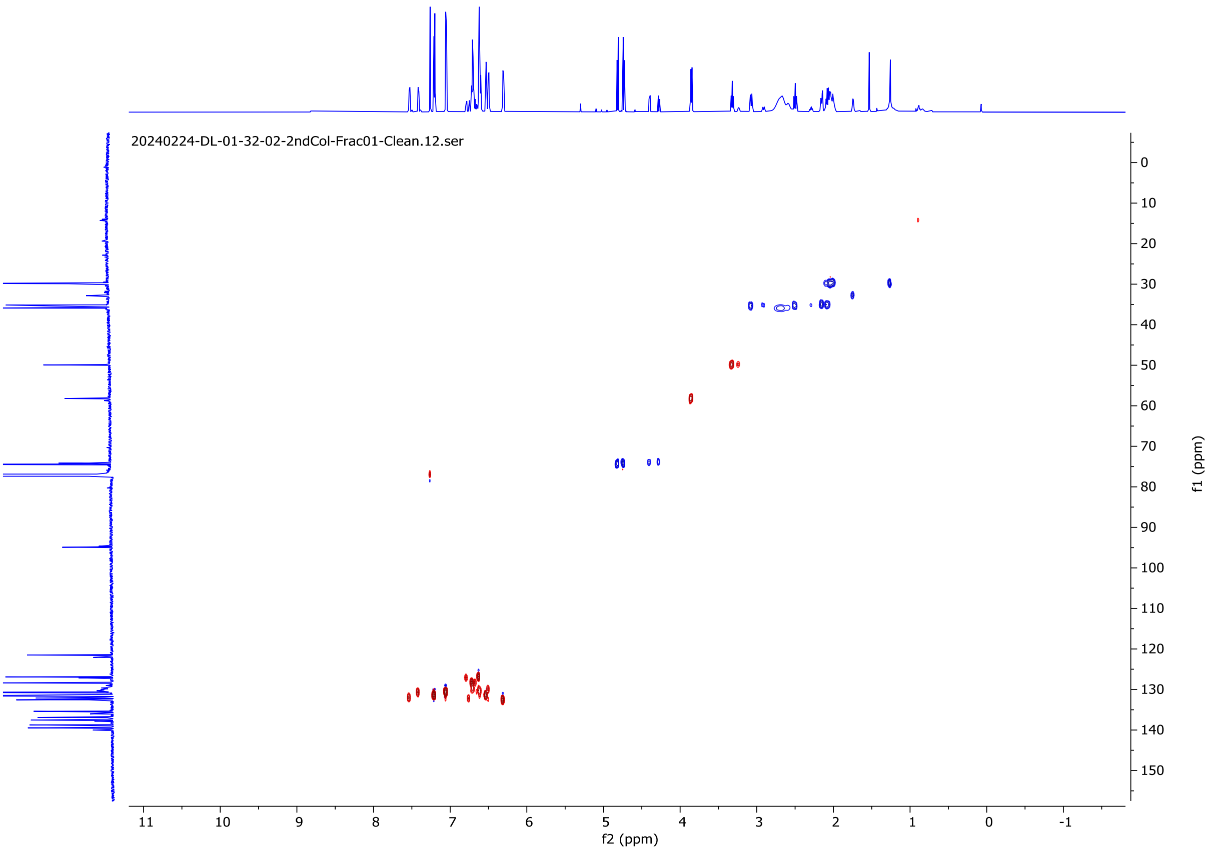

Figure S44. HSQC of 6

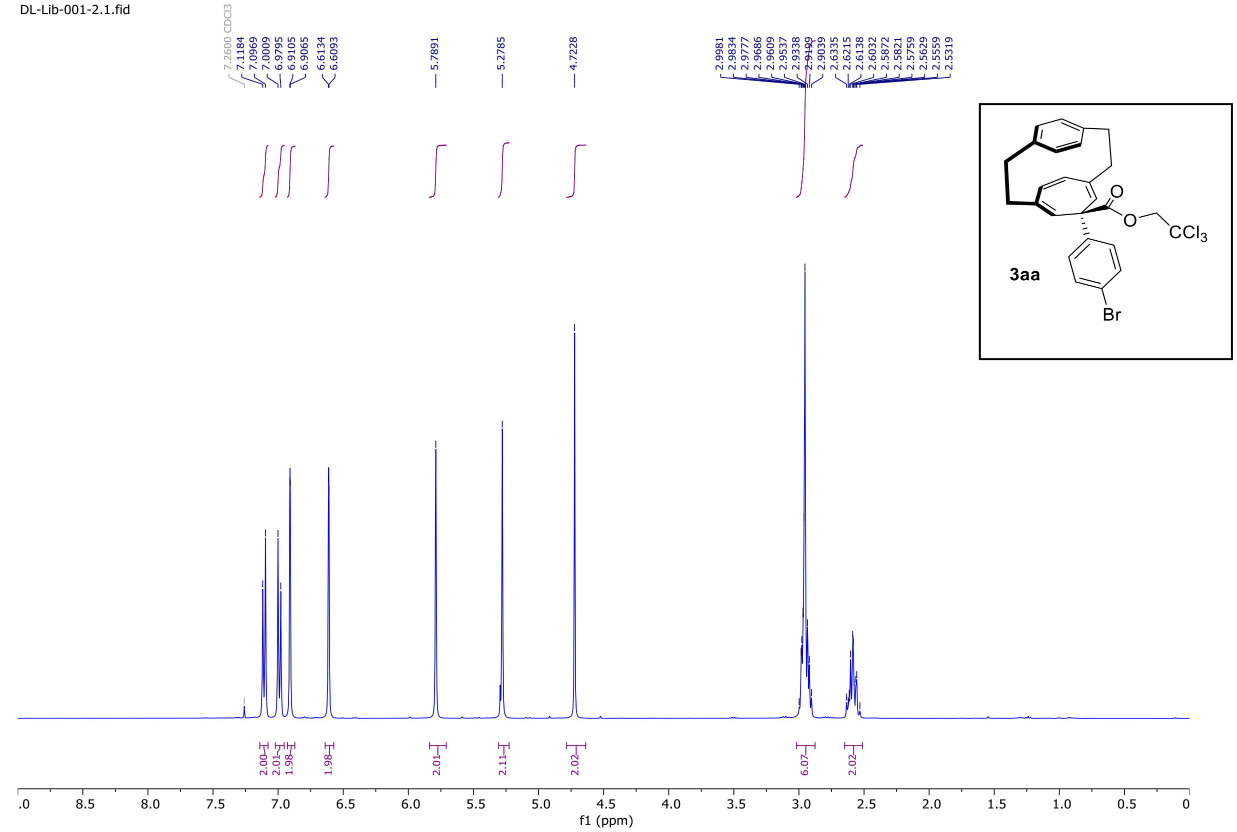

Figure S45. ^{1}H-NMR of 3aa

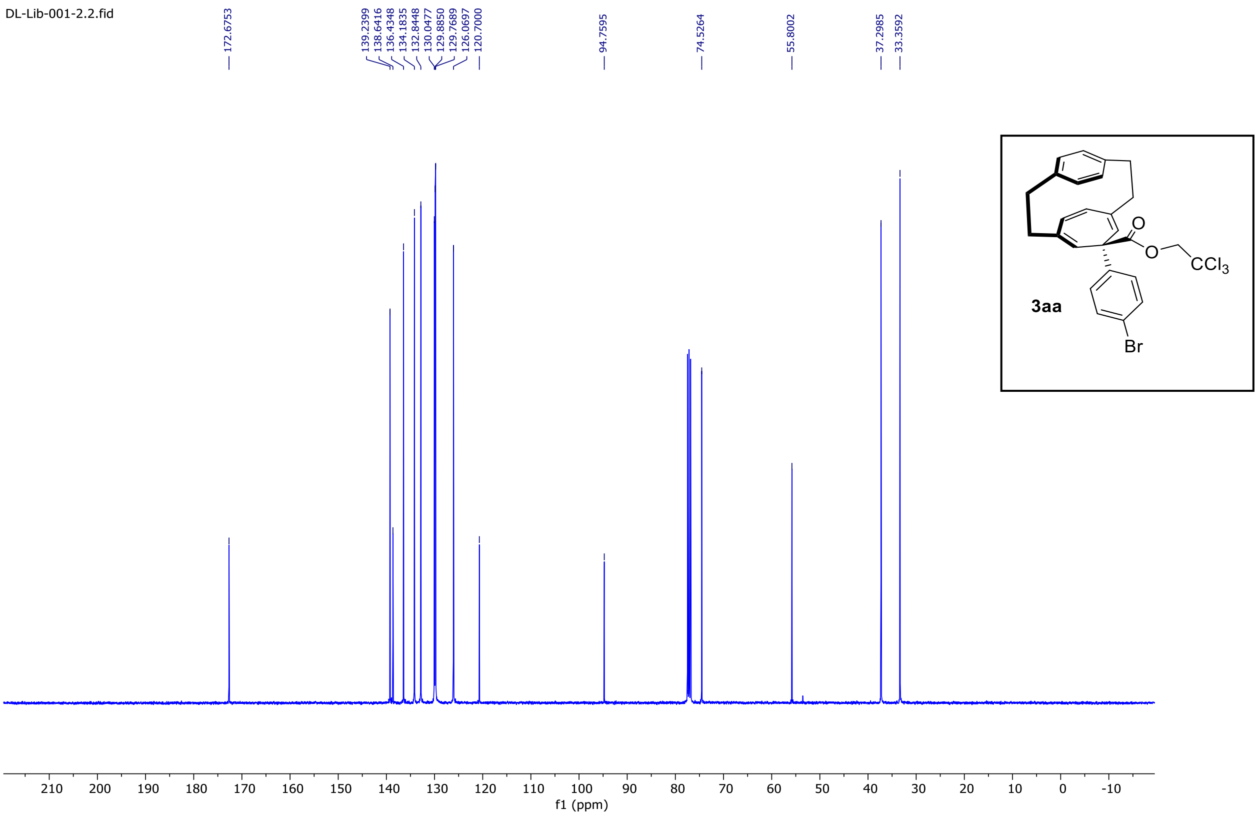

Figure S46. ^{13}C-NMR of 3aa

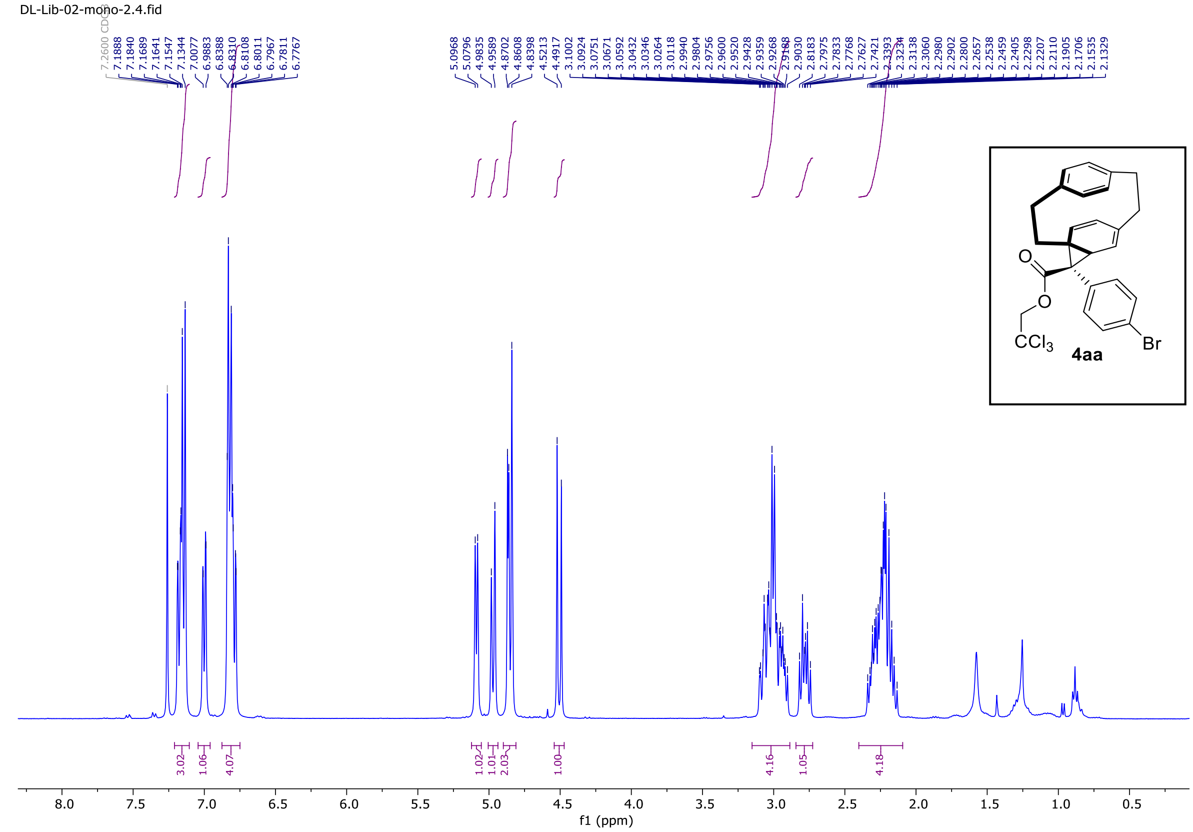

Figure S47. ^{1}H-NMR of 4aa

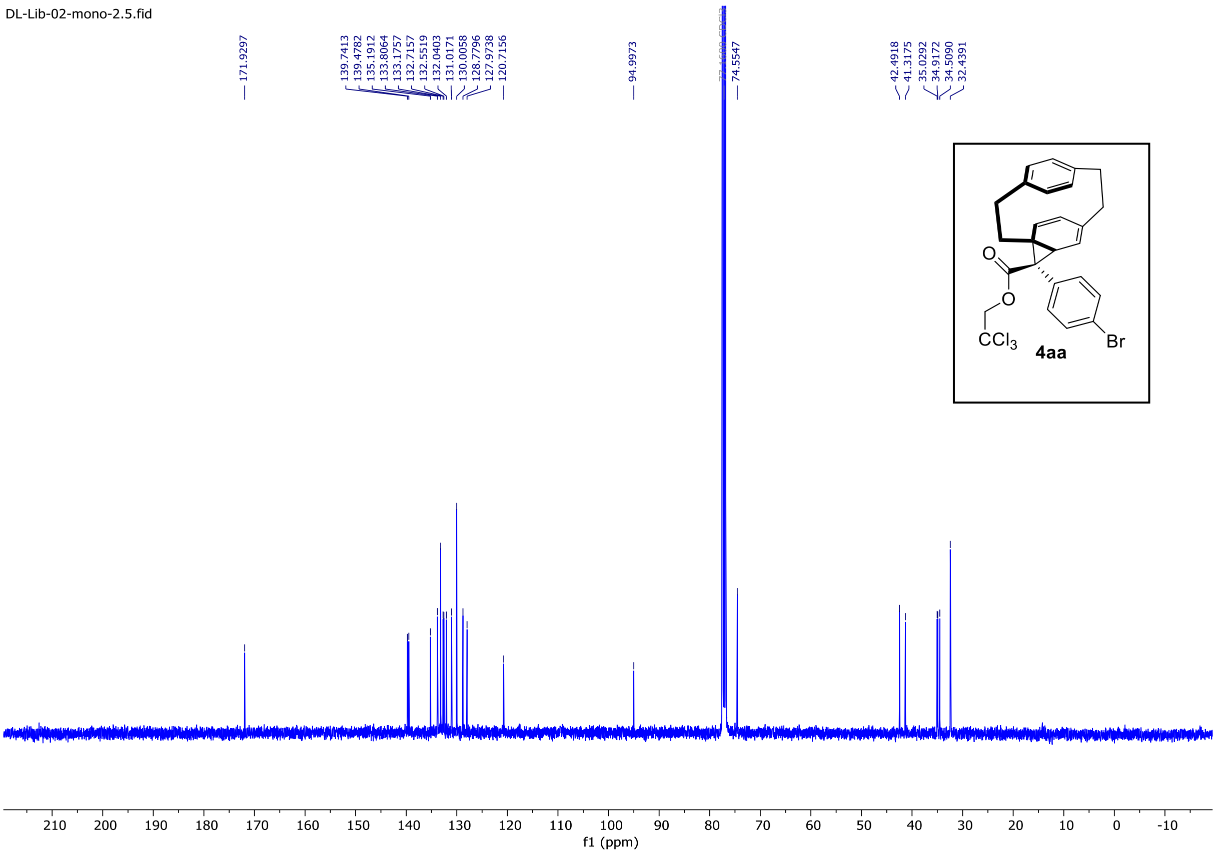

Figure S48. ^{13}C-NMR of 4aa

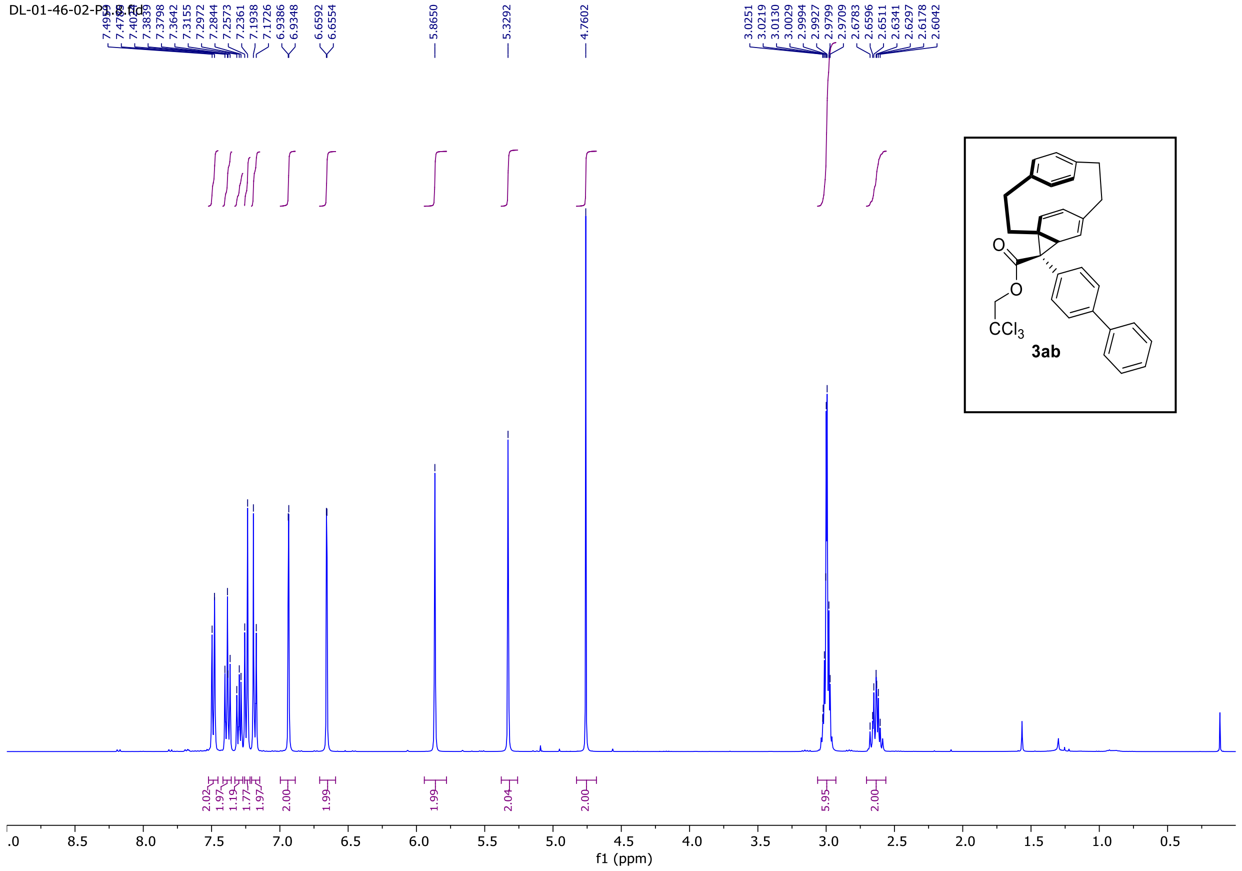

Figure S49. ^{1}**H-NMR of 3ab**

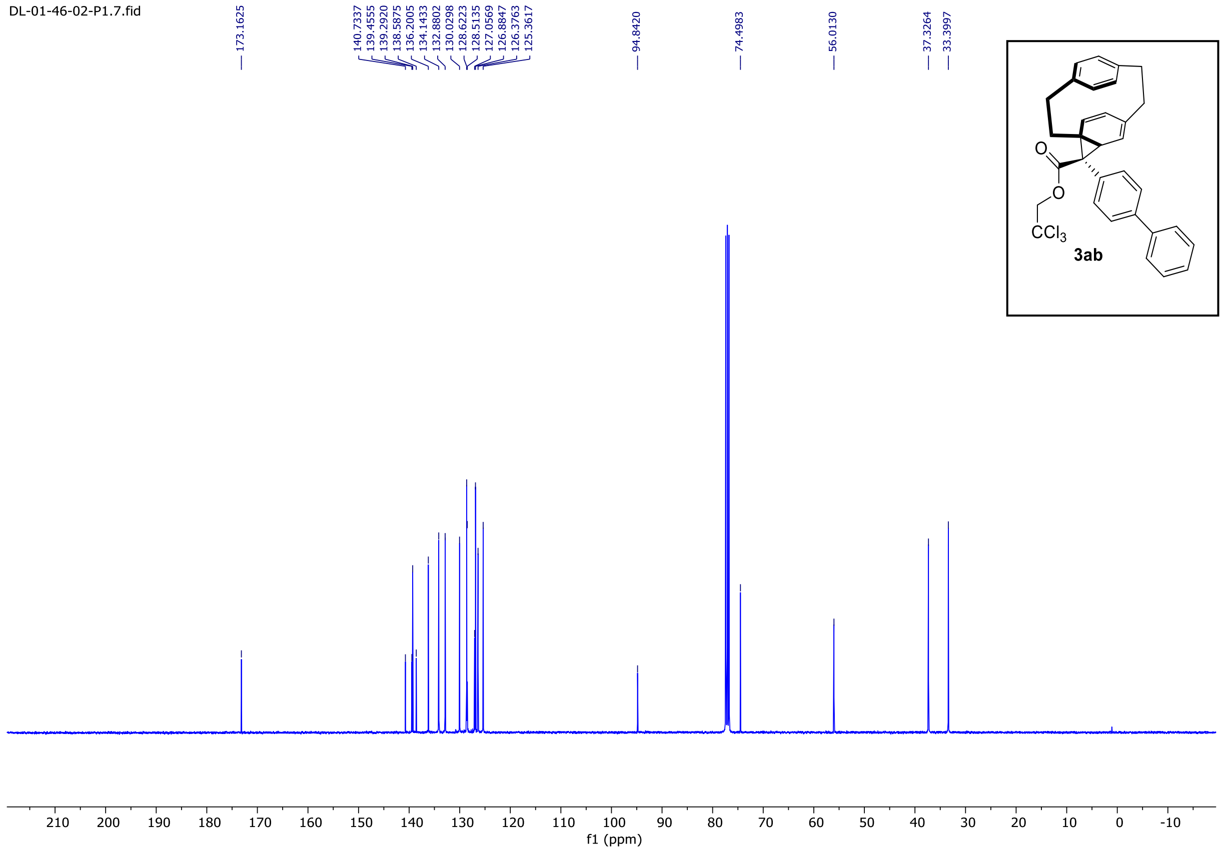

Figure S50. ^{13}**C-NMR of 3ab**

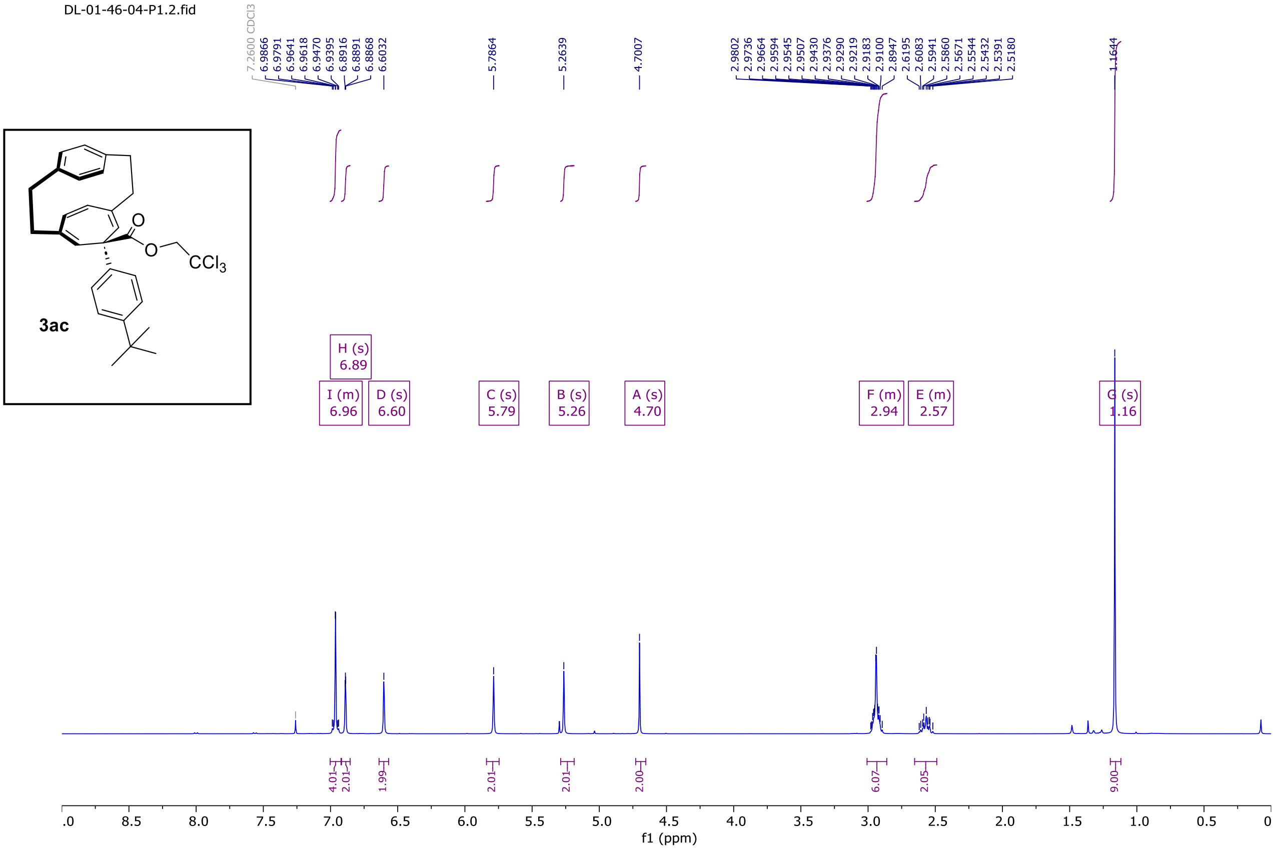

Figure S51. ^{1}H-NMR of 3ac

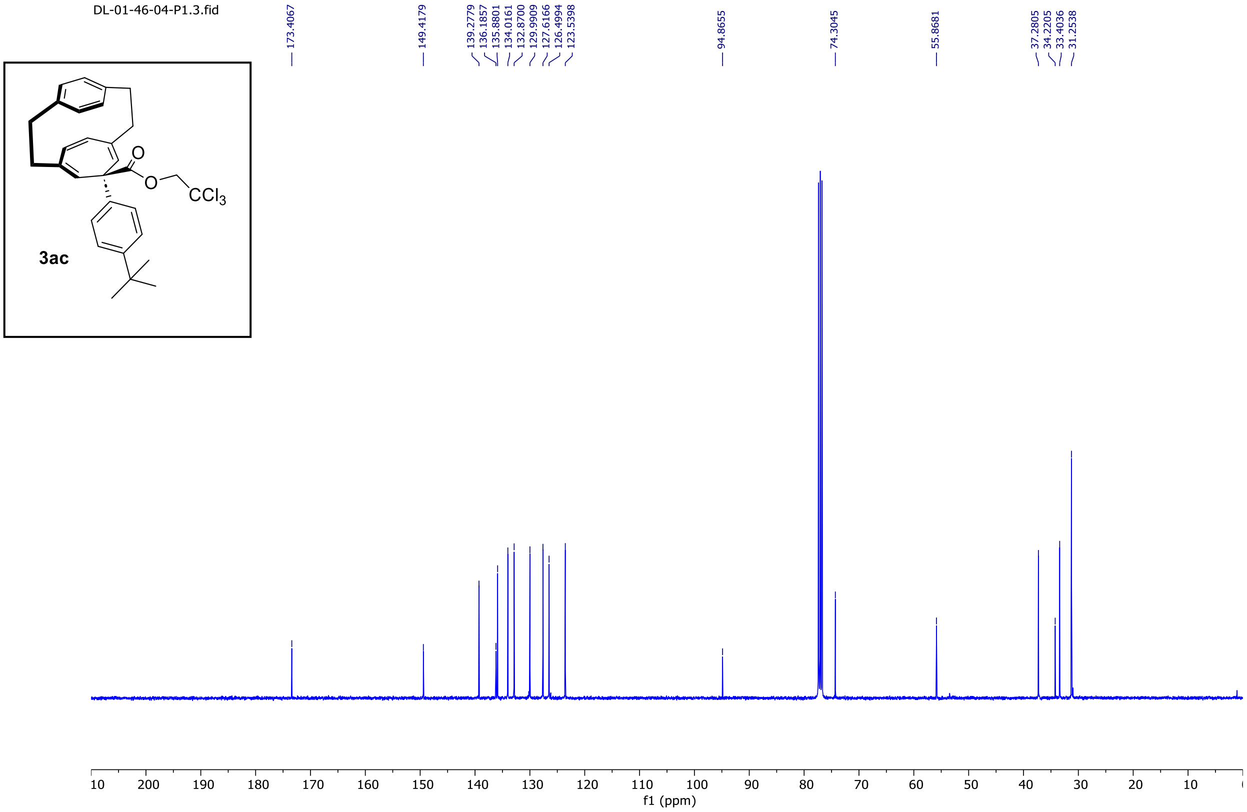

Figure S52. ^{13}C-NMR of 3ac

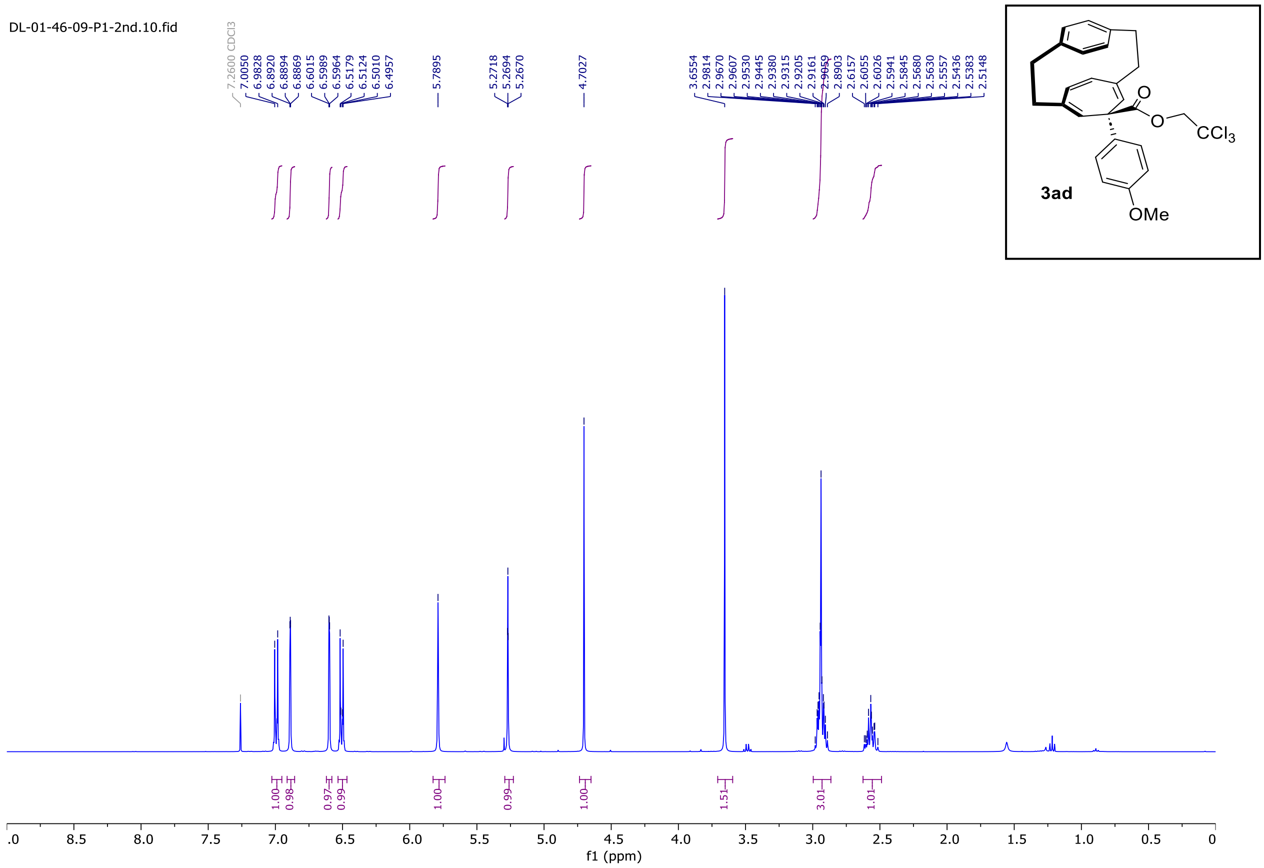

Figure S53. ^{1}H-NMR of 3ad

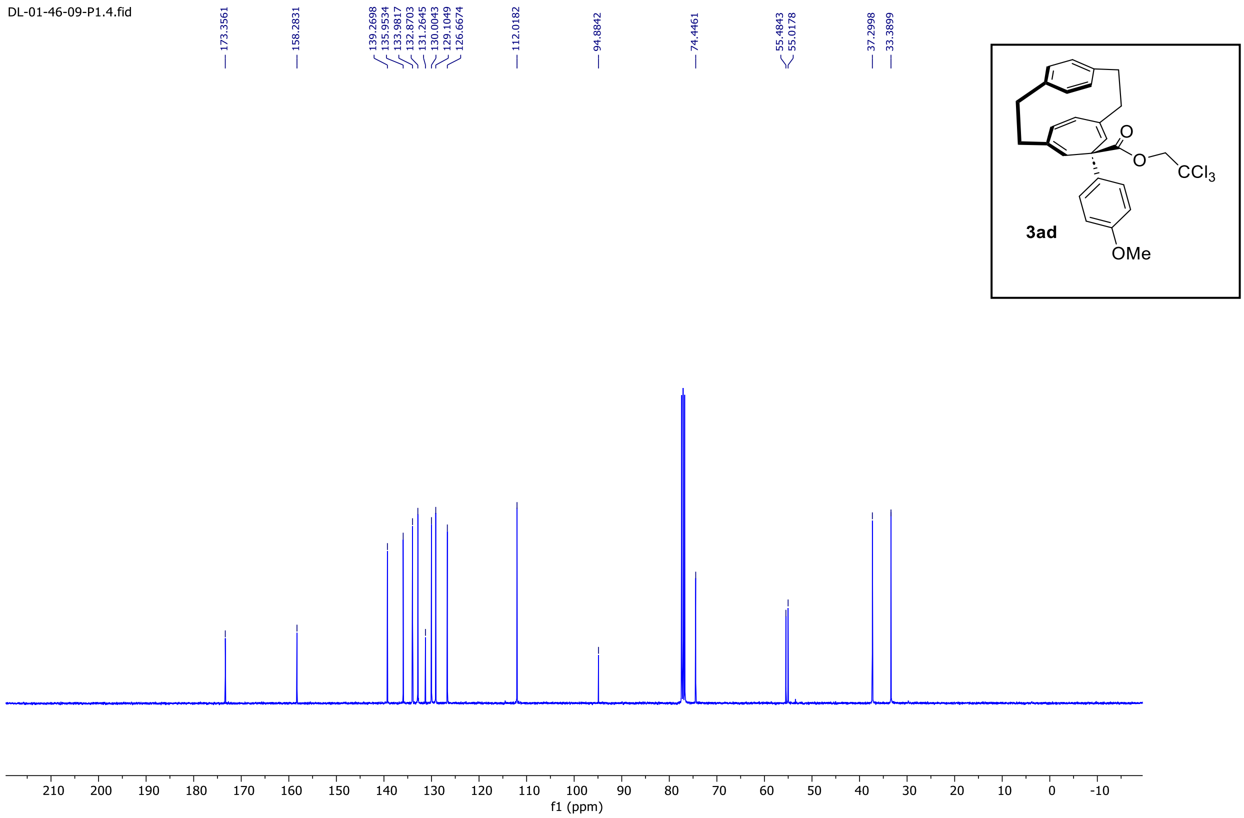

Figure S54. ^{13}C-NMR of 3ad

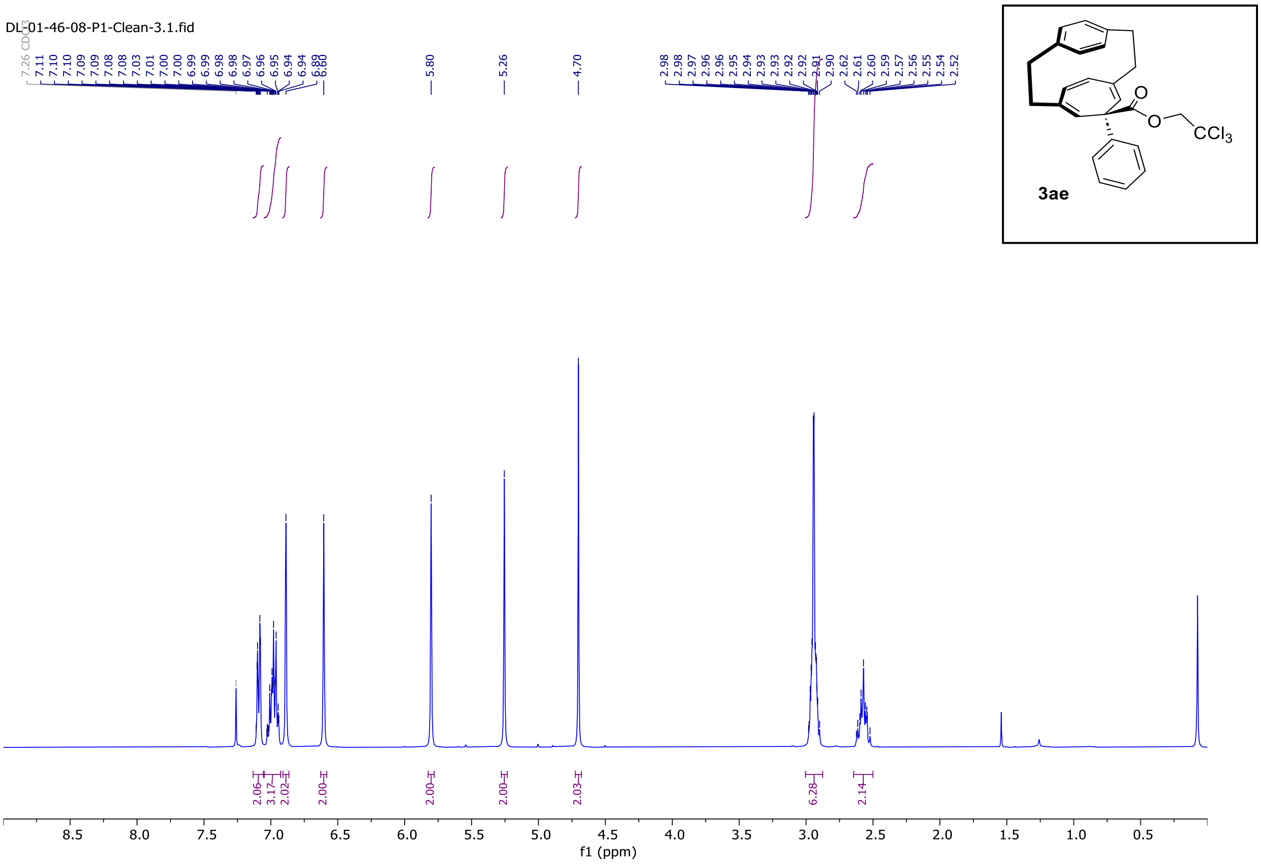

Figure S55. ^{1}**H-NMR of 3ae**

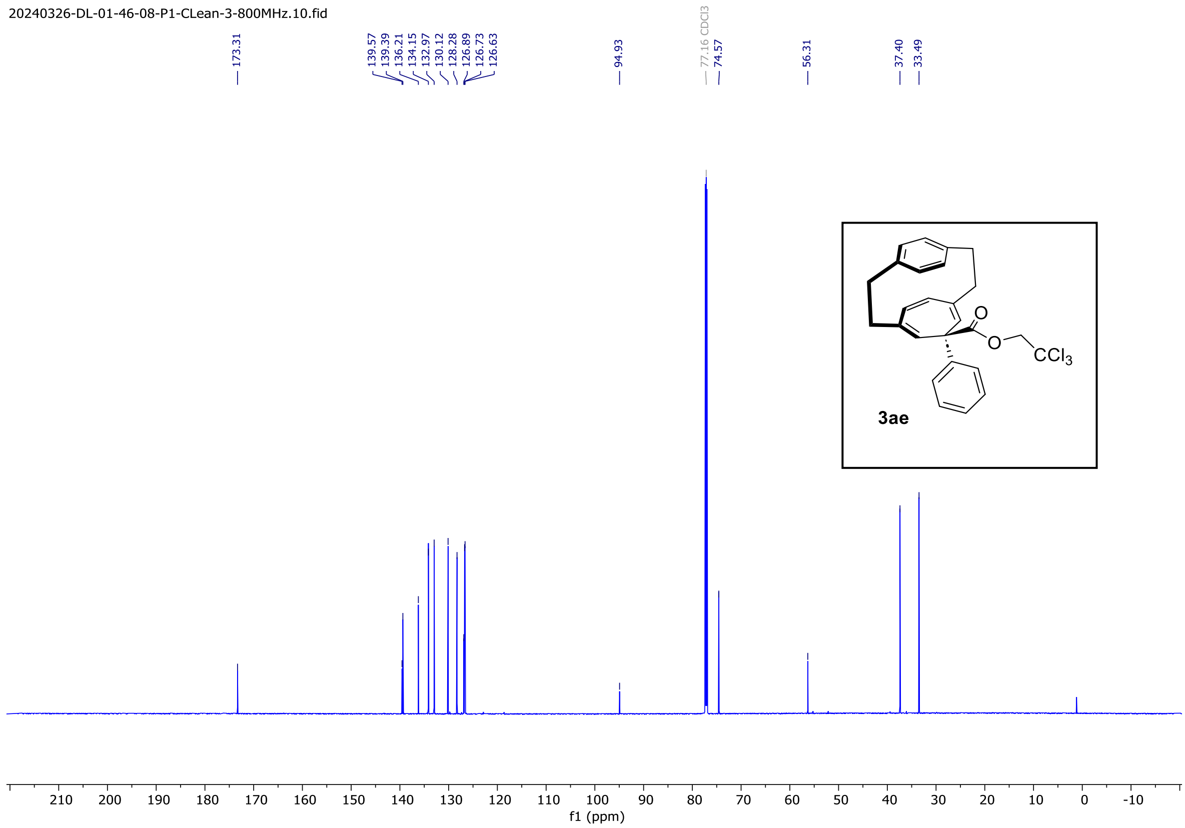

Figure S56. ^{13}**C-NMR of 3ae**

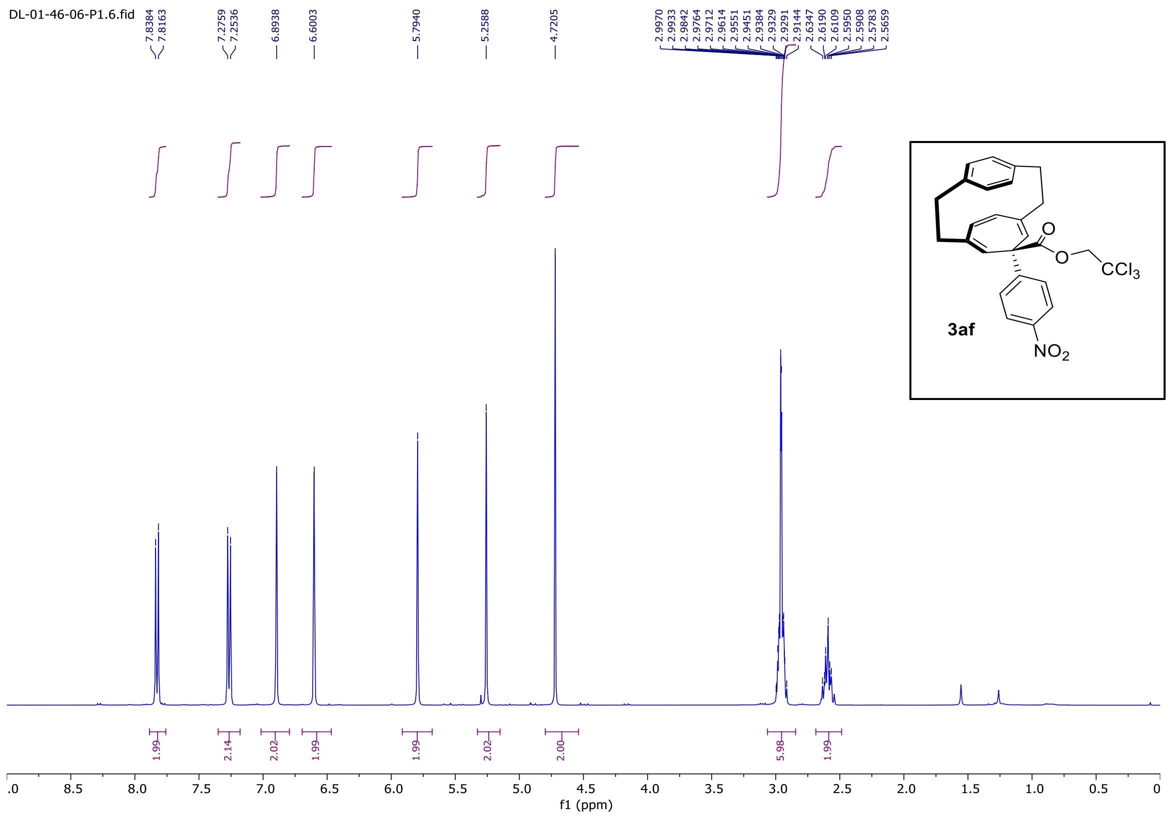

Figure S57. ^{1}H-NMR of 3af

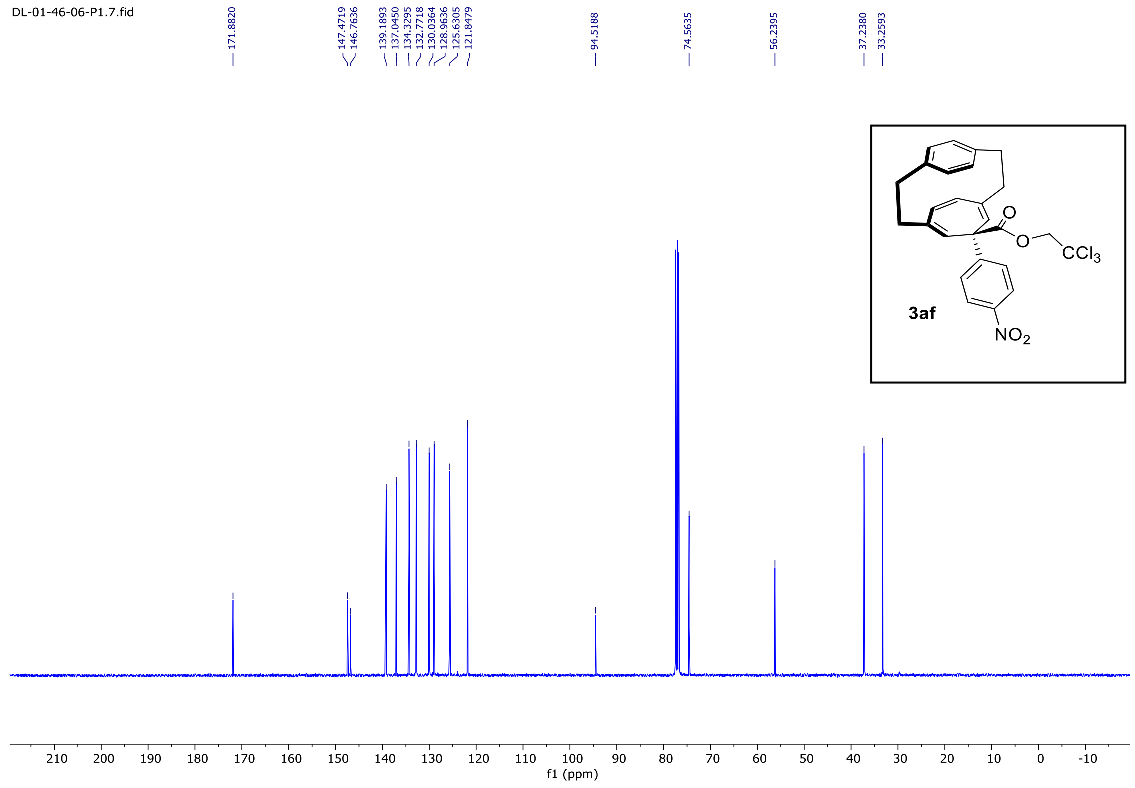

Figure S58. ^{13}C-NMR of 3af

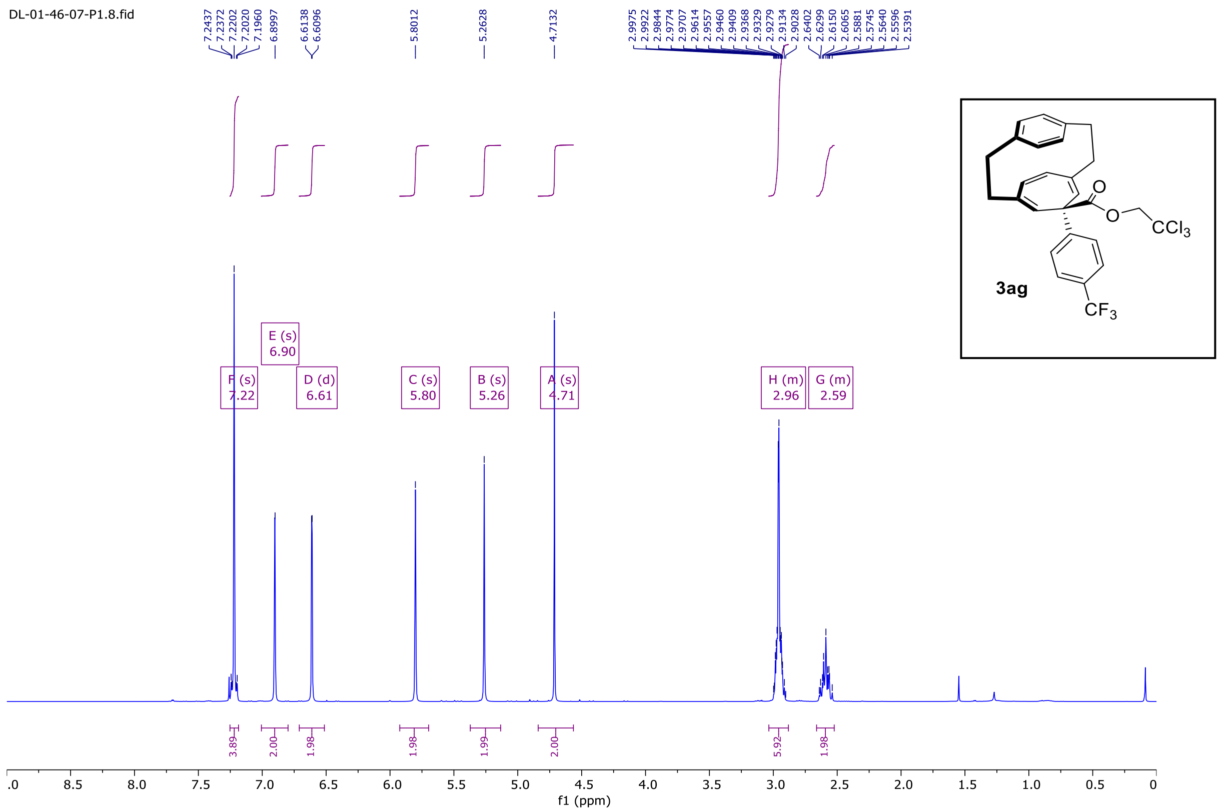

Figure S59. ^{1}**H-NMR of 3ag**

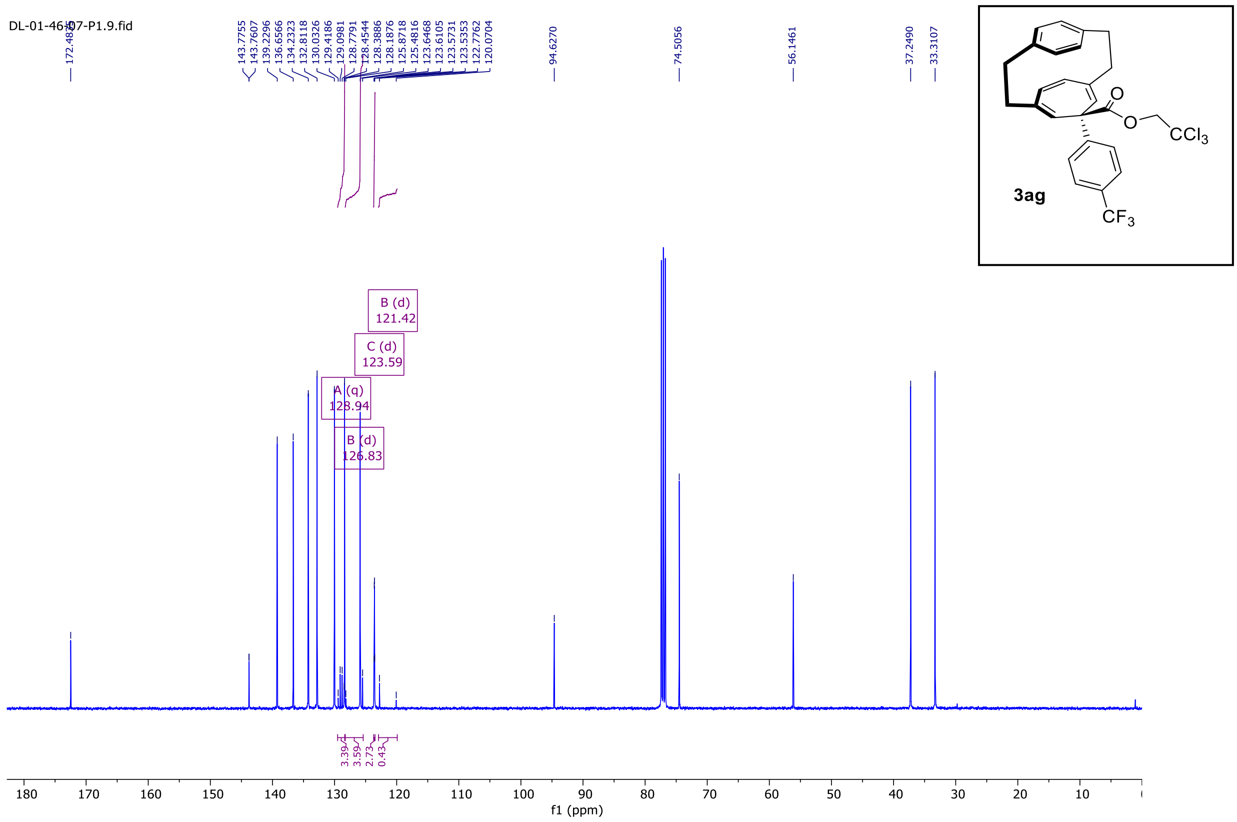

Figure S60. ^{13}**C-NMR of 3ag**

DL-01-46-07-P1-19F-NMR
STANDARD FLUORINE PARAMETERS

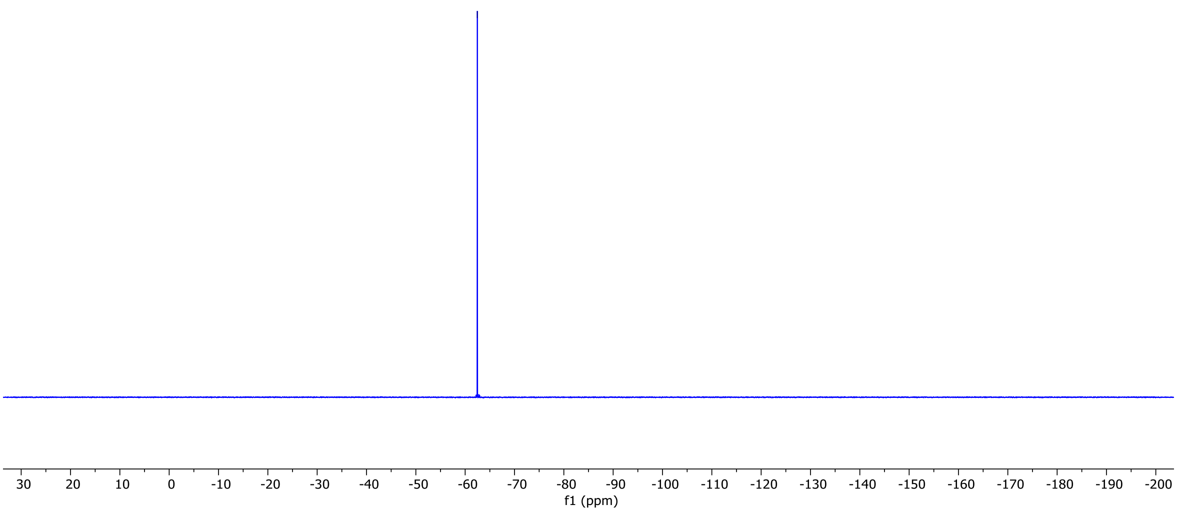

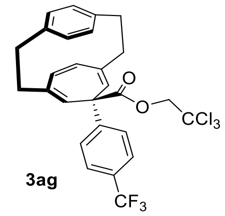

3ag

Figure S61. ^{19}F-NMR of 3ag

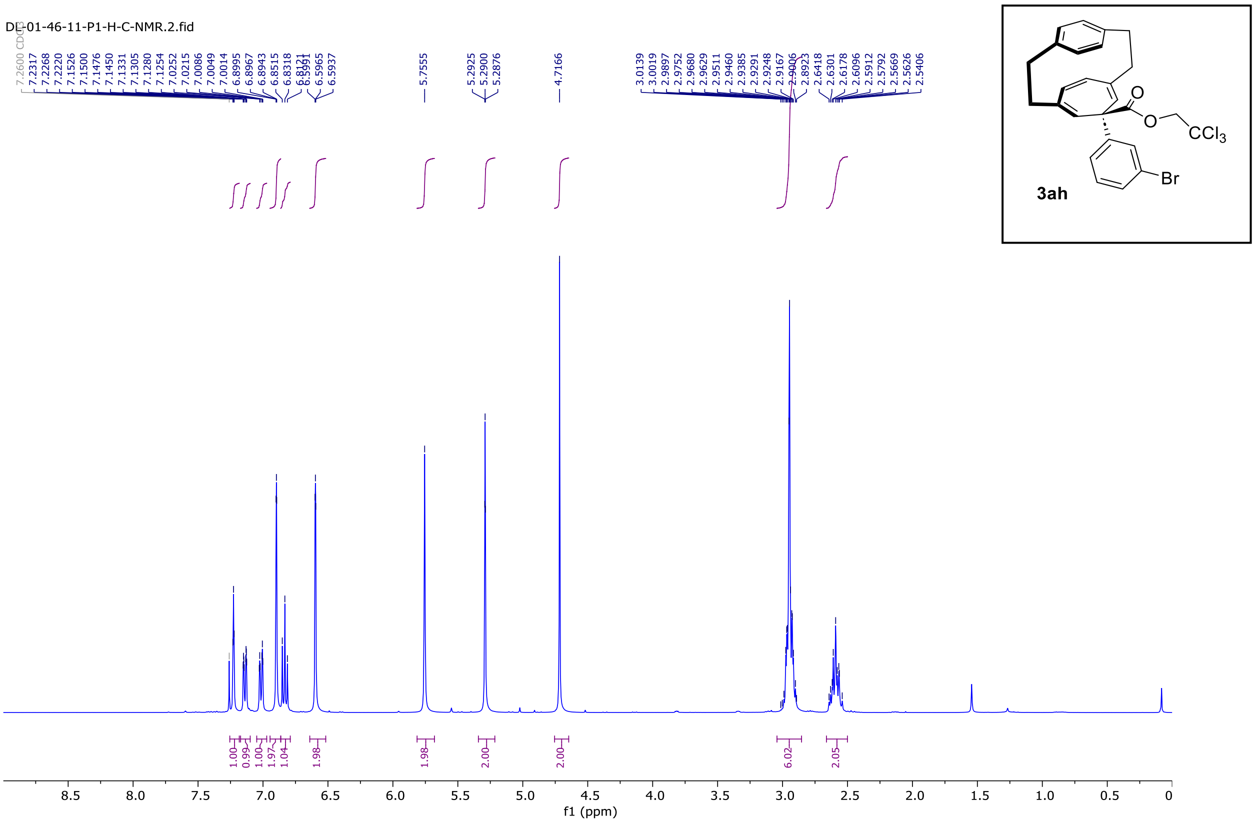

Figure S62. ^{1}H-NMR of 3ah

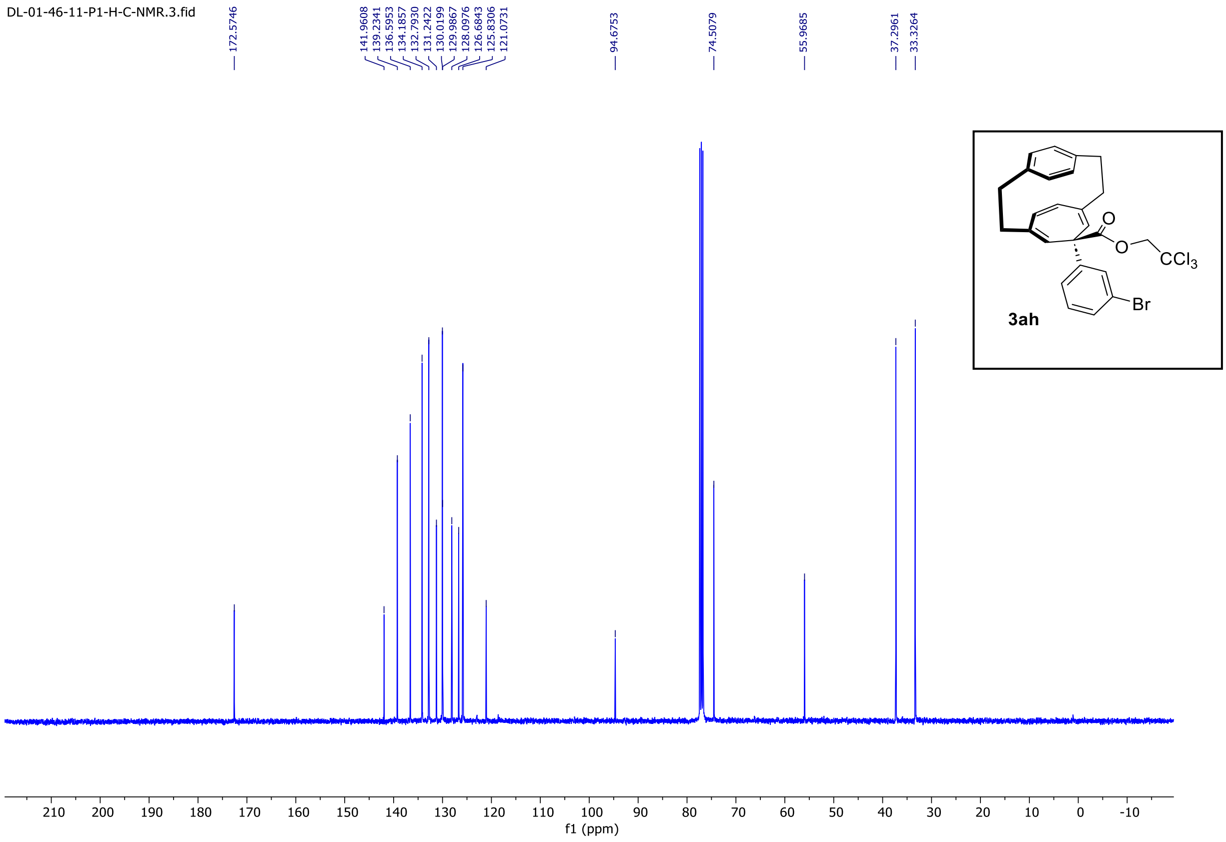

Figure S63. ^{13}C-NMR of 3ah

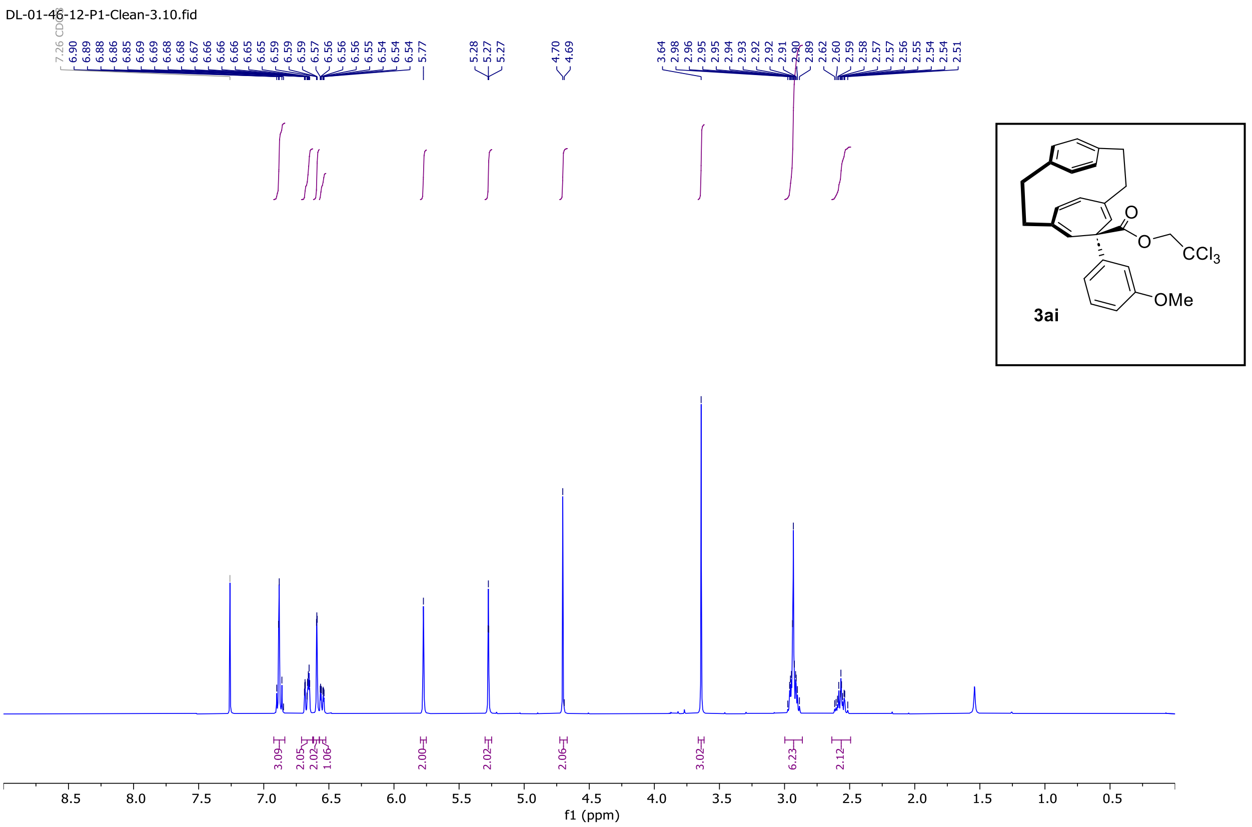

Figure S64. ^{1}H-NMR of 3ai

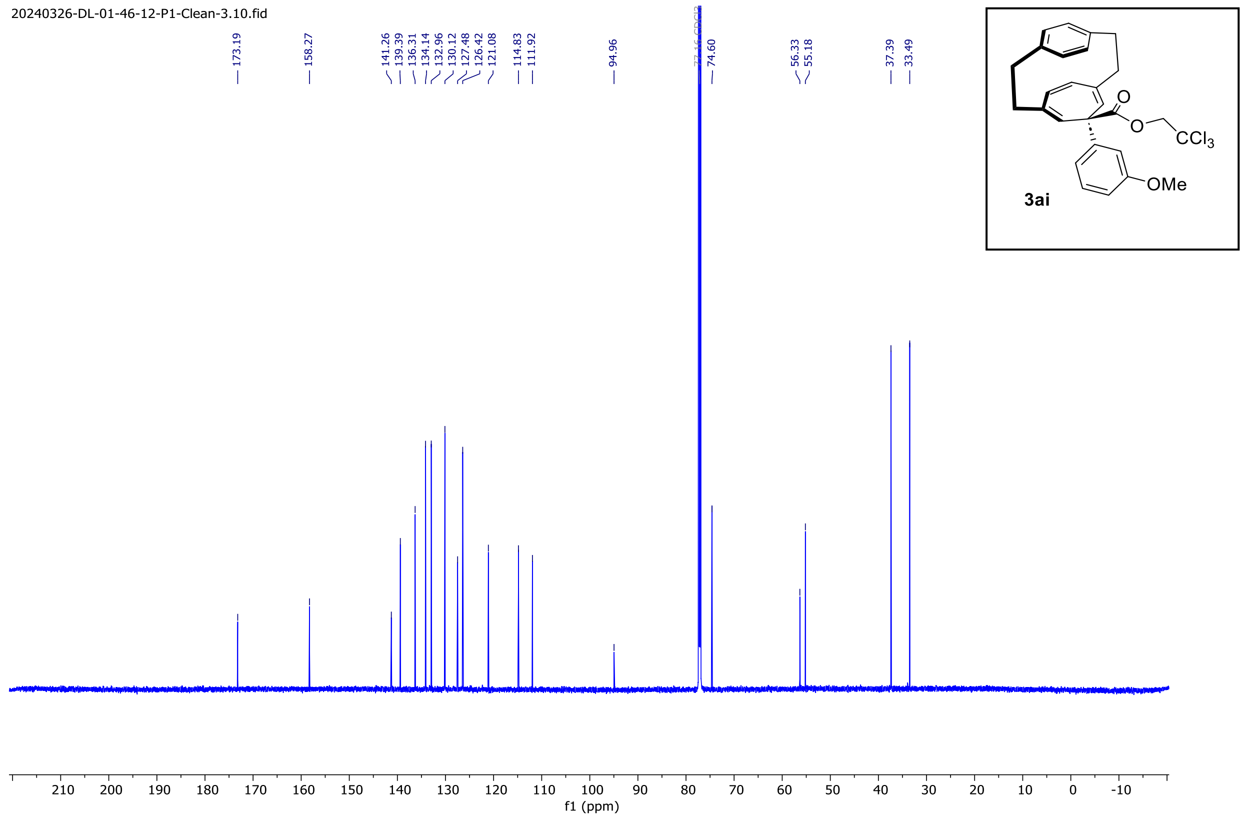

Figure S65. ^{13}C-NMR of 3ai

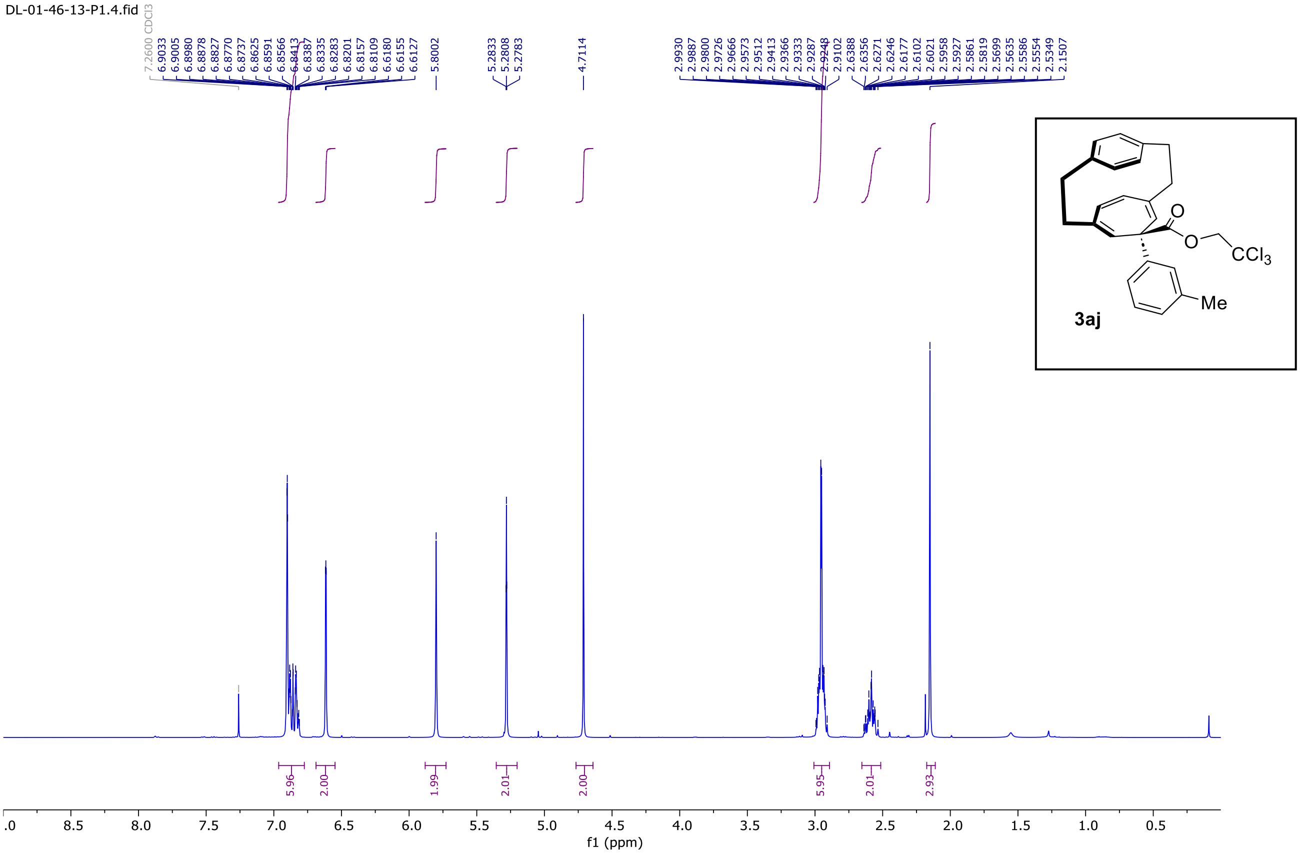

Figure S66. ¹H-NMR of 3aj

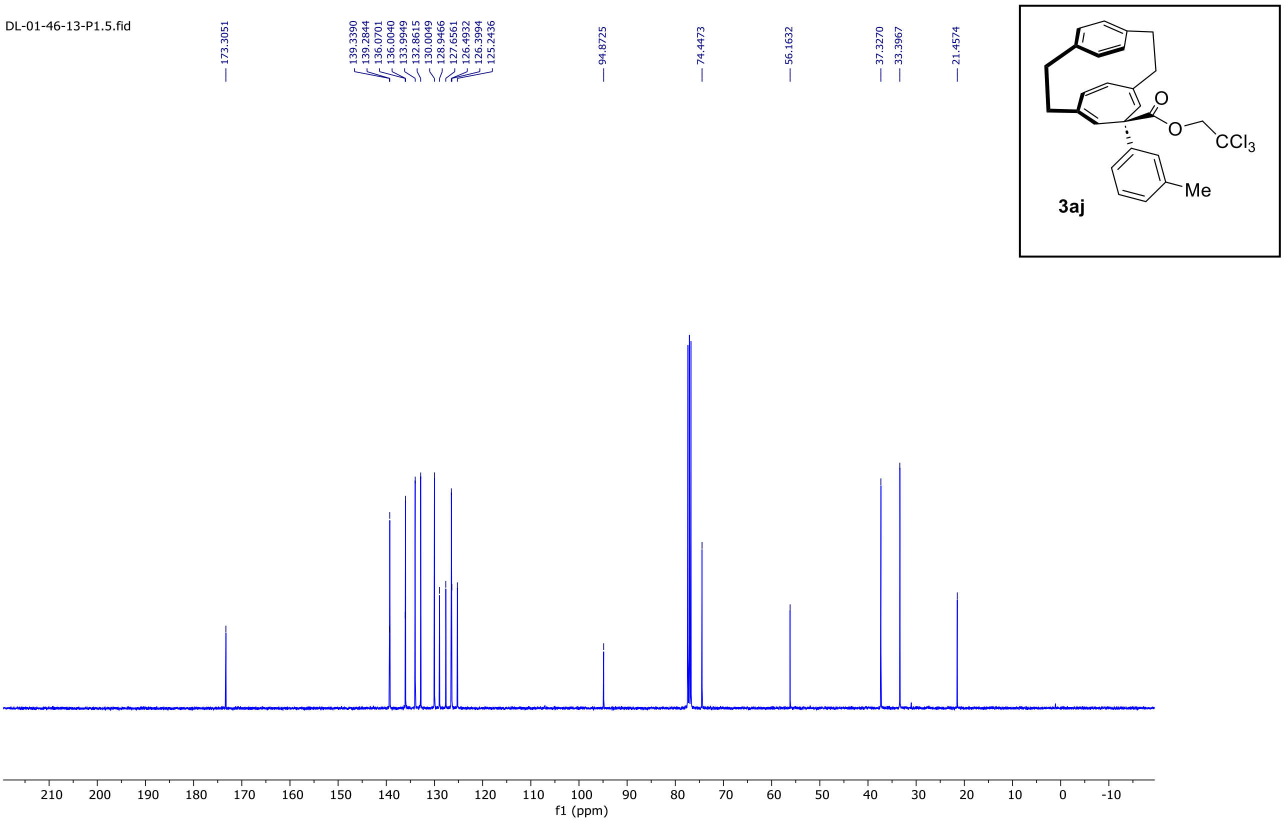

Figure S67. ¹³C-NMR of 3aj

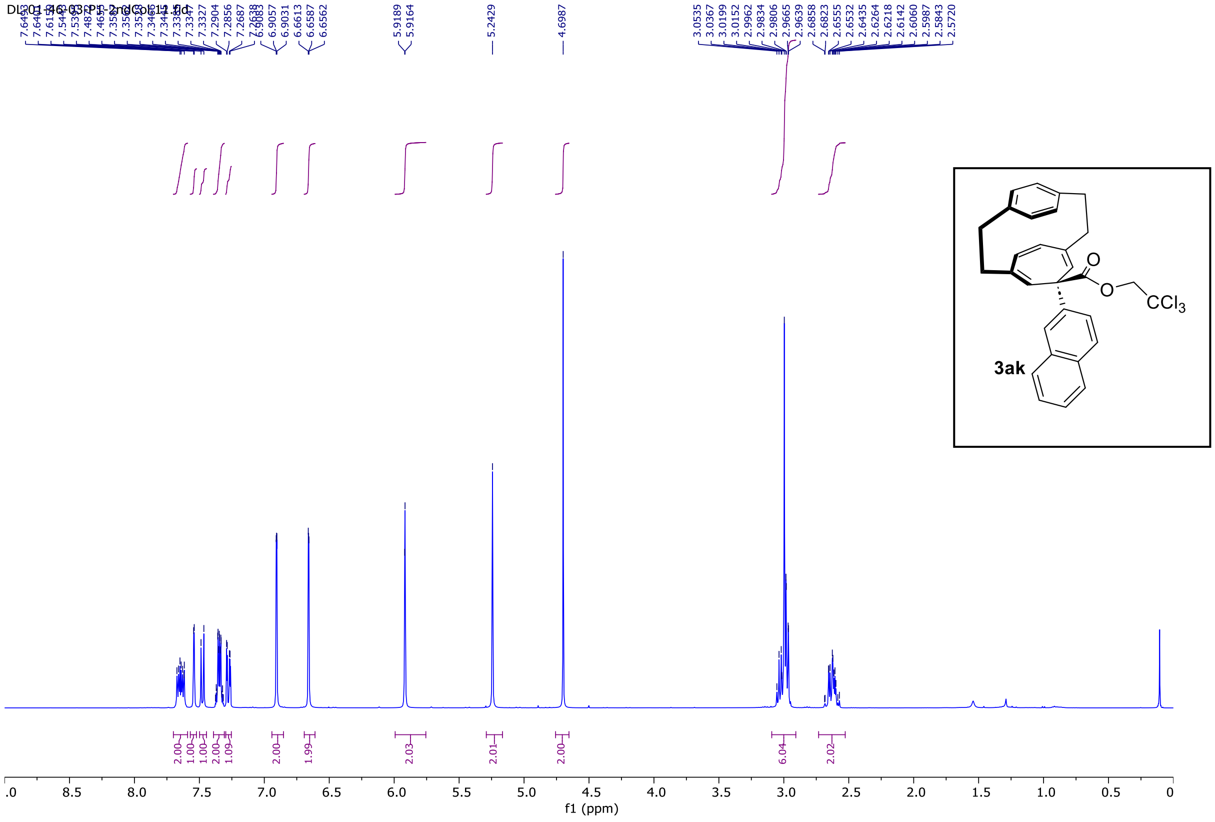

Figure S68. ^{1}**H-NMR of 3ak**

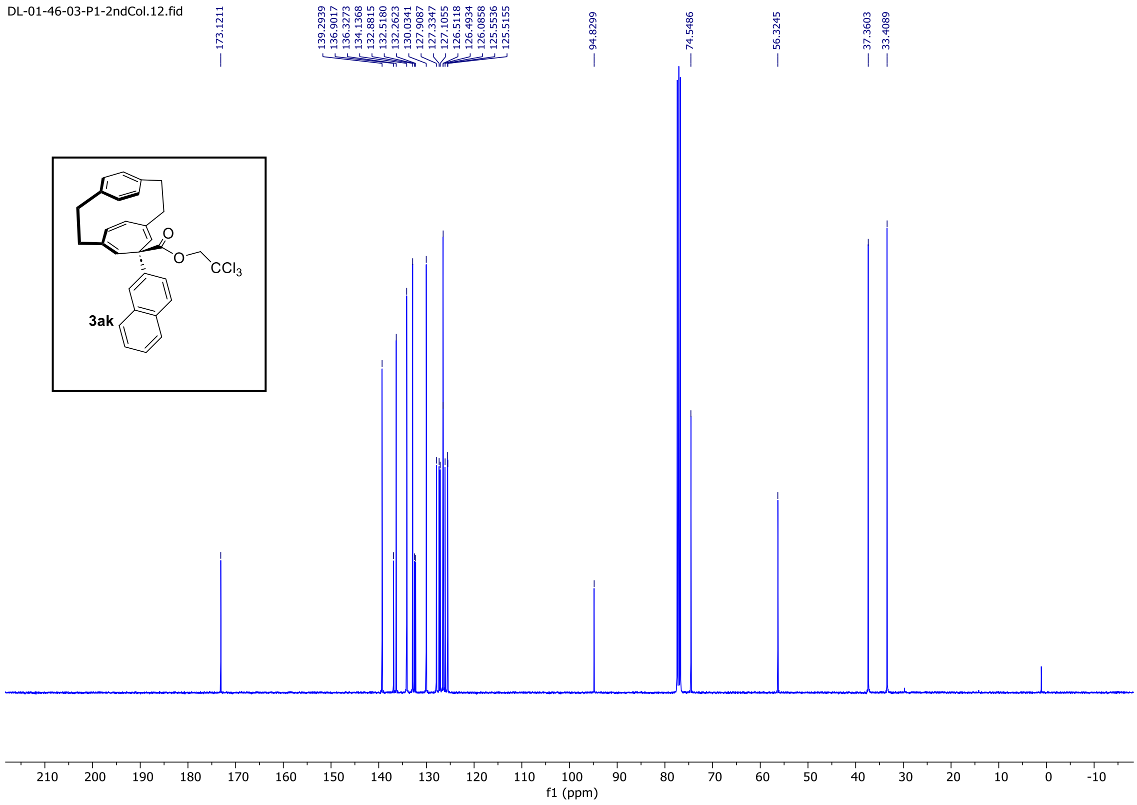

Figure S69. ^{13}**C-NMR of 3ak**

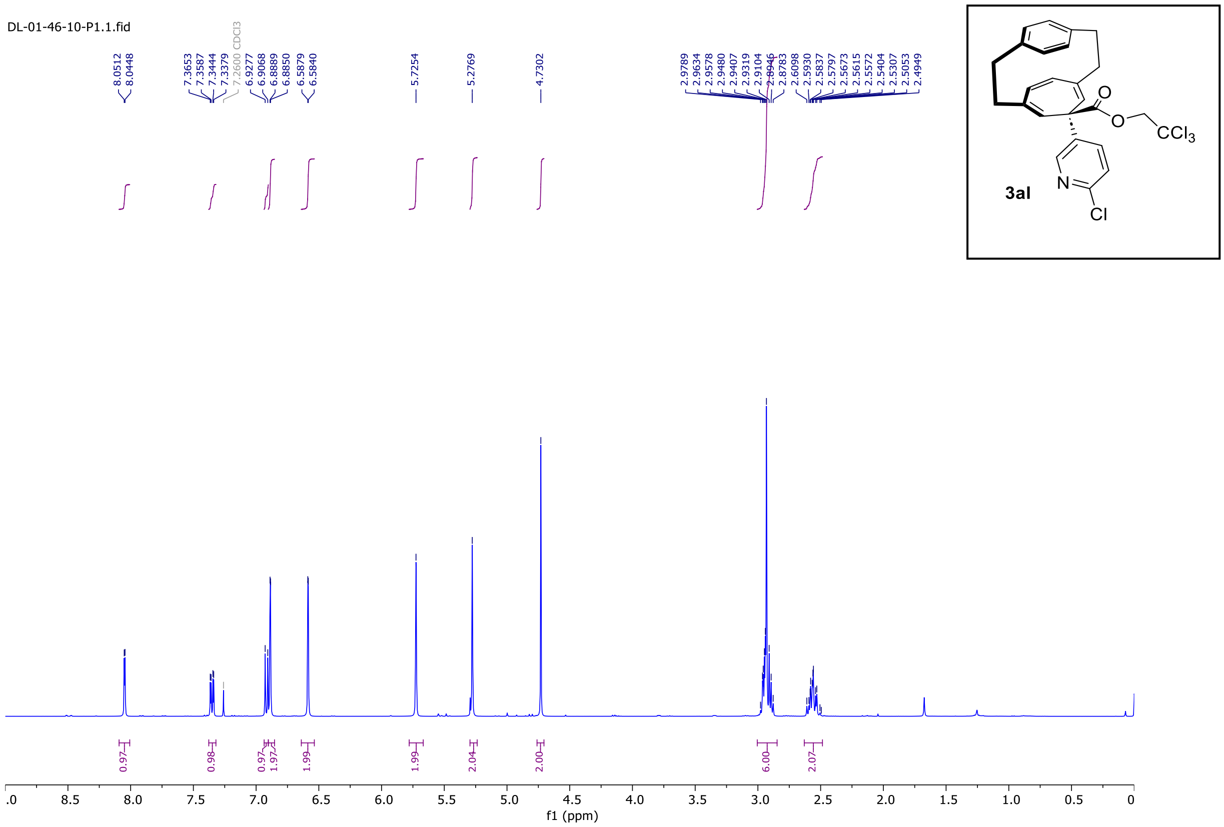

Figure S70. ^{1}H-NMR of 3al

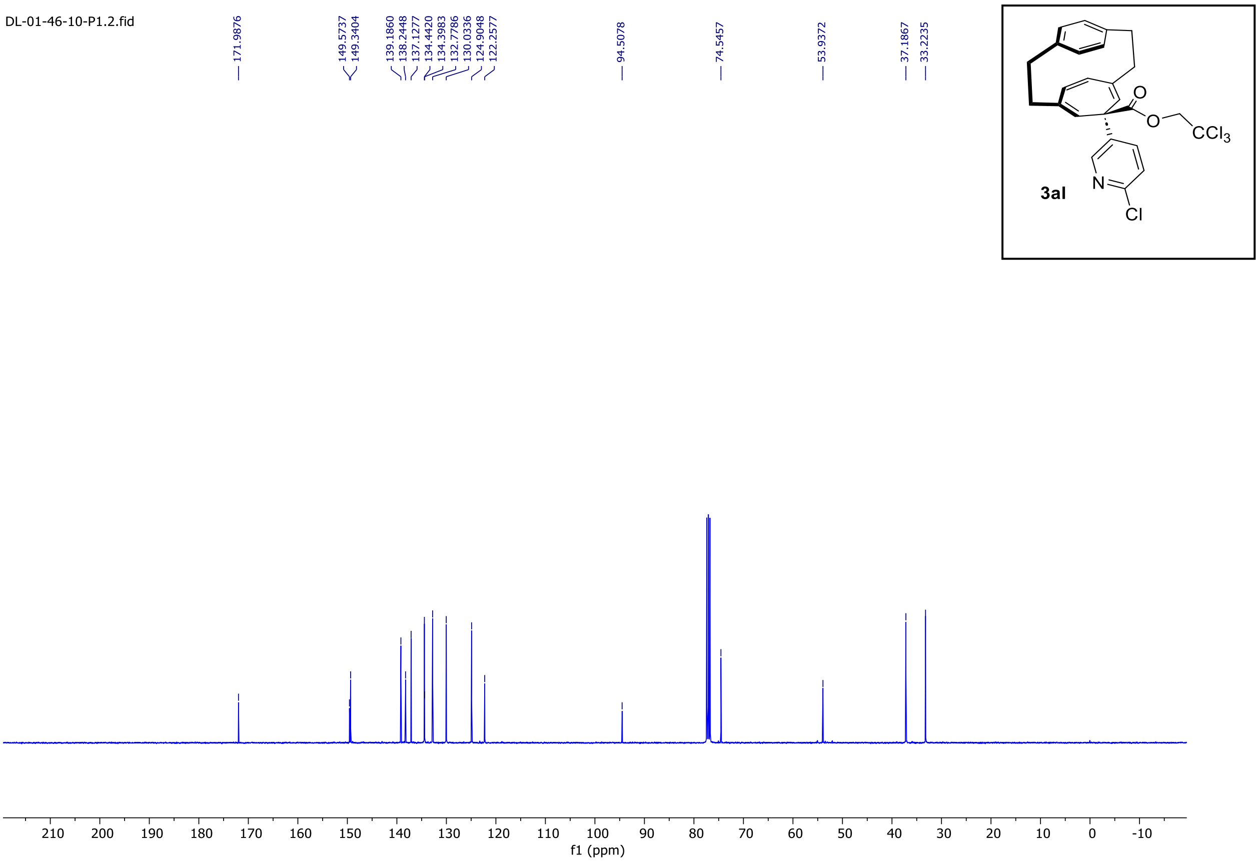

Figure S71. ^{13}C-NMR of 3al

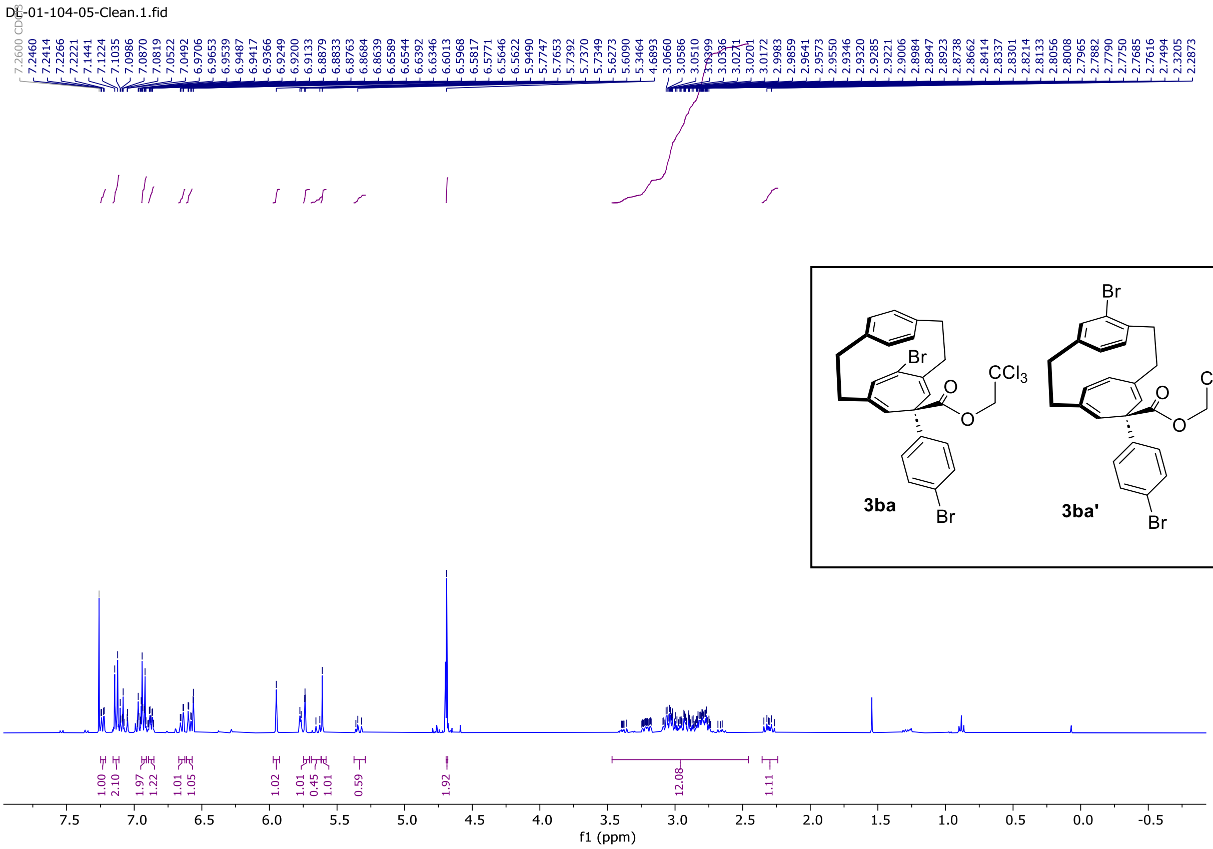

Figure S72. ^{1}**H-NMR of 3ba and 3ba' (2:1 ratio)**

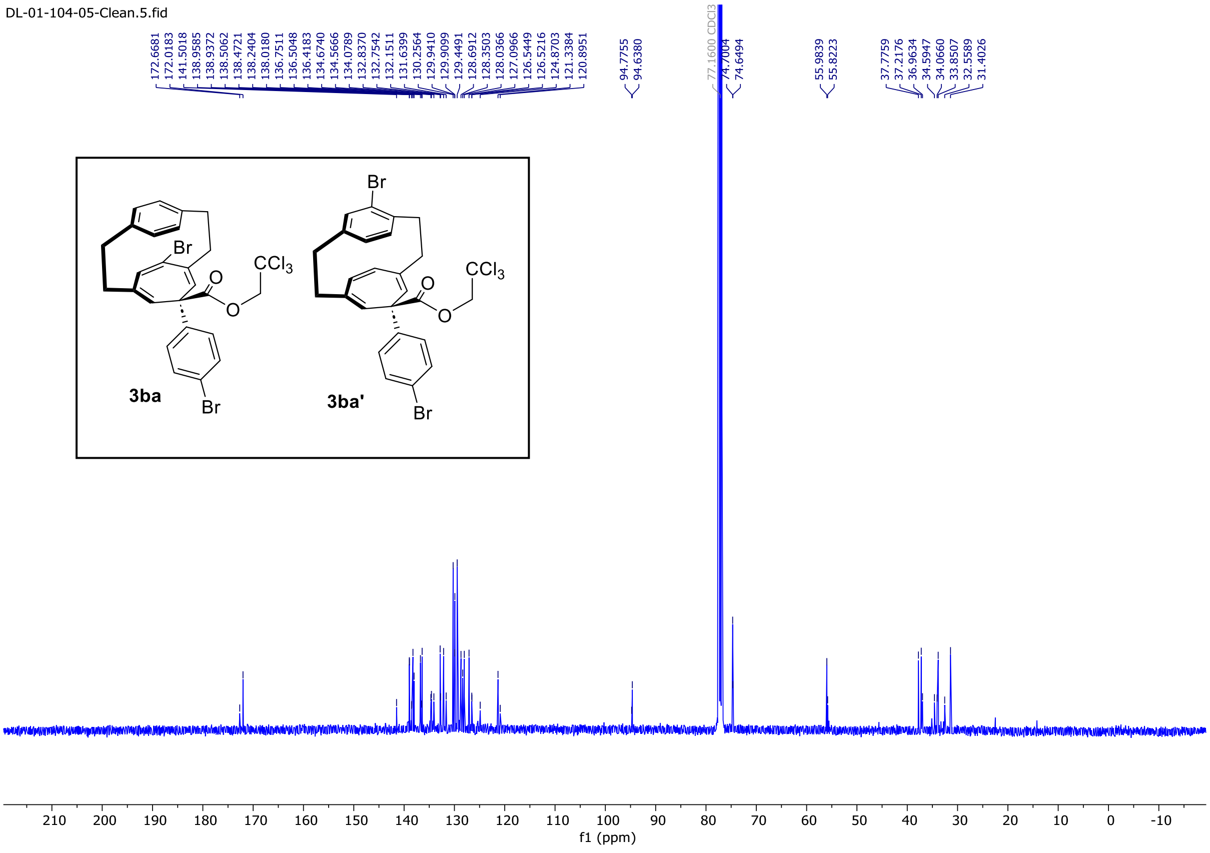

Figure S73. ^{13}**C-NMR of 3ba and 3ba' (2:1 ratio)**

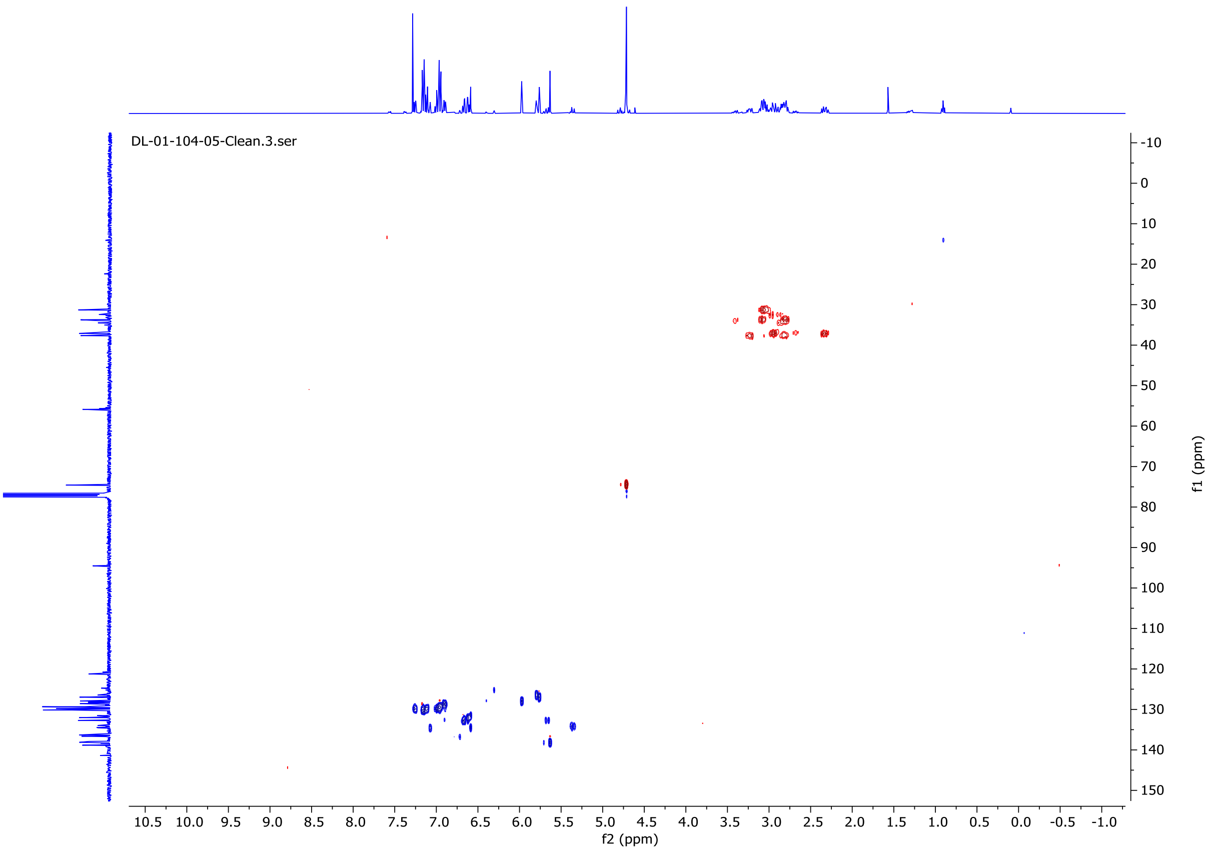

Figure S74. HSQC of 3ba and 3ba’ (2:1 ratio)

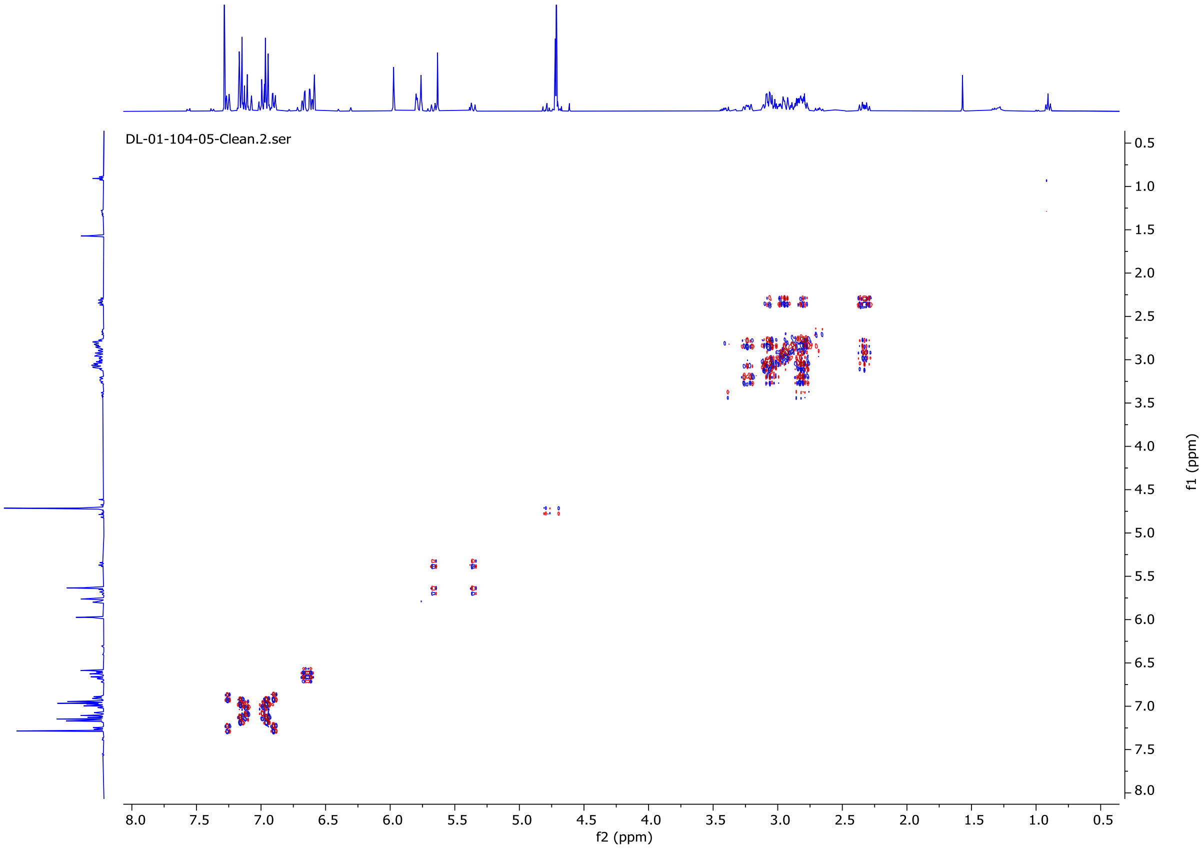

Figure S75. COSEY of 3ba and 3ba’ (2:1 ratio)

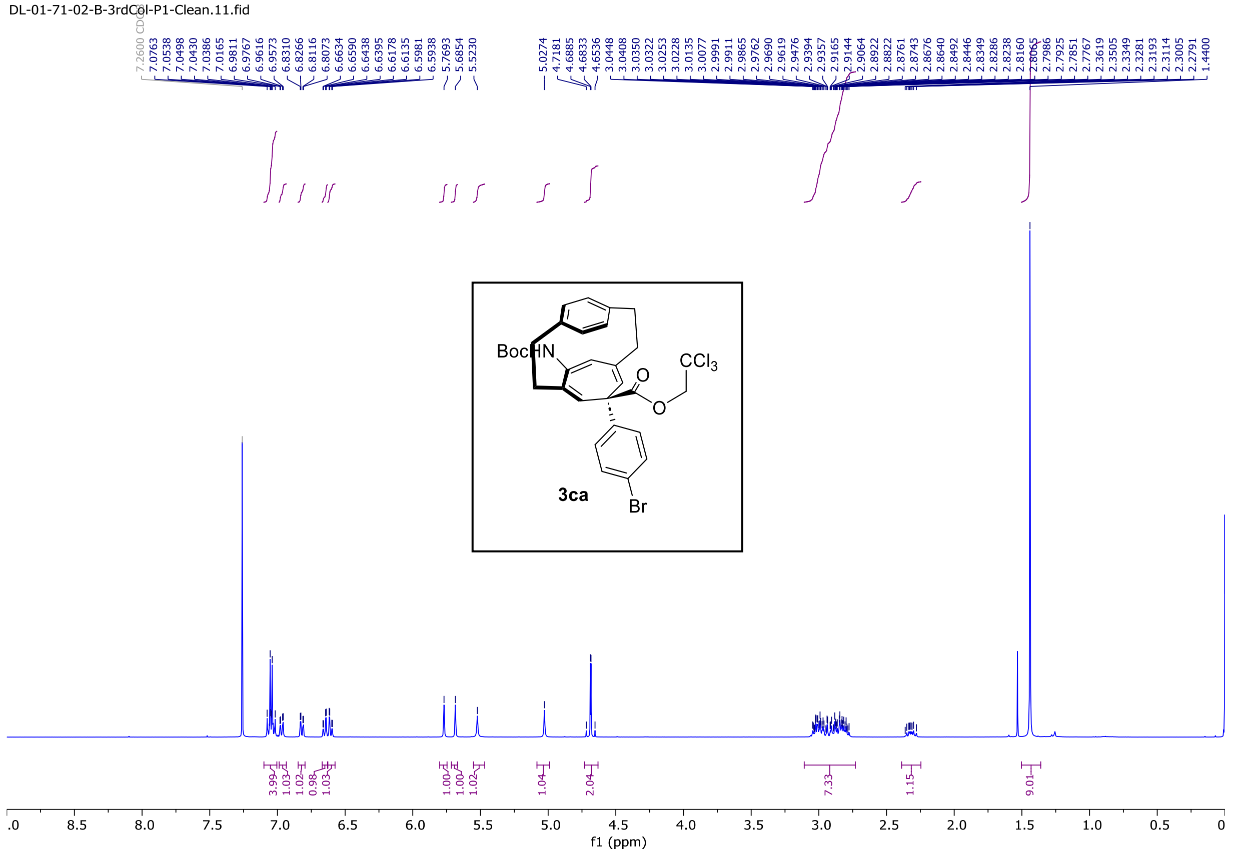

Figure S76. ^{1}H-NMR of 3ca

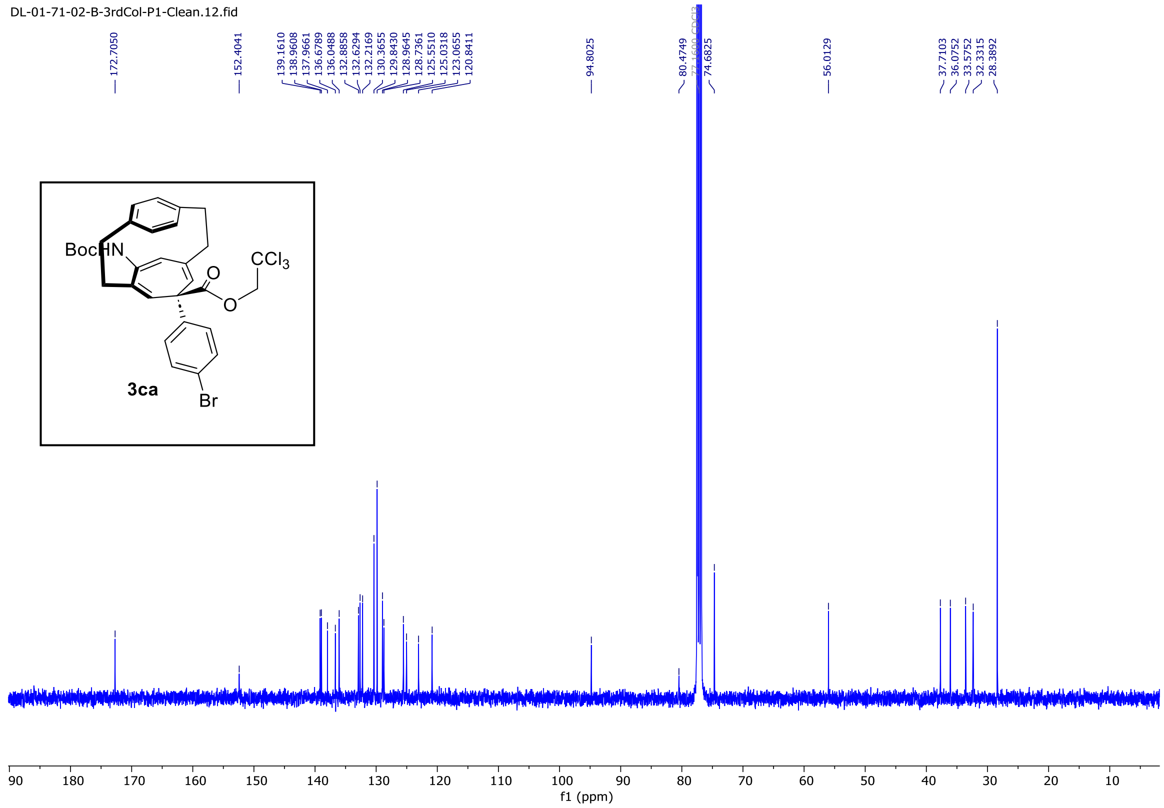

Figure S77. ^{13}C-NMR of 3ca

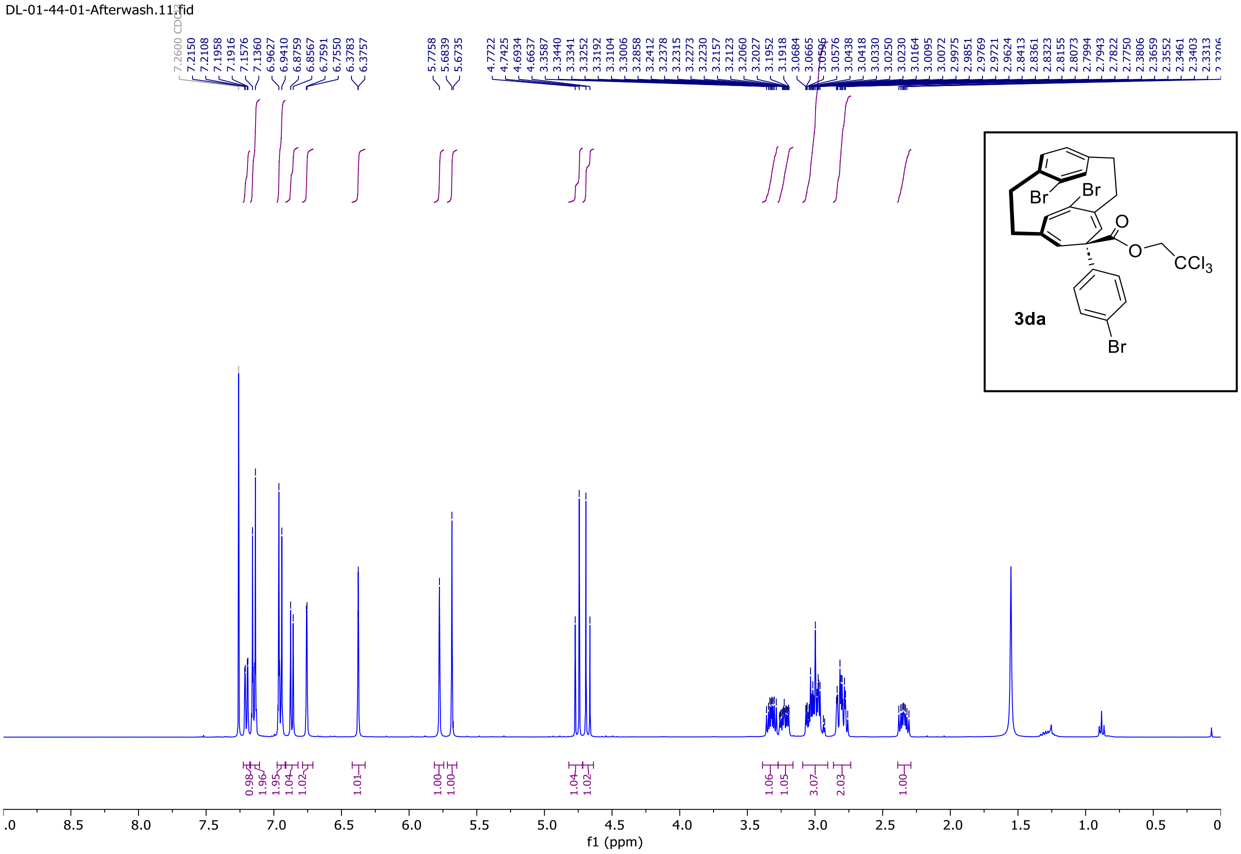

Figure S78. ¹H-NMR of 3da

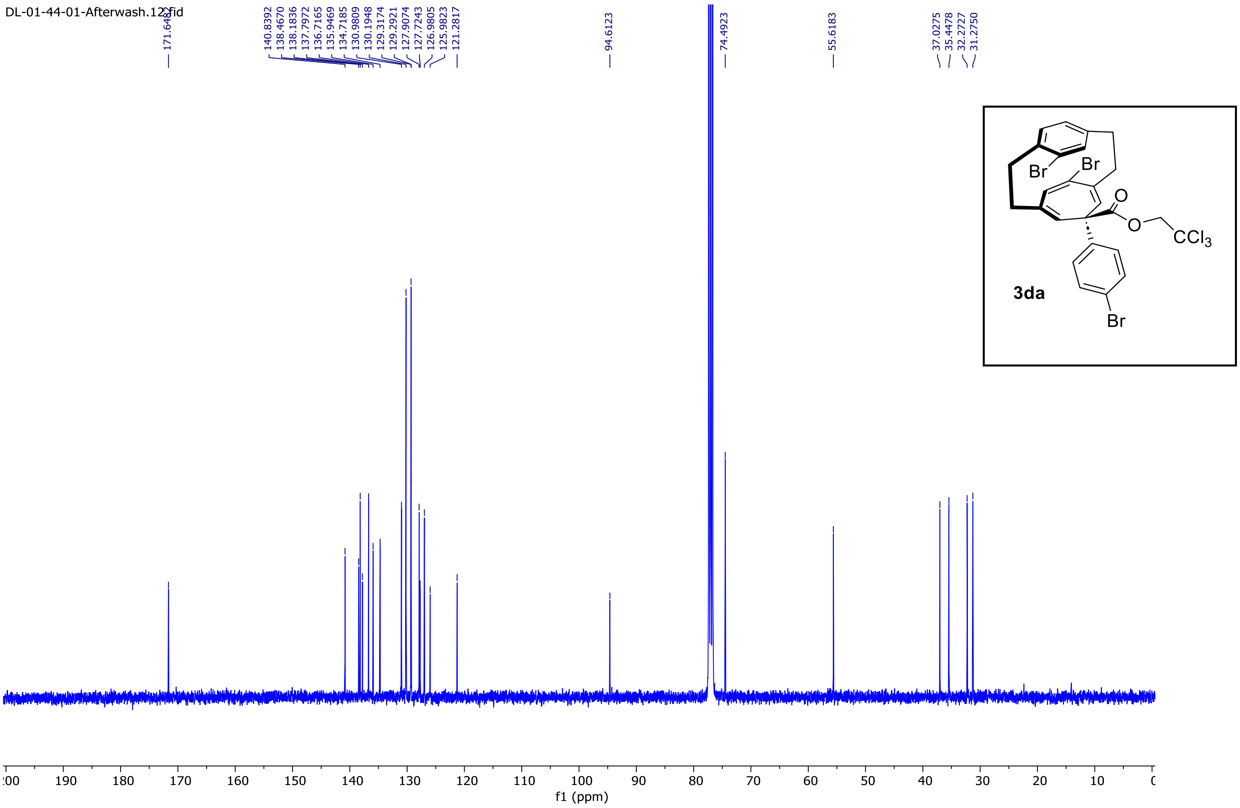

Figure S79. ¹³C-NMR of 3da

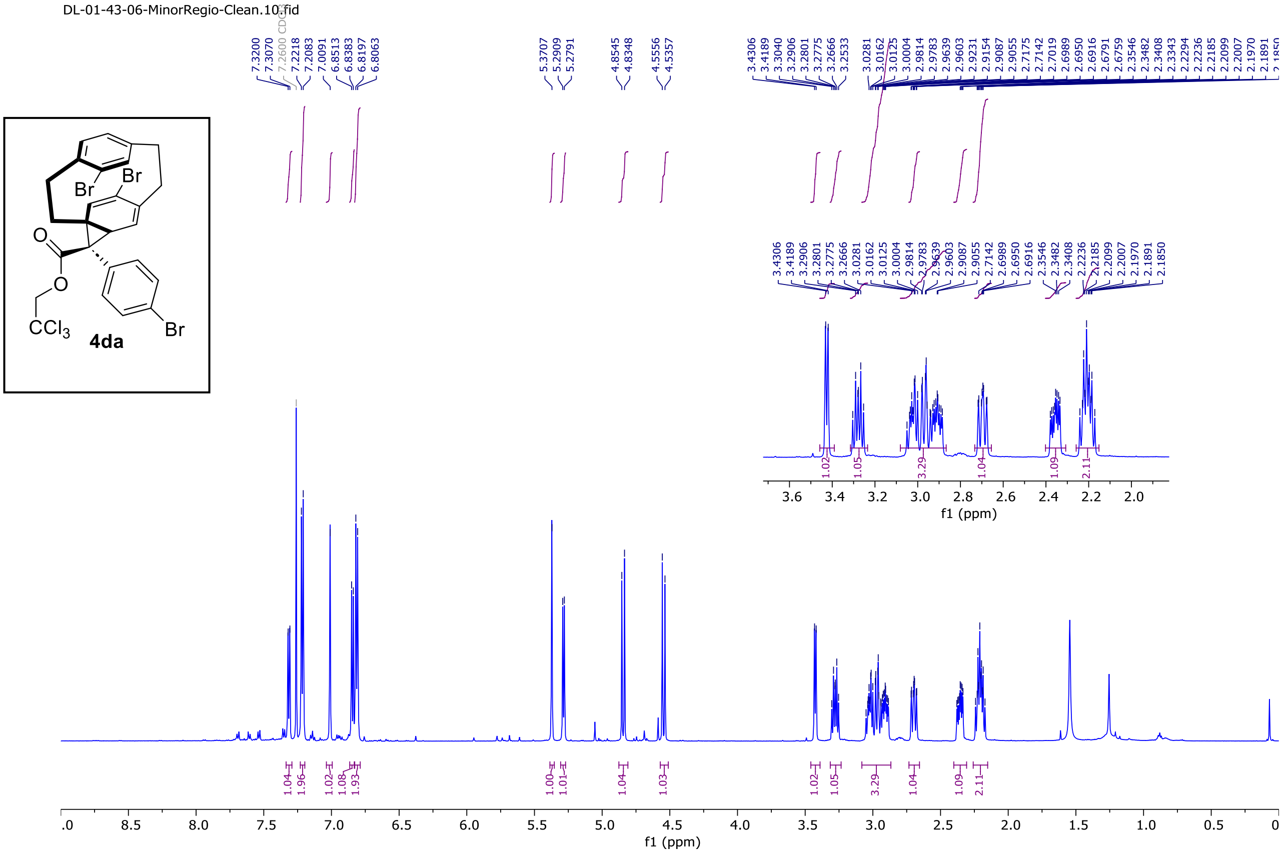

Figure S80. ^{1}H-NMR of 4da

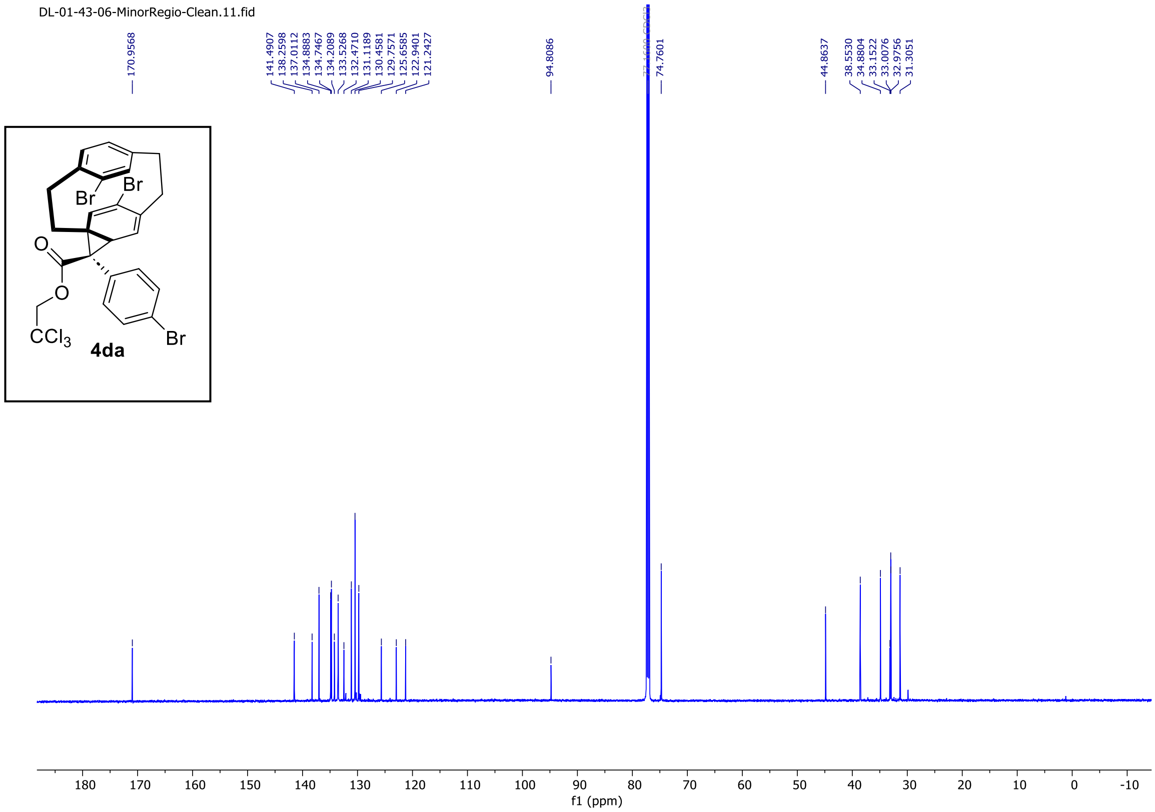

Figure S81. ^{13}C-NMR of 4da

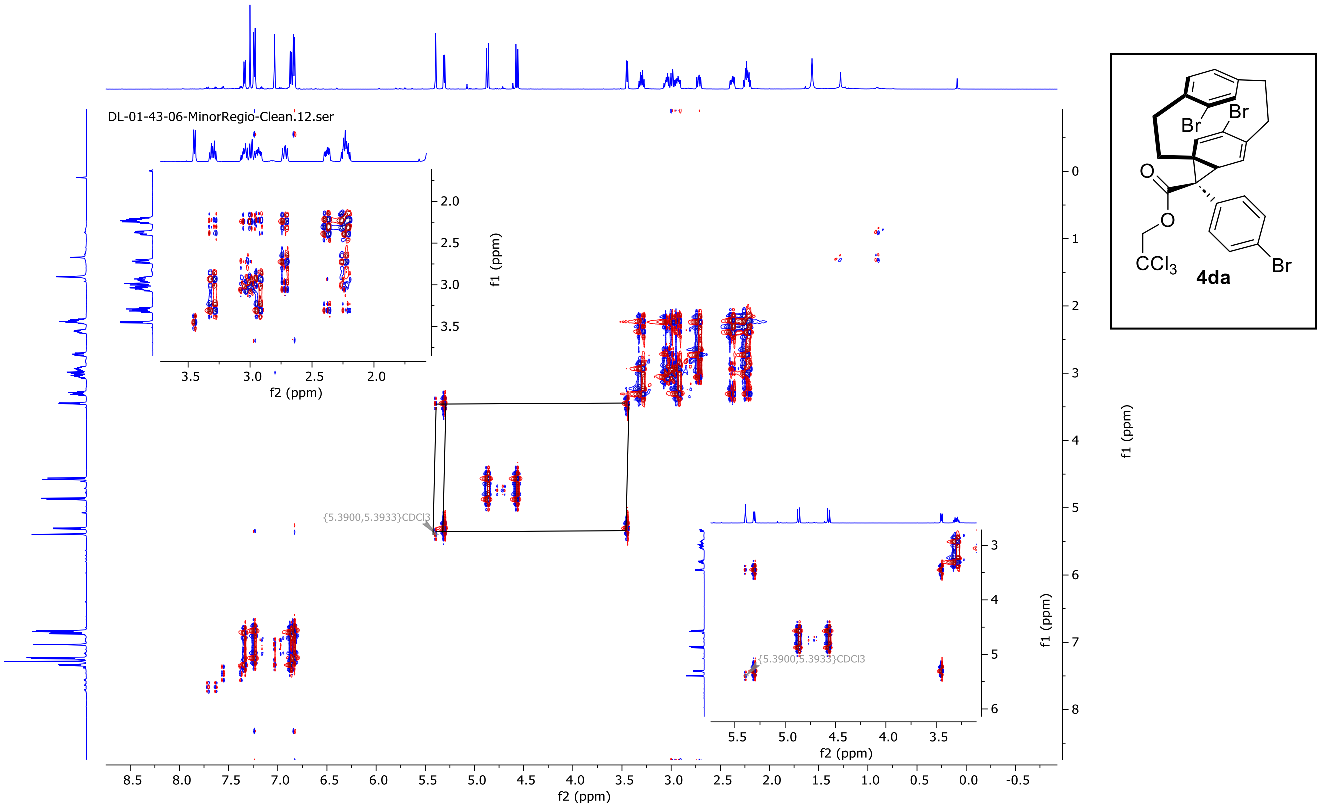

Figure S82. COSEY of 4da

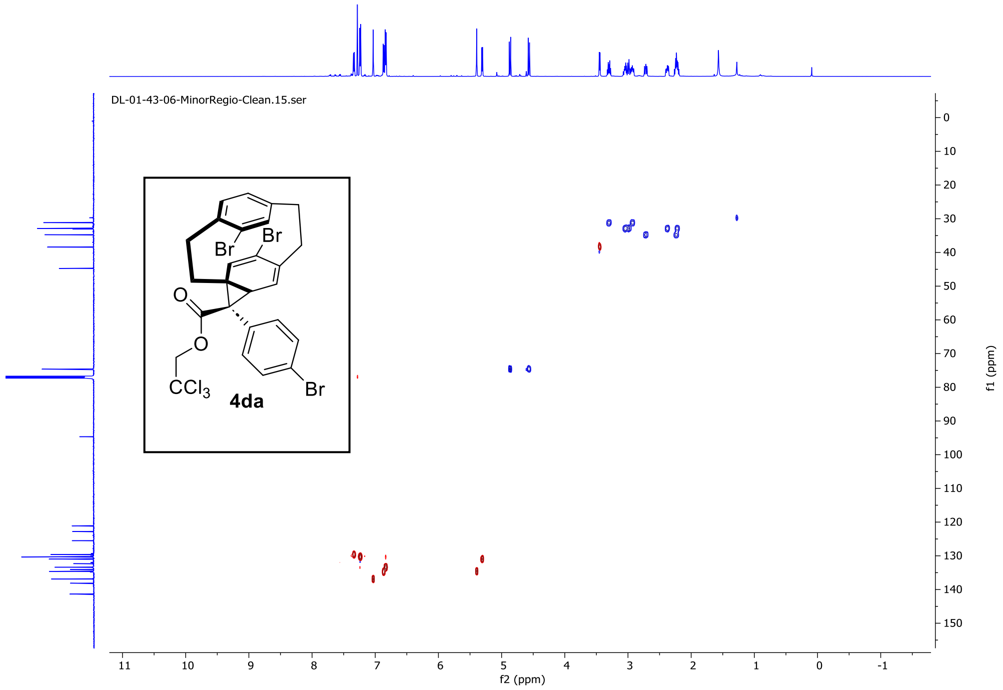

Figure S83. HSQC of 4da

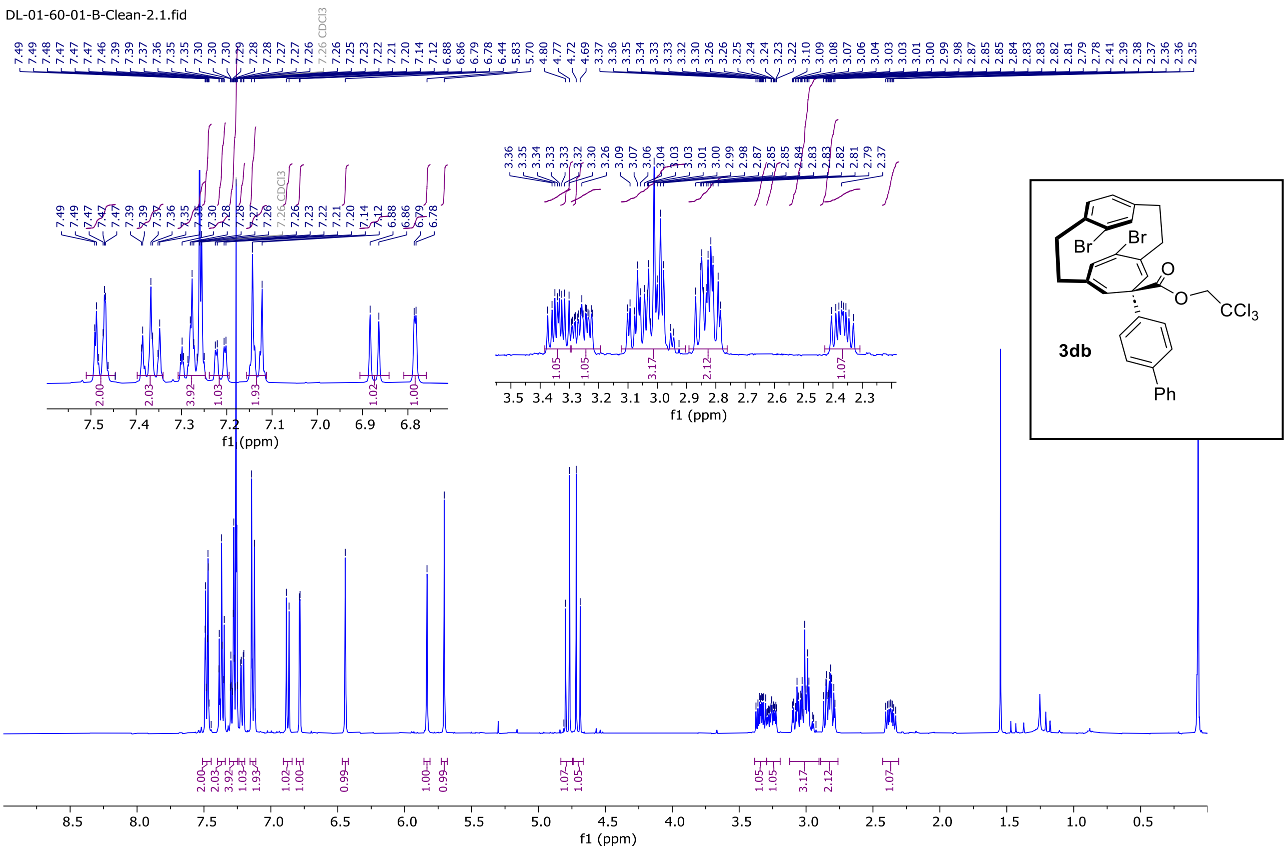

Figure S84. ^{1}H-NMR of 3db

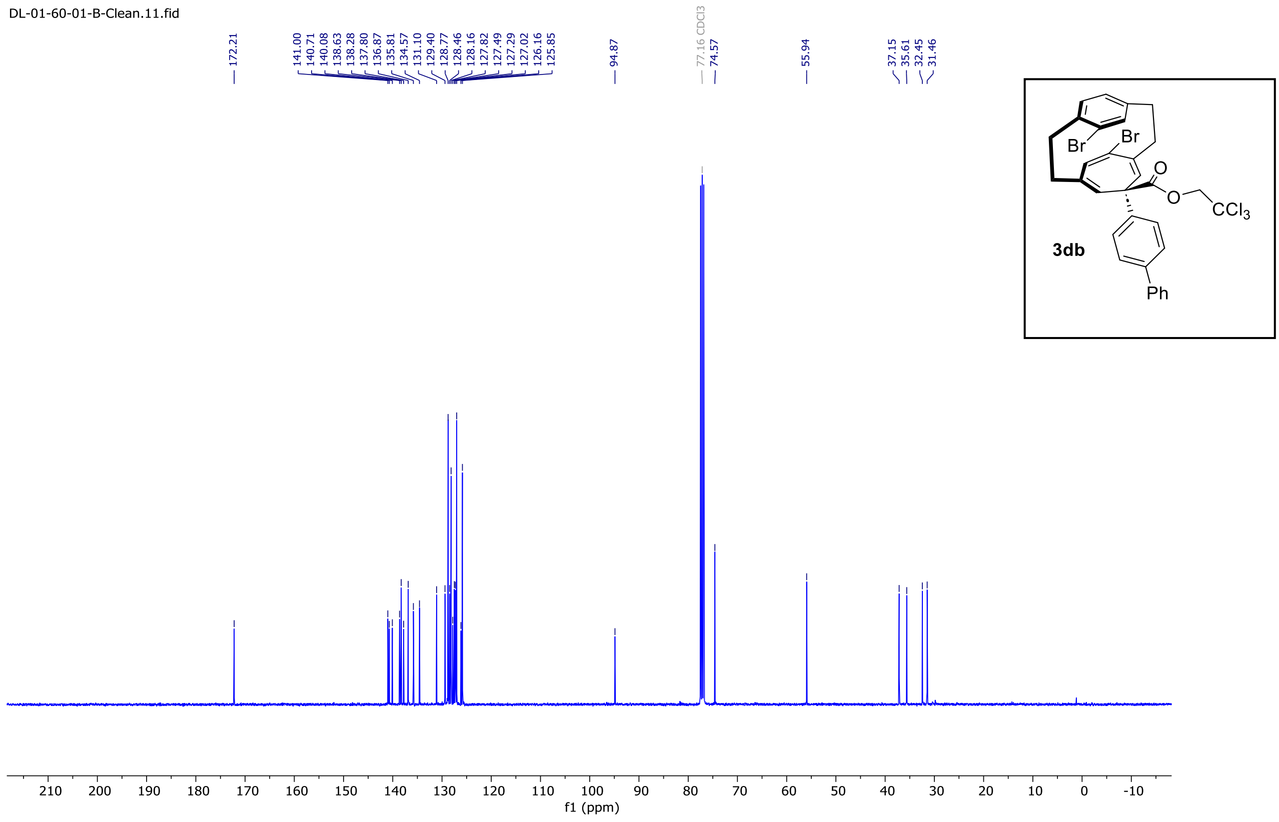

Figure S85. ^{13}C-NMR of 3db

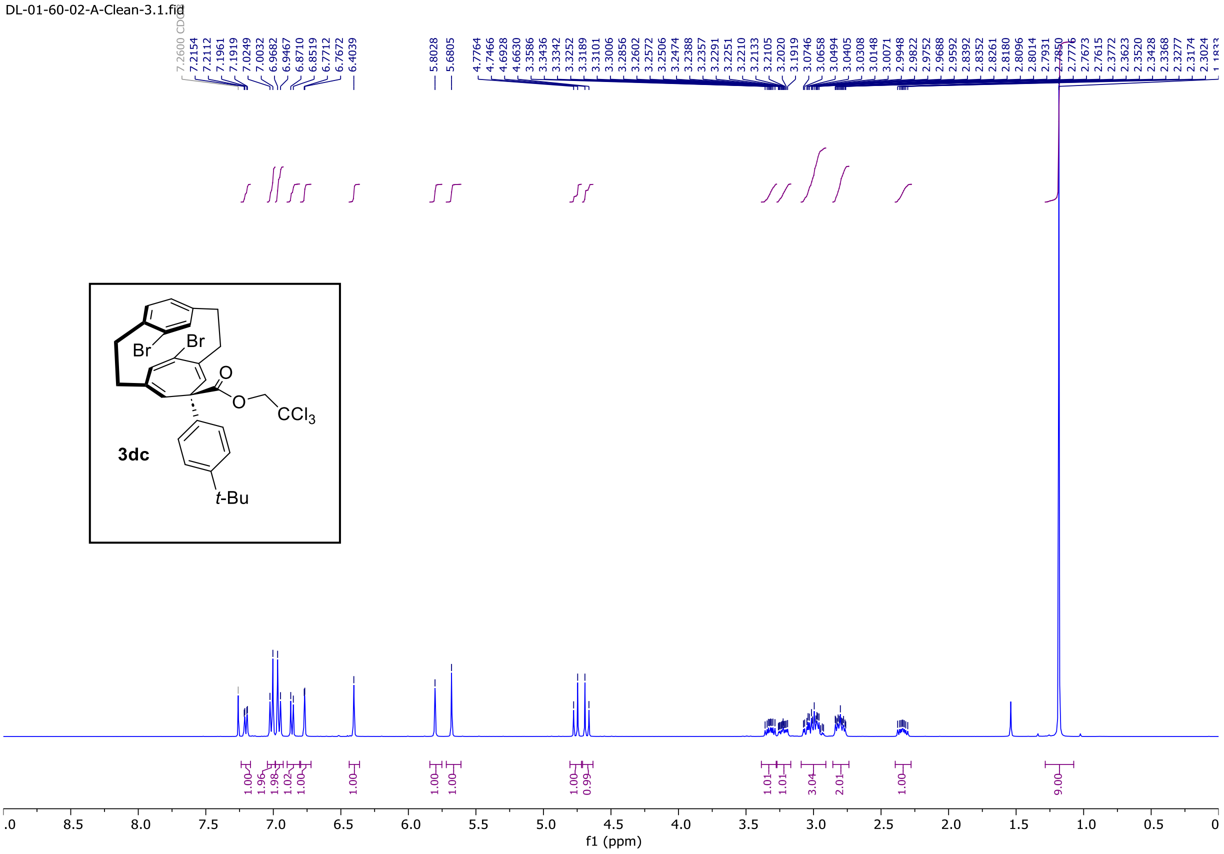

Figure S86. ^{1}H-NMR of 3dc

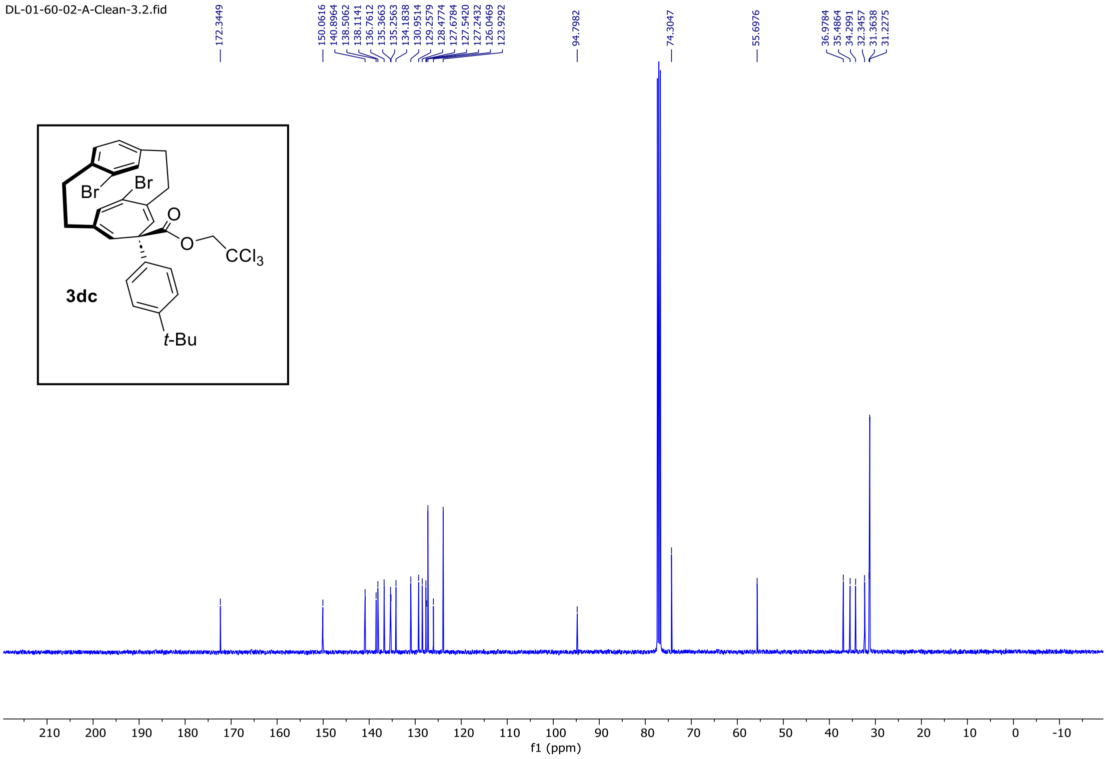

Figure S87. ^{13}C-NMR of 3dc

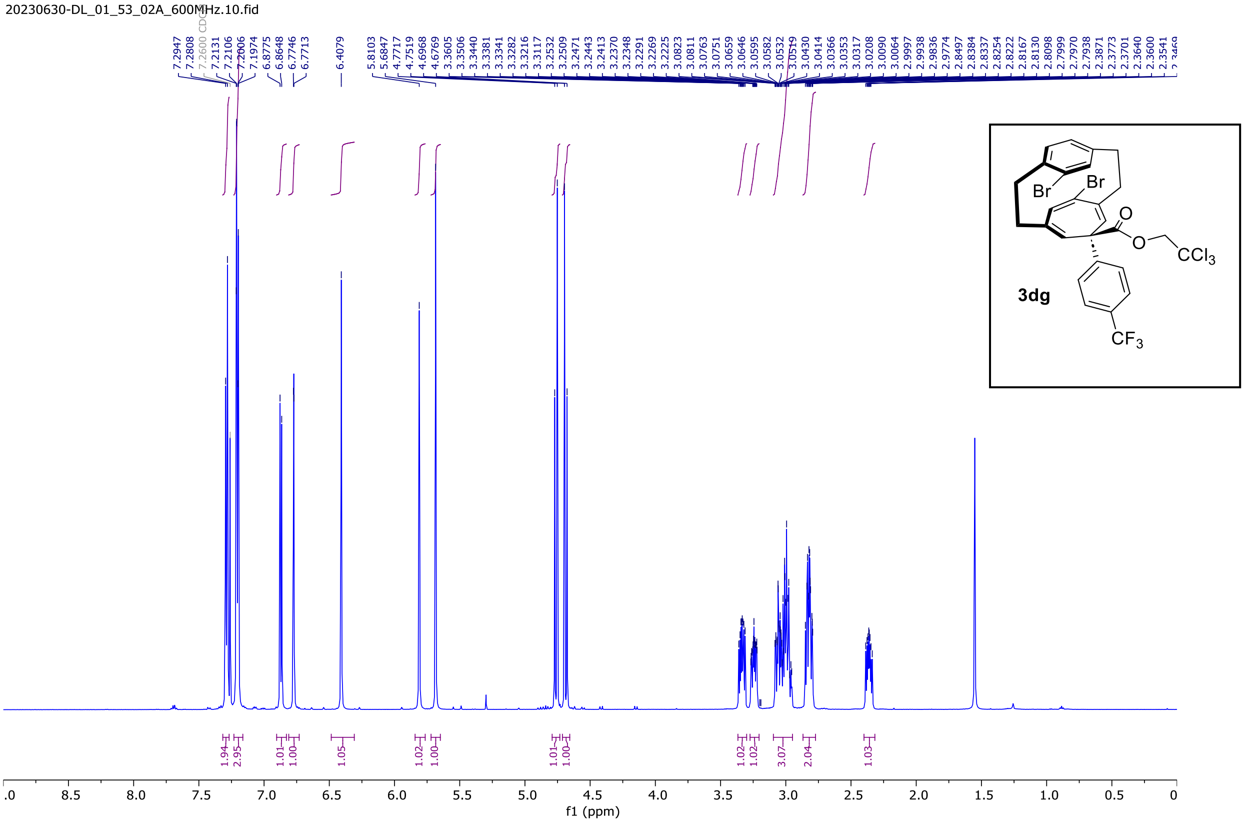

Figure S88. ^{1}H-NMR of 3dg

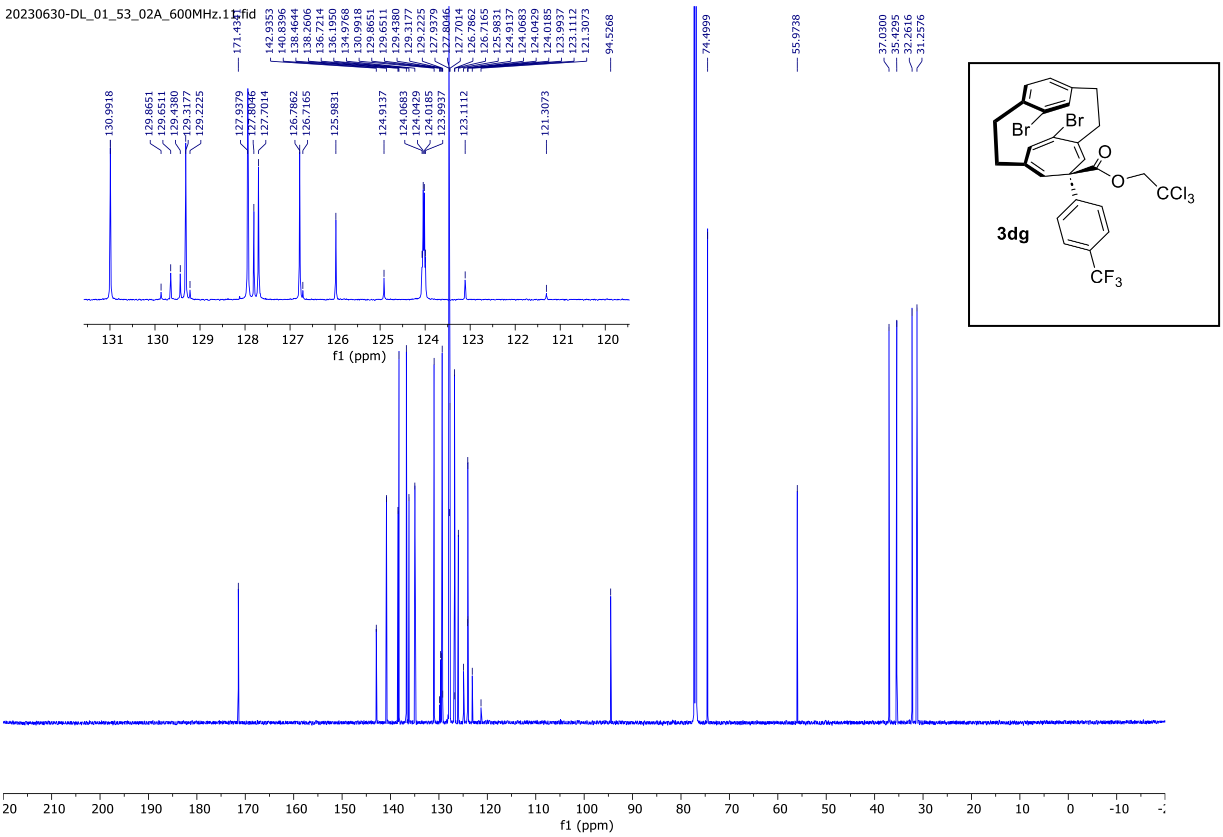

Figure S89. ^{13}C-NMR of 3dg

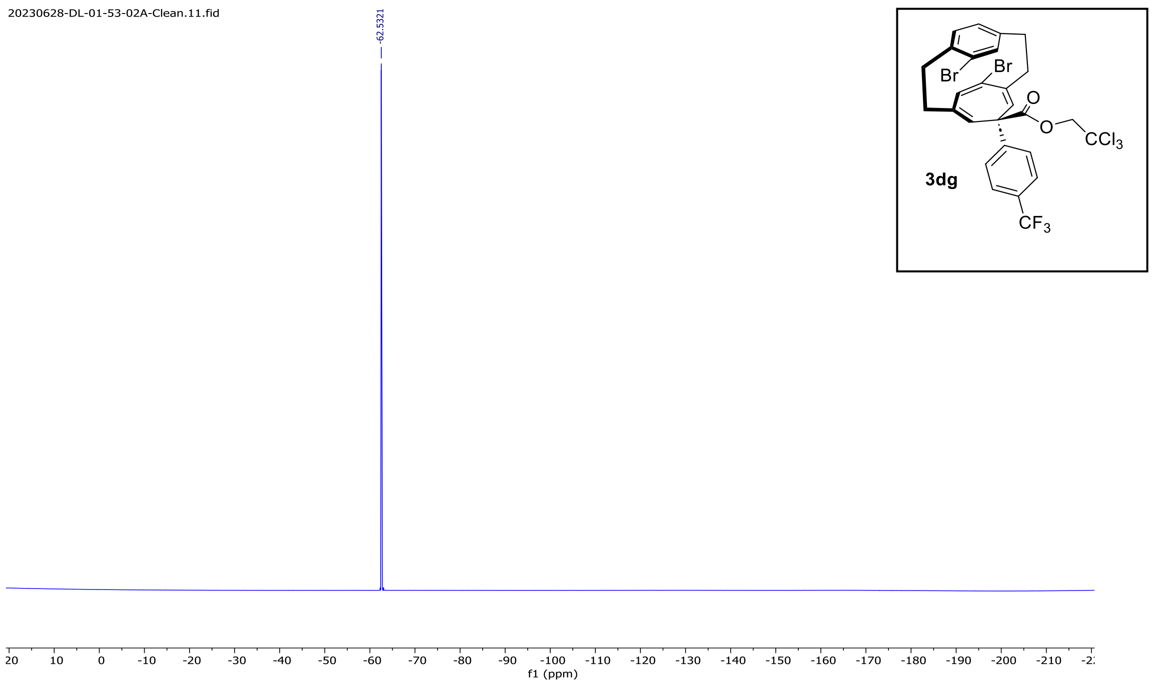

3dg

Figure S90. ^{19}F-NMR of 3dg

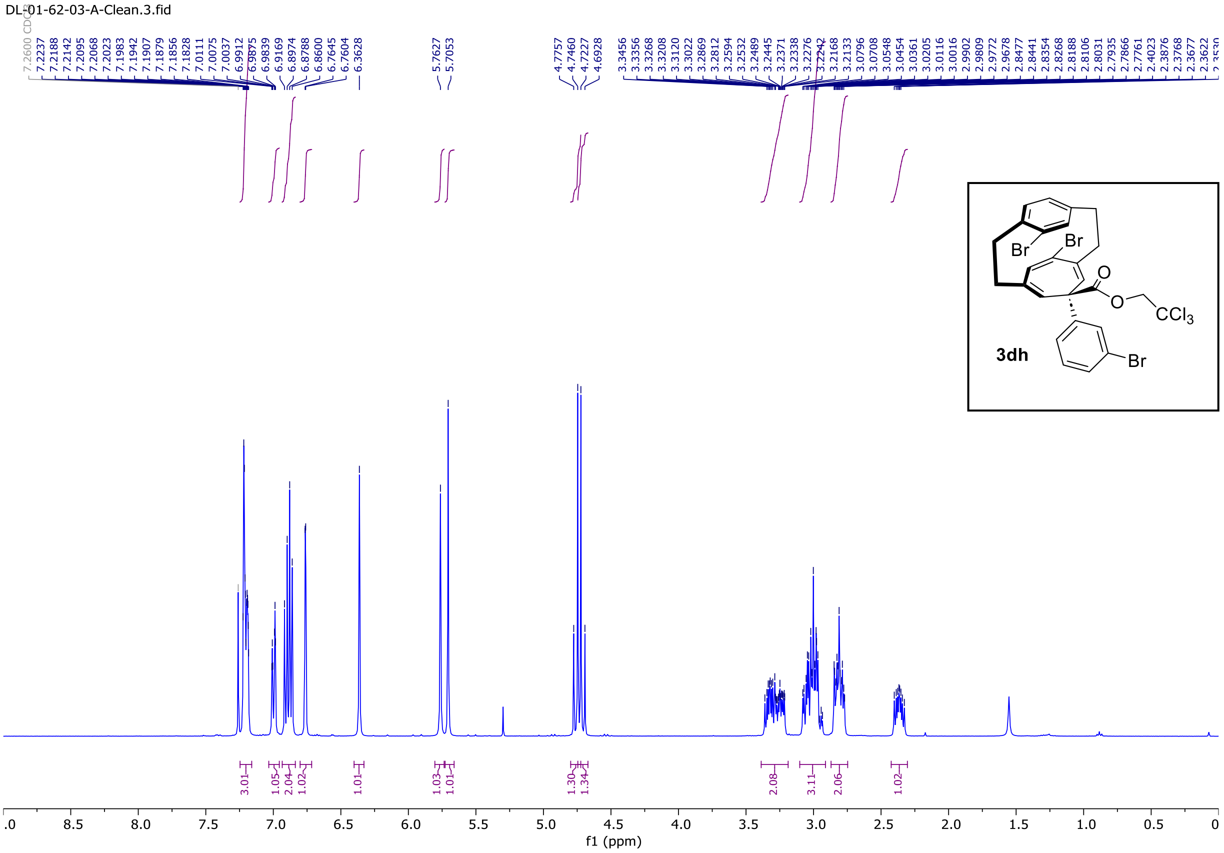

Figure S91. ^{1}H-NMR of 3dh

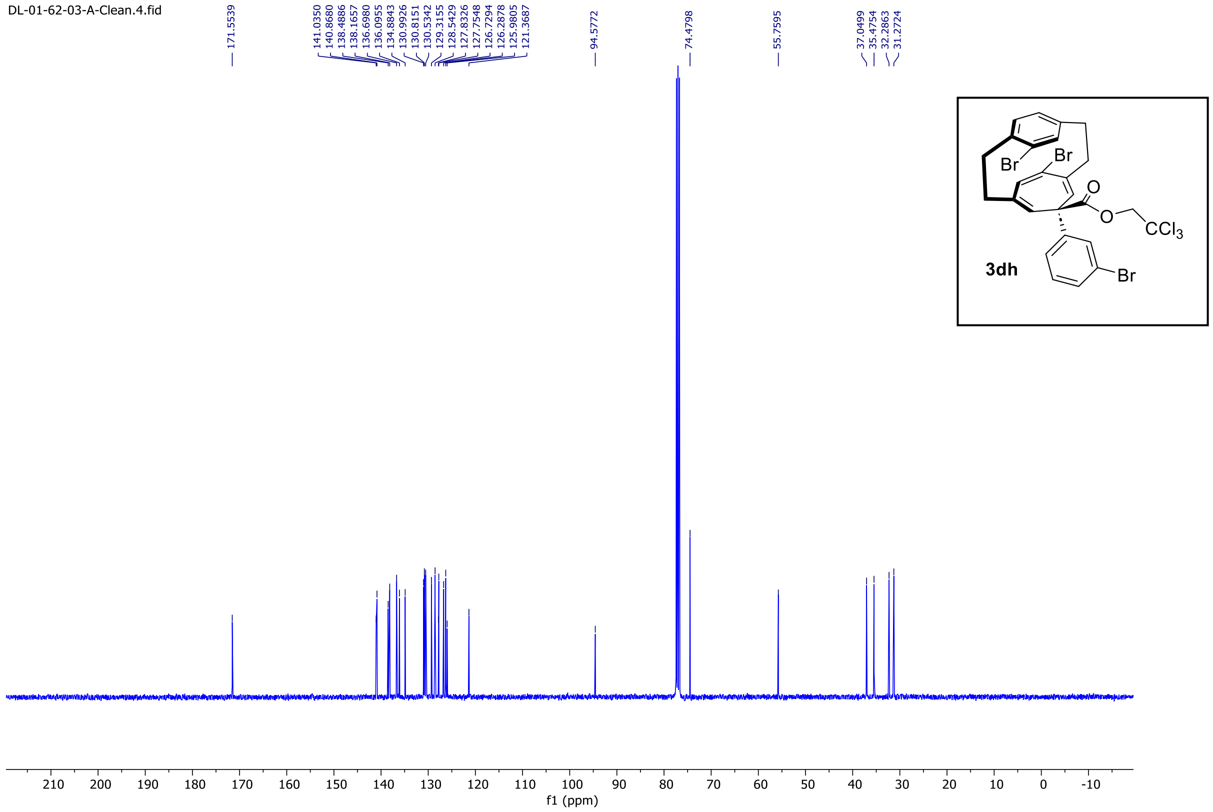

Figure S92. ^{13}C-NMR of 3dh

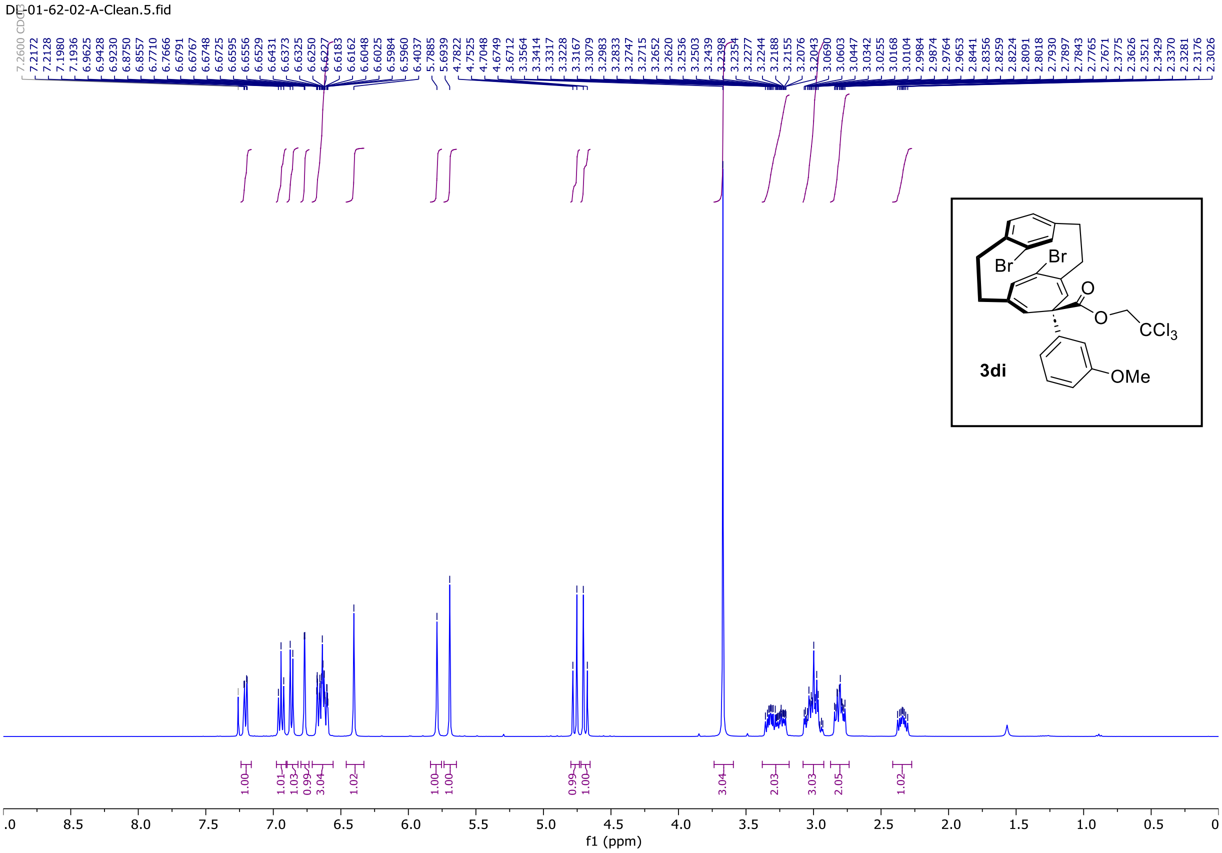

Figure S93. ^{1}**H-NMR of 3di**

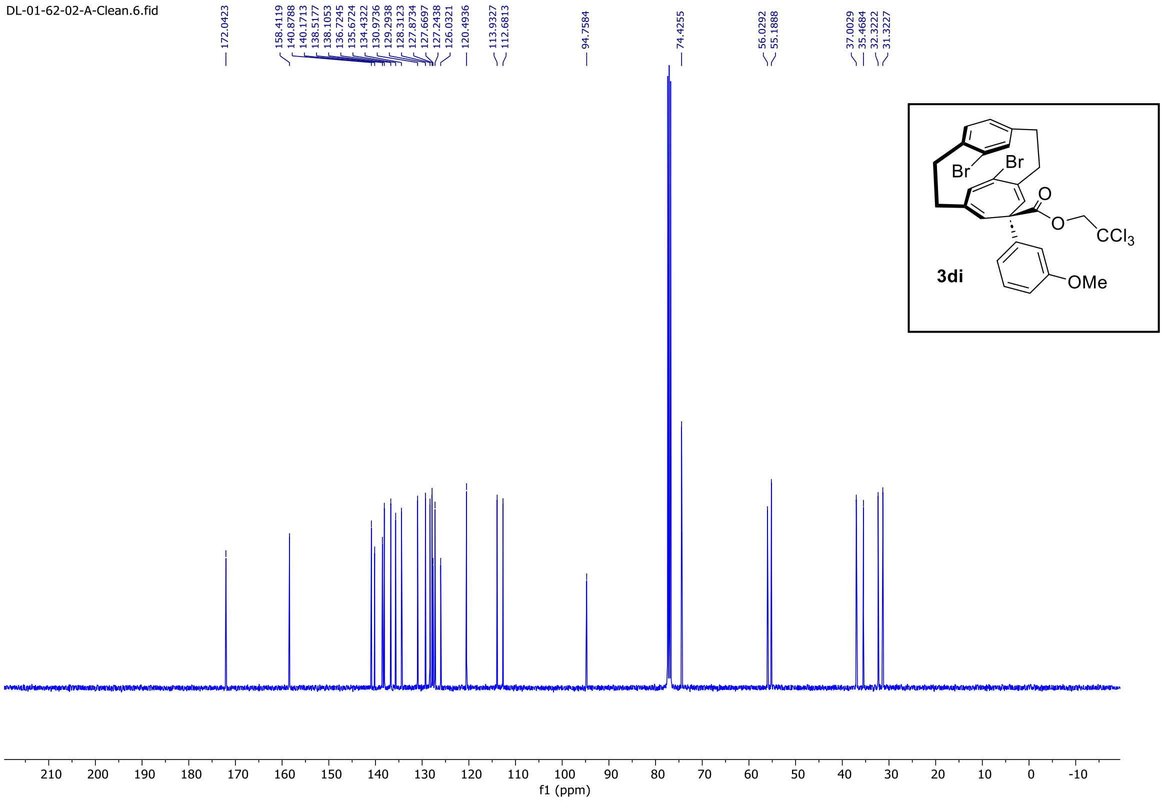

Figure S94. ^{13}**C-NMR of 3di**

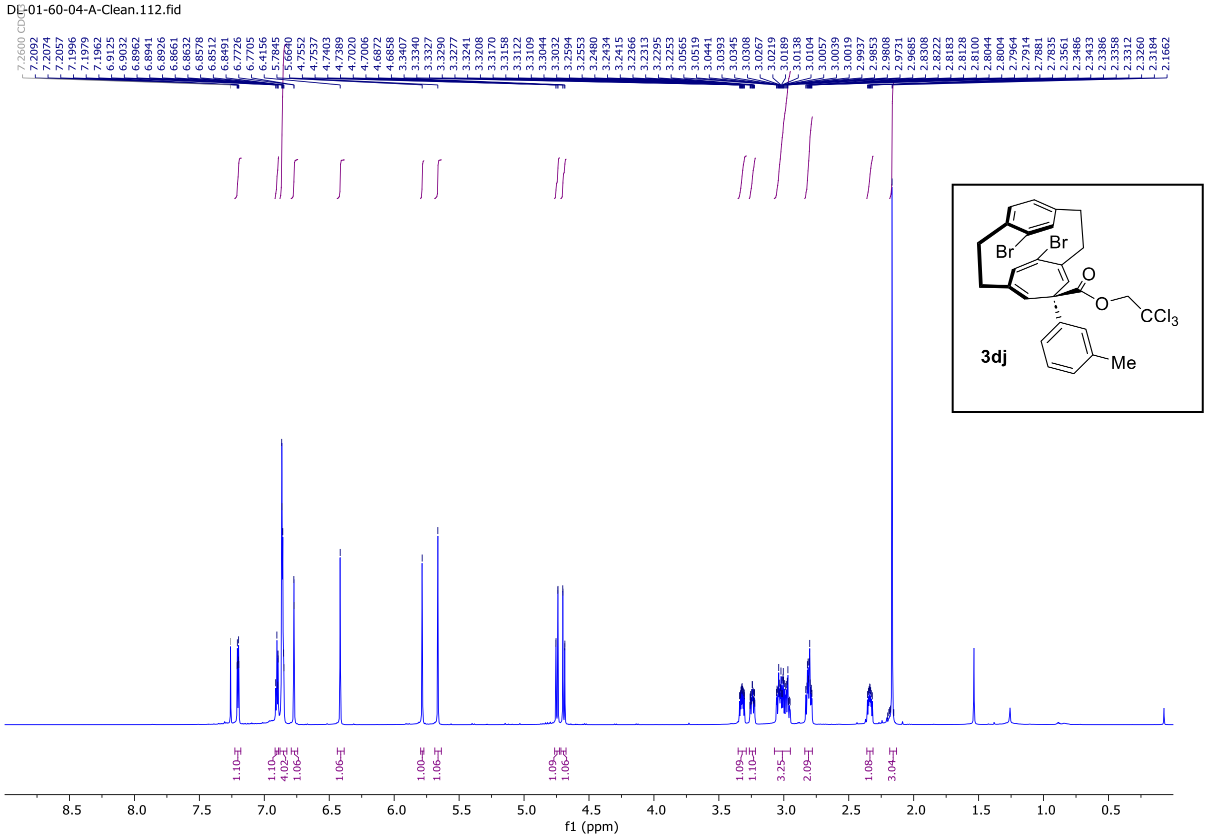

Figure S95. ^{1}H-NMR of 3dj

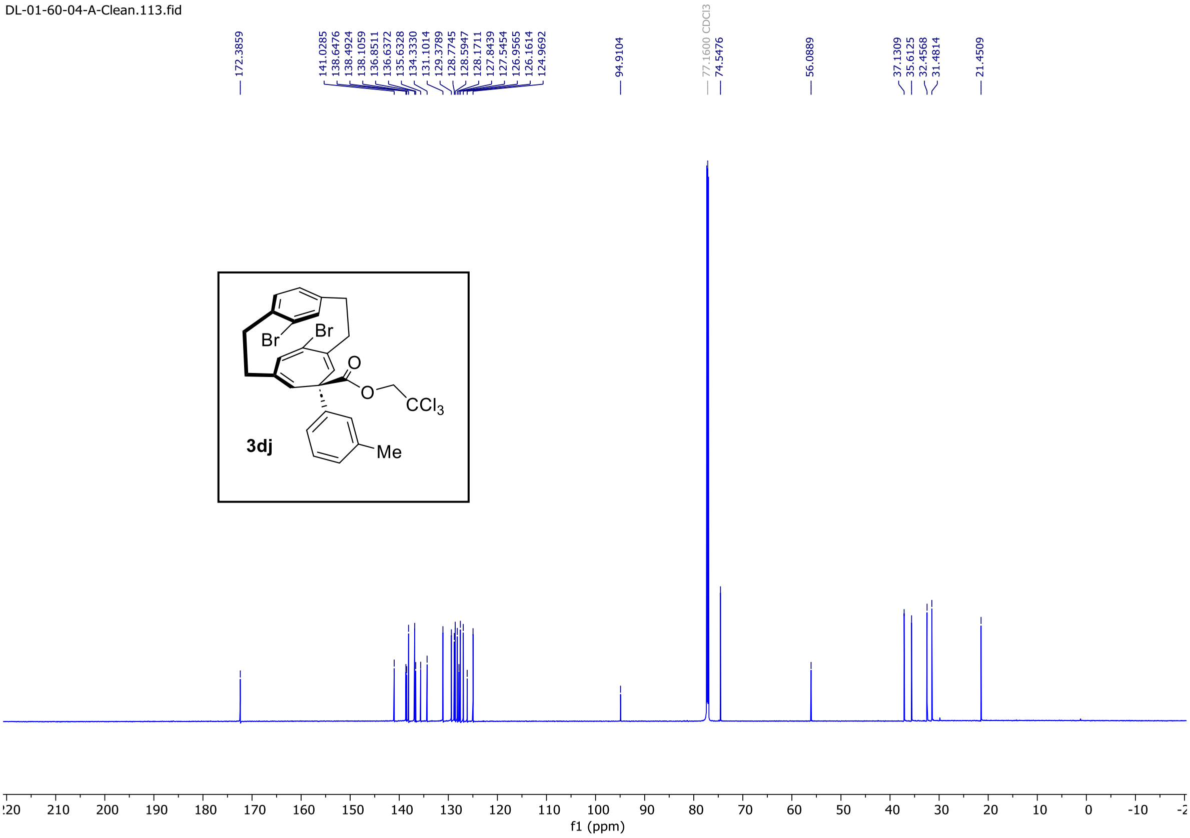

Figure S96. ^{13}C-NMR of 3dj

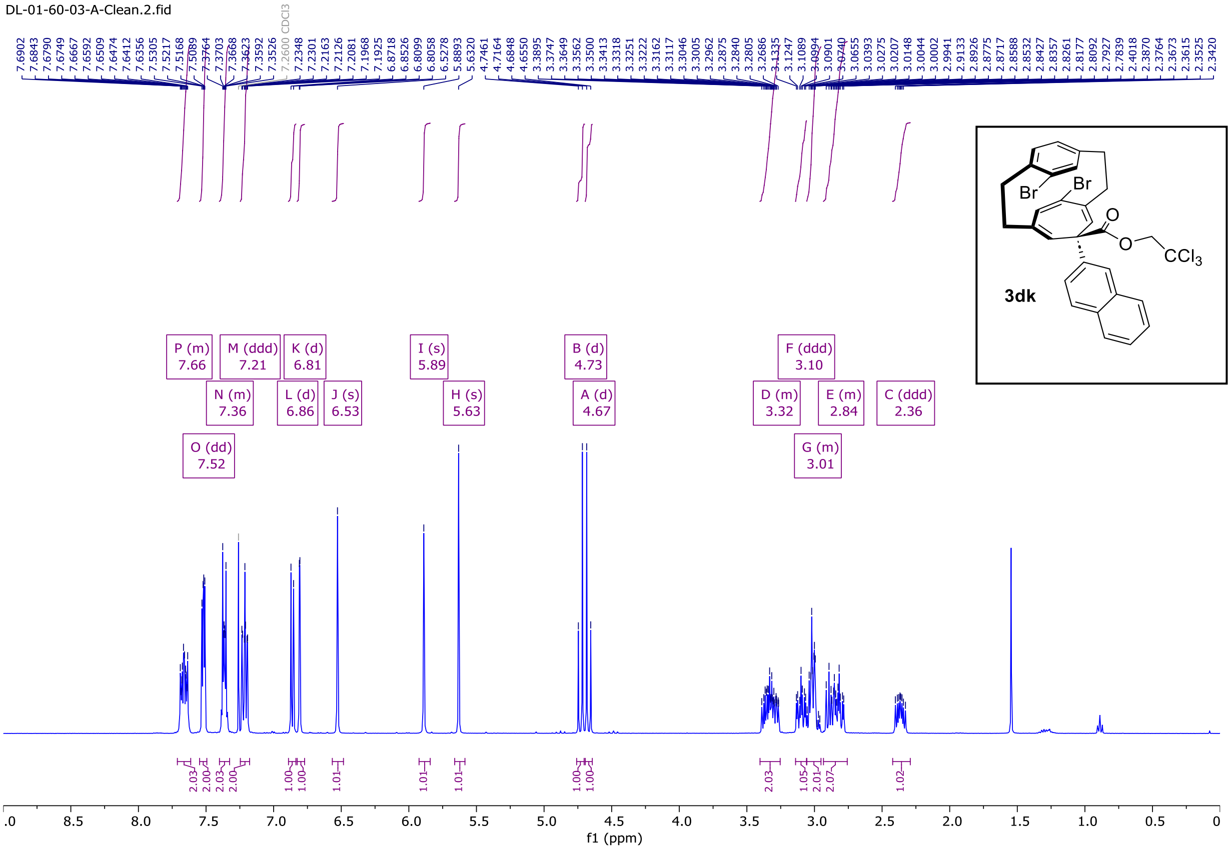

Figure S97. ^{1}H-NMR of 3dk

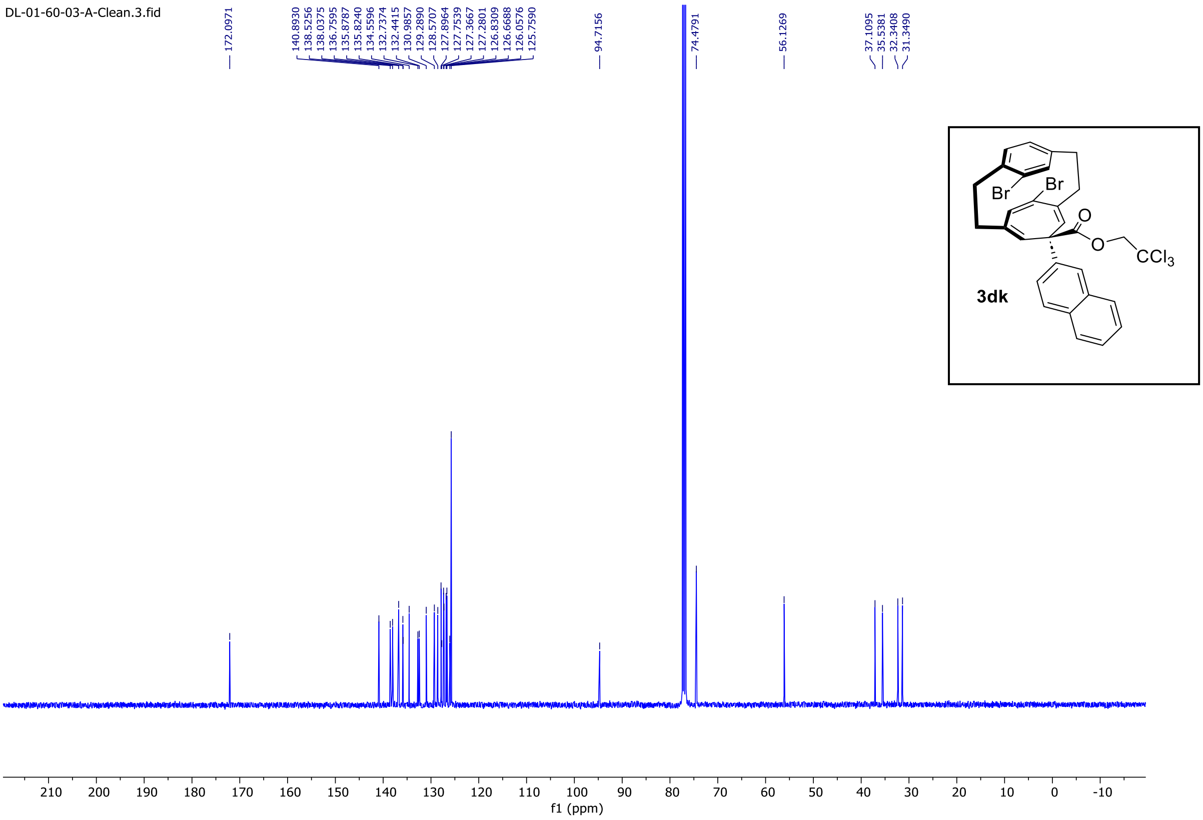

Figure S98. ^{13}C-NMR of 3dk

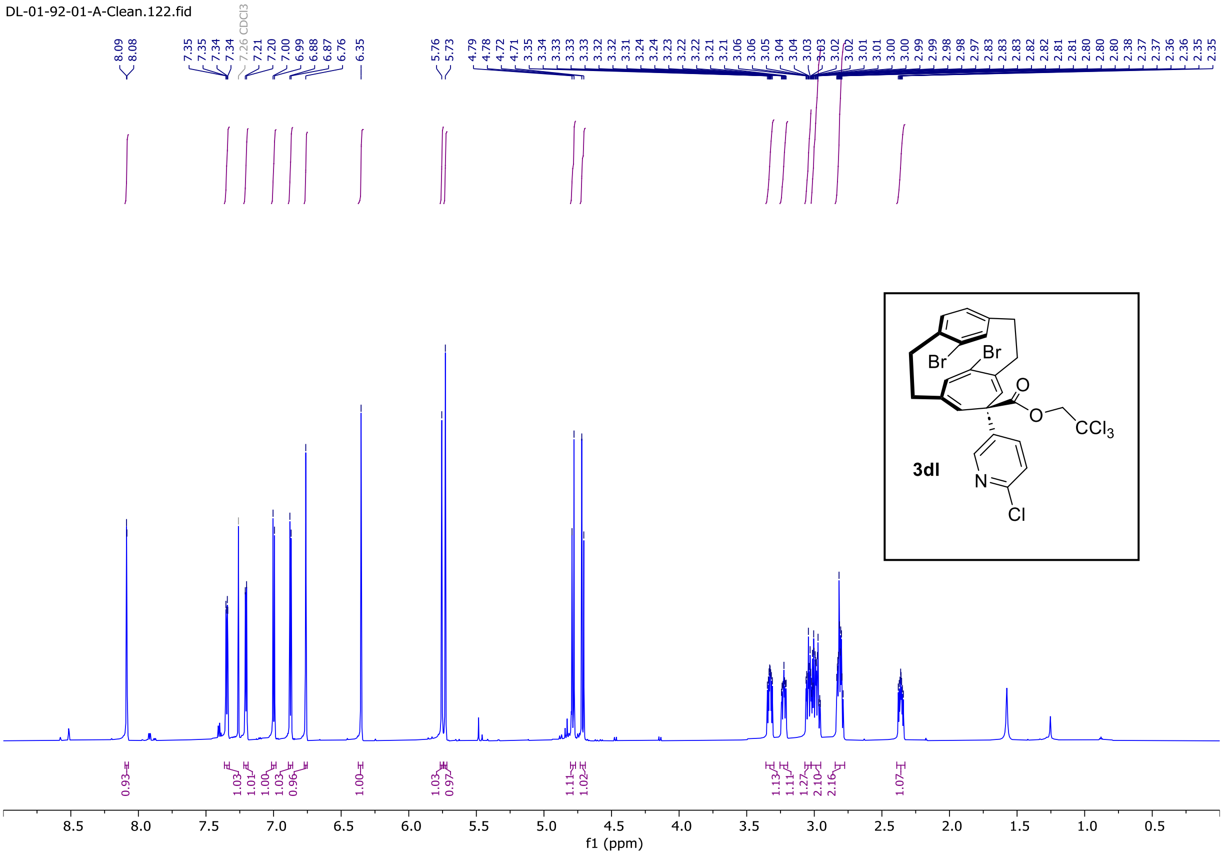

Figure S99. ^{1}H-NMR of 3dl

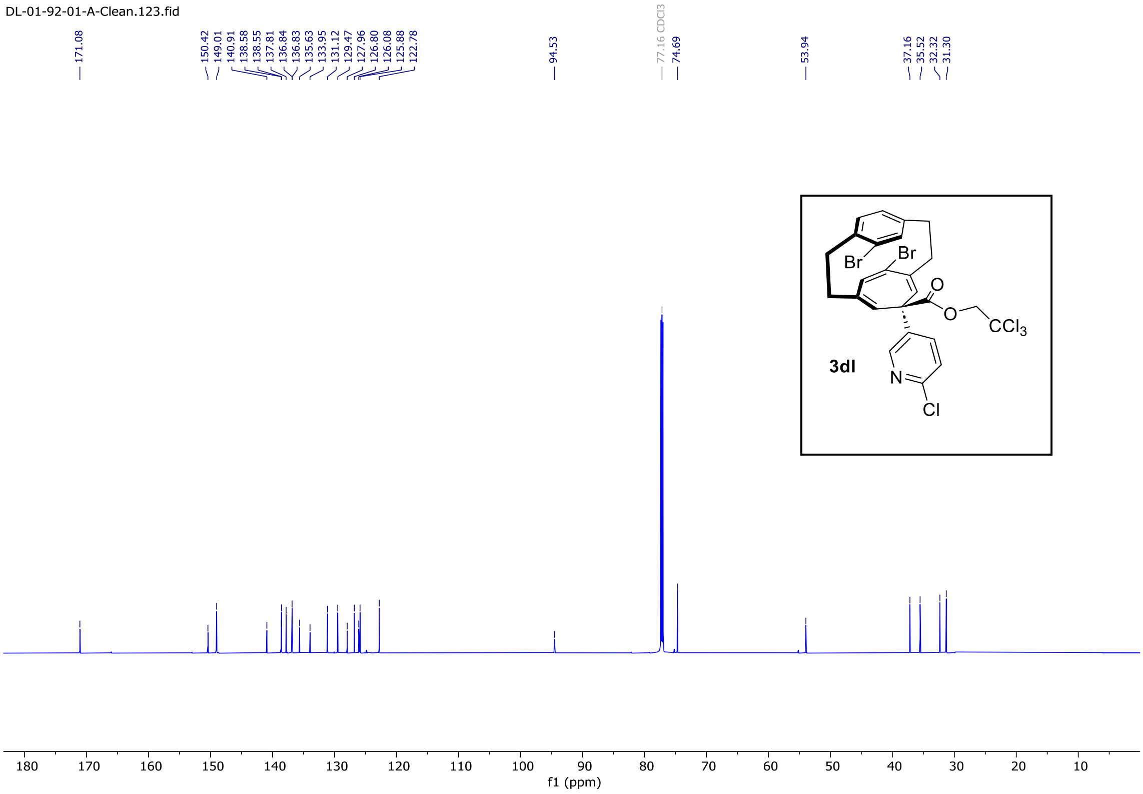

Figure S100. ^{13}C-NMR of 3dl

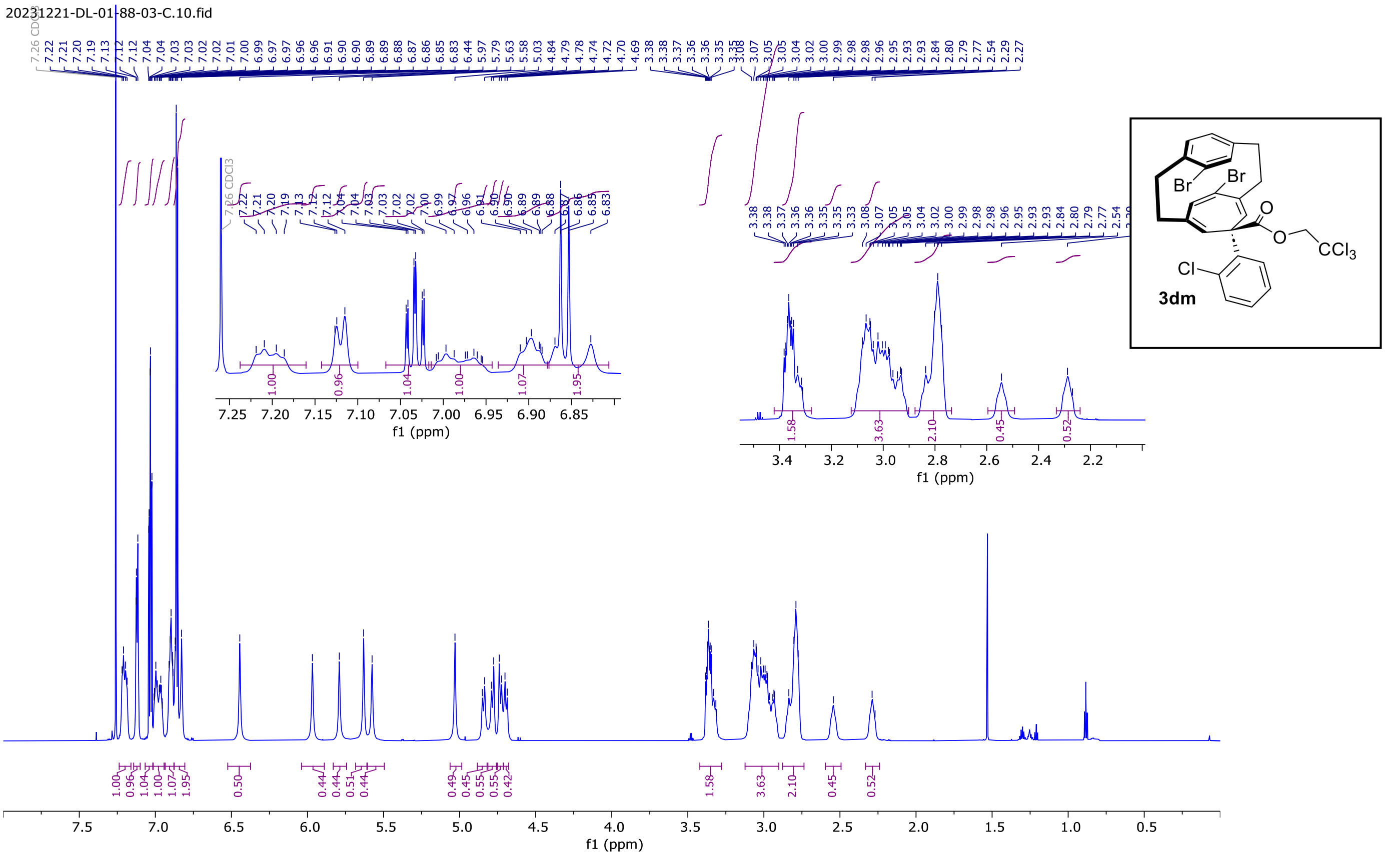

Figure S101. ^{1}H-NMR of 3dm

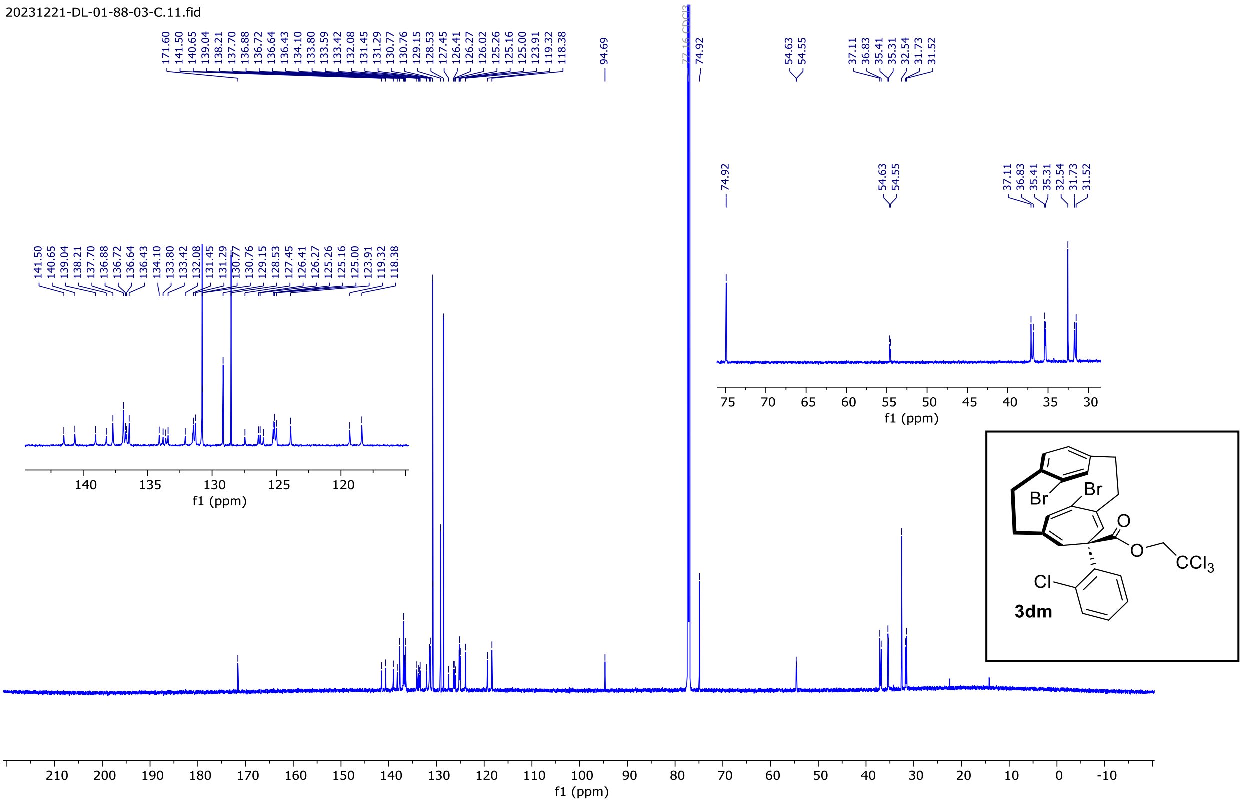

Figure S102. ^{13}C-NMR of 3dm

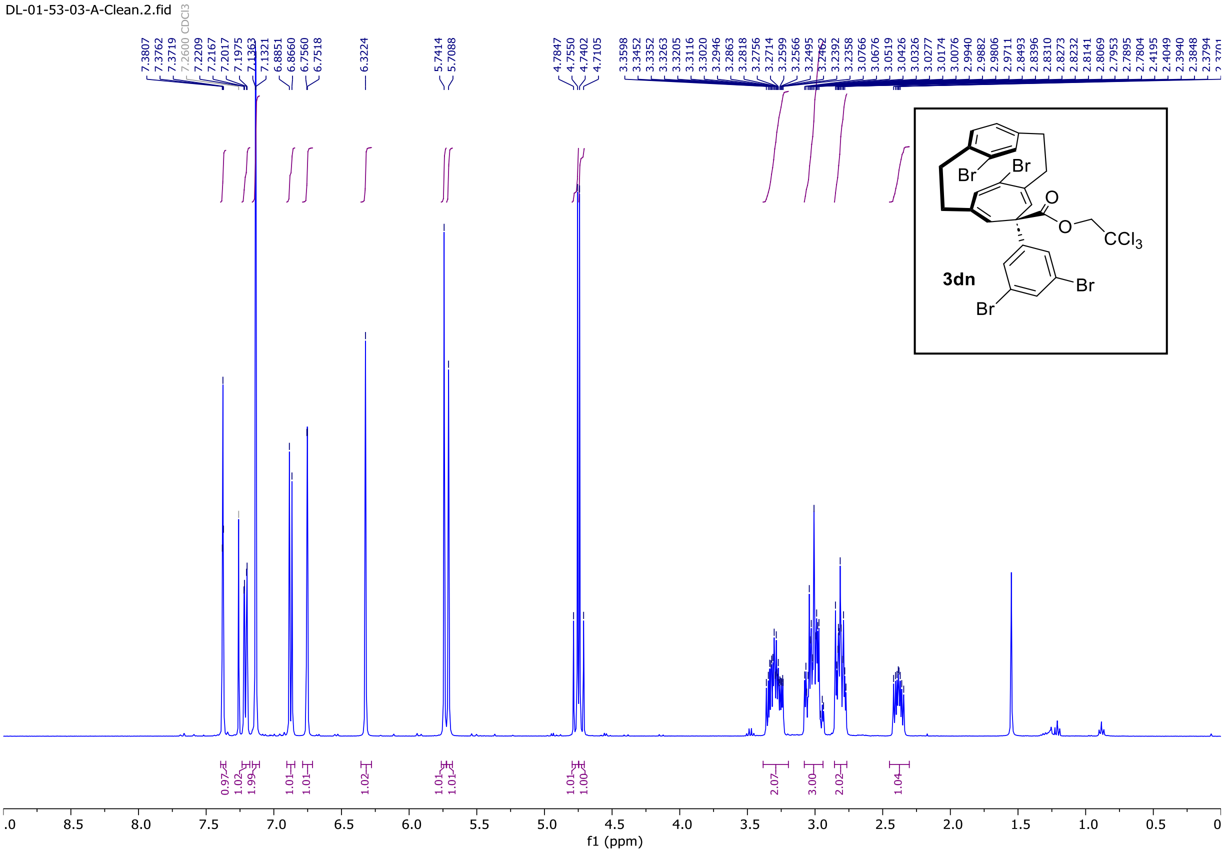

Figure S103. ^{1}H-NMR of 3dn

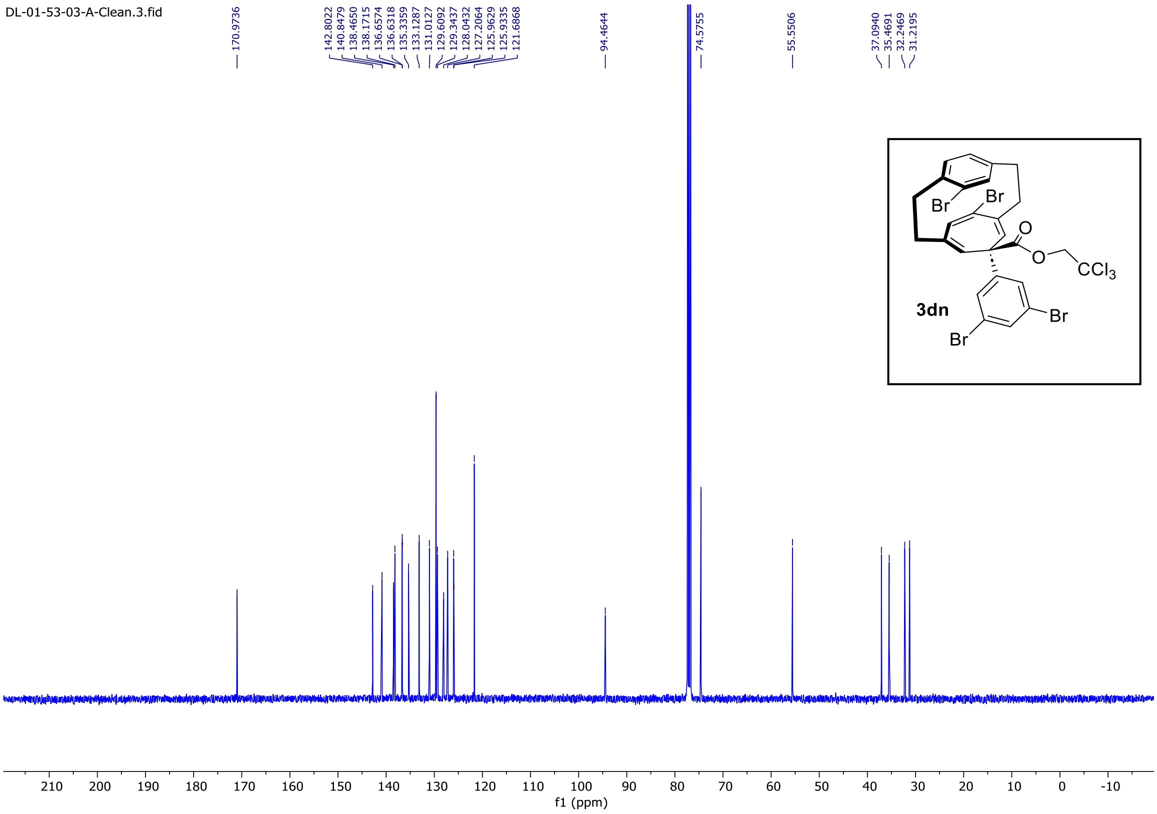

Figure S104. ^{13}C-NMR of 3dn

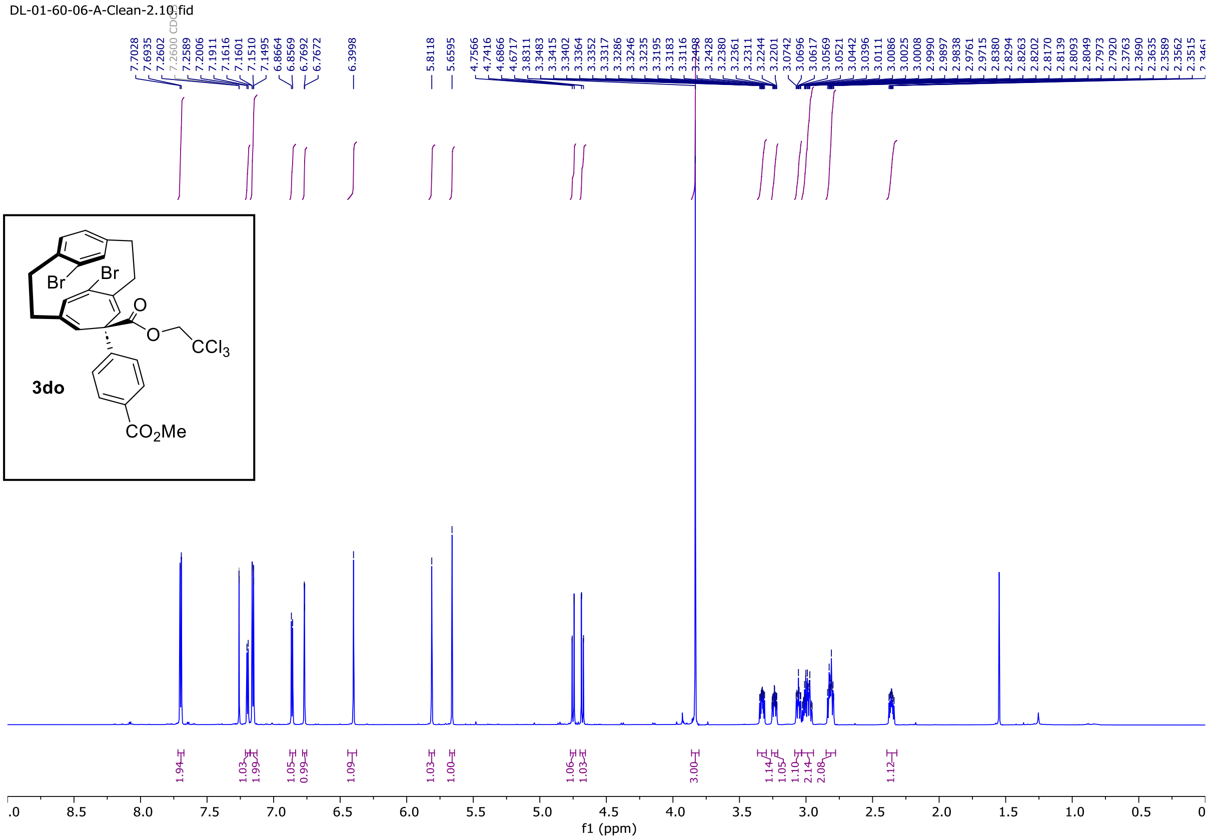

Figure S105. ^{1}H-NMR of 3do

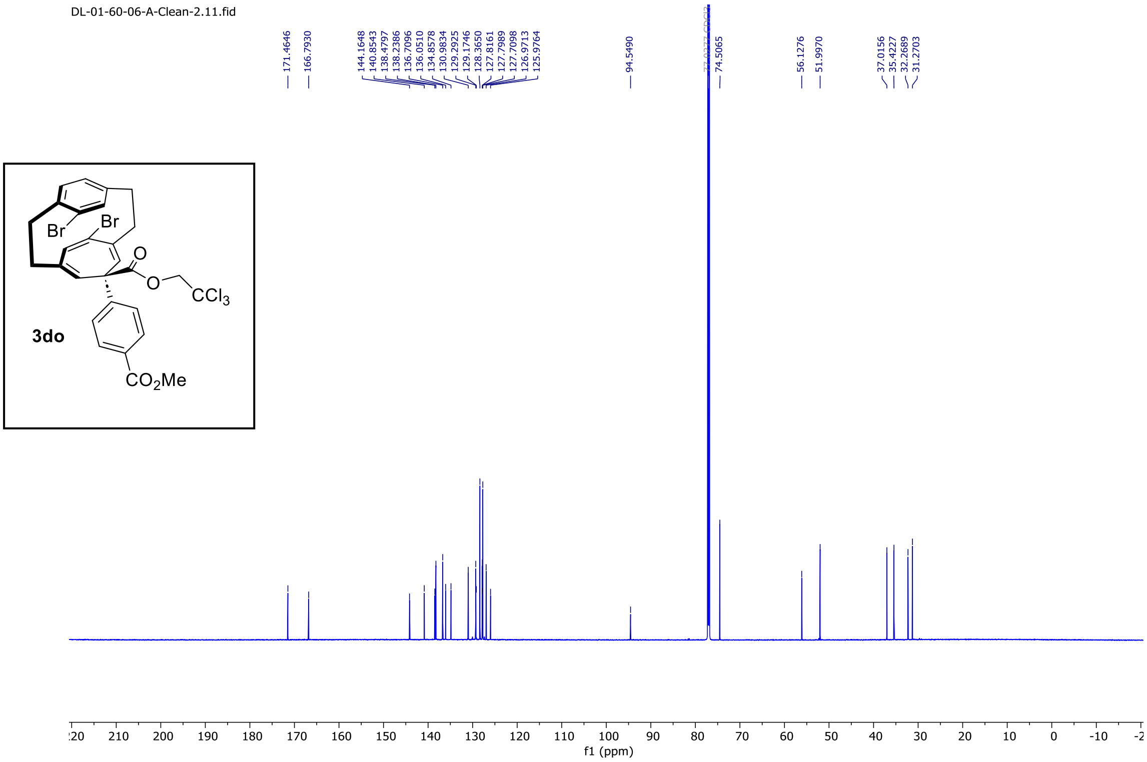

Figure S106. ^{13}C-NMR of 3do

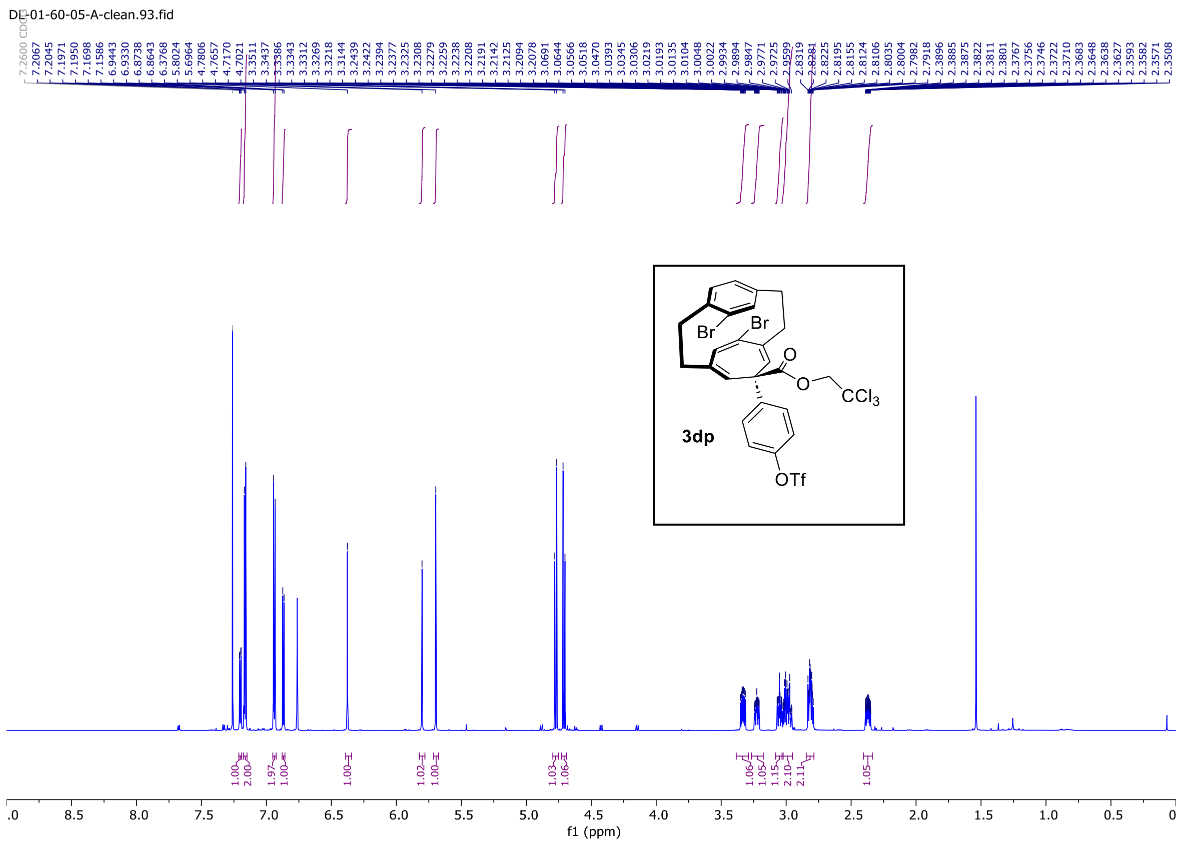

Figure S107. ^{1}H-NMR of 3dp

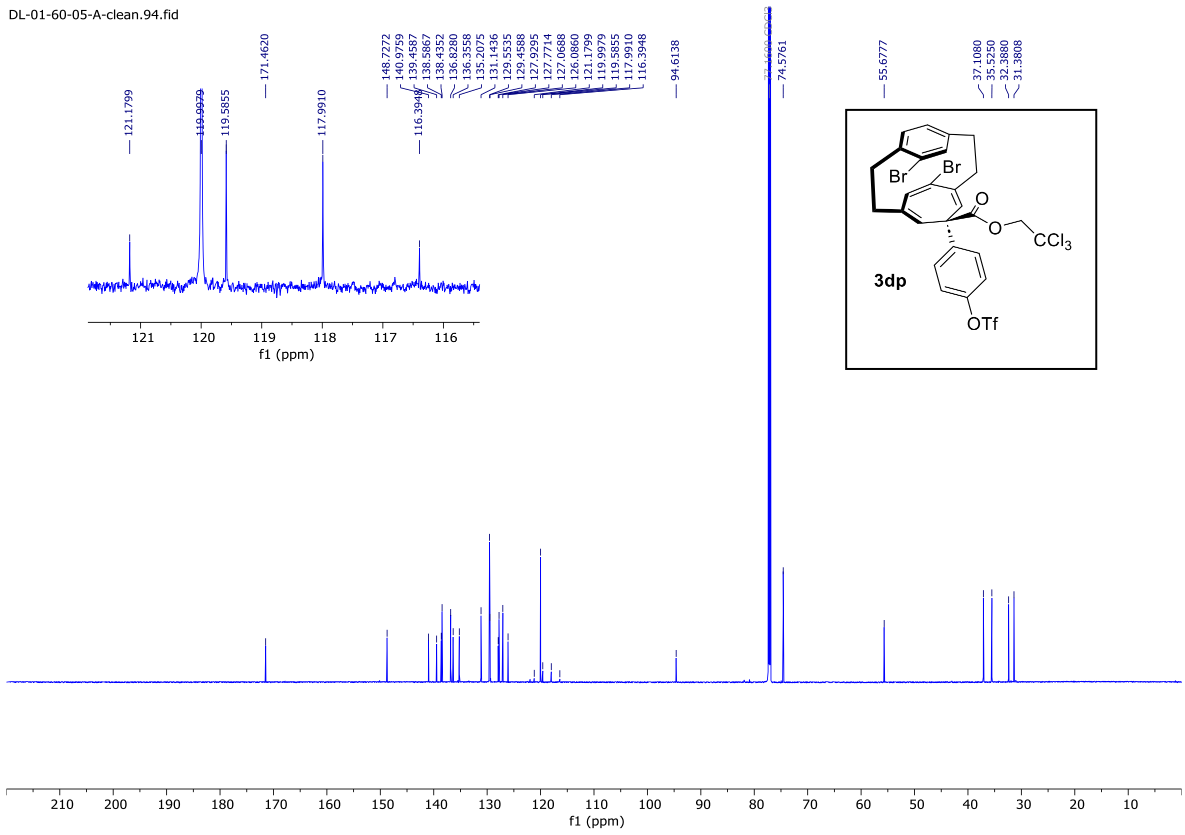

Figure S108. ^{13}C-NMR of 3dp

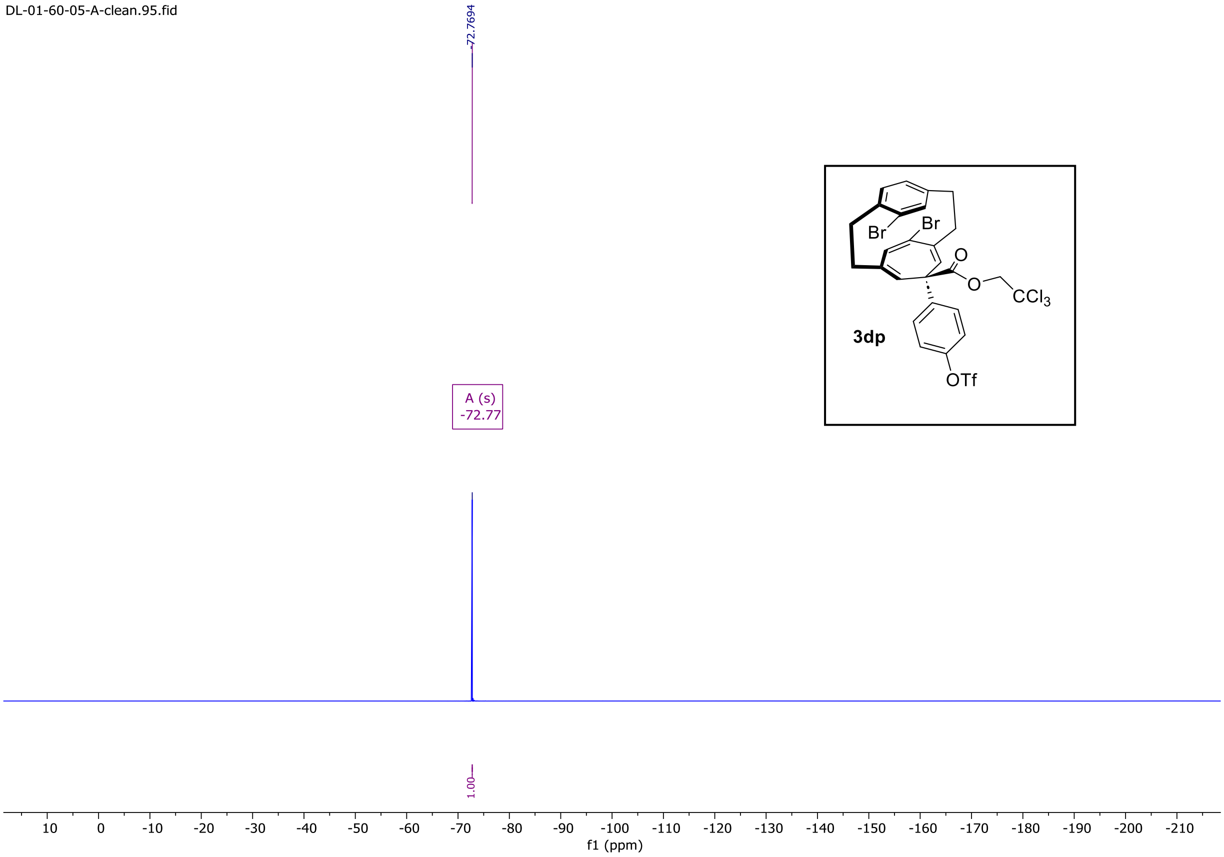

Figure S109. ^{19}F-NMR of 3dp

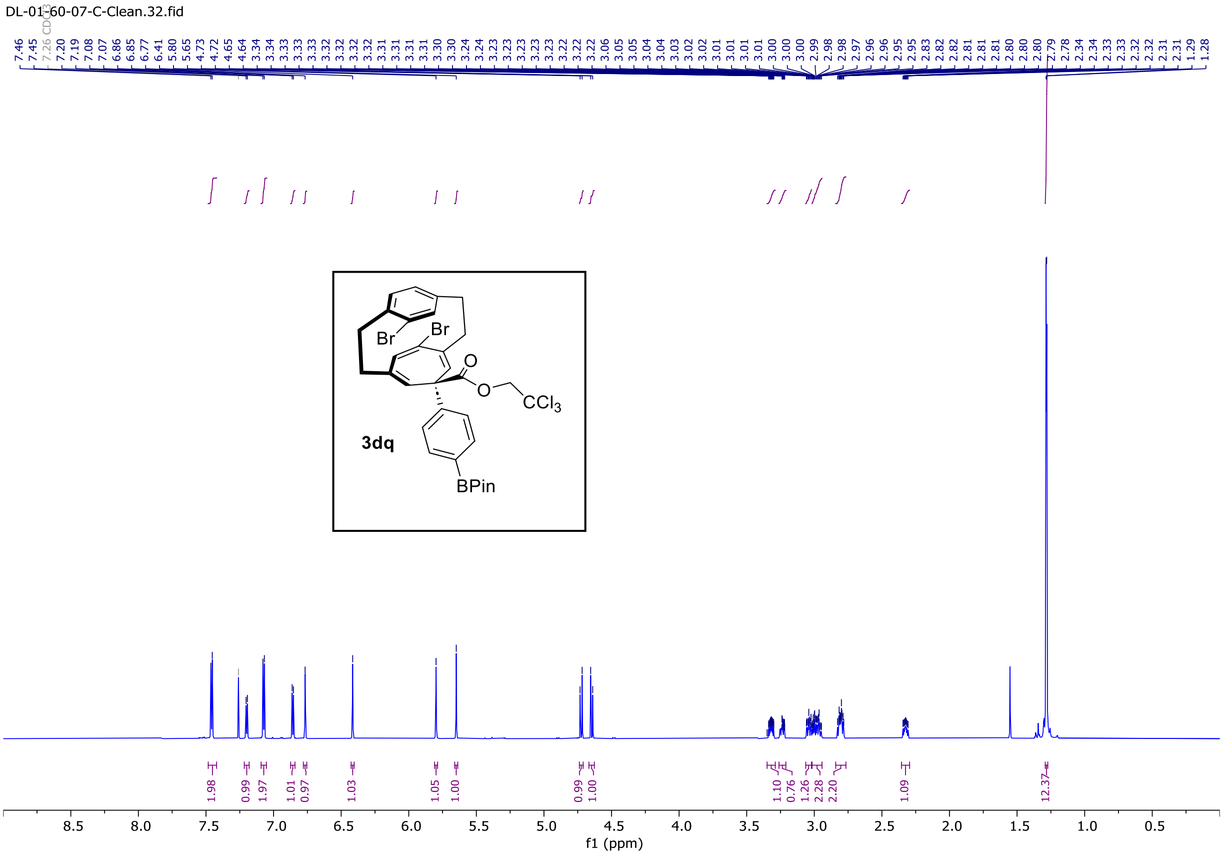

Figure S110. ^{1}**H-NMR of 3dq**

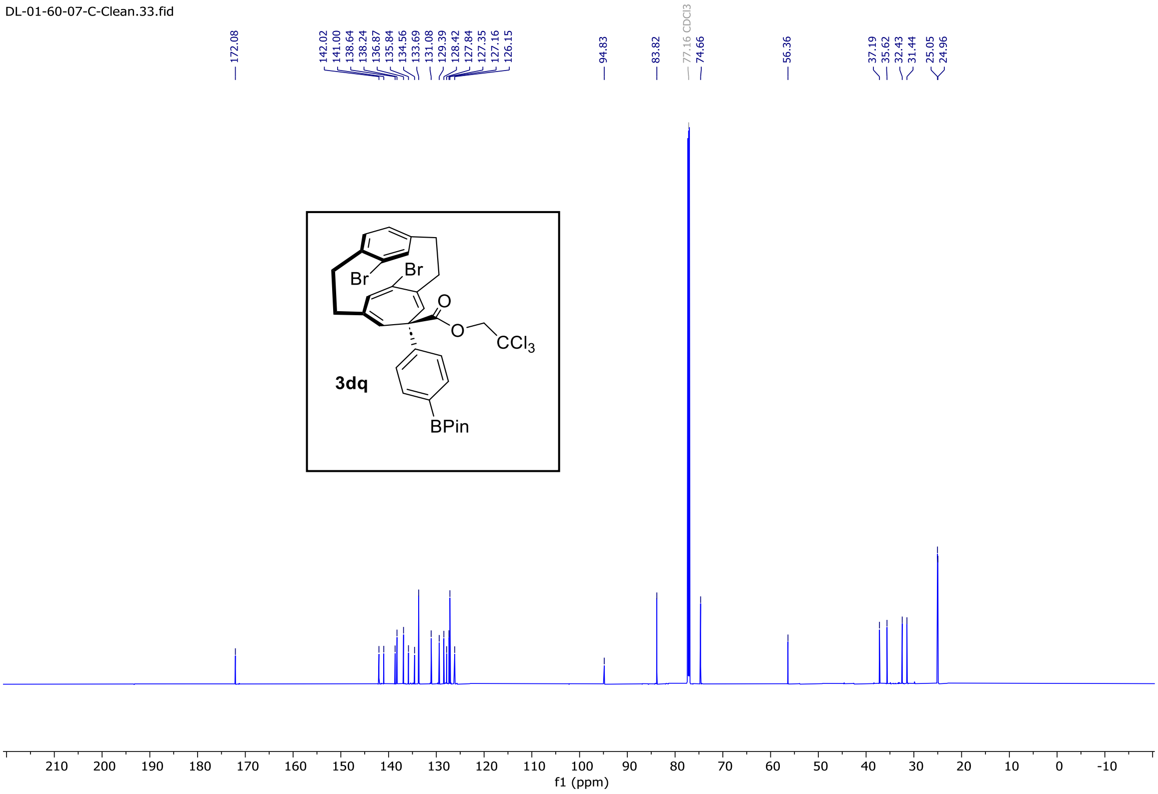

Figure S111. ^{13}**C-NMR of 3dq**

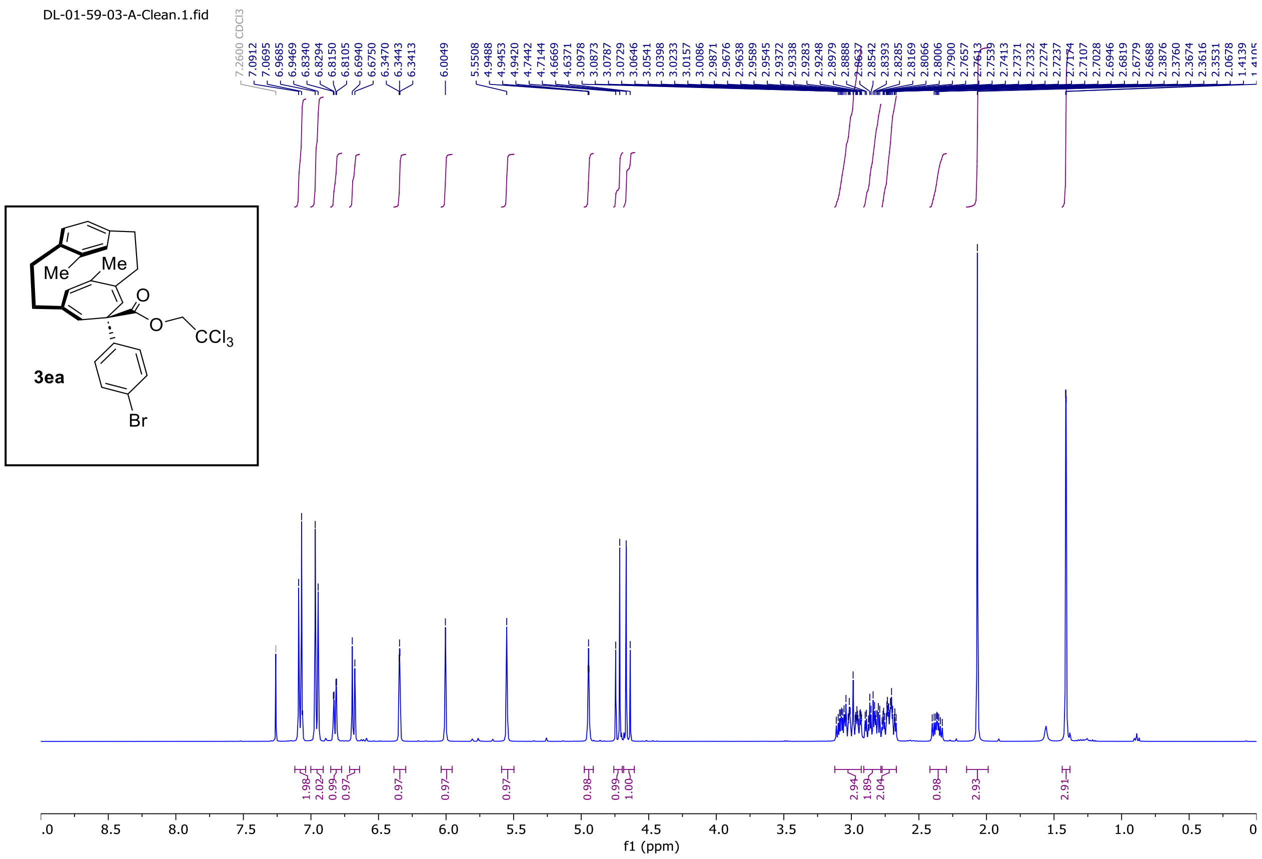

Figure S112. ^{1}H-NMR of 3ea

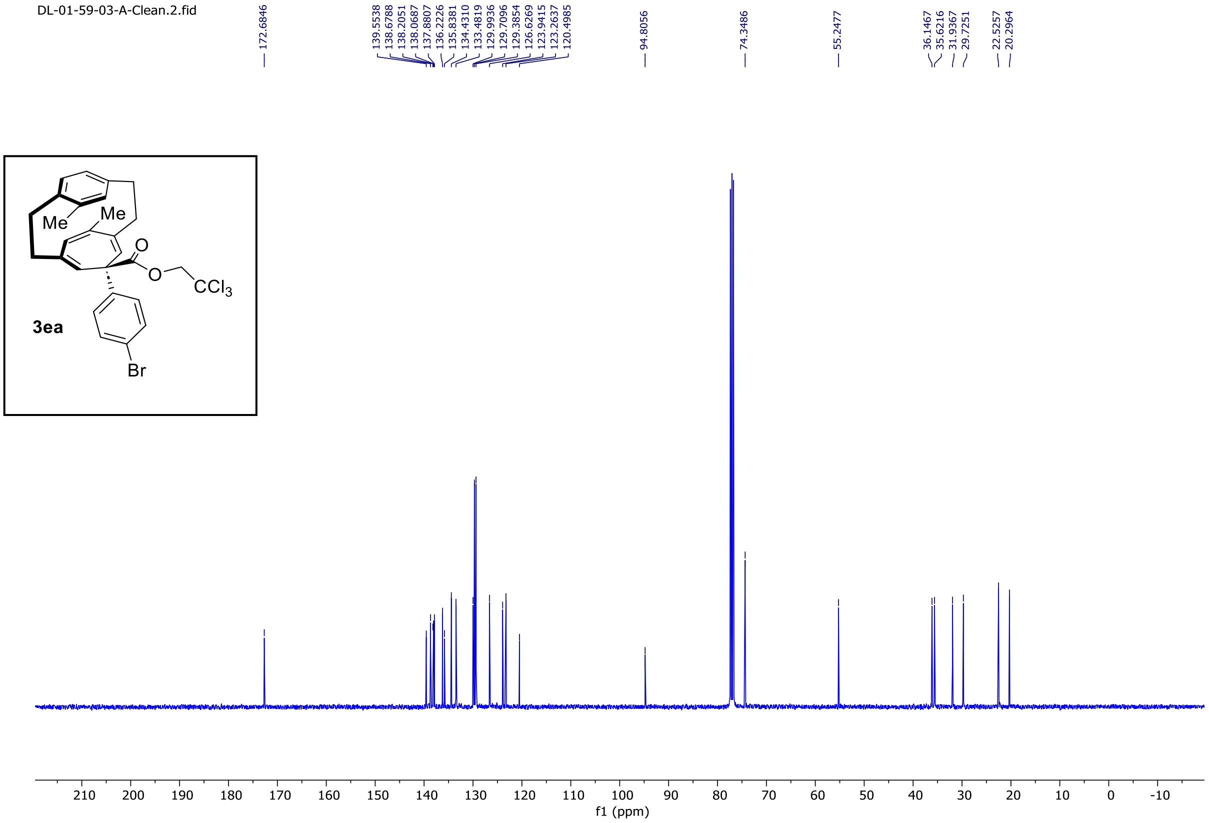

Figure S113. ^{13}C-NMR of 3ea

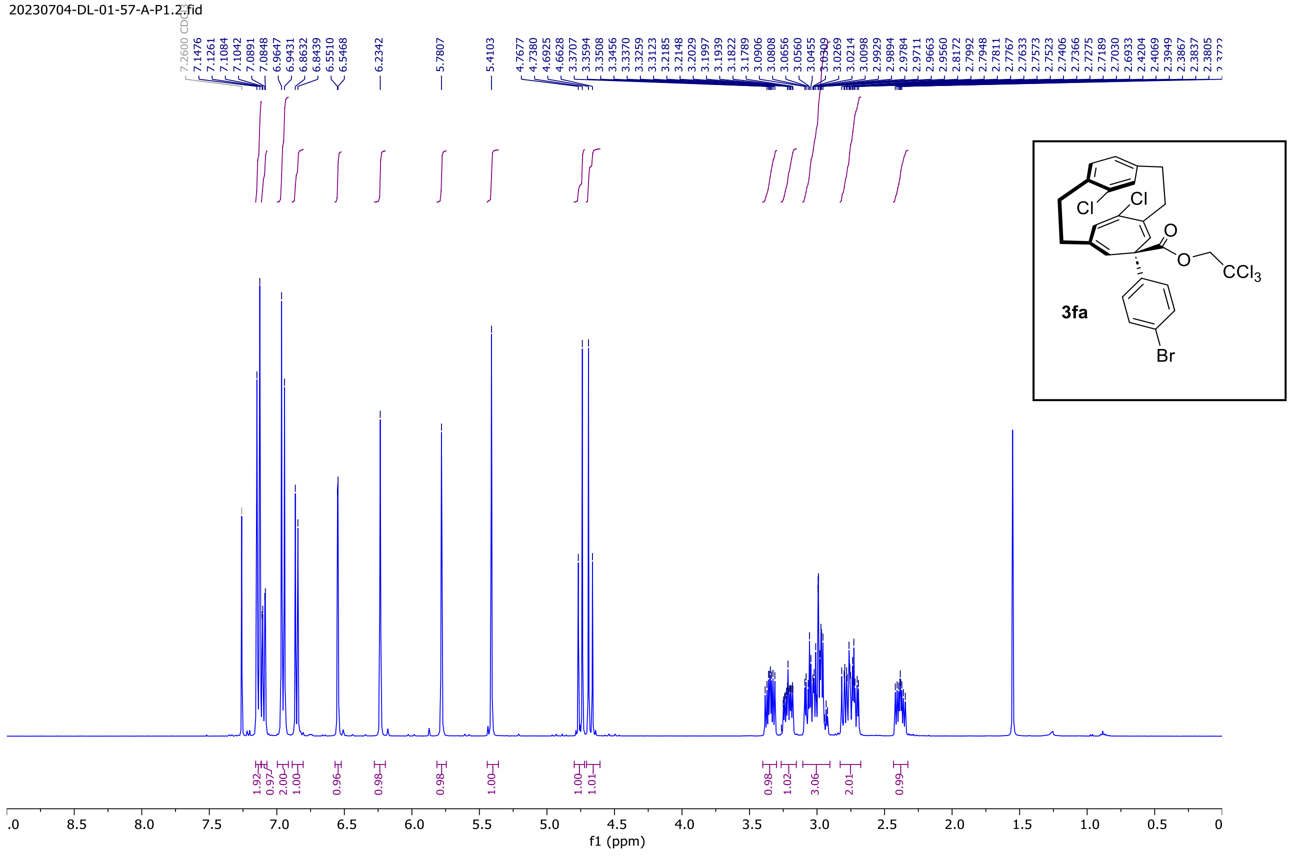

Figure S114. ^{1}H-NMR of 3fa

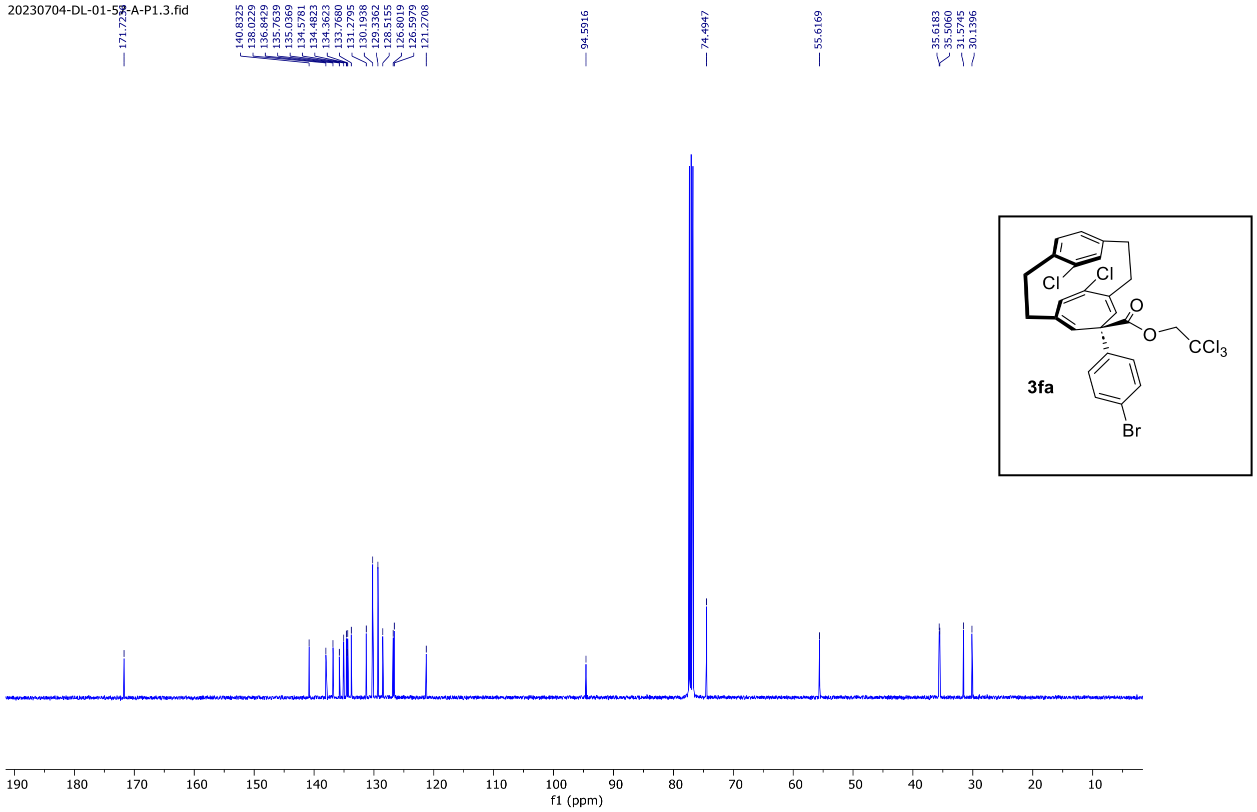

Figure S115. ^{13}C-NMR of 3fa

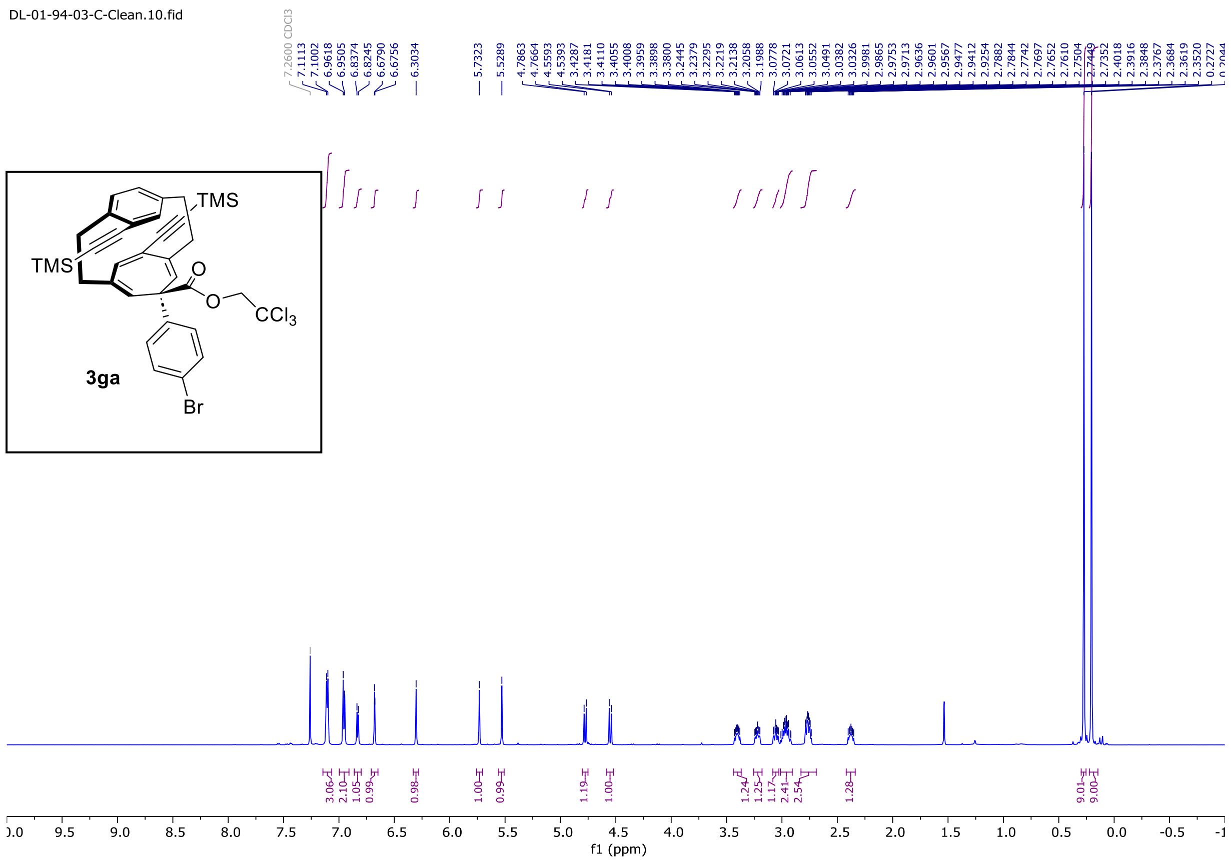

Figure S116. ^{1}H-NMR of 3ga

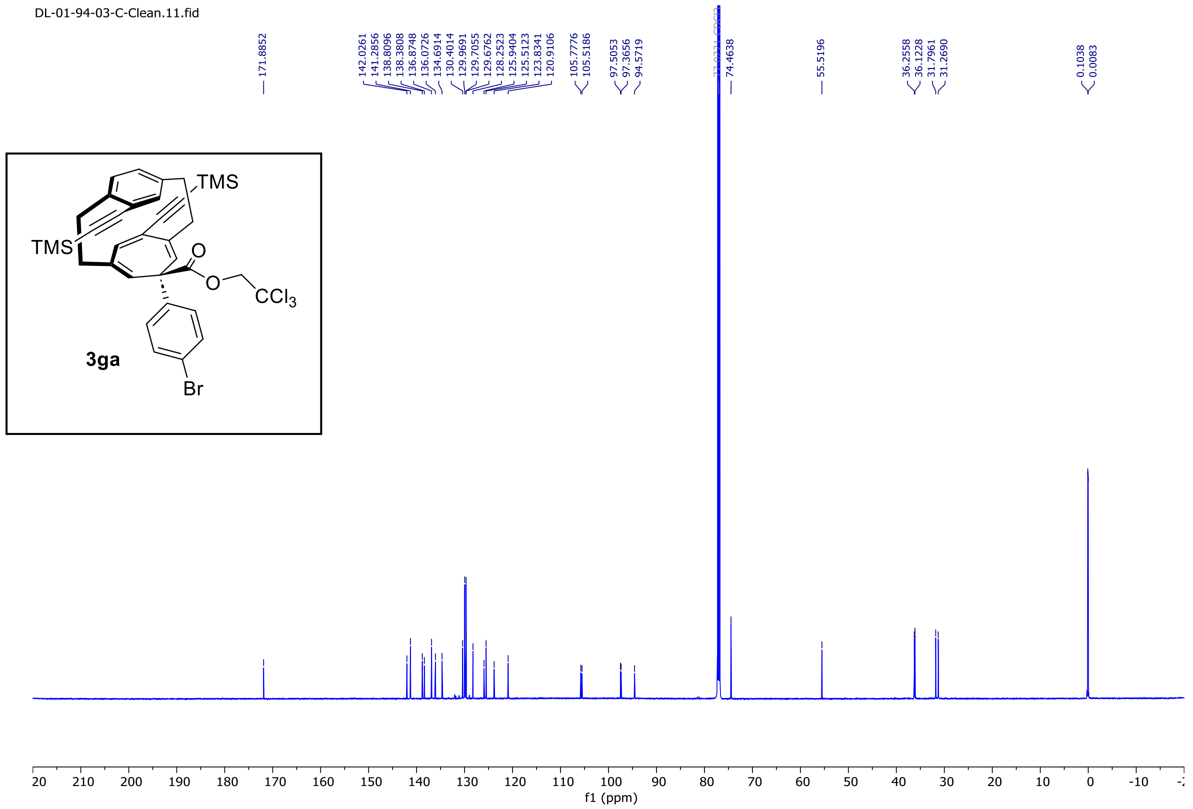

Figure S117. ^{13}C-NMR of 3ga

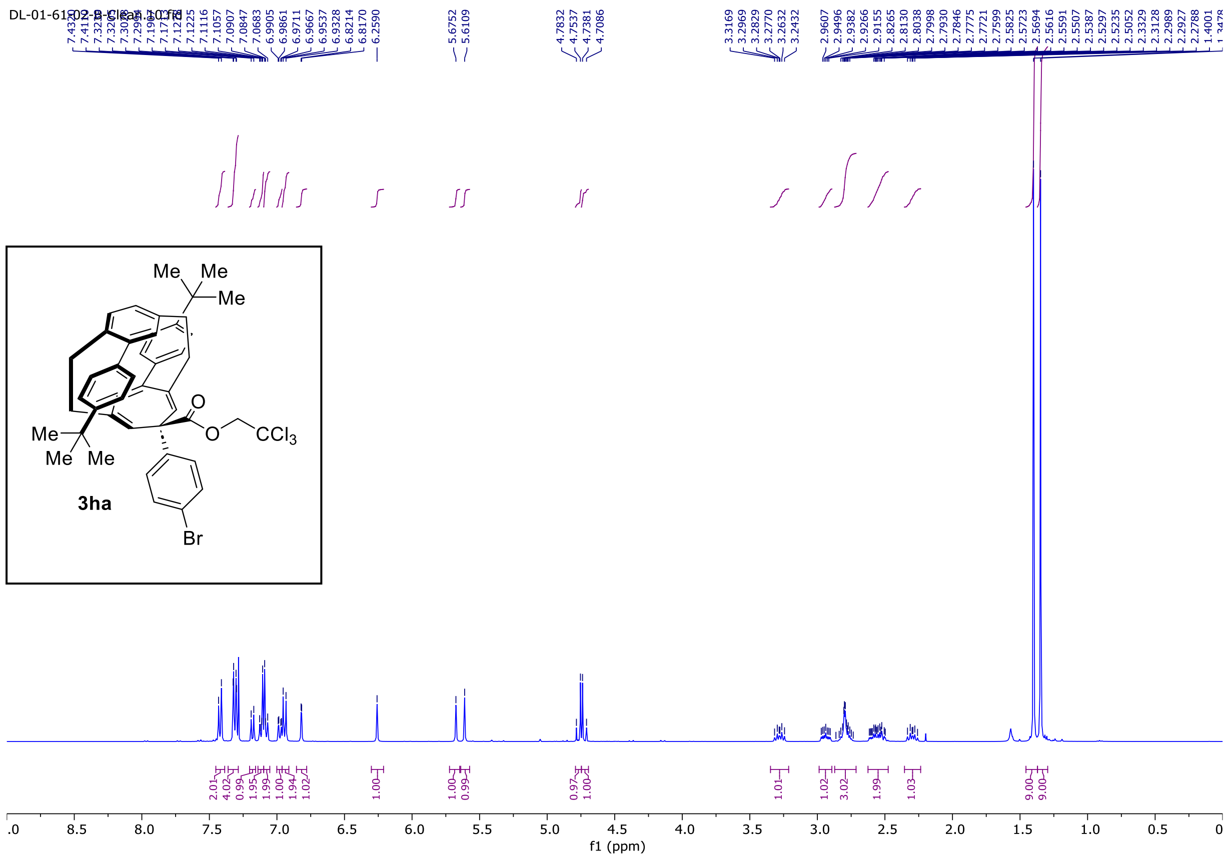

Figure S118. ^{1}H-NMR of 3ha

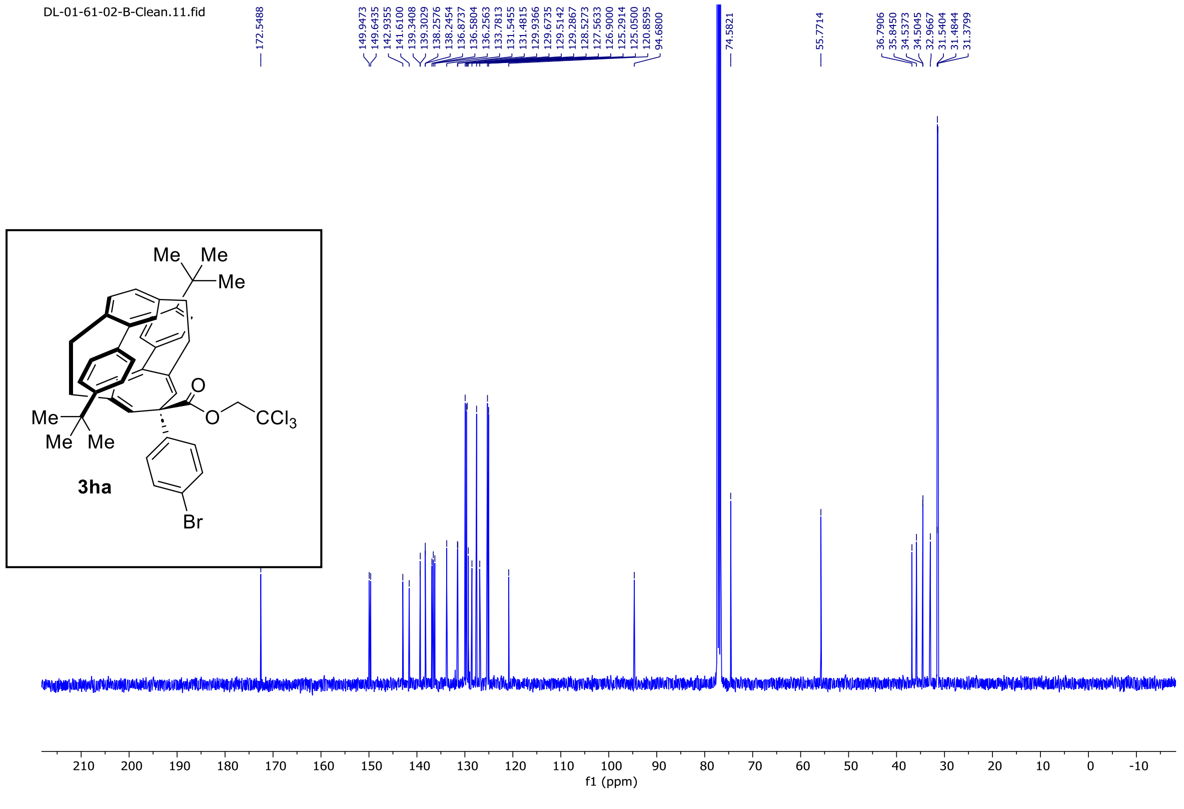

Figure S119. ^{13}C-NMR of 3ha

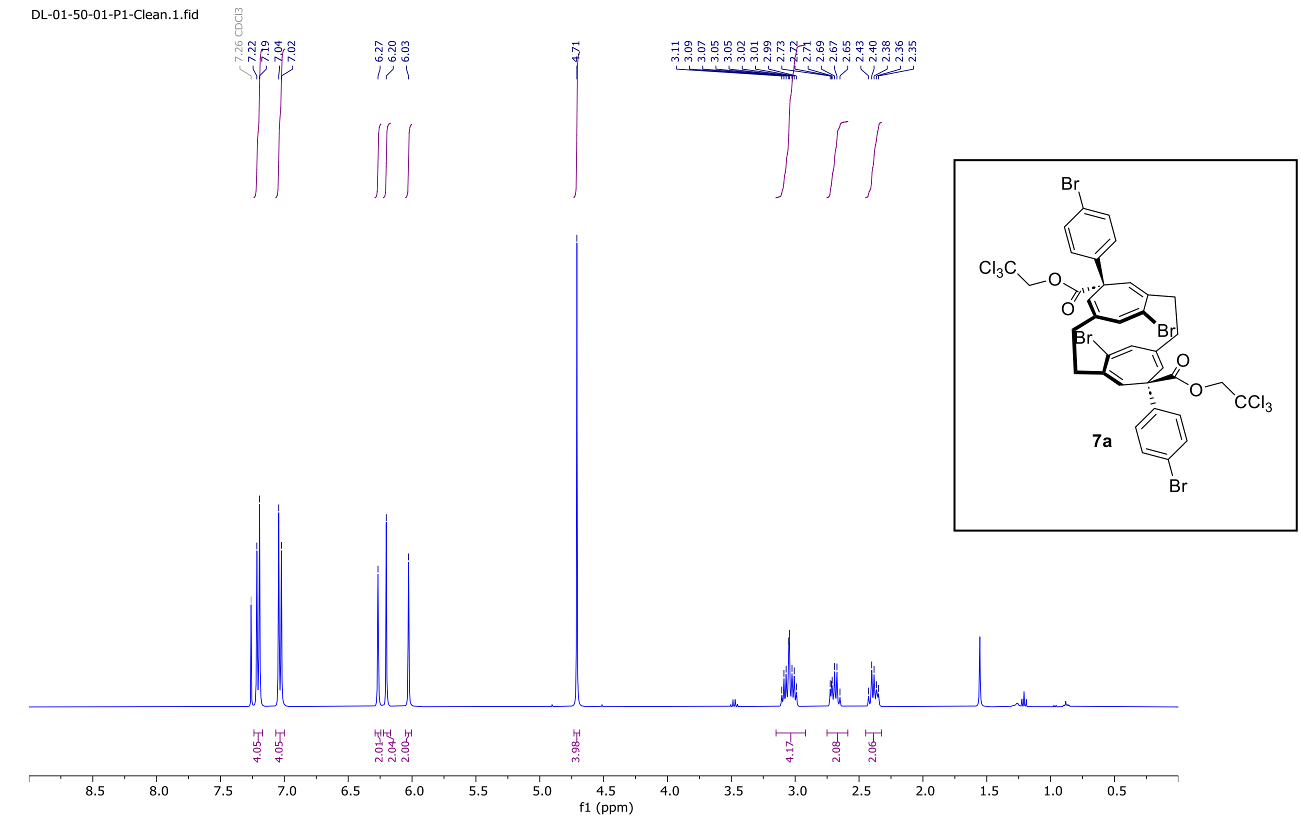

Figure S120. ^{1}**H-NMR of 7a**

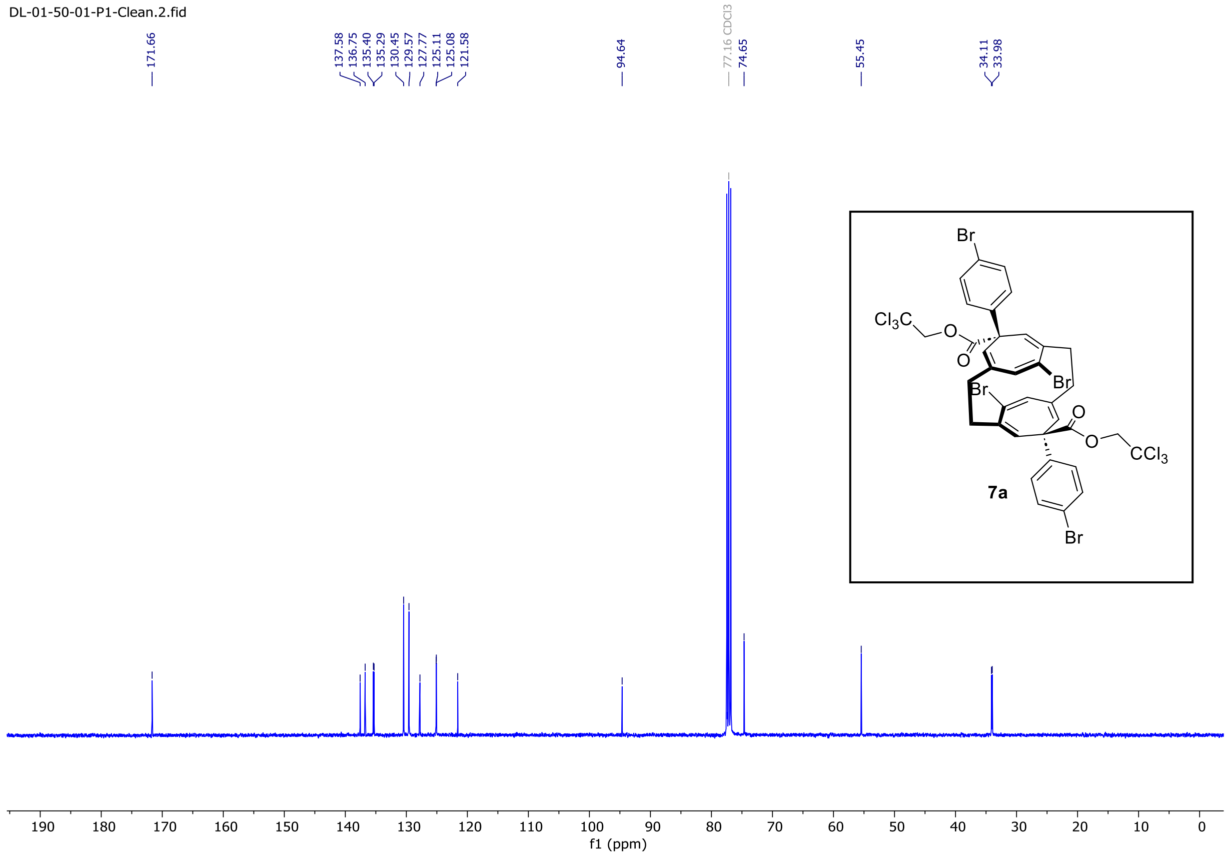

Figure S121. ^{13}**C-NMR of 7a**

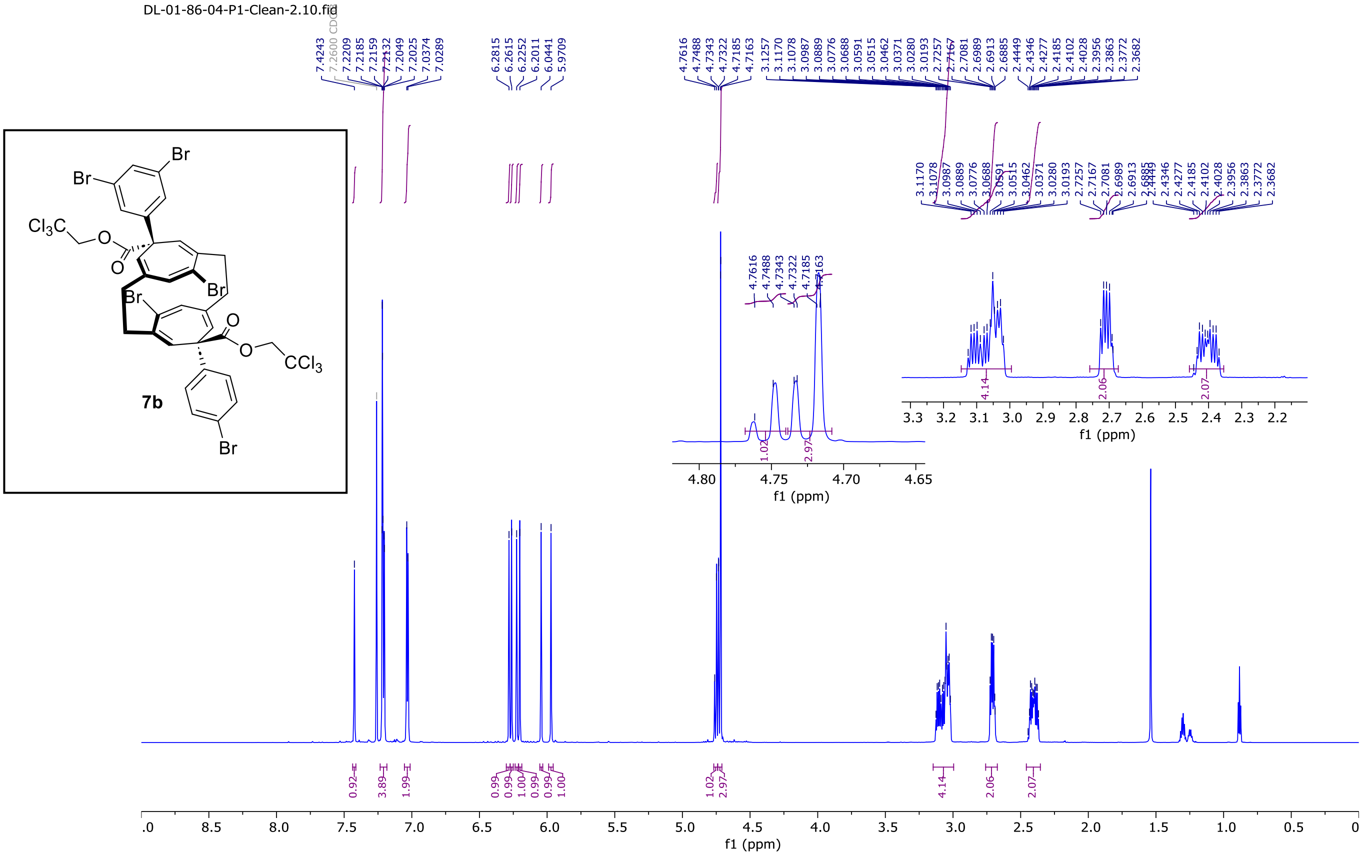

Figure S122. ^{1}**H-NMR of 7b**

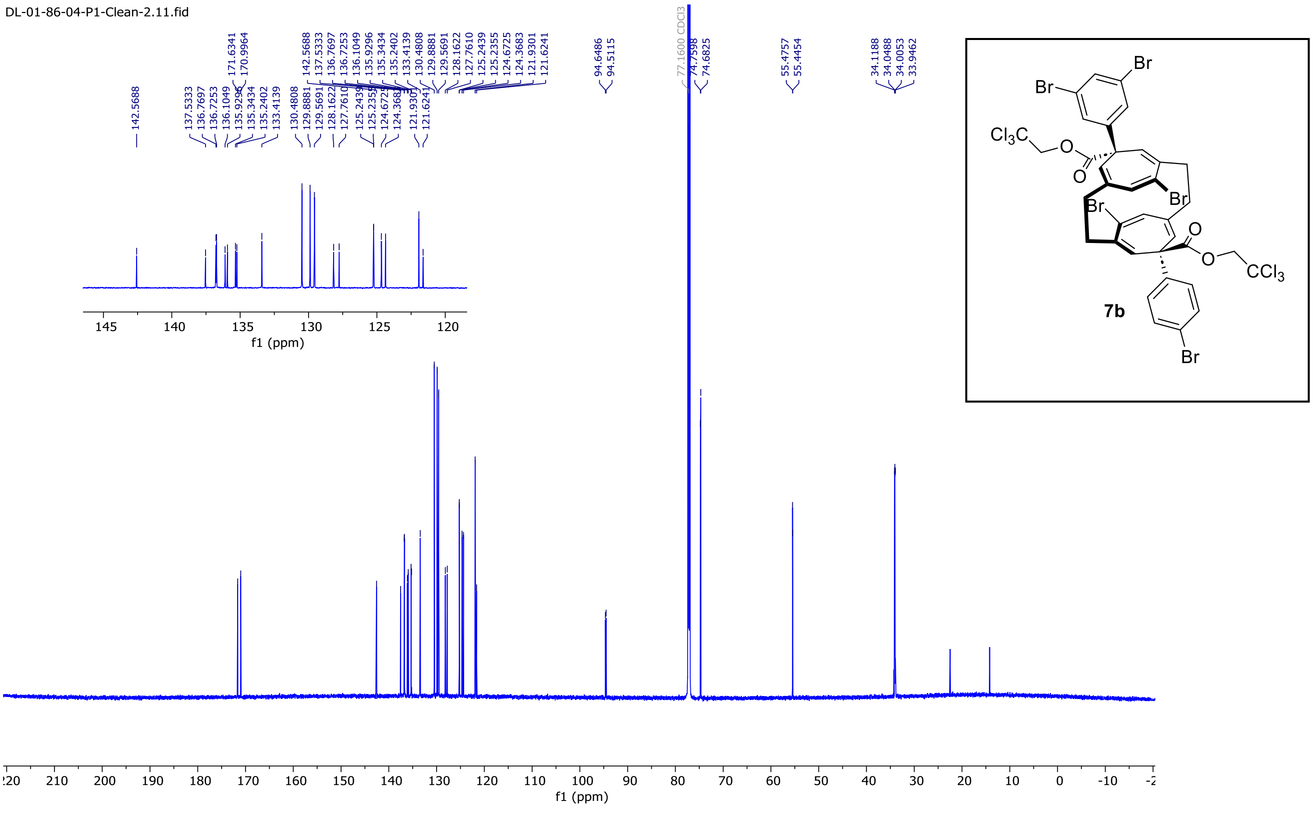

Figure S123. ^{13}**C-NMR of 7b**

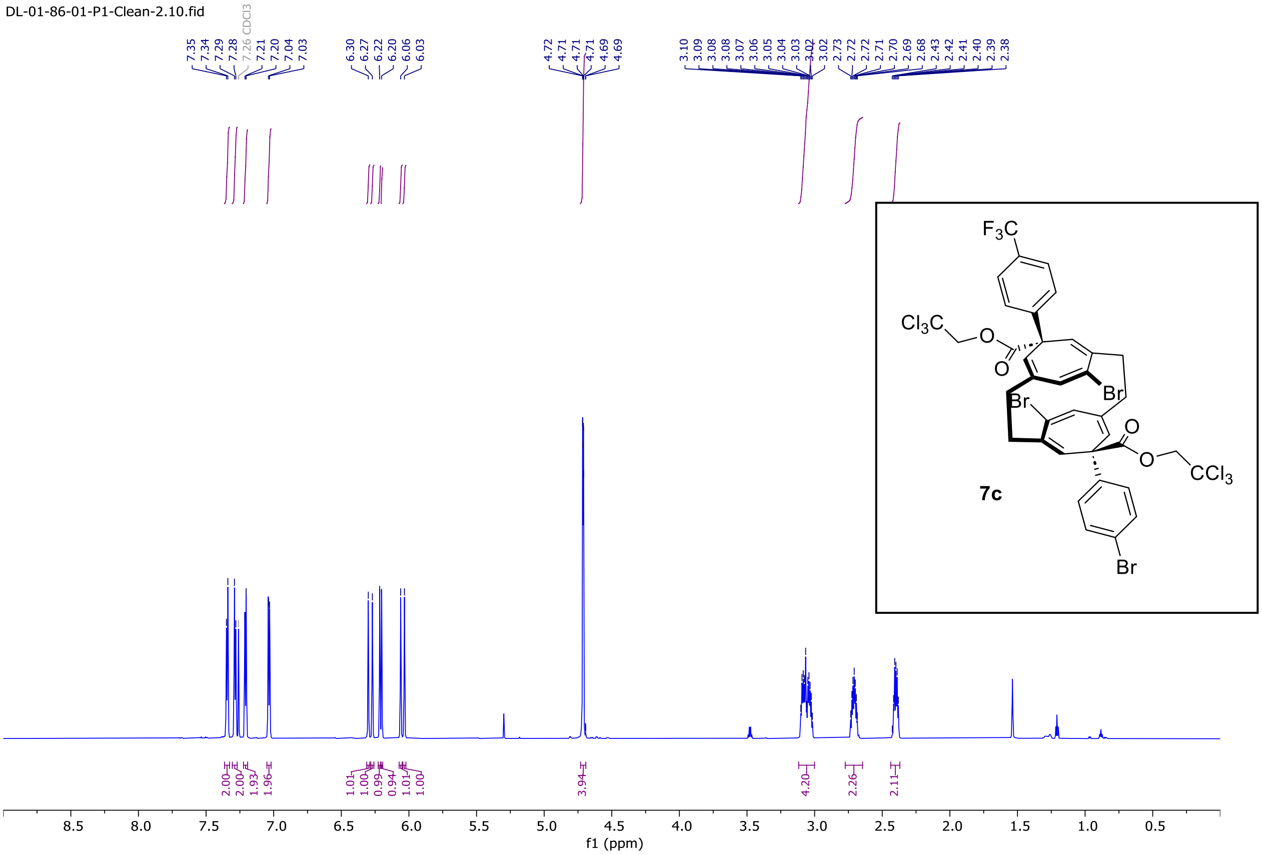

Figure S124. ^{1}**H-NMR of 7c**

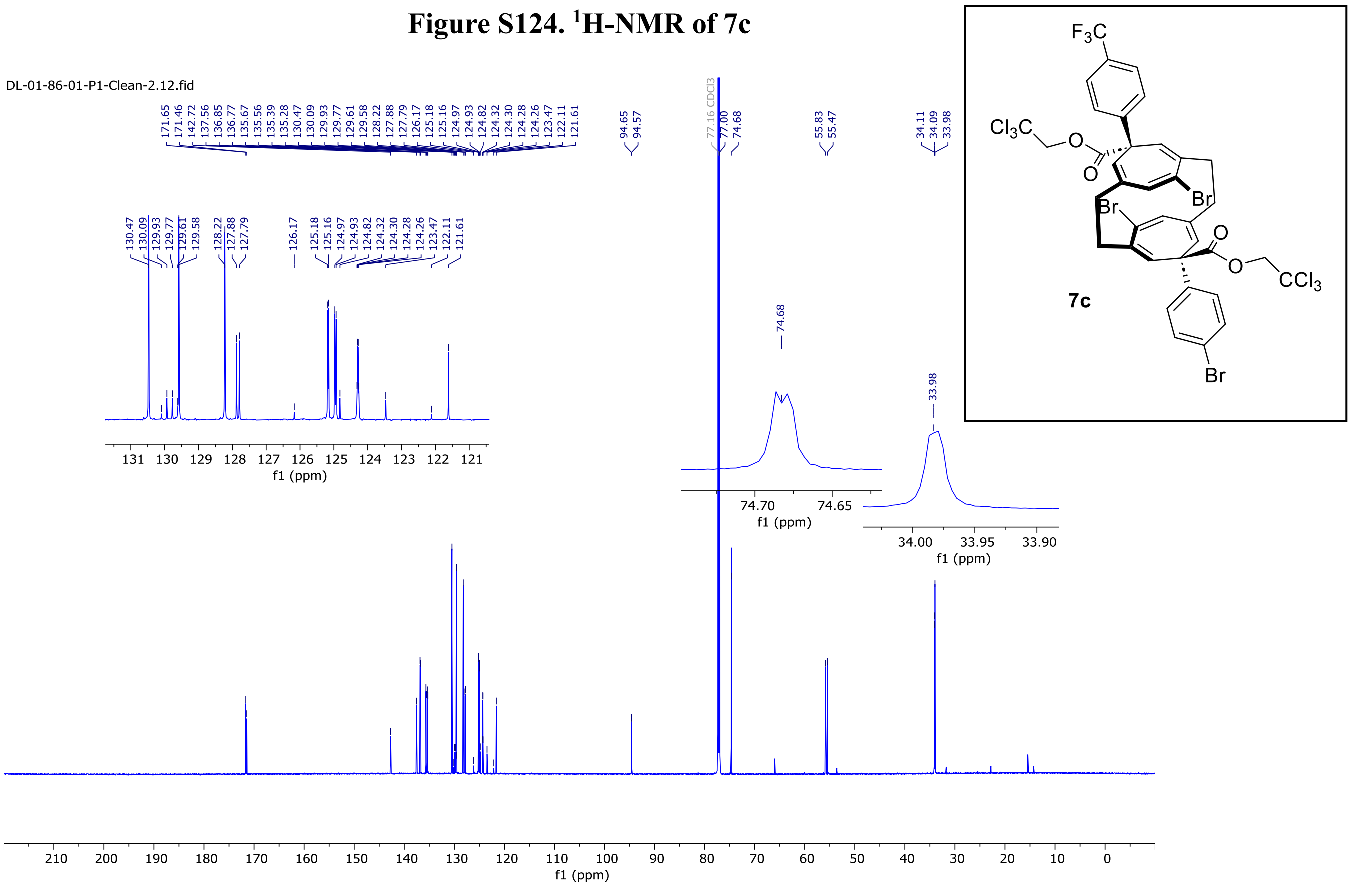

Figure S125. ^{13}**C-NMR of 7c**

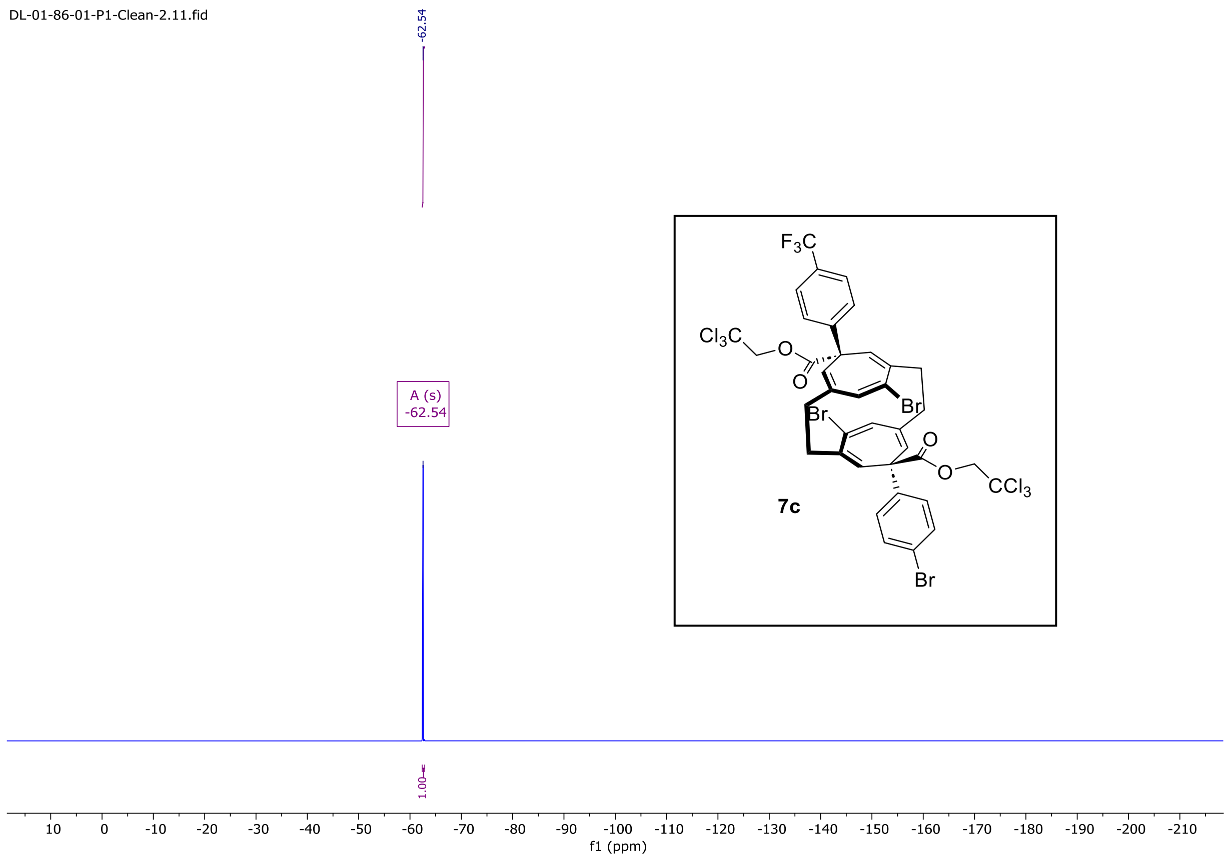

Figure S126. ^{19}F-NMR of 7c

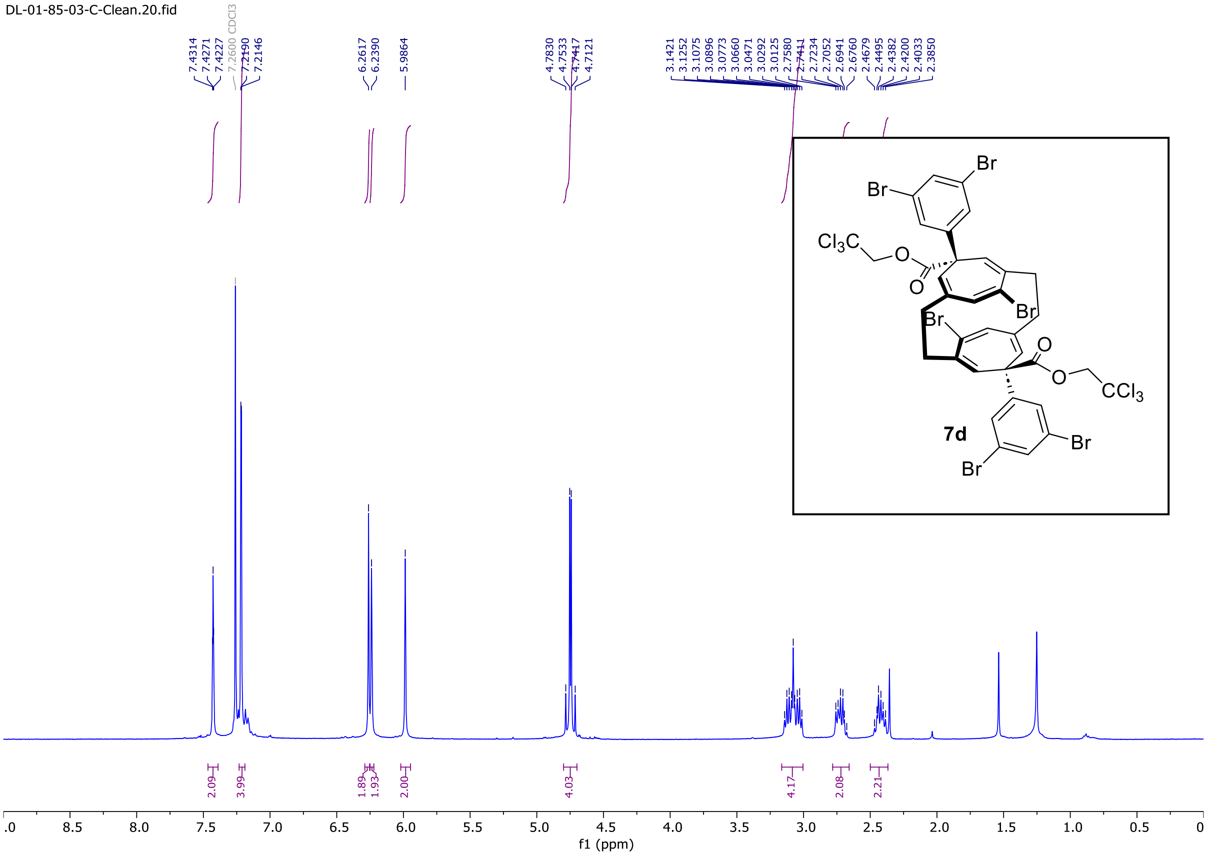

Figure S127. ^{1}**H-NMR of 7d**

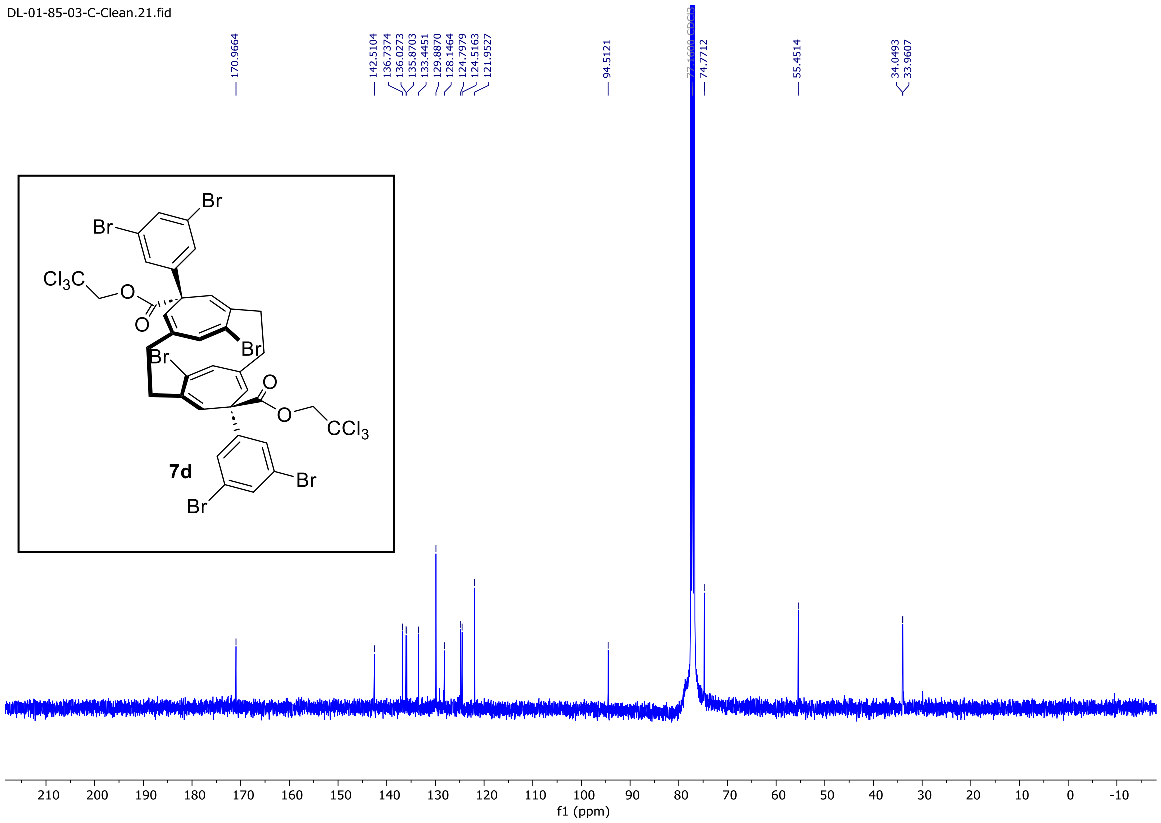

Figure S128. ^{13}**C-NMR of 7d**

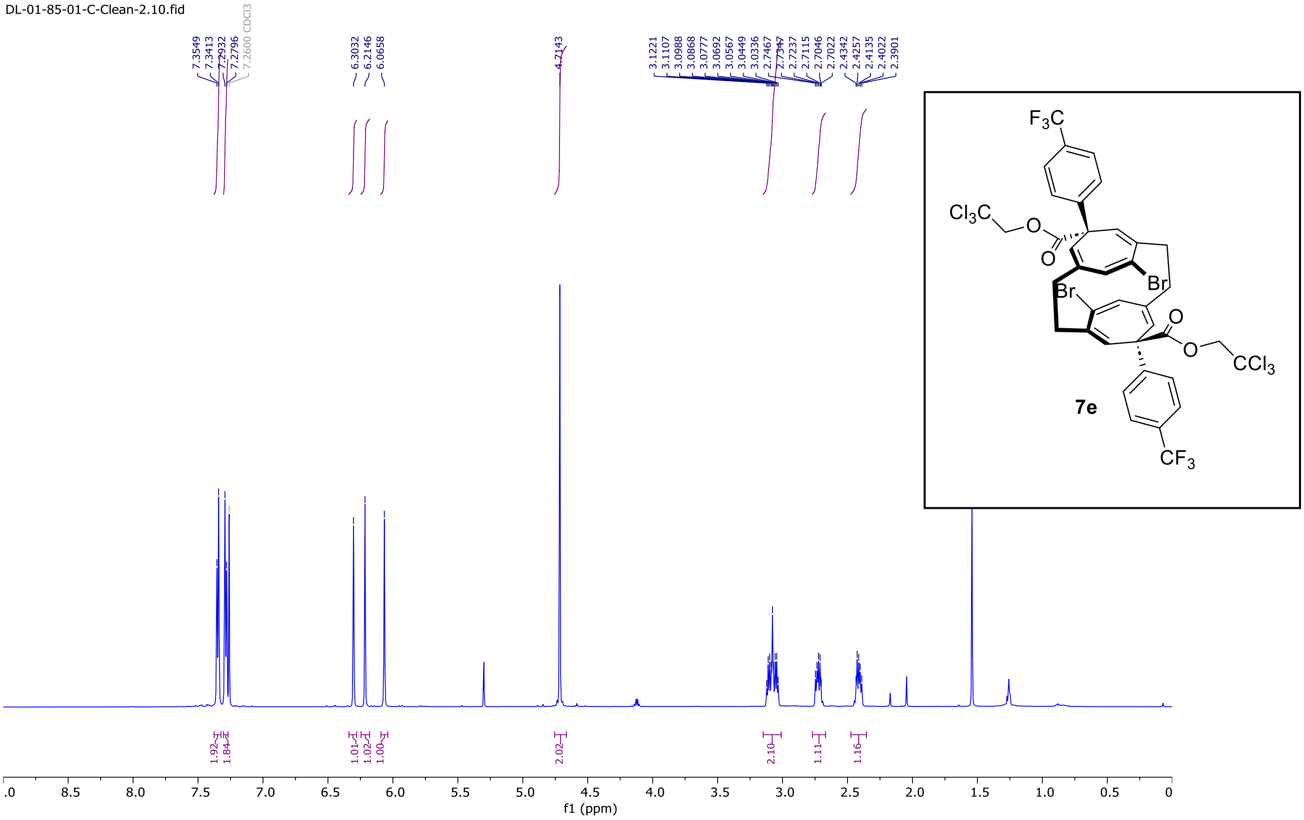

Figure S129. ^{1}**H-NMR of 7e**

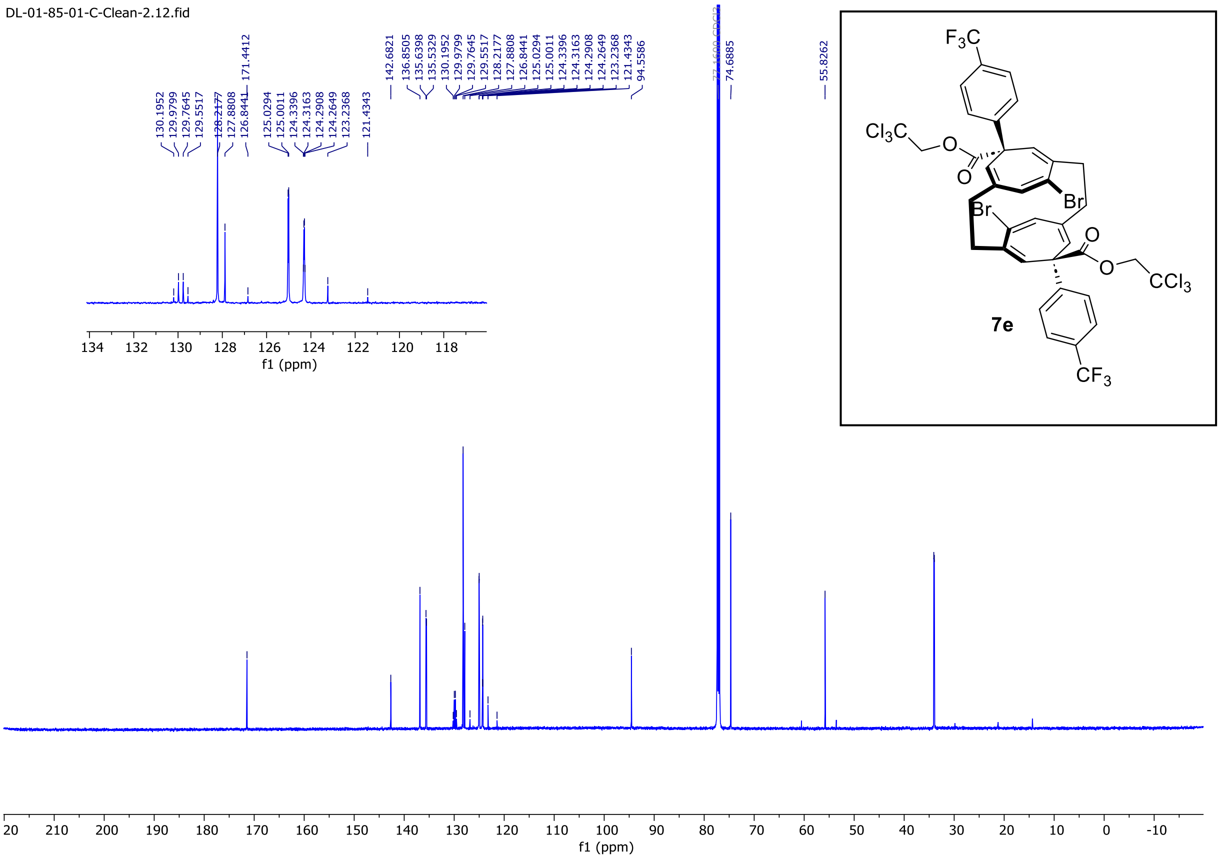

Figure S130. ^{13}**C-NMR of 7e**

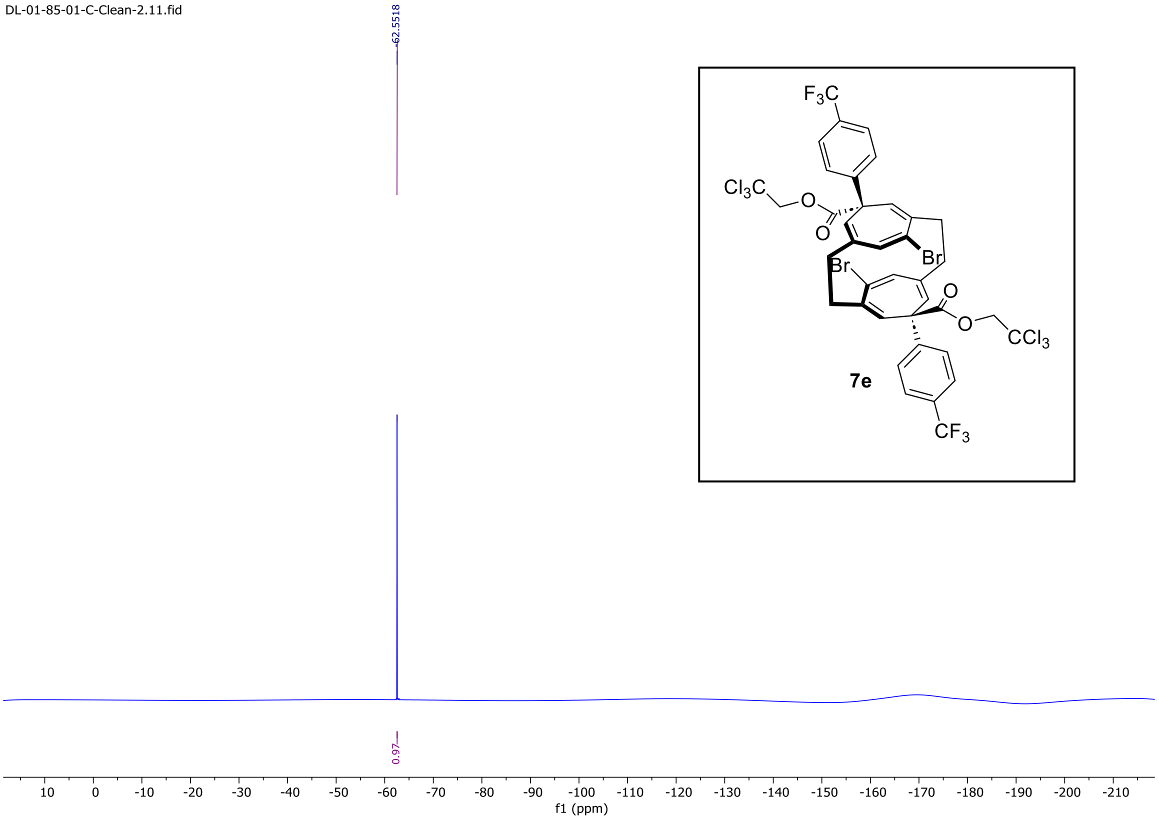

Figure S131. ^{19}**F-NMR of 7e**

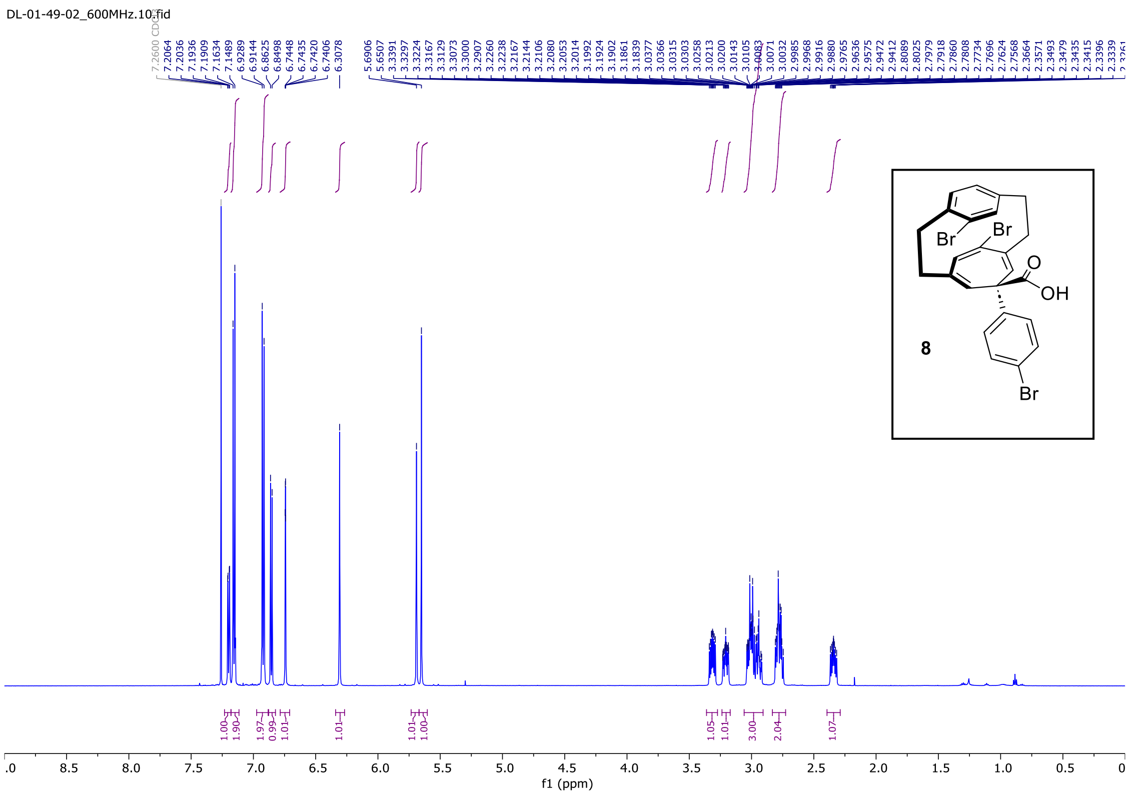

Figure S132. ^{1}H-NMR of 8

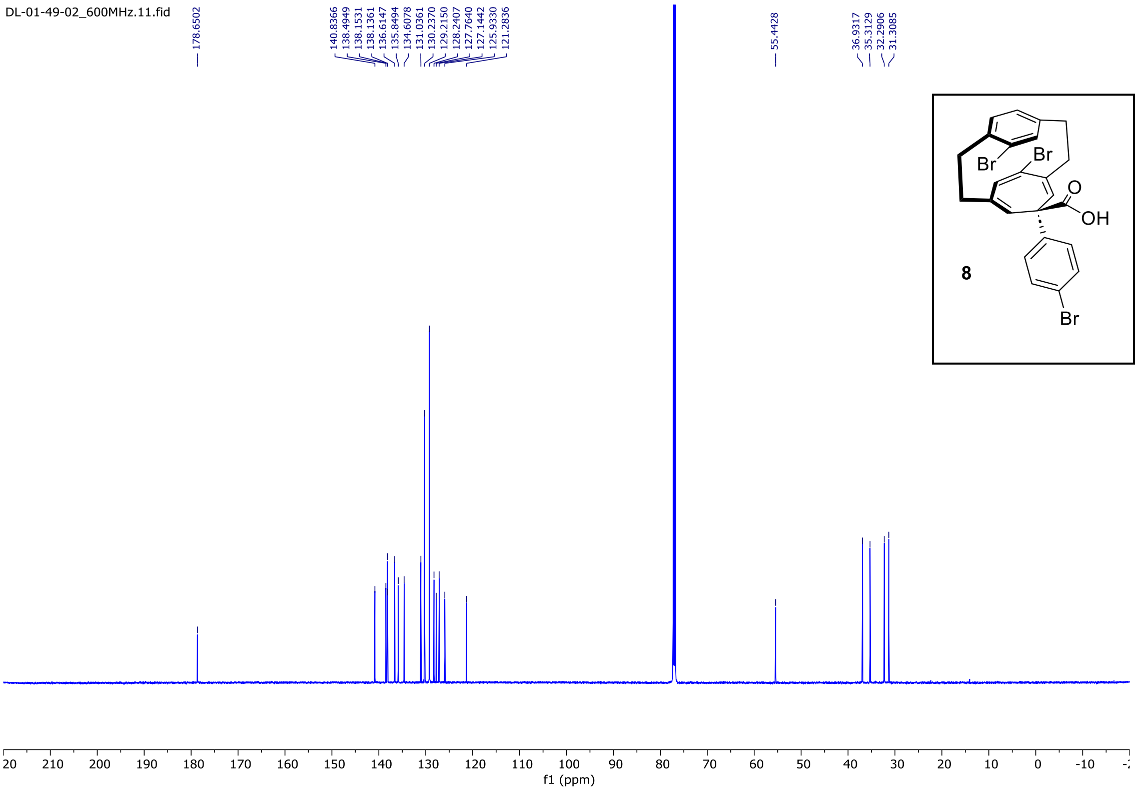

Figure S133. ^{13}C-NMR of 8

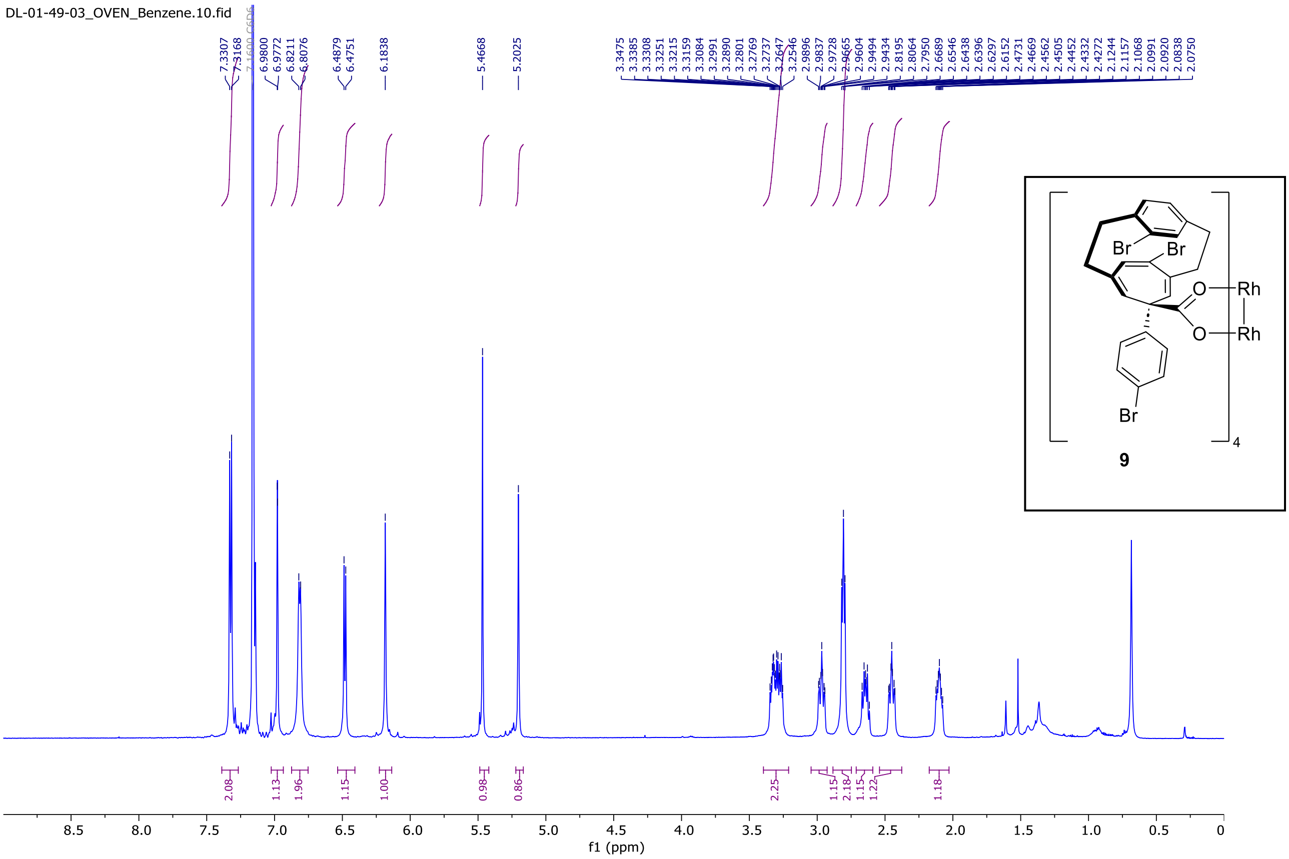

Figure S134. ¹H-NMR of 9

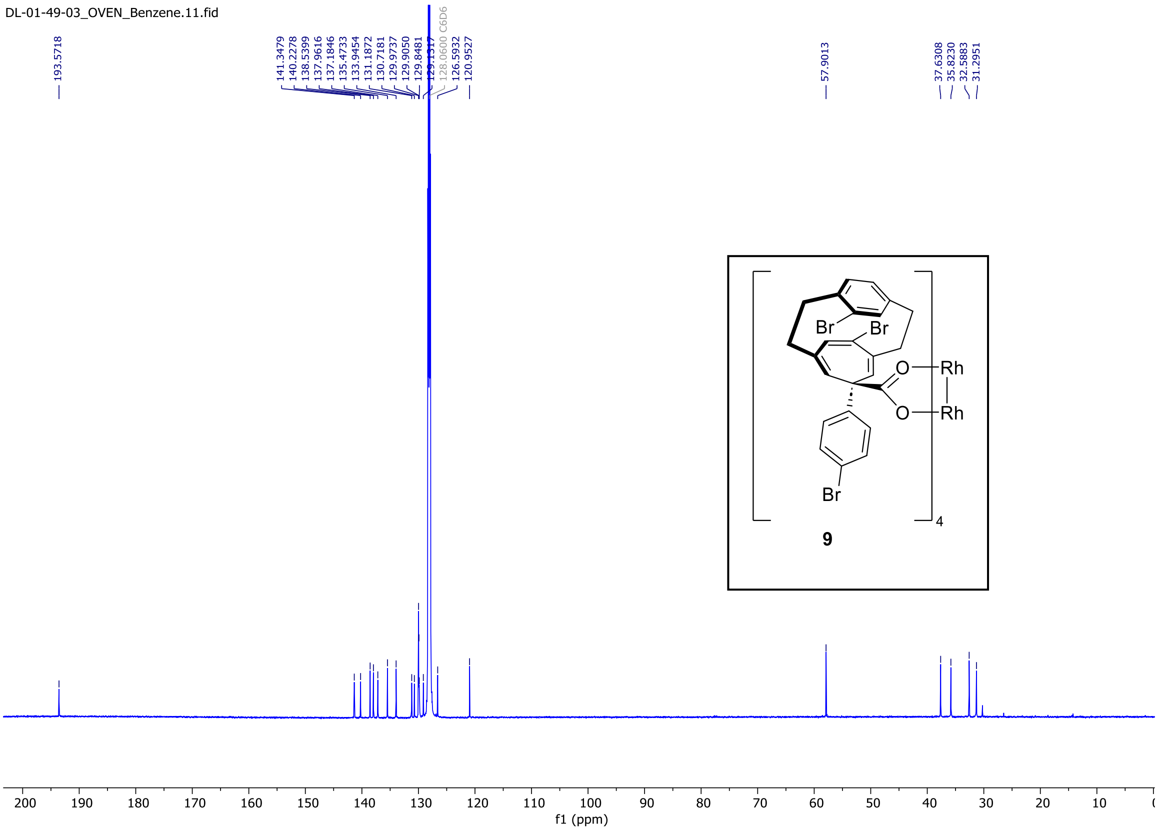

Figure S135. ¹³C-NMR of 9

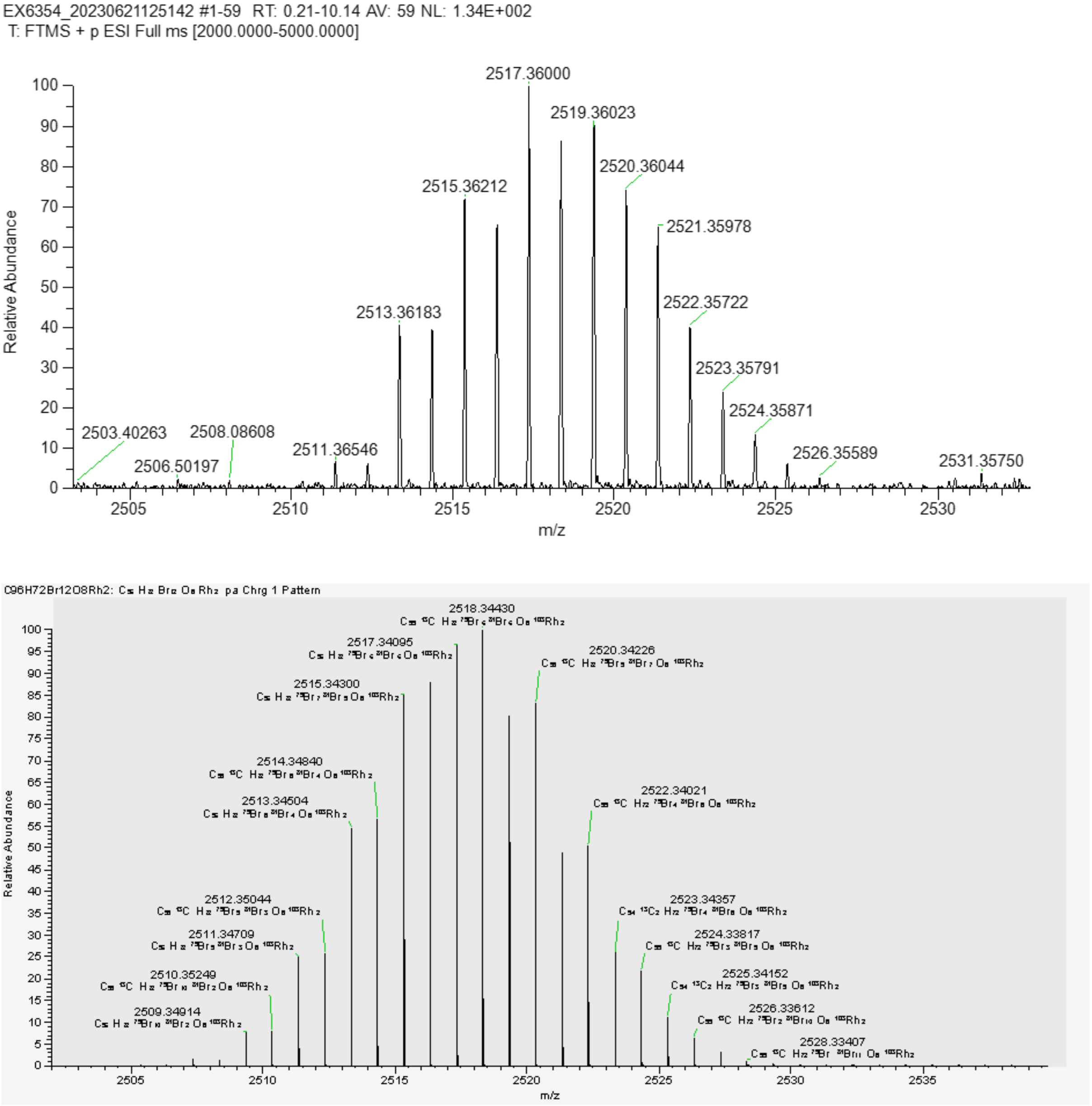

Figure S136. HRMS of 9

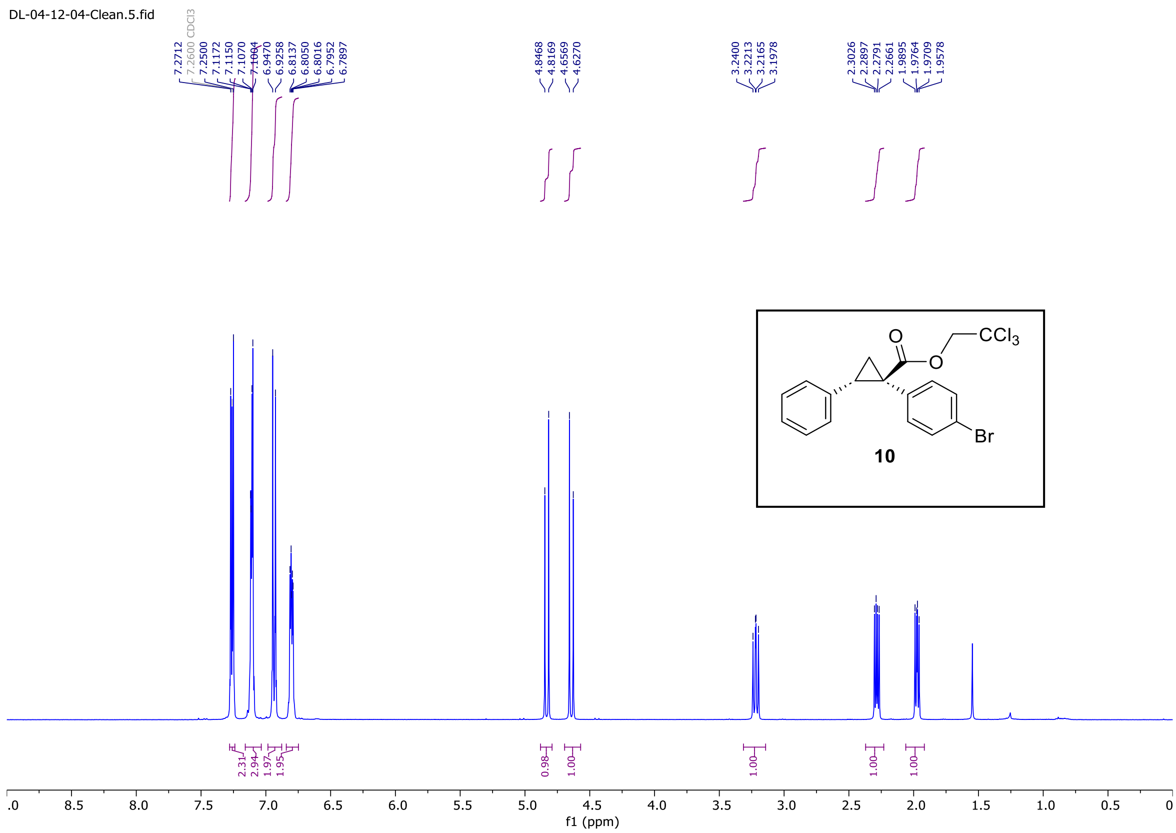

Figure S137. ^{1}H-NMR of 10

13. X-Ray Crystallographic data

**Compound**	**3aa**
Formula	$C_{26}H_{22}BrCl_3O_2$
$D_{calc.}$/ g cm^{-3}	1.588
μ/mm^{-1}	2.147
Formula Weight	552.724
Color	colorless
Shape	plate-shaped
Size/mm^3	0.73×0.33×0.08
T/K	100.0(4)
Crystal System	monoclinic
Space Group	$C2/c$
a/Å	20.8818(8)
b/Å	12.5085(4)
c/Å	19.6654(9)
$\alpha/^{\circ}$	90
$\beta/^{\circ}$	115.785(5)
$\gamma/^{\circ}$	90
V/Å^3	4625.2(4)
Z	8
Z'	1
Wavelength/Å	0.71073
Radiation type	Mo K_{α}
$\Theta_{min}/^{\circ}$	2.38
$\Theta_{max}/^{\circ}$	33.10
Measured Refl's.	53835
Indep't Refl's	8098
Refl's I≥2 σ(I)	6446
R_{int}	0.0692
Parameters	712
Restraints	1097
Largest Peak	0.8711
Deepest Hole	-0.6574
GooF	1.0289
wR_2 (all data)	0.0557
wR_2	0.0519
R_1 (all data)	0.0490
R_1	0.0312

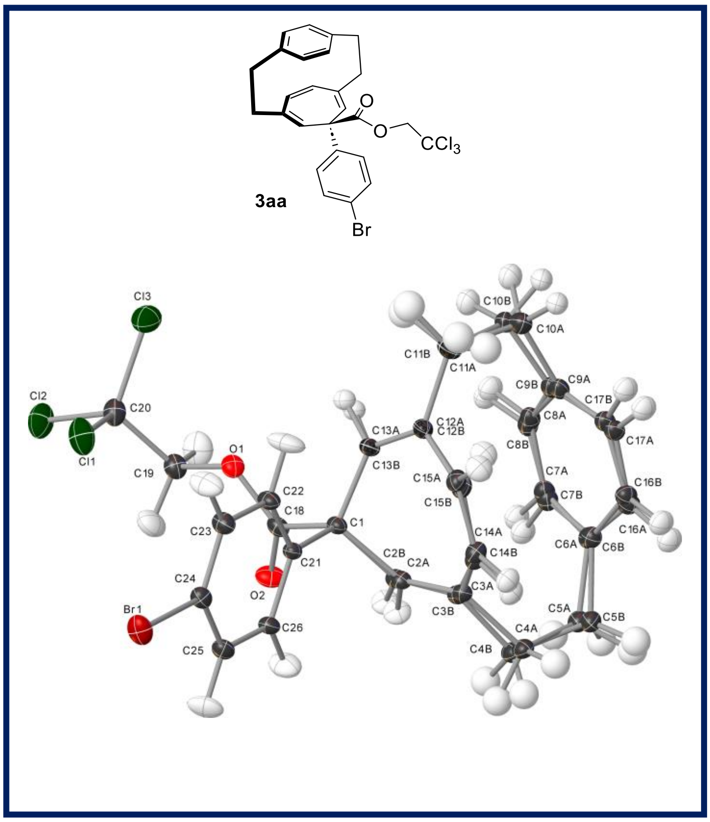

Structure Quality Indicators

**Reflections:**	d min (MoKα) 2Θ=66.2° 0.65	I/σ(I) 22.0	Rint m=6.75 6.92%	Full 50.5° 92% to 66.2° 99.1
**Refinement:**	Shift -0.005	Max Peak 0.9	Min Peak -0.7	GooF 1.029

**Compound**	**3da**
Formula	$C_{26}H_{20}Br_3Cl_3O_2$
$D_{calc.}$/ g cm^{-3}	1.809
μ/mm^{-1}	4.969
Formula Weight	710.515
Color	colorless
Shape	block-shaped
Size/mm^3	0.48×0.37×0.18
T/K	100.00(10)
Crystal System	monoclinic
Flack Parameter	0.002(2)
Hooft Parameter	0.002(2)
Space Group	$P2_1$
a/Å	10.1086(5)
b/Å	10.1442(3)
c/Å	13.5286(7)
α/°	90
β/°	109.907(5)
γ/°	90
V/Å^3	1304.38(11)
Z	2
Z'	1
Wavelength/Å	0.71073
Radiation type	Mo K_α
Θ_{min}/°	2.94
Θ_{max}/°	38.78
Measured Refl's.	52483
Indep't Refl's	13495
Refl's I≥2 σ(I)	11661
R_{int}	0.0468
Parameters	688
Restraints	430
Largest Peak	0.5739
Deepest Hole	-0.5293
GooF	0.9870
wR_2 (all data)	0.0411
wR_2	0.0395
R_1 (all data)	0.0405
R_1	0.0298

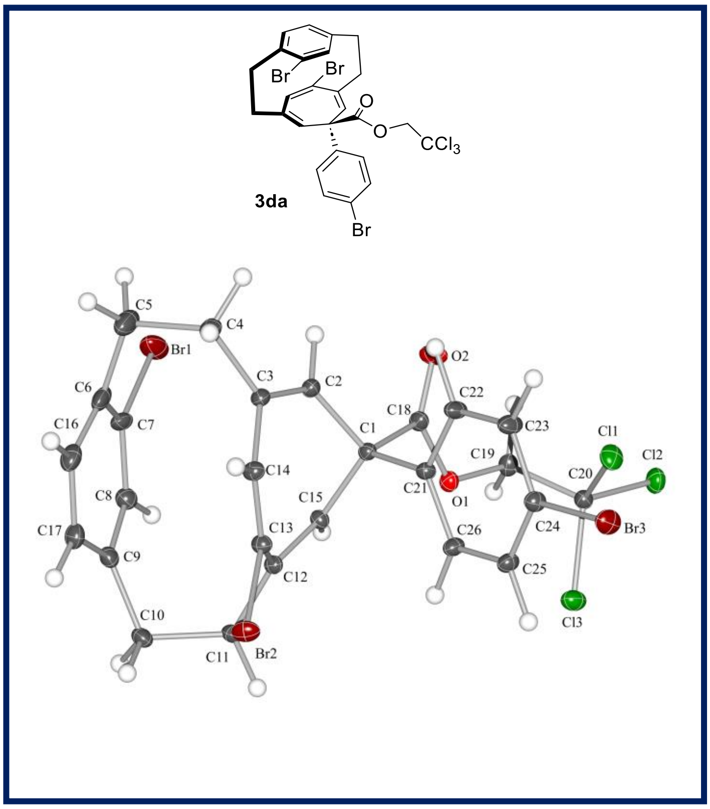

Structure Quality Indicators

Reflections: d min (MoKα) 2Θ=77.6° 0.57 | I/σ(I) 20.1 | Rint m=3.89 4.68% | Full 50.5° 92% to 77.6° 98.4

Refinement: Shift 0.001 | Max Peak 0.6 | Min Peak -0.5 | GooF 0.987 | Hooft .002(2)

**Compound**	**3fa**
Formula	$C_{26}H_{20}BrCl_5O_2$
$D_{calc.}$/ g cm^{-3}	1.594
μ/mm^{-1}	7.098
Formula Weight	621.614
Color	colorless
Shape	plate-shaped
Size/mm^3	0.20×0.17×0.10
T/K	173.0(2)
Crystal System	orthorhombic
Flack Parameter	-0.018(3)
Hooft Parameter	-0.018(3)
Space Group	$P2_12_12_1$
a/Å	10.19212(8)
b/Å	13.93645(13)
c/Å	18.23321(15)
α/°	90
β/°	90
γ/°	90
V/Å^3	2589.88(4)
Z	4
Z'	1
Wavelength/Å	1.54184
Radiation type	Cu K$_\alpha$
Θ_{min}/°	4.85
Θ_{max}/°	72.22
Measured Refl's.	68525
Indep't Refl's	4985
Refl's I≥2 σ(I)	4890
R_{int}	0.0318
Parameters	513
Restraints	408
Largest Peak	0.1560
Deepest Hole	-0.1237
GooF	1.0801
wR_2 (all data)	0.0226
wR_2	0.0225
R_1 (all data)	0.0136
R_1	0.0127

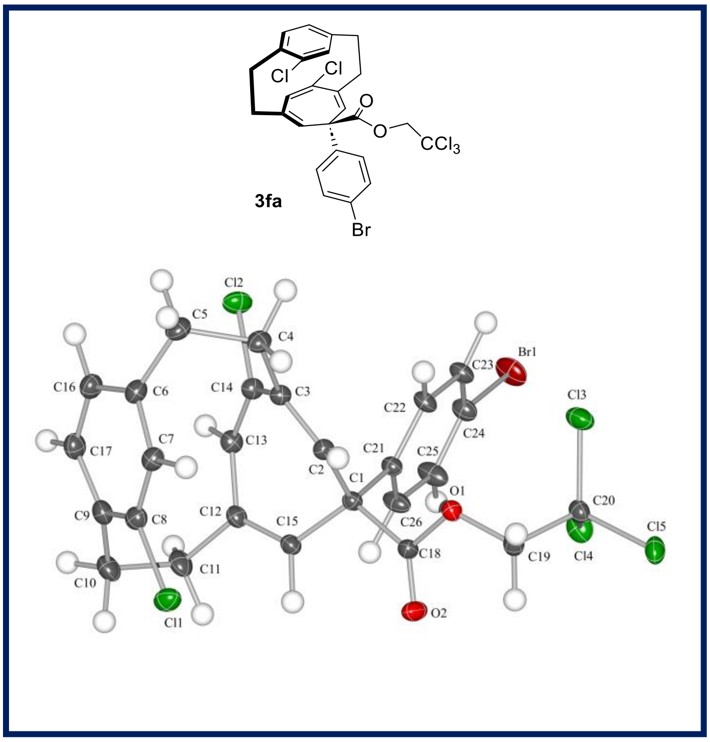

Structure Quality Indicators

**Reflections:**	d min (CuKα) 2Θ=144.4° 0.81	I/σ(I) 71.3	Rint m=13.75 3.18%	Full 135.4° 97% to 144.4° 97.7	
**Refinement:**	Shift -0.000	Max Peak 0.2	Min Peak -0.1	GooF 1.080	Hooft -.018(3)

**Compound**	**3ha**
Formula	$C_{46}H_{46}BrCl_3O_2$
$D_{calc.}$/ g cm^{-3}	1.321
μ/mm^{-1}	3.437
Formula Weight	817.138
Color	colorless
Shape	plate-shaped
Size/mm^3	0.12×0.05×0.05
T/K	173.04(18)
Crystal System	triclinic
Flack Parameter	-0.021(4)
Hooft Parameter	-0.021(4)
Space Group	P1
a/Å	11.1786(2)
b/Å	13.4151(4)
c/Å	16.0095(5)
α/°	65.847(3)
β/°	83.948(2)
γ/°	69.785(2)
V/Å^3	2053.89(11)
Z	2
Z'	2
Wavelength/Å	1.54184
Radiation type	Cu K$_\alpha$
Θ_{min}/°	3.03
Θ_{max}/°	72.30
Measured Refl's.	76231
Indep't Refl's	14204
Refl's I≥2 σ(I)	12421
R_{int}	0.0428
Parameters	1682
Restraints	1758
Largest Peak	0.3053
Deepest Hole	-0.3048
GooF	1.0511
wR_2 (all data)	0.0676
wR_2	0.0644
R_1 (all data)	0.0403
R_1	0.0323

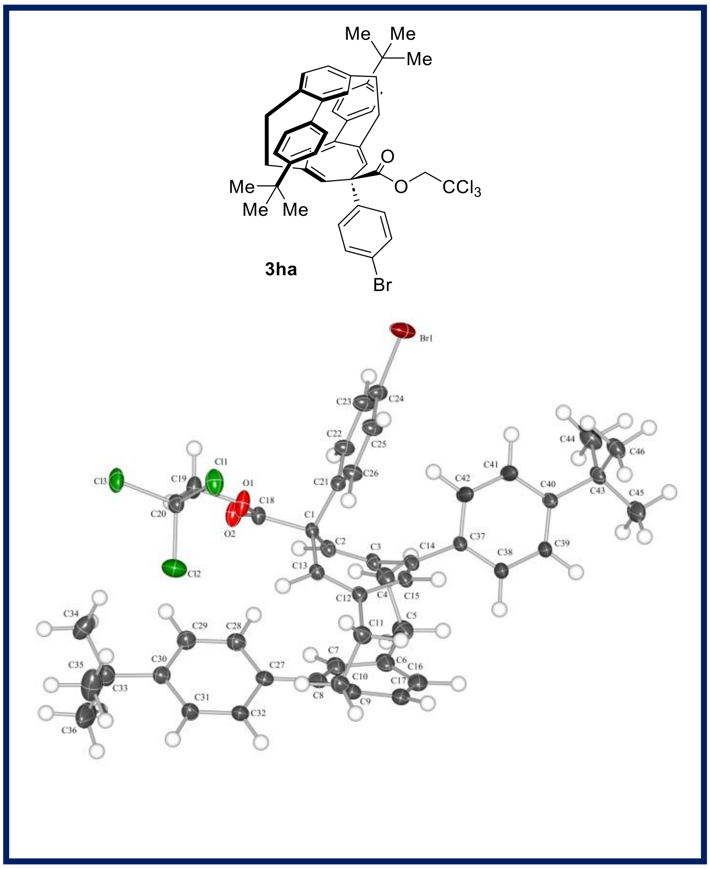

Structure Quality Indicators

Reflections: d min (CuKα) 2Θ=144.6° 0.81 | I/σ(I) 29.2 | Rint m=5.33 4.28% | Full 135.4° 96% to 144.6° 97.6

Refinement: Shift 0.007 | Max Peak 0.3 | Min Peak -0.3 | GooF 1.051 | Hooft -.021(4)

**Compound**	**8**
Formula	$C_{27.5}H_{28}Br_3O_2$
$D_{calc.}$/ g cm^{-3}	1.654
μ/mm^{-1}	6.084
Formula Weight	630.238
Colour	colourless
Shape	plate-shaped
Size/mm^3	0.48×0.35×0.15
T/K	100.15
Crystal System	monoclinic
Flack Parameter	-0.009(12)
Hooft Parameter	-0.009(12)
Space Group	$P2_1$
a/Å	8.56462(8)
b/Å	13.21683(13)
c/Å	22.4399(2)
α/°	90
β/°	94.8828(9)
γ/°	90
V/Å^3	2530.91(5)
Z	4
Z'	2
Wavelength/Å	1.54184
Radiation type	Cu K$_\alpha$
Θ_{min}/°	3.95
Θ_{max}/°	70.07
Measured Refl's.	51776
Indep't Refl's	9449
Refl's I≥2 σ(I)	9174
R_{int}	0.0842
Parameters	564
Restraints	650
Largest Peak	0.8752
Deepest Hole	-0.5227
GooF	1.0352
wR_2 (all data)	0.1208
wR_2	0.1196
R_1 (all data)	0.0487
R_1	0.0475

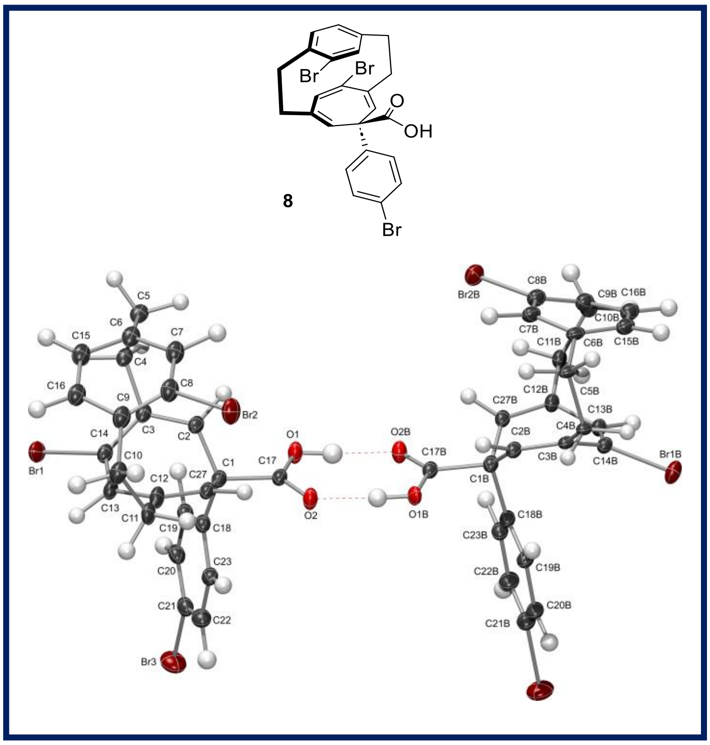

Structure Quality Indicators

**Reflections:**	d min (Cu\a) 2Θ=140.1° 0.82	I/σ(I) 20.0	Rint m=5.40 8.42%	Full 135.4° 98.3	
**Refinement:**	Shift 0.008	Max Peak 0.9	Min Peak -0.5	GooF 1.035	Hooft -.009(12)

**Compound**	DL-01-49-03
Formula	$C_{102}H_{82}Br_{12}Cl_6O_{10}Rh_2$
$D_{calc.}$/ g cm^{-3}	1.783
μ/mm^{-1}	9.656
Formula Weight	2845.11
Colour	green
Shape	prism-shaped
Size/mm^3	0.08×0.05×0.04
T/K	99.98(10)
Crystal System	orthorhombic
Flack Parameter	0.032(14)
Hooft Parameter	0.032(14)
Space Group	$P2_12_12$
a/Å	14.9255(10)
b/Å	20.5981(7)
c/Å	17.2404(8)
$\alpha/^\circ$	90
$\beta/^\circ$	90
$\gamma/^\circ$	90
V/Å^3	5300.3(5)
Z	2
Z'	0.5
Wavelength/Å	1.54184
Radiation type	Cu K_α
$\Theta_{min}/^\circ$	3.343
$\Theta_{max}/^\circ$	65.089
Measured Refl's.	34887
Indep't Refl's	9001
Refl's I≥2 σ(I)	6473
R_{int}	0.1246
Parameters	548
Restraints	542
Largest Peak	1.172
Deepest Hole	-0.766
GooF	1.031
wR_2 (all data)	0.1723
wR_2	0.1576
R_1 (all data)	0.0941
R_1	0.0672

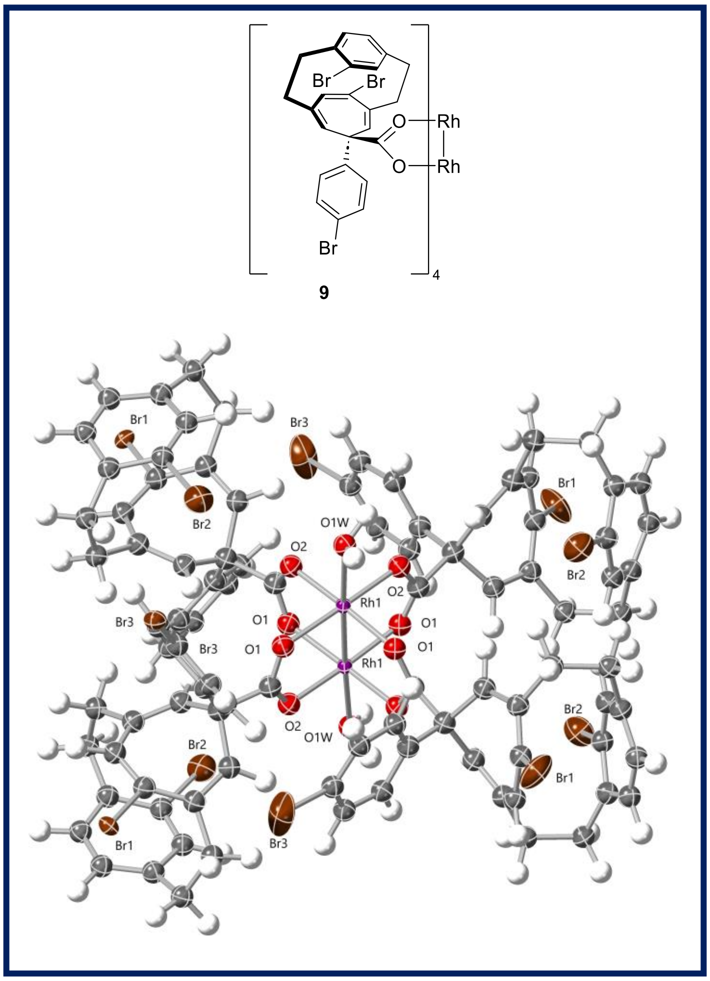

Structure Quality Indicators

**Reflections:**	d min (CuKα) 2Θ=130.2° 0.85	I/σ(I) 9.4	Rint m=3.88 12.46%	Full 130.2° 99.4	
**Refinement:**	Shift -0.001	Max Peak 1.2	Min Peak -0.8	GooF 1.031	Hooft .032(14)

14. Reference

(1) Davies, H. M. L.; Bruzinski, P. R.; Lake, D. H.; Kong, N.; Fall, M. J. Asymmetric Cyclopropanations by Rhodium(II) N-(Arylsulfonyl)prolinate Catalyzed Decomposition of Vinyldiazomethanes in the Presence of Alkenes. Practical Enantioselective Synthesis of the Four Stereoisomers of 2-Phenylcyclopropan-1-amino Acid. *J. Am. Chem. Soc.* **1996**, *118*, 6897-6907. DOI: 10.1021/ja9604931.

(2) Fu, J.; Ren, Z.; Bacsa, J.; Musaev, D. G.; Davies, H. M. L. Desymmetrization of cyclohexanes by site- and stereoselective C–H functionalization. *Nature* **2018**, *564* , 395-399. DOI: 10.1038/s41586-018-0799-2.

(3) Müller, P.; Allenbach, Y.; Robert, E. Rhodium(II)-catalyzed olefin cyclopropanation with the phenyliodonium ylide derived from Meldrum's acid. *Tetrahedron: Asymmetry* **2003**, *14*, 779-785. DOI: https://doi.org/10.1016/S0957-4166(03)00029-6.

(4) Reddy, R. P.; Davies, H. M. L. Dirhodium Tetracarboxylates Derived from Adamantylglycine as Chiral Catalysts for Enantioselective C−H Aminations. *Org. Lett.* **2006**, *8* (22), 5013-5016. DOI: 10.1021/ol061742l.

(5) Qin, C.; Davies, H. M. L. Role of Sterically Demanding Chiral Dirhodium Catalysts in Site-Selective C–H Functionalization of Activated Primary C–H Bonds. *J. Am. Chem. Soc.* **2014**, *136*, 9792-9796. DOI: 10.1021/ja504797x.

(6) Mori, W.; Hoshino, H.; Nishimoto, Y.; Takamizawa, S. Synthesis and Gas Occlusion of New Micropore Substance Rhodium(II) Carboxylates Bridged by Pyrazine. *Chem. Lett.* **2003**, *28*, 331-332. DOI: 10.1246/cl.1999.331 (acccessed 3/26/2024).

(7) Guptill, D. M.; Davies, H. M. L. 2,2,2-Trichloroethyl Aryldiazoacetates as Robust Reagents for the Enantioselective C–H Functionalization of Methyl Ethers. *J. Am. Chem. Soc.* **2014**, *136*, 17718-17721. DOI: 10.1021/ja5107404.

(8) Fu, L.; Mighion, J. D.; Voight, E. A.; Davies, H. M. L. Synthesis of 2,2,2,-Trichloroethyl Aryl- and Vinyldiazoacetates by Palladium-Catalyzed Cross-Coupling. *Chem. Eur. J.* **2017**, *23*, 3272-3275. DOI: https://doi.org/10.1002/chem.201700101.

(9) Garlets, Z. J.; Hicks, E. F.; Fu, J.; Voight, E. A.; Davies, H. M. L. Regio- and Stereoselective Rhodium(II)-Catalyzed C–H Functionalization of Organosilanes by Donor/Acceptor Carbenes Derived from Aryldiazoacetates. *Org. Lett.* **2019**, *21*, 4910-4914. DOI: 10.1021/acs.orglett.9b01833.

(10) Braddock, D. C.; MacGilp, I. D.; Perry, B. G. Improved Synthesis of (±)-4,12-Dihydroxy[2.2]paracyclophane and Its Enantiomeric Resolution by Enzymatic Methods: Planar Chiral (R)- and (S)-Phanol. *J. Org. Chem.* **2002**, *67*, 8679-8681. DOI: 10.1021/jo020451x.

(11) Bier, A. K.; Bognitzki, M.; Mogk, J.; Greiner, A. Synthesis, Structure, and Properties of Alkyl-Substituted PPXs by Chemical Vapor Deposition for Stent Coatings. *Macromolecules* **2012**, *45*, 1151-1157. DOI: 10.1021/ma202270w.

(12) Krasnova, I. Y.; Antonov, D. Y.; Shapovalov, A. V.; Shifrina, Z. B. Dendritic polyphenylene framework as a light-harvesting shell for highly emissive [2.2]Paracyclophane core. *Polymer* **2021**, *234*, 124227. DOI: https://doi.org/10.1016/j.polymer.2021.124227.

(13) Shinmyozu, T.; Hirai, Y.; Inazu, T. Synthesis of [3.3]heterophanes containing the pyridine, furan, and thiophene rings by the TosMIC method. *J. Org. Chem.* **1986**, *51*, 1551-1555. DOI: 10.1021/jo00359a033.

(14) Yu, S.; Bao, H.; Zhang, D.; Yang, X. Kinetic resolution of substituted amido[2.2]paracyclophanes via asymmetric electrophilic amination. *Nat. Commun.* **2023**, *14*, 5239. DOI: 10.1038/s41467-023-40718-8.

(15) Wu, Y.; Zhuang, G.; Cui, S.; Zhou, Y.; Wang, J.; Huang, Q.; Du, P. Through-space π-delocalization in a conjugated macrocycle consisting of [2.2]paracyclophane. *Chem. Commun.* **2019**, *55*, 14617-14620. DOI: 10.1039/C9CC06492C.

(16) Sasaki, H.; Kitagawa, T. Synthesis of [3ⁿ]Cyclophanes and Related Compounds by Alkylation of Tosylmethyl Isocyanide with Bis (bromomethyl) benzenes. *Chem. Pharm. Bull.* **1983**, *31*, 2868-2878. DOI: 10.1248/cpb.31.2868.

(17) Sailer, J. K.; Sharland, J. C.; Bacsa, J.; Harris, C. F.; Berry, J. F.; Musaev, D. G.; Davies, H. M. L. Diruthenium Tetracarboxylate-Catalyzed Enantioselective Cyclopropanation with Aryldiazoacetates. *Organometallics* **2023**, *42*, 2122-2133. DOI: 10.1021/acs.organomet.3c00268.
